# Mapping inequalities in health service coverage in Africa: a scoping review

**DOI:** 10.1136/bmjopen-2023-082918

**Published:** 2024-11-24

**Authors:** Humphrey Cyprian Karamagi, Doris Osei Afriyie, Ali Ben Charif, Sokona Sy, Hillary Kipruto, Thandelike Moyo, Taiwo Oyelade, Benson Droti

**Affiliations:** 1 World Health Organization Regional Office for Africa, Brazzaville, Congo; 2 Epidemiology and Public Health, Swiss Tropical and Public Health Institute, Allschwil, Switzerland; 3 University of Basel, Basel, Switzerland; 4 CubecXpert, Quebec City, Quebec, Canada; 5 Health Systems & Services, World Health Organization, Harare, Kenya; 6 World Health Organization Regional Office for Africa, Brazzaville, South Africa; 7 World Health Organization, Geneva, Switzerland

**Keywords:** PUBLIC HEALTH, Health Services, Health Equity

## Abstract

**Abstract:**

**Objective:**

In this scoping review, we aim to consolidate the evidence on inequalities in service coverage in Africa using a comprehensive set of stratifiers. These stratifiers include place of residence, race/ethnicity/culture/language, occupation, gender/sex, religion, education, socioeconomic status and social capital. Our approach provides a more holistic understanding of the different dimensions of inequality in the context of universal health coverage (UHC).

**Design:**

We conducted a scoping review following the Joanna Briggs Institute Manual for Evidence Synthesis.

**Data sources:**

We searched MEDLINE, Embase, Web of Science, CINAHL, PyscINFO, Cochrane Library, Google Scholar and Global Index Medicus for articles published between 1 January 2005 and 29 August 2022 examining inequalities in utilisation of health services for reproductive, maternal, newborn and child health (RMNCH), infectious or non-communicable diseases in Africa.

**Eligibility criteria for selecting studies:**

We included any empirical research that assessed inequalities in relation to services for RMNCH (eg, family planning), infectious diseases (eg, tuberculosis treatment) and non-communicable diseases (eg, cervical cancer screening) in Africa.

**Data extraction and synthesis:**

The data abstraction process followed a stepwise approach. A pilot-tested form capturing study setting, inequality assessment and service coverage indicators was developed and finalised. Data were extracted by one reviewer and cross-checked by another, with discrepancies resolved through consensus meetings. If a consensus was not reached, senior reviewers made the final decision. We used a narrative approach to describe the study characteristics and mapped findings against PROGRESS-Plus stratifiers and health service indicators. Quantitative findings were categorised as ‘proequity’, ‘antiequity’ or ‘equal’ based on service utilisation across social groups.

**Results:**

We included 178 studies in our review, most studies published within the last 5 years (61.1%). Most studies assessed inequality using socioeconomic status (70.6%), followed by age (62.4%), education (60.7%) and place of residence (59.0%). Few studies focused on disability, social capital and ethnicity/race and intersectionality of stratifiers. Most studies were on RMNCH services (53.4%) and infectious disease services (43.3%). Few studies were qualitative or behavioural analyses. Results highlight significant inequalities across different equity stratifiers and services with inconsistent trends of inequalities over time after the implementation of strategies to increase demand of services and strengthen health systems.

**Conclusion:**

There is a need to examine equity in service coverage for a variety of health conditions among various populations beyond the traditional classification of social groups. This also requires using diverse research methods identifying disparities in service use and various barriers to care. By addressing these knowledge gaps, future research and health system reforms can support countries in moving closer to achievement of UHC targets.

**STRENGTHS AND LIMITATIONS OF THIS STUDY**

- A strength of our scoping review is bringing together a wide scope of studies using a comprehensive strategy.
- By applying the PROGRESS-Plus framework, we ensured a broader dimension of social process factors to uncover inequities in service utilisation.
- Adapting the WHO Universal Health Coverage service coverage indicators prevented us from exploring inequalities in the quality of services provided, as the coverage indicators are primarily focused on utilisation.
- Research was skewed towards certain drivers of health inequalities such as language and ethnicity.
- Our search strategy primarily included articles published in English due to resource constraints and the predominant use of English in the databases we searched.

## Introduction

 Achieving universal health coverage (UHC) is a key priority of the global agenda to improve health and well-being. UHC, the umbrella target for the third sustainable development goal (SDG), means ensuring that all populations have access to quality health services without experiencing financial hardship.^1 2^ Minimising the impact of inequalities on access to and utilisation of essential health services is a core expectation for achieving UHC, reflected in the SDG’s promise to ‘leave no one behind’. The impact of these inequalities contributes to the disparities in health outcomes observed within the African region.^3 4^

While the need to address these inequalities is shared by most health stakeholders, the problem has remained intractable. Current evidence suggests that while overall health and well-being are significantly improving in many countries, the differences in outcomes between different populations within countries have persisted in many instances.^5 6^

Efforts to address inequalities typically focused on specific services or addressing one of the inequality drivers: place of residence, race/ethnicity/culture/language, occupation, gender/sex, religion, education, socioeconomic status (SES) and social capital (‘PROGRESS’).^7^ Many efforts to address inequalities focus on one or a few of these inequality drivers. However, it is recognised that the ways in which these drivers act to address inequalities are complex, evolving and context-specific. The framing of health inequality is, therefore, not fit for purpose, as it takes a vertical siloed approach that does not take cognizance of the contextual needs and drivers. The primary healthcare (PHC) approach, which defines how health investments need to be made, requires a shift from a vertical issue-based approach to one centred around the needs of the individuals as beneficiaries of services.^8^ Person-centredness and sustainability need to be viewed as ontological to health inequality framing. In this paper, we explore what this means for countries in the African region.

We aim to provide information on the relative contribution of each driver of health inequalities to the variations in health outcomes for beneficiaries in the African Region. To achieve this, two questions are addressed: (1) What is the evidence on the contribution of each driver to reducing health inequalities in the African region? and (2) What drives this relative contribution? This information is crucial for policy and decision-makers as it provides them with evidence of the relative prioritisation they need to place on each driver of inequality given their health goals.

## Methods

### Scoping review methodology

We conducted a scoping review following the Joanna Briggs Institute (JBI) Manual for Evidence Synthesis, to explore what evidence exists for the contribution of each driver of health inequalities in Africa. This was done following the methodology recommended in the JBI Manual for Evidence Synthesis, a manual that provides guidance on developing systematic reviews^9^ and was reported according to the PRISMA (Preferred Reporting Items for Systematic reviews and Meta-Analyses).^10^ Our protocol was registered with the Open Science Framework (OSF) (identifier: https://osf.io/zd5bt), peer reviewed and published.^11^

The electronic search strategy (online supplemental appendix 1) was conducted on MEDLINE, Embase, Web of Science, CINAHL, PyscINFO and Cochrane Library from their dates of inception to 29 August 2022, in line with this protocol to identify studies of interest. We ensured the comprehensiveness of our search strategy by incorporating all relevant search terms and validated filters to reflect three core themes: (1) equity, (2) UHC and (3) African regions. We also expanded our review to include publicly available sources, such as information produced by governments at all levels, academic institutions and industry, both in print and electronic formats, that are not controlled by commercial publishers. Our search strategy was reviewed by an information specialist and our core research team of international experts in health equity, UHC, health information systems or knowledge syntheses from Africa reviewed the preliminary search strategy to ensure its thoroughness and relevance. We included any empirical research that assessed inequalities in relation to services for reproductive, maternal, newborn and child health (RMNCH) (eg, family planning), infectious diseases (eg, tuberculosis treatment) and non-communicable diseases (NCDs) (eg, cervical cancer screening) in Africa.^11^ Additionally, we performed a search to specifically capture studies on subnational health inequalities to ensure we included relevant work on the subnational burden of diseases. Recognising the importance of quality assessment in providing context to our findings, we conducted a preliminary appraisal of the included studies based on several criteria: study design, sample size, data collection methods and relevance to the research question. Studies with significant methodological flaws or those lacking direct relevance were excluded. For the remaining eligible studies, data were extracted using prepiloted, standardised forms (online supplemental table 1). Additionally, we excluded retracted publications, conference abstracts, study protocols and editorial materials (online supplemental appendix 2), as well as studies not directly relevant to health inequalities (online supplemental appendix 2). Only studies published from 1 January 2005 to 26 August 2022 were considered as 2005 marked the first introduction of UHC into public health discourse.^11^ Our search strategy primarily included English-language articles. However, we included studies indexed in databases that cover a wide range of languages, such as Global Index Medicus, which includes literature from various WHO regional databases, often in multiple languages. We calculated an inter-reviewer agreement using the weighted Cohen’s kappa.^12^

The data abstraction and presentation process followed a stepwise process. First, a data abstraction form that included information on study setting, information on how inequality was assessed and the context of the study and service coverage indicators was developed and pilot-tested by ABC, DOA and SS. Following this, the final agreed form was used to extract information from the identified reports by one single reviewer (ABC, DOA, SS or TM) and checked by another (ABC or DOA). Any discrepancies were resolved through consensus meetings between the reviewers (ABC, DOA, SS and TM). Where consensus could not be achieved, reports were reviewed by HK and HCK to decide on their inclusion.

After extracting information from the included reports, we described the main characteristics of studies using a narrative approach involving content analysis. We mapped included studies against the appropriate PROGRESS-Plus stratifiers and health service coverage indicators. We characterised quantitative findings for different social groups receiving or using services as ‘proequity’ (ie, higher service utilisation by worst-off groups), ‘antiequity’ (ie, higher service utilisation by better-off groups) or equal (no significant differences in groups receiving or using services).

### Patient and public involvement

None.

## Results

### Selection of studies

**Figure 1:**
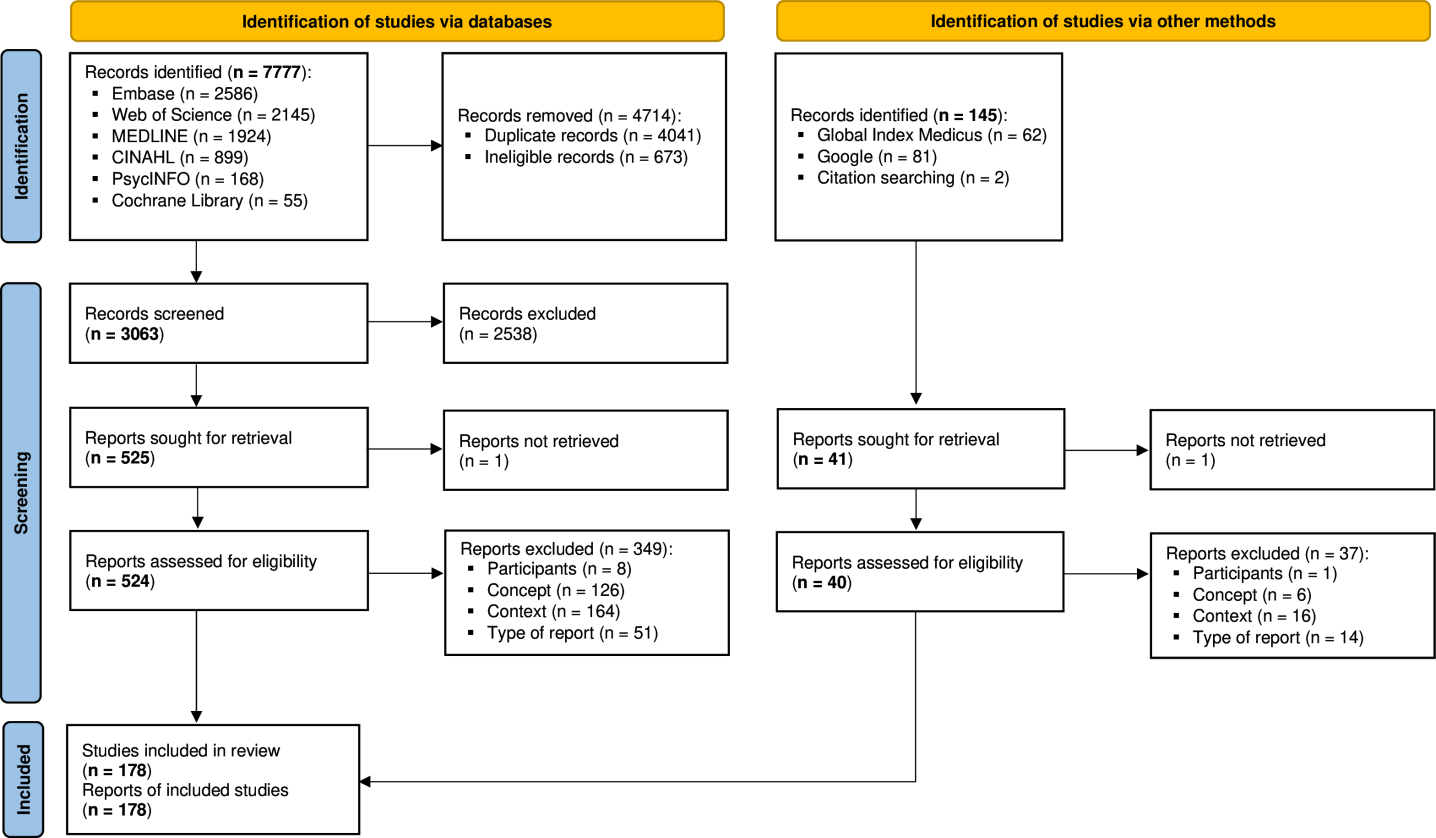
PRISMA 2020 flow diagram of the study inclusion process. PRISMA, Preferred Reporting Items for Systematic reviews and Meta-Analyses.

Our electronic search identified 7777 potentially relevant records. Of these, 4041 were duplicates and 673 were ineligible (because published before 2005), leaving 3063 records. The subsequent review of the 3063 records found 2538 did not meet the review criteria. Full-text eligibility review was, therefore, done on the 525 remaining reports. This remained 525 reports were reviewed for eligibility, 175 of which the met the inclusion criteria.^13^,^187^ An additional three articles^188^,^190^ were identified from other sources. Overall, 178 articles were included in the review (figure 1), as the others were outside the Africa geographical region, did not include a PROGRESS-plus driver or did not include a health service outcome indicator (see online supplemental appendix 3).

### Characteristics of included studies

Geographically, most of the studies involved single African countries (81.5%, n=145) with more than half of these studies from five countries: Nigeria (n=22), Ethiopia (n=17), Ghana (n=16), Uganda (n=13) and Tanzania (n=11). Many of the studies were published within the last 5 years (n=109, 61.2). Over three out of four studies were cross-sectional studies (n=141, 79.2%), followed by experimental or quasi-experimental (n=12, 6.7%), longitudinal studies (n=11, 6.2%) qualitative studies (n=5, 2.8%), knowledge syntheses (n=4, 2.2%), multipronged study designs (n=3, 1.7%) and mixed-methods studies (n=2, 1.1%). With data sources, over half (n=96, 53.9%) were from secondary sources, 42.1% (n=75) were from primary sources and 3.9% (n=7) were from both sources. Over half (n=92, 51.7%) of studies used national-level data, followed by subnational level (n=73, 41.0%), organisational level (n=8, 4.5%) and multiple levels (n=5, 2.8%). Characteristics of included studies are outlined in online supplemental table 1 and description of each included study can be found in online supplemental appendix 3.

### Description of inequality stratifiers

Most of the studies assessed inequality using more than one PROGRESS-Plus stratifier (82.0% n=146) with a median of three. The most frequent stratifiers used were SES (n=125, 70.2%), age (n=111, 62.4%), education (n=108, 60.7%) and place of residence (n=105, 59.0%), with the least used stratifiers by studies being disability (n=3, 1.7%) and ethnicity/race (n=7, 3.9%).

### Description of health service coverage indicators

RMNCH services were the most common (n=98, 53.4%) followed by infectious disease services (n=77, 43.3%), NCD services (n=15, 8.4%) and composite indices (n=5, 2.8%). In terms of specific interventions, nearly one-third of studies focused on the use of long-lasting insecticidal nets (n=54, 30.3%), followed by antenatal care (n=47, 26.4%), family planning (n=37, 20.8%), skilled birth attendance (n=33, 18.5%) and child immunisation (n=25, 14.0%).

### Overview of evidence of impact for different drivers of inequality

**Figure 2:**
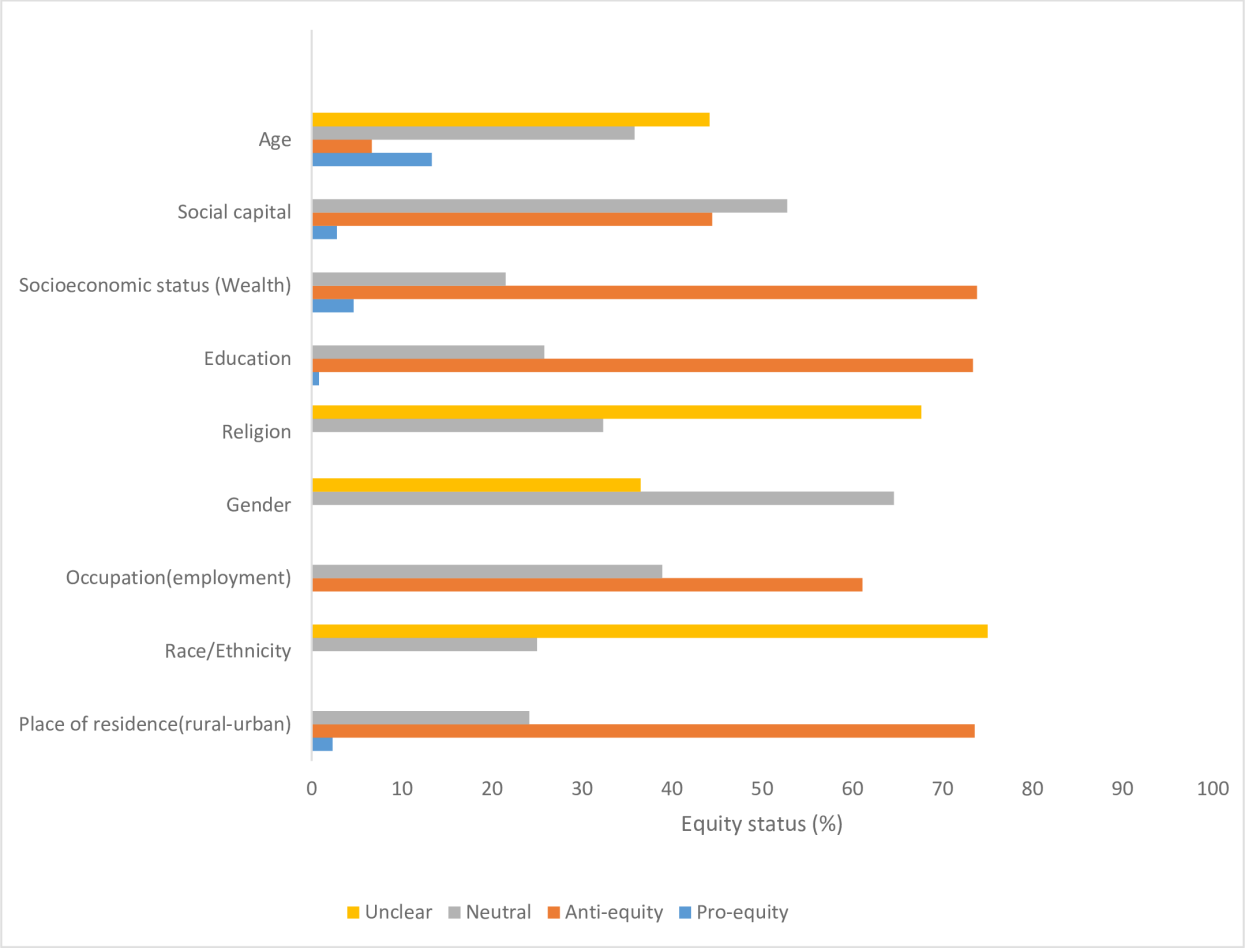
Overview of service utilisation by equity stratifiers.

We characterised quantitative findings for different social groups receiving or using services as ‘proequity’ (higher service utilisation by worst-off groups), antiequity (higher service utilisation by better-off groups) or neutral (no significant differences in groups receiving or using services). Some studies examined equity in more than one service indicator; therefore, in figure 2, we provide an overview of the reported findings for service utilisation by equity stratifiers. In general, the quantitative findings show that better-off groups across the different equity stratifiers are more likely to receive or use services.

### Synthesis of quantitative and qualitative studies by equity stratifier

#### Socioeconomic status

SES refers to social and economic factors that influence an individual’s societal class; this includes income, wealth, education and occupation. This section focuses on studies that reported directly on SES (composite measure) or used household financial or asset-based indicators for SES (wealth quintile or tertile or income).^7^ Among quantitative studies, there were 120 studies, which assessed SES across 149 service coverage indicators. In 72% (n=107) of services that were assessed by SES, those in the wealthier groups are more likely to use services with 23% of services (n=35) reporting there are no significant differences in coverage by SES and about 5% of services (n=7) that reach the poor. Nearly 60% (n=25) of services in which studies found no significant differences by SES or either reached the worst-off groups were free access to use insecticide treatment net (ITN), immunisation or antiretroviral therapy (ART).

17 quantitative studies also examined the trends of inequalities for utilisation of 28 RMNCH service indicators. Studies found that wealth inequalities in service utilisation (n=16, 57%) have decreased and this was due to the removal of financial barriers through national health insurance programmes, exemptions or waivers of user fees for RMNCH services.^16 30 37 67 127^ However, for some services and context, wealth inequalities were reported to have increased (n=10, 42%) or remained the same (n=2, 8%) over time despite national strategies to decrease inequalities.^26 37 39 40^

Evidence on the reasons for the disparity among SES groups was limited. One study^54^ which conducted programmatic analysis on mass drug administration for neglected tropical diseases (NTDs) found that although drugs were free, communities that fear side effects could lead to unaffordable health costs shaped the acceptability of coverage.

#### Place of residence

There were 105 studies that examined service coverage according to place of residence focused on three main dimensions—rural/urban differences, regional differences (n=34) and distance to health facilities. Studies that examined service coverage according to place of residence focused on three main dimensions; rural–urban differences, subnational regional differences and distance to health facilities. Evidence from quantitative studies (n=73) examining inequalities for 80 service indicators suggests that those living in urban communities were more likely to receive or use services. Nearly 74% (n=59) of service use was found to be higher among those in urban communities with only 2% of services (n=2) being used more among individuals in rural communities. However, for some services, such as ITN use (n=19, 24%), studies found that there were no significant differences in use among rural and urban groups. Studies that assessed inequalities by region, reported significant differences (n=28, 100%) in service coverage across different regions in countries. In addition, studies also found that service utilisation (n=5, 100%) was higher among those living closer to health facilities.

Quantitative findings from seven studies showed that the trend of rural–urban gap is inconsistent for eight services. Two studies^16 39^ reported that the rural–urban gap has declined over time for utilisation of four services while other studies reported^37 40 67^ this gap has either increased for three services or remained the same for one service.

Qualitative studies on place of residence were limited. One study^107^ found that those in periurban areas were mostly factory workers who finished work late when health facilities were closed and this resulted in their children missing vaccinations and pregnant women not receiving necessary antenatal services.

#### Education

A number of quantitative studies assessed inequalities in 118 service indicators according to individuals’ educational attainment. Studies reported that about 73% of the services, the educated groups were more likely to receive or use these services compared with the non-educated groups while 23% of services had equal use by both groups. More than half of the services in which there were no differences in use were for ITN and ART. Only one study reported a higher service use by individuals with lower education.^143^

12 studies^26 30 39 40 67 80 80 89 106 127 178^ reported the inequalities trend by education level for 21 services. Among these 19 services, studies reported that inequalities decreased over time in 10, while inequalities increased for another 8. In three services, studies found no change in equalities by education.

Qualitative studies on education were also limited. One study^117^ on the use of skilled-birth attendance in Ethiopia observed that giving birth at home was higher among uneducated women due to the need to abide by the decision of their mothers-in-laws who believed in giving birth at home compared with educated women who have the agency to make their own decision.

#### Gender

57^7^ quantitative studies assessed inequalities using gender differences (men or women) of service users or their heads of households for utilisation in 63 service indicators. There were no differences in service use by men and women for 43 of them (n=43), of which nearly half of the services were ITN use. In 24% of services, females or households headed by females were more likely to receive or use services. Only about 8% of services did studies report higher use by males or households headed by males.

Four of the quantitative studies^97 129 185 188^ examined the trend of inequalities by gender for seven service indicators. Studies reported that service use among women and men remained the same over time except for two services.

The two qualitative findings^17 54^ show that the differences in service use among men and women vary for each gender. In South-South Nigeria, one study reported that although water, sanitation and hygiene (WASH) services are poor for slum dwellers, women in slums bear the disproportionate burden of inadequate services due to the gendered distribution of childcare, household work and the need to fulfil the personal sanitary needs.^17^ However, another study about NTDs found that men were left out of the distribution of treatment drugs due to their livelihood activities such as farming and fishing being concentrated outside of communities.^54^

#### Language/race/ethnicity

Seven quantitative studies^16 31 52 71 118 151 177^ reported inequalities by either language, race or ethnicity for eight service indicators. In six of these services, studies reported that those in majority groups were more likely to receive or use services while in two services, there were equal use among groups.

No qualitative study reported inequalities by language, race or ethnicity.

#### Religion

There were 29 quantitative studies, which examined inequalities in the use of 34 services by religion. Among these 34 services, studies found that in 23, there were significant differences among religious groups in receiving or using services. In 20 of them, studies reported that Christians or one of its types were more likely to use services compared with non-Christians. However, it was not clear whether Christians were the religious majority group in study settings.

No qualitative study reported inequalities by religion.

#### Occupation

48 quantitative studies examined inequalities for coverage in 60 services by occupation. Among these studies, 28 of them examined inequalities for utilisation for 36 service indicators by occupation through employment status (employed vs non-employed) of individuals, their spouse or parents. For these 36 service indicators, studies reported that for about 61% (n=22,), those employed were more likely to receive or use services compared with non-employed, while for the remaining (n=14, 39%), there were no significant differences between these two groups. The studies (n=20) that assessed occupation using occupational groups, examined inequalities for utilisation of 23 service indicators. They reported that for the majority (n=16, 70%) of these services, there were differences in utilisation among the different occupational groups, while for the remaining service indicators, there were no differences among them. Studies were not explicit about the occupational groups that were better off or the worst-off in their contexts, and so making it difficult to determine equitable use among the groups. Only two studies^39 129^ examined the trend of inequalities by occupation over time, and they reported that inequalities by occupation have increased.

#### Social capital

35 quantitative studies examined inequalities by social capital for 41 service indicators. Among these studies, 31 assessed social capital using marital status for 36 service indicators, and they reported that for half of services (n=19), there were no significant differences in use among those married compared with other groups such as single, widowed or divorced. About 44% of services (n=16), those married were more likely to receive or use services. Only one study^144^ reported one service (ITN use) whereby those not married were least likely to use ITNs.

Two studies^28 178^ examined inequalities over time by marital status for one service indicator each. Both found that inequalities in previous periods whereby previously there were no significant differences among groups, this has changed to service coverage favouring those that are married. The qualitative findings from two studies^119 128^ may explain the reason marital status did not contribute to inequalities in service coverage. The findings show that for married women, lack of husbands’ consent inhibited taking contraceptives.^119^ In addition, those married women could be ‘thrown out of their marital laws by their in-laws just because they used contraceptives’. The other study^128^ observed that for pregnant married women in need of antenatal care services, minimal or lack of support from husbands in the form of financial assistance or domestic chores hindered women from receiving these services. Three quantitative studies^114 139 143^ examined social capital for one service each and they all reported that those with stronger social support or social network were more likely to use or receive services.

Only qualitative studies explored inequalities by social support.^117^ In Ethiopia, the study reported that women with strong social support such as close relatives, neighbours and friends were more vulnerable to social persuasion to deliver at home than skilled birth care in health facility.

#### Age

The 111 quantitative studies that examined inequalities by age assessed it by the age of users or their parents for 121 service indicators. Studies reported that for nearly 36% (n=43) of services, there was an equal use of services by different age groups while about for 35% (n=42) of services, older age groups were more likely to receive or use services and for the remaining of services (n=36, 30%), younger age groups were more likely to use. No clear distinction between services (NCDs, CDs or RMNCH) that each age group were more likely to use or receive.

Six studies^30 89 127 129 159 167^ assessed the trend of inequalities by age groups over time for eight service indicators. The findings show that the trend of inequalities by age group is unclear. Among the eight service indicators, inequalities remained the same, while for three, it increased and for one service, it decreased.

One qualitative study^117^ reported experiences of different age groups in the use of antenatal care, infant immunisation and SBA. The study reported that services were accessed equally across the different age groups except for SBA where younger women (18–25 years) were more likely to deliver with SBA. However, they did not elaborate on the factors that contributed to the differences in SBA use among the different age groups.

#### Disability

Only one quantitative study examined inequalities by disability alone and they reported that disabled individuals were less likely to be on ART compared with non-disabled persons.^66^

#### Intersectionality

Some studies (n=3) also assessed inequalities through an intersectionality lens. Intersectionality is a theoretical framework in which human experiences are shaped by a web of intersecting equity stratifiers (eg, SES and gender). Two quantitative studies^73 89^ from Kenya showed the intersection of geographical location and other equity stratifiers on service coverage. One study^93^ showed that generally, regardless of urban or rural residence, service use was still very low for the poorest populations compared with the better-off populations while the other^73^ reported that in each type of geographic residence, there are different individual factors that affect service use. One study^174^ examined the intersection of disability type and multiple equity stratifiers and found that the more educated women with hearing difficulties were more likely to use antenatal care compared with non-disabled women without an education while wealthier women with hearing difficulties had fewer chances of receiving the same services. Qualitative findings revealed how service utilisation by men and women is shaped by their other social positions. Two studies found that gendered roles intersected with occupation to decrease service utilisation; migrant women working as head porters with long working schedules faced many challenges in accessing health facilities for contraceptives uptake.^119^ On the other hand, in rural areas, men who worked farther away from communities are less likely to receive treatment for NTDs during drug mass drug administration campaigns.^54^ Studies also revealed the intersection of gender and SES, age, marital status and social support from husband or in-laws.^119 128^

## Discussion

Ensuring fair access to essential services is crucial to the movement towards UHC and other health goals. We provide a comprehensive and current consolidation of evidence on the state of health inequalities in the African region. We see over half of the evidence coming from only five countries—Ethiopia, Ghana, Kenya, Nigeria and Uganda—and most evidence is focused on inequalities due to SES, age and education while the least evidence looks at inequalities due to disability, social capital and ethnicity/race.

A striking finding of this review is the persistent inequalities in UHC service indicators across the different equity stratifiers. This finding is consistent with previous summaries on socioeconomic inequalities and service utilisation.^191^,^193^ Qualitative evidence seems to suggest that these inequalities are driven by multiple factors. These include the inability to pay for services and other indirect costs that create barriers for low-income individuals to access services. Many studies have shown that financial barriers, such as direct health costs, transportation costs and informal payments, deter service utilisation by those in the lowest wealth groups in Africa.^194^,^196^ Another factor hindering service utilisation is the limited accommodation of services, particularly for those with long working hours. Another important driver is sociocultural norms surrounding the use of services such as skilled birth deliveries and contraceptives. These driving factors can intersect to exacerbate or reduce inequalities.^174 197^

We see some services have limited disparities across different stratifiers. These are the interventions that emphasise mass distribution and free access to everyone and target vulnerable populations as persons needing special attention in the mass distributed interventions. These include those interventions for ITN and ART services. This approach to providing services would, therefore, be useful in addressing inequalities in utilisation. However, the sustainability of this approach is questionable without external financing.^198^,^200^

The review reveals that strategies to reduce financial barriers, such as health insurance programmes, exemptions and subsidies, may have reduced inequality gaps over time to an extent for some services. However, they may not be sufficient to address inequalities in universal service coverage. These financial initiatives may be effective in reducing inequalities for services covered under them or those in which providers are incentivised to increase utilisation for vulnerable populations. Their inadequacy is probably due to socioeconomic inequalities in insurance enrolment^201 202^ and governance structures around funding flows for their effective implementation.^203 204^ Other strategies that are been used to reduce other accessibility barriers are supply-side interventions such as the availability of community health workers for equitable PHC services. In Ethiopia, the review showed that its community health extension programme has contributed to narrowing equity gaps for some services, but inequities still exist. The persisting inequities are due to challenges the programme faces with the productivity and efficiency of extension workers, the capacity of health posts and social determinants of health.^205^

Our findings highlight the need to consider how context and social processes influence service utilisation. For example, the review showed that structural and financial social support from spouses were crucial factors for women using ANC in Ghana. In Ethiopia, on the other hand, not having social support from family increased the need for skilled birth deliveries. Likewise, although women face more barriers in accessing services than men,^206^ the review revealed they are more likely to use services than men. The underutilisation of services by men is linked to a low priority of men’s health in health systems and masculine social norms that perpetuate that healthcare seeking or use health services is a female task.^207^

While our study provides a comprehensive overview of consolidated evidence on health inequalities in the African region, several methodological limitations should be considered when interpreting the findings. These include our search strategy, which primarily included articles published in English due to resource constraints and the predominance of English in the databases searched. Additionally, adapting the WHO UHC service coverage indicators limited our ability to explore inequalities in the quality of services provided, as these indicators are primarily focused on utilisation.

## Conclusion

We have explored the evidence on health inequalities driving health outcomes across the African Region. We have seen that a lot of the evidence is informed by a few health service interventions, and inequality stratifiers. Even with this limitation, however, we have been able to derive some inferences about how to address health inequalities in the region. First, we see that the way different services are provided impacts their contribution to addressing inequalities. Interventions that are targeted at whole populations with vulnerability targeting as a subset of the programme lead to fewer inequalities. Targeting inequalities should be done as part of a wider set of interventions aimed at ensuring everyone who needs the service has access to it. Second, we also see different stratifiers have different effects on health outcomes depending on the context. It is important that efforts to address inequalities are contextualised, and not only driven by advocacy for specific areas. Health inequality interventions in the African region be tailored to the specific context of each country. A comprehensive understanding of the distinctive drivers of health inequalities across different settings is of critical importance for the design of effective interventions.

It is critical that further research is conducted to address the gaps in understanding health inequalities. There are, for instance, few studies exploring inequalities in NCD service access, or health emergency response—both of which are crucial to health and well-being in the region. Additionally, few studies look at multiple stratifiers, and yet many of these stratifiers are inter-related and have similar effects on health outcomes. There is a need to use diverse research methods, this include expand the study of overlooked social stratifiers and countries and investigating the impact of various health system interventions on equity. Importantly, stimulating research efforts across services for different diseases is crucial to fill these knowledge gaps to examine the progress being made towards UHC as countries make epidemiological transitions and population health needs shift

A strength of our scoping review is bringing together a wide scope of studies using a comprehensive strategy. Furthermore, by applying the PROGRESS-Plus framework, we ensured a broader dimension of social process factors to uncover inequities in service utilisation. However, there were some limitations. Adapting the WHO UHC service coverage indicators prevented us from exploring inequalities in the quality of services provided, as the coverage indicators are primarily focused on utilisation.

## supplementary material

10.1136/bmjopen-2023-082918 online supplemental file 1

10.1136/bmjopen-2023-082918 online supplemental file 2

10.1136/bmjopen-2023-082918 online supplemental file 3

10.1136/bmjopen-2023-082918 online supplemental table 1

## Data Availability

All data relevant to the study are included in the article or uploaded as online supplemental information.
