# Supplementary material for: Mapping inequalities in health service coverage in Africa: a scoping review
Source: BMJ Open. 2024 Nov 24;14(11):e082918. doi: 10.1136/bmjopen-2023-082918 (PMC11590813; doi:10.1136/bmjopen-2023-082918)
Supplement: online supplemental file 2 [file bmjopen-14-11-s002.pdf]

Appendix 2. Excluded reports with reason for exclusion

Identification	Authors	Year	Title	Reason of exclusion
D_0012	Abbott P., Sapsford R.,Rwirahira J.	2015	Rwanda's potential to achieve the millennium development goals for health	Context
D_0019	Abdel-Razik M. S., El Shafei A. M. H., Abd Al Moety A. M., Al Amir R. Y.,Hosney M. S.	2020	Capitalizing on specialists' services in rural family health unit: Interventional study	Concept
D_0028	Abiiro G. A., Mbera G. B.,De Allegri M.	2014	Gaps in universal health coverage in Malawi: a qualitative study in rural communities	Concept
D_0035	Abodey E., Vanderpuye I., Mensah I.,Badu E.	2020	In search of universal health coverage - highlighting the accessibility of health care to students with disabilities in Ghana: a qualitative study	Context
D_0046	Access G. B. D. Healthcare, Quality Collaborators. Electronic address cjlm uw edu, Access G. B. D. Healthcare,Quality Collaborators	2017	Healthcare Access and Quality Index based on mortality from causes amenable to personal health care in 195 countries and territories, 1990-2015: a novel analysis from the Global Burden of Disease Study 2015	Context
D_0047	Access G. B. D. Healthcare,Quality Collaborators	2018	Measuring performance on the Healthcare Access and Quality Index for 195 countries and territories and selected subnational locations: a systematic analysis from the Global Burden of Disease Study 2016	Context
D_0048	Accorsi S., Somigliana E., Farese P., Ademe T., Desta Y., Putoto G.,Manenti F.	2017	Gender Inequalities in Remote Settings: Analysis of 105,025 Medical Records of a Rural Hospital in Ethiopia (2005-2015)	Context
D_0049	Acey C., Kisiangani J., Ronoh P., Delaire C., Makena E., Norman G., Levine D., Khush R.,Peletz R.	2019	Cross-subsidies for improved sanitation in low income settlements:	Concept

Appendix 2. Excluded reports with reason for exclusion

			Assessing the willingness to pay of water utility customers in Kenyan cities	
D_0054	Acosta A., Obi E., Ato Selby R., Ugot I., Lynch M., Maire M., Belay K., Okechukwu A., Inyang U., Kafuko J., Greer G., Gerberg L., Fotheringham M., Koenker H.,Kilian A.	2018	Design, Implementation, and Evaluation of a School Insecticide-Treated Net Distribution Program in Cross River State, Nigeria	Concept
D_0060	Adejumo O. A.,Adejumo O. A.	2021	Recalling the universal health coverage vision and equity in the covid-19 vaccine distribution plan	Type of report
D_0087	Agadjanian V., Yao J.,Hayford S. R.	2016	Place, Time and Experience: Barriers to Universalization Of Institutional Child Delivery in Rural Mozambique	Context
D_0115	Aikins A. D., Kushitor M., Koram K., Gyamfi S.,Ogedegbe G.	2014	Chronic non-communicable diseases and the challenge of universal health coverage: insights from community-based cardiovascular disease research in urban poor communities in Accra, Ghana	Context
D_0125	Akazili J., Kanmiki E. W., Anaseba D., Govender V., Danhoundo G.,Koduah A.	2020	Challenges and facilitators to the provision of sexual, reproductive health and rights services in Ghana	Concept
D_0136	Akpabio E. M.,Takara K.	2014	Understanding and confronting cultural complexities characterizing water, sanitation and hygiene in Sub-Saharan Africa	Type of report
D_0148	Alamneh T. S., Teshale A. B., Yeshaw Y., Alem A. Z., Ayalew H. G., Liyew A. M., Tessema Z. T., Tesema G. A.,Worku M. G.	2022	Socioeconomic inequality in barriers for accessing health care among married reproductive aged women in sub-Saharan African countries: a decomposition analysis	Context
D_0151	Alebachew A., Hatt L.,Kukla M.	2014	Monitoring and Evaluating Progress towards Universal Health Coverage in Ethiopia	Type of report
D_0154	Alexander K. T., Tesfaye Y., Dreibelbis R., Abaire	2015	Governance and functionality of	Concept

Appendix 2. Excluded reports with reason for exclusion

	B.,Freeman M. C.		community water schemes in rural Ethiopia	
D_0171	Ali M., Farron M., Ramachandran Dilip T.,Folz R.	2018	Assessment of Family Planning Service Availability and Readiness in 10 African Countries	Concept
D_0173	Aliyu A. A.,Dahiru T.	2017	Predictors of delayed Antenatal Care (ANC) visits in Nigeria: secondary analysis of 2013 Nigeria Demographic and Health Survey (NDHS)	Context
D_0181	Alonso P. L., Bell D., Hanson K., Mendis K., Newman R. D., de Savigny D., Schapira A., Slutsker L., Tanner M., Teuscher T.,mal E. R. A. Consultative Grp Hlth Syst	2011	A Research Agenda for Malaria Eradication: Health Systems and Operational Research	Type of report
D_0184	Alshamsan R., Lee J. T., Rana S., Areabi H.,Millett C.	2017	Comparative health system performance in six middle-income countries: cross-sectional analysis using World Health Organization study of global ageing and health	Concept
D_0185	Alwan Ala	2013	Universal health coverage in the context of emergencies	Type of report
D_0187	Amanullah F., Bacha J. M., Fernandez L. G.,Mandalakas A. M.	2019	Quality matters: Redefining child TB care with an emphasis on quality	Type of report
D_0189	Ameh S., Akeem B. O., Ochimana C., Oluwasanu A. O., Mohamed S. F., Okello S., Muhihi A.,Danaei G.	2021	A qualitative inquiry of access to and quality of primary healthcare in seven communities in East and West Africa (SevenCEWA): perspectives of stakeholders, healthcare providers and users	Context
D_0192	Amissah J., Nakua E. K., Badu E., Amissah A. B.,Lariba L.	2020	In search of universal health coverage: the hidden cost of family planning to women in Ghana	Context
D_0193	Amoah P. A.,Adjei M.	2021	Social capital, access to healthcare, and health-related quality of life in urban Ghana	Context

Appendix 2. Excluded reports with reason for exclusion

D_0198	Amos O. A., Adebisi Y. A., Bamisaiye A., Olayemi A. H., Ilesanmi E. B., Micheal A. I., Ekpenyong A.,Lucero-Prisno D. E.	2021	COVID-19 and progress towards achieving universal health coverage in Africa: A case of Nigeria	Type of report
D_0199	Amouzou A., Habi O.,Bensaïd K.	2012	Reduction in child mortality in Niger: A Countdown to 2015 country case study	Concept
D_0216	Anarwat Samuel George	2018	Ghana's national health insurance model: Advancing financial risk protection, equity of health care access, and financial sustainability	Context
D_0222	Angèle M. N., Abel N. M., Jacques O. M., Henri M. T.,Françoise M. K.	2021	Social and economic consequences of the cost of obstetric and neonatal care in Lubumbashi, Democratic Republic of Congo: a mixed methods study	Context
D_0228	Anonymous	2018	Taking up Africa's cancer challenge	Type of report
D_0229	Anonymous	2021	Expanding universal health coverage among refugees and migrants: challenges and opportunities	Type of report
D_0237	Appleford G., RamaRao S.,Bellows B.	2020	The inclusion of sexual and reproductive health services within universal health care through intentional design	Concept
D_0254	Arroz J. A. H., Candrinho B., Mendis C., Varela P., Pinto J.,Martins M. D. O.	2018	Effectiveness of a new long-lasting insecticidal nets delivery model in two rural districts of Mozambique: a before-after study	Concept
D_0256	Arroz J. A. H., Candrinho B., Pedro S., Mathe G., Da Silva M., Tsabete S., Ismael L., Juleca I., Chande M., Bambo F., Munguande O., Julane S., Mussambala F., Alfai E., Muianga O., Matsimbe H., Varela P., Latif C., Mendis C., Lopez M., Karapetyan G.,Erskine M.	2018	Planning and implementation of a countrywide campaign to deliver over 16 million long-lasting insecticidal nets in Mozambique	Concept
D_0257	Arroz J. A. H., Mendis C., Pinto L., Candrinho B., Pinto J.,Martins M. D. R. O.	2017	Implementation strategies to increase access and demand of long-lasting insecticidal nets: A before-and-after	Concept

Appendix 2. Excluded reports with reason for exclusion

			study and scale-up process in Mozambique	
D_0258	Arsenault C., Jordan K., Lee D., Dinsa G., Manzi F., Marchant T.,Kruk M. E.	2018	Equity in antenatal care quality: an analysis of 91 national household surveys	Concept
D_0259	Asaarik Mathias J. A.,Adongo Wilfred B.	2018	Factors Influencing Unmet Need for Family Planning among Women in Fertility Age (15-49 Years Old) in West Mamprusi District in the Northern Region of Ghana	Context
D_0278	Ashton R. A., Kyabayinze D. J., Opio T., Auma A., Edwards T., Matwale G., Onapa A., Brooker S.,Kolaczinski J. H.	2011	The impact of mass drug administration and long-lasting insecticidal net distribution on Wuchereria bancrofti infection in humans and mosquitoes: An observational study in northern Uganda	Concept
D_0286	Assefa Y., Damme W. V., Mariam D. H.,Kloos H.	2010	Toward universal access to HIV counseling and testing and antiretroviral treatment in ethiopia: Looking beyond HIV testing and art initiation	Concept
D_0292	Assefa Y., Lynen L., Kloos H., Hill P., Rasschaert F., Hailemariam D., Neilsen G.,Van Damme W.	2015	Long-term outcomes and their determinants in patients on antiretroviral treatment in Ethiopia, 2005/6-2011/12: A retrospective cohort study	Context
D_0304	Ataguba J. E.,Ingabire M. G.	2016	Universal Health Coverage: Assessing Service Coverage and Financial Protection for All	Type of report
D_0308	Ataguba J. E.,McIntyre D.	2012	Paying for and receiving benefits from health services in South Africa: is the health system equitable?	Context
D_0309	Ataguba John E., Day Candy,McIntyre Di	2014	Monitoring and evaluating progress	Type of

Appendix 2. Excluded reports with reason for exclusion

			towards Universal Health Coverage in South Africa	report
D_0314	Atake E. H.	2020	Does the type of health insurance enrollment affect provider choice, utilization and health care expenditures?	Context
D_0337	Awolude O. A., Oyerinde S. O.,Akinyemi J. O.	2018	Screen and triage by community extension workers to facilitate screen and treat: Task-sharing strategy to achieve universal coverage for cervical cancer screening in Nigeria	Concept
D_0351	Ayieko J., Petersen M. L., Kabami J., Mwangwa F., Opel F., Nyabuti M., Charlebois E. D., Peng J., Koss C. A., Balzer L. B., Chamie G., Bukusi E. A., Kamya M. R.,Havlir D. V.	2021	Uptake and outcomes of a novel community-based HIV post-exposure prophylaxis (PEP) programme in rural Kenya and Uganda	Concept
D_0355	Ayyangar A., Narayanan S., Devaraj R., Kumar V. S., Devkar G.,Annamalai T. R.	2019	Target segmentation in WASH policies, programmes and projects: a systematic review	Context
D_0357	Azuogu B. N., Umeokonkwo C. D., Azuogu V. C., Onwe O. E., Okedo-Alex I. N.,Egbuji C. C.	2019	Appraisal of willingness to vaccinate daughters with human papilloma virus vaccine and cervical cancer screening uptake among mothers of adolescent students in Abakaliki, Nigeria	Context
D_0365	Baernighausen T., Bloom D. E.,Humair S.	2016	Human resources for treating HIV/AIDS: Are the preventive effects of antiretroviral treatment a game changer?	Concept
D_0371	Baker U., Peterson S., Marchant T., Mbaruku G., Temu S., Manzi F.,Hanson C.	2015	Identifying implementation bottlenecks for maternal and newborn health interventions in rural districts of the United Republic of Tanzania	Concept
D_0373	Bakibinga P., Kabaria C., Kasiira Z., Kibe P., Kyobutungi C., Mbaya N., Mberu B., Mohammed S., Njeri A., Azam I., Iqbal R., Nazish A., Rizvi N., Ahmed	2021	Inequity of healthcare access and use and catastrophic health spending in slum communities: a retrospective,	Context

Appendix 2. Excluded reports with reason for exclusion

	Saks, Choudhury N., Alam O., Khan A. Z., Rahman O., Yusuf R., Odubanjo D., Ayobola M., Fayehun O., Omigbodun A., Osuh M., Owoaje E., Taiwo O., Lilford R. J., Sartori J., Watson S. I., Diggle P. J., Aujla N., Chen Y. F., Gill P., Griffiths F., Harris B., Madan J., Muir H., Oyebode O., Pitidis V., de Albuquerque J. P., Smith S., Taylor C., Ulbrich P., Uthman O. A., Wilson R., Yeboah G.,Improving Hlth Slums Collaborative		cross-sectional survey in four countries	
D_0381	Banke-Thomas A., Abejirinde I. O. O., Ayomoh F. I., Banke-Thomas O., Eboreime E. A.,Ameh C. A.	2020	The cost of maternal health services in low-income and middle-income countries from a provider's perspective: a systematic review	Concept
D_0390	Barasa E., Nguhiu P.,McIntyre D.	2018	Measuring progress towards Sustainable Development Goal 3.8 on universal health coverage in Kenya	Concept
D_0397	Bärnighausen T., Bloom D. E.,Humair S.	2007	Human resources for treating HIV/AIDS: Needs, capacities, and gaps	Concept
D_0415	Batisso E., Habte T., Tesfaye G., Getachew D., Tekalegne A., Kilian A., Mpeka B.,Lynch C.	2012	A stitch in time: A cross-sectional survey looking at long lasting insecticide-treated bed net ownership, utilization and attrition in SNNPR, Ethiopia	Concept
D_0442	Bemelmans M., Van Den Akker T., Ford N., Philips M., Zachariah R., Harries A., Schouten E., Hermann K., Mwagomba B.,Massaquoi M.	2010	Providing universal access to antiretroviral therapy in Thyolo, Malawi through task shifting and decentralization of HIV/AIDS care	Type of report
D_0448	Benatar S.,Gill S.	2021	Universal Access to Healthcare: The Case of South Africa in the Comparative Global Context of the Late Anthropocene Era	Type of report
D_0450	Bendavid E., Leroux E., Bhattacharya J., Smith N.,Miller G.	2010	The relation of price of antiretroviral drugs and foreign assistance with coverage of HIV treatment in Africa: retrospective study	Concept

Appendix 2. Excluded reports with reason for exclusion

D_0466	Bertoncello Chiara, Cocchio Silvia, Fonzo Marco, Bennici Silvia Eugenia, Russo Francesca,Putoto Giovanni	2020	The potential of mobile health clinics in chronic disease prevention and health promotion in universal healthcare systems. An on-field experiment	Context
D_0472	Beyene M. G., Zemedu T. G., Gebregiorgis A. H., Ruano A. L.,Bailey P. E.	2021	Cesarean delivery rates, hospital readiness and quality of clinical management in Ethiopia: national results from two cross-sectional emergency obstetric and newborn care assessments	Concept
D_0475	Bhan Gautam, Surie Aditi, Horwood Christiane, Dobson Richard, Alfers Laura, Portela Anayda,Rollinse Nigel	2020	Informal work and maternal and child health: a blind spot in public health and research	Type of report
D_0480	Bhatt Ami S.,Huang Franklin W.	2021	GLOBAL ONCOLOGY: PUTTING OUR VISION OF AN EQUITABLE WORLD INTO ACTION	Type of report
D_0481	Bhatt S., Weiss D. J., Mappin B., Dalrymple U., Cameron E., Bisanzio D., Smith D. L., Moyes C. L., Tatem A. J., Lynch M., Fergus C. A., Yukich J., Bennett A., Eisele T. P., Kolaczinski J., Cibulskis R. E., Hay S. I.,Gething P. W.	2015	Coverage and system efficiencies of insecticide-treated nets in Africa from 2000 to 2017	Concept
D_0485	Biadgo A., Legesse A., Estifanos A. S., Singh K., Mulissa Z., Kiflie A., Magge H., Bitewulign B., Abate M.,Alemu H.	2021	Quality of maternal and newborn health care in Ethiopia: a cross-sectional study	Concept
D_0486	Bicaba F., Browne L., Kadio K., Bila A., Bicaba A.,Druetz T.	2020	National user fee abolition and health insurance scheme in Burkina Faso: How can they be integrated on the road to universal health coverage without increasing health inequities?	Type of report
D_0496	Binagwaho Agnes,Adhanom Ghebreyesus Tedros	2019	Primary healthcare is cornerstone of universal health coverage	Type of report
D_0502	Binyaruka P., Patouillard E., Powell-Jackson T., Greco G., Maestad O.,Borghi J.	2015	Effect of paying for performance on utilisation, quality, and user costs of	Concept

Appendix 2. Excluded reports with reason for exclusion

			health services in Tanzania: A controlled before and after study	
D_0503	Binyaruka P., Robberstad B., Torsvik G.,Borghi J.	2018	Who benefits from increased service utilisation? Examining the distributional effects of payment for performance in Tanzania	Context
D_0507	Binyaruka P.,Mori A. T.	2021	Economic consequences of caesarean section delivery: evidence from a household survey in Tanzania	Concept
D_0526	Boerma T., Requejo J., Victora C. G., Amouzou A., George A., Agyepong I., Barroso C., Barros A. J. D., Bhutta Z. A., Black R. E., Borghi J., Buse K., Aguirre L. C., Chopra M., Chou D., Chu Y., Claeson M., Daelmans B., Davis A., DeJong J., Diaz T., El Arifeen S., Ewerling F., Fox M., Gillespie S., Grove J., Guenther T., Haakenstad A., Hosseinpoor A. R., Hounton S., Huicho L., Jacobs T., Jiwani S., Keita Y., Khosla R., Kruk M. E., Kuo N. T., Kyobutungi C., Langer A., Lawn J. E., Leslie H., Liang M. J., Maliqi B., Manu A., Masanja H., Marchant T., Menon P., Moran A. C., Mujica O. J., Nambiar D., Ohiri K., Park L. A., Patton G. C., Peterson S., Piwoz E., Rasanathan K., Raj A., Ronsmans C., Saad-Haddad G., Sabin M. L., Sanders D., Sawyer S. M., Silva I. C. M., Singh N. S., Somers K., Spiegel P., Tappis H., Temmerman M., Vaz L. M. E., Ved R. R., Vidaletti L. P., Waiswa P., Wehrmeister F. C., Weiss W., You D. Z., Zaidi S.,Countdown Collaboration	2018	Countdown to 2030: tracking progress towards universal coverage for reproductive, maternal, newborn, and child health	Type of report
D_0531	Bomfim E., Mupueleque M. A., Dos Santos D. M. M., Abdirazak A., Bernardo R. A., Zakus D., Martins Pires P. H. D. N., Siemens R.,Belo C. F.	2020	Quality assessment in primary health care: Adolescent and youth friendly service, a mozambican case study	Concept
D_0536	Bonfrer I., Breebaart L.,De Poel E. V.	2016	The effects of Ghana's national health insurance scheme on maternal and infant health care utilization	Concept
D_0556	Bovet P., Chiolero A., Paccaud F.,Banatvala N.	2015	Screening for cardiovascular disease	Type of

Appendix 2. Excluded reports with reason for exclusion

			risk and subsequent management in low and middle income countries: challenges and opportunities	report
D_0560	Boyer S., Marcellin F., Ongolo-Zogo P., Abega S. C., Nantchouang R., Spire B.,Moatti J. P.	2009	Financial barriers to HIV treatment in Yaounde, Cameroon: First results of a national cross-sectional survey	Context
D_0567	Brenner S., Mazalale J., Wilhelm D., Nesbitt R. C., Lohela T. J., Chinkhumba J., Lohmann J., Muula A. S.,De Allegri M.	2018	Impact of results-based financing on effective obstetric care coverage: evidence from a quasi-experimental study in Malawi	Concept
D_0574	Bright T., Felix L., Kuper H.,Polack S.	2017	A systematic review of strategies to increase access to health services among children in low and middle income countries	Concept
D_0575	Bright T., Felix L., Kuper H.,Polack S.	2018	Systematic review of strategies to increase access to health services among children over five in low- and middle-income countries	Concept
D_0577	Bright T.,Kuper H.	2018	A systematic review of access to general healthcare services for people with disabilities in low and middle income countries	Context
D_0578	Brown C. A., Kohler R. E., John O., Motswetla G., Mmalane M., Tapela N., Grover S., Dryden-Peterson S., Lockman S.,Dryden-Peterson S. L.	2018	Multilevel Factors Affecting Time to Cancer Diagnosis and Care Quality in Botswana	Context
D_0582	Brown L. B., Havlir D. V., Ayieko J., Mwangwa F., Owaraganise A., Kwarisiima D., Jain V., Ruel T., Clark T., Chamie G., Bukusi E. A., Cohen C. R., Kamya M. R., Petersen M. L., Charlebois E. D.,Collaboration Search	2016	High levels of retention in care with streamlined care and universal test and treat in East Africa	Context
D_0588	Bukenya D., Wringe A., Moshabela M., Skovdal M., Ssekubugu R., Paparini S., Renju J., McLean E., Bonnington O., Wamoyi J.,Seeley J.	2017	Where are we now? A multicountry qualitative study to explore access to pre-Antiretroviral care services: A precursor to antiretroviral therapy initiation	Context

Appendix 2. Excluded reports with reason for exclusion

D_0589	Bukhman G., Mocumbi A. O., Atun R., Becker A. E., Bhutta Z., Binagwaho A., Clinton C., Coates M. M., Dain K., Ezzati M., Gottlieb G., Gupta I., Gupta N., Hyder A. A., Jain Y., Kruk M. E., Makani J., Marx A., Miranda J. J., Norheim O. F., Nugent R., Roy N., Stefan C., Wallis L., Mayosi B., Adjaye-Gbewonyo K., Adler A., Amegashie F., Amuyunzu-Nyamongo M. K., Arwal S. H., Bassoff N., Beste J. A., Boudreaux C., Byass P., Cadet J. R., Dagnaw W. W., Eagan A. W., Feigl A., Gathecha G., Haakenstad A., Haileamlak A. M., Johansson K. A., Kamanda M., Karmacharya B., Kasomekera N., Kintu A., Koirala B., Kwan G. F., Larco N. C., Maongezi S., Masiye J., Mayige M., McLaughlin A., Memirie S. T., Muquingue H. N., Mwangi K. J. M., Ndayisaba G. F., Noble C. A., Noormal B., Olsen M., Park P., Aguilar G. R., Sankoh O. A., Saxena A., Schwartz L. N., Schwarz D. K., Shaffer J. D., Sumner A. P., Doe Z. T., Upreti S. R., Verguet S., Watkins D.,Wroe E. B.	2020	The Lancet NCDI Poverty Commission: bridging a gap in universal health coverage for the poorest billion	Type of report
D_0602	Buzasi K.,Vu T. H.	2020	Are Recent Improvements in Healthcare Utilisation and Under-Five Mortality Inclusive in Kenya? Evidence Based on Selected Indicators from the Demographic and Health Surveys	Concept
D_0605	Byrne A., Hodge A., Jimenez-Soto E.,Morgan A.	2014	What works? Strategies to increase reproductive, maternal and child health in difficult to access mountainous locations: A systematic literature review	Concept
D_0607	Cahill N., Sonneveldt E., Stover J., Weinberger M., Williamson J., Wei C., Brown W.,Alkema L.	2018	Modern contraceptive use, unmet need, and demand satisfied among women of reproductive age who are married or in a union in the focus countries of the Family Planning 2020	Concept

Appendix 2. Excluded reports with reason for exclusion

			initiative: a systematic analysis using the Family Planning Estimation Tool	
D_0608	Cahill N., Weinberger M.,Alkema L.	2020	What increase in modern contraceptive use is needed in FP2020 countries to reach 75% demand satisfied by 2030? An assessment using the Accelerated Transition Method and Family Planning Estimation Model	Concept
D_0611	Calhoun L. M., Speizer I. S., Guilkey D.,Bukusi E.	2018	The Effect of the Removal of User Fees for Delivery at Public Health Facilities on Institutional Delivery in Urban Kenya	Context
D_0636	Cates Jr W.,Burris H.	2010	The global roadmap to universal access to family planning: from Cairo to Kampala	Type of report
D_0639	Cawley C., McRobie E., Oti S., Njamwea B., Nyaguara A., Odhiambo F., Otieno F., Njage M., Shoham T., Church K., Mee P., Todd J., Zaba B., Reniers G.,Wringe A.	2017	Identifying gaps in HIV policy and practice along the HIV care continuum: evidence from a national policy review and health facility surveys in urban and rural Kenya	Concept
D_0645	Chabrol F.	2014	Biomedicine, public health, and citizenship in the advent of antiretrovirals in Botswana	Type of report
D_0664	Chawana R.,Van Bogaert D. K.	2011	Risk management in HIV/AIDS: Ethical and economic issues associated with restricting HAART access only to adherent patients	Concept
D_0688	Chirgwin H., Cairncross S., Zehra D.,Waddington H. S.	2021	Interventions promoting uptake of water, sanitation and hygiene (WASH) technologies in low- and middle-income countries: An evidence and gap map of effectiveness studies	Concept
D_0698	Chomi E. N., Mujinja P. G. M., Enemark U., Hansen K.,Kiwara A. D.	2014	Health care seeking behaviour and utilisation in a multiple health	Context

Appendix 2. Excluded reports with reason for exclusion

			insurance system: does insurance affiliation matter?	
D_0712	Cintas C., Akinwande V., Raghavendra R., Tadesse G. A., Walcott-Bryant A., Wayua C., Makumbi F., Wanyenze R. K.,Weldemariam K.	2021	Data-Driven Sequential Uptake Pattern Discovery for Family Planning Studies	Concept
D_0723	Coalson J. E., Santos E. M., Little A. C., Anderson E. J., Stroupe N., Agawo M., Hayden M., Munga S.,Ernst K. C.	2020	Insufficient ratio of long-lasting insecticidal nets to household members limited universal usage in western kenya: A 2015 cross-sectional study	Context
D_0733	Colson K. E., Dwyer-Lindgren L., Achoki T., Fullman N., Schneider M., Mulenga P., Hangoma P., Ng M., Masiye F.,Gakidou E.	2015	Benchmarking health system performance across districts in Zambia: A systematic analysis of levels and trends in key maternal and child health interventions from 1990 to 2010	Concept
D_0748	Coulaud P. J., Protopopescu C., Ndiaye K., Baudoin M., Maradan G., Laurent C., Spire B., Vidal L., Kuaban C., Boyer S.,Grp E. VOLCam	2021	Individual and healthcare supply-related barriers to treatment initiation in HIV-positive patients enrolled in the Cameroonian antiretroviral treatment access programme	Context
D_0763	Cronk Ryan D.	2018	Using monitoring data to identify water and sanitation service delivery improvement opportunities in low- and middle-income countries	Context
D_0764	Crossland N., Hadden W. C., Vargas W. E., Valadez J. J.,Jeffery C.	2015	Sexual and reproductive health among Ugandan youth: 2003-04 to 2012	Context
D_0768	Cumming O.,Cairncross S.	2016	Can water, sanitation and hygiene help eliminate stunting? Current evidence and policy implications	Type of report
D_0772	Dadari I., Higgins-Steele A., Sharkey A., Charlet D., Shahabuddin A., Nandy R.,Jackson D.	2021	Pro-equity immunization and health systems strengthening strategies in select Gavi-supported countries	Concept
D_0779	Dagnaw F. T., Azanaw M. M., Adamu A., Ashagrie T.,	2022	Community-based health insurance,	Context

Appendix 2. Excluded reports with reason for exclusion

	Mohammed A. A., Dawid H. Y., Tiruneh M., Demissie B., Yemata G. A., Yitbarek G. Y., Abebaw Y.,Hailemeskel H. S.		healthcare service utilization and associated factors in South Gondar Zone Northwest, Ethiopia, 2021: A comparative cross-sectional study	
D_0789	Dalinjong P. A., Wang A. Y.,Homer C. S. E.	2018	Are health facilities well equipped to provide basic quality childbirth services under the free maternal health policy? Findings from rural Northern Ghana	Concept
D_0793	Dalinjong P. A., Welaga P., Akazili J., Kwarteng A., Bangha M., Oduro A., Sankoh O.,Goudge J.	2017	The association between health insurance status and utilization of health services in rural Northern Ghana: evidence from the introduction of the National Health Insurance Scheme	Context
D_0798	Danhoundo G., Wiktorowicz M. E., Premji S. S.,Nasiri K.	2018	Determinants of bed net policy implementation: A case study of Southern Benin	Concept
D_0802	Darroch J. E.,Singh S.	2013	Trends in contraceptive need and use in developing countries in 2003, 2008, and 2012: An analysis of national surveys	Concept
D_0805	Datiko D. G., Yassin M. A., Tulloch O., Asnake G., Tesema T., Jamal H., Markos P., Cuevas L. E.,Theobald S.	2015	Exploring providers' perspectives of a community based TB approach in Southern Ethiopia: implication for community based approaches	Concept
D_0811	Dawkins B., Renwick C., Ensor T., Shinkins B., Jayne D.,Meads D.	2021	What factors affect patients' ability to access healthcare? An overview of systematic reviews	Context
D_0814	Day L. T., Ruysen H., Gordeev V. S., Gore-Langton G. R., Boggs D., Cousens S., Moxon S. G., Blencowe H., Baschieri A., Rahman A. E., Tahsina T., Zaman S. B., Hossain T., Rahman Q. S. U., Ameen S., El Arifeen S., Kc A., Shrestha S. K., Kc N. P., Singh D., Jha A. K., Jha	2019	Every Newborn-BIRTH protocol: observational study validating indicators for coverage and quality of maternal and newborn health care in Bangladesh, Nepal and Tanzania	Concept

Appendix 2. Excluded reports with reason for exclusion

	B., Rana N., Basnet O., Joshi E., Paudel A., Shrestha P. R., Jha D., Bastola R. C., Ghimire J. J., Paudel R., Salim N., Shamb D., Manji K., Shabani J., Shirima K., Mkopi N., Mrisho M., Manzi F., Jaribu J., Kija E., Assenga E., Kisenge R., Pembe A., Hanson C., Mbaruku G., Masanja H., Amouzou A., Azim T., Jackson D., Kabuteni T. J., Mathai M., Monet J. P., Moran A., Ram P., Rawlins B., Sæbø J. I., Serbanescu F., Vaz L., Zaka N.,Lawn J. E.			
D_0815	De Allegri M., Louis V. R., Tiendrébeogo J., Souares A., Yé M., Tozan Y., Jahn A.,Mueller O.	2013	Moving towards universal coverage with malaria control interventions: Achievements and challenges in rural Burkina Faso	Context
D_0817	de Beyl C. Z., Koenker H., Acosta A., Onyefunafoa E. O., Adegbe E., McCartney-Melstad A., Selby R. A.,Kilian A.	2016	Multi-country comparison of delivery strategies for mass campaigns to achieve universal coverage with insecticide-treated nets: what works best?	Context
D_0824	de Montigny S., Boily M. C., Mâsse B. R., Mitchell K. M.,Dimitrov D. T.	2018	Assessing the utility of the tipping point ratio to monitor HIV treatment programmes in the era of universal access to ART	Concept
D_0840	Degge H. M., Laurenson M., Dumbili E. W.,Hayter M.	2020	Insights from birthing experiences of fistula survivors in North-central Nigeria: Interplay of structural violence	Concept
D_0852	Demissie B.,Negeri K. G.	2020	Effect of community-based health insurance on utilization of outpatient health care services in southern ethiopia: A comparative cross-sectional study	Context
D_0866	Deshpande A., Miller-Petrie M. K., Lindstedt P. A., Baumann M. M., Johnson K. B., Blacker B. F., Abbastabar H., Abd-Allah F., Abdelalim A., Abdollahpour I., Abegaz K. H., Abejie A. N., Abreu L.	2020	Mapping geographical inequalities in access to drinking water and sanitation facilities in low-income and middle-income countries, 2000-17	Concept

Appendix 2. Excluded reports with reason for exclusion

	G., Abrigo M. R. M., Abualhasan A., Accrombessi M. M. K., Adamu A. A., Adebayo O. M., Adedeji I. A., Adedoyin R. A., Adekanmbi V., Adetokunboh O. O., Adhikari T. B., Afarideh M., Agudelo-Botero M., Ahmadi M., Ahmadi K., Ahmed A. E., Ahmed M. B., Akalu T. Y., Akanda A. S., Alahdab F., Al-Aly Z., Alam N., Alam S., Alamene G. M., Alanzi T. M., Albright J., Albujeer A., Alcalde-Rabanal J. E., Alebel A., Alemu Z. A., Ali M., Alijanzadeh M., Alipour V., Aljunid S. M., Almasi A., Almasi-Hashiani A., Al-Mekhlafi H. M., Altirkawi K. A., Alvis-Guzman N., Alvis-Zakzuk N. J., Amini S., Amit A. M. L., Amul G. G. H., Andrei C. L., Anjomshoa M., Ansariadi A., Antonio C. A. T., Antony B., Antriyandarti E., Arabloo J., Aref H. M. A., Aremu O., Armoon B., Arora A., Aryal K. K., Arzani A., Asadi-Aliabadi M., Asmelash D., Atalay H. T., Athari S. S., Athari S. M., Atre S. R., Ausloos M., Awasthi S., Awoke N., Quintanilla B. P. A., Ayano G., Ayanore M. A., Aynalem Y. A., Azari S., Azman A. S., Babaee E., Badawi A., Bagherzadeh M., Bakkannavar S. M., Balakrishnan S., Banach M., Banoub J. A. M., Barac A., Barboza M. A., Bärnighausen T. W., Basu S., Bay V. D., Bayati M., Bedi N., Beheshti M., Behzadifar M., Behzadifar M., Ramirez D. F. B., Bell M. L., Bennett D. A., Benzian H., Berbada D. A., Bernstein R. S., Bhat A. G., Bhattacharyya K., Bhaumik S., Bhutta Z. A., Bijani A., Bikbov B., Sayeed M. S. B., Biswas R. K., Bohlouli S., Boufous S., Brady O. J., Briko A. N., Briko N. I., Britton G. B., Brown A., Nagaraja S. B., Butt Z. A., Cámera L. A., Campos-Nonato I. R., Rincon J. C. C., Cano J., Car J., Cárdenas R., Carvalho F., Castañeda-Orjuela C. A., Castro F., Cerin E., Chalise B., Chattu V. K., Chin K. L., Christopher D. J., Chu D. T., Cormier N. M., Costa V. M., Cromwell E. A., Dadi A. F., Dahiru T.,	

Appendix 2. Excluded reports with reason for exclusion

	Dahlawi S. M. A., Dandona R., Dandona L., Dang A. K., Daoud F., Darwesh A. M., Darwish A. H., Daryani A., Das J. K., Das Gupta R., Dash A. P., Dávila-Cervantes C. A., Weaver N. D., De La Hoz F. P., De Neve J. W., Demissie D. B., Demoz G. T., Denova-Gutiérrez E., Deribe K., Desalew A., Dharmaratne S. D., Dhillon P., Dhimal M., Dhungana G. P., Diaz D., Dipeolu I. O., Do H. T., Dolecek C., Doyle K. E., Dubljanin E., Duraes A. R., Edinur H. A., Effiong A., Eftekhari A., El Nahas N., El Sayed Zaki M., El Tantawi M., Elhabashy H. R., El-Jaafary S. I., El-Khatib Z., Elkout H., Elsharkawy A., Enany S., Endalew D. A., Eshrati B., Eskandarieh S., Etemadi A., Ezekannagha O., Faraon E. J. A., Fareed M., Faro A., Farzadfar F., Fasil A., Fazlzadeh M., Feigin V. L., Fekadu W., Fentahun N., Fereshtehnejad S. M., Fernandes E., Filip I., Fischer F., Flohr C., Foigt N. A., Folayan M. O., Foroutan M., Franklin R. C., Frostad J. J., Fukumoto T., Gad M. M., Garcia G. M., Gatotoh A. M., Gayesa R. T., Gebremedhin K. B., Geramo Y. C. D., Gesesew H. A., Gezae K. E., Ghashghaee A., Sherbaf F. G., Gill T. K., Gill P. S., Ginindza T. G., Girmay A., Gizaw Z., Goodridge A., Gopalani S. V., Goulart A. C., Goulart B. N. G., Grada A., Green M. S., Gubari M. I. M., Gugnani H. C., Guido D., Guimarães R. A., Guo Y., Gupta R., Gupta R., Ha G. H., Haagsma J. A., Hafezi-Nejad N., Haile D. H., Haile M. T., Hall B. J., Hamidi S., Handiso D. W., Haririan H., Hariyani N., Hasaballah A. I., Hasan M. M., Hasanzadeh A., Hassen H. Y., Hayelom D. H., Hegazy M. I., Heibati B., Heidari B., Hendrie D., Henok A., Herteliu C., Heydarpour F., de Hidru H. D., Hird T. R., Hoang C. L., Hollerich G. I., Hoogar P., Hossain N., Hosseinzadeh M., Househ M., Hu G., Humayun A., Hussain S. A., Hussen M. A. A., Ibitoye S. E., Ilesanmi O. S., Ilic M. D., Imani-Nasab M.	

Appendix 2. Excluded reports with reason for exclusion

	H., Iqbal U., Irvani S. S. N., Islam S. M. S., Ivers R. Q., Iwu C. J., Jahanmehr N., Jakovljevic M., Jalali A., Jayatilleke A. U., Jenabi E., Jha R. P., Jha V., Ji J. S., Jonas J. B., Jozwiak J. J., Kabir A., Kabir Z., Kanchan T., Karch A., Karki S., Kasaeian A., Kasahun G. G., Kasaye H. K., Kassa G. M., Kassa G. G., Kayode G. A., Kebede M. M., Keiyoro P. N., Ketema D. B., Khader Y. S., Khafaie M. A., Khalid N., Khalilov R., Khan E. A., Khan J., Khan M. N. N., Khatab K., Khater M. M., Khater A. M., Khayamzadeh M., Khazaei M., Khosravi M. H., Khubchandani J., Kiadaliri A., Kim Y. J., Kimokoti R. W., Kisa S., Kisa A., Kochhar S., Kolola T., Komaki H., Kosen S., Koul P. A., Koyanagi A., Krishan K., Defo B. K., Kugbey N., Kumar P., Anil Kumar G., Kumar M., Kusuma D., La Vecchia C., Lacey B., Lal A., Lal D. K., Lam H., Lami F. H., Lansingh V. C., Lasrado S., Lebedev G., Lee P. H., LeGrand K. E., Leili M., Lenjebo T. L., Leshargie C. T., Levine A. J., Lewycka S., Li S., Linn S., Liu S., Lopez J. C. F., Lopukhov P. D., El Razek M. M. A., Mahadeshwara Prasad D. R., Mahasha P. W., Mahotra N. B., Majeed A., Malekzadeh R., Malta D. C., Mamun A. A., Manafi N., Mansournia M. A., Mapoma C. C., Martinez G., Martini S., Martins-Melo F. R., Mathur M. R., Mayala B. K., Mazidi M., McAlinden C., Meharie B. G., Mehndiratta M. M., Nasab E. M., Mehta K. M., Mekonnen T., Mekonnen T. C., Meles G. G., Meles H. G., Memiah P. T. N., Memish Z. A., Mendoza W., Menezes R. G., Mereta S. T., Meretoja T. J., Mestrovic T., Metekiya W. M., Miazgowski B., Miller T. R., Mini G. K., Mirrakhimov E. M., Moazen B., Mohajer B., Mohammad Y., Mohammad D. K., Mezerji N. M. G., Mohammadibakhsh R., Mohammed S., Mohammed J. A., Mohammed H., Mohebi F., Mokdad A. H.,	

Appendix 2. Excluded reports with reason for exclusion

	Moodley Y., Moradi G., Moradi M., Moradi-Joo M., Moraga P., Morales L., Mosapour A., Mosser J. F., Mouodi S., Mousavi S. M., Mozaffor M., Munro S. B., Muriithi M. K., Murray C. J. L., Musa K. I., Mustafa G., Muthupandian S., Naderi M., Nagarajan A. J., Naghavi M., Naik G., Nangia V., Nascimento B. R., Nazari J., Ndwandwe D. E., Negoi I., Netsere H. B., Ngunjiri J. W., Nguyen C. T., Nguyen H. L. T., Nguyen Q. P., Nigatu S. G., Ningrum D. N. A., Nnaji C. A., Nojomi M., Norheim O. F., Noubiap J. J., Oancea B., Ogbo F. A., Oh I. H., Olagunju A. T., Olusanya B. O., Olusanya J. O., Onwujekwe O. E., Ortega-Altamirano D. V., Osarenotor O., Osei F. B., Owolabi M. O., Mahesh P. A., Padubidri J. R., Pakhale S., Pana A., Park E. K., Patel S. K., Pathak A., Patle A., Paulos K., Pepito V. C. F., Perico N., Pervaiz A., Pescarini J. M., Pesudovs K., Pham H. Q., Pigott D. M., Pilgrim T., Pirsaheb M., Poljak M., Pollock I., Postma M. J., Pourmalek F., Pourshams A., Prada S. I., Preotescu L., Quintana H., Rabiee N., Rabiee M., Radfar A., Rafiei A., Rahim F., Rahimi S., Rahimi-Movaghar V., Rahman M. H. U., Rahman M. A., Rajati F., Ranabhat C. L., Rao P. C., Rasella D., Rath G. K., Rawaf S., Rawal L., Rawasia W. F., Remuzzi G., Renjith V., Renzaho A. M. N., Resnikoff S., Riahi S. M., Ribeiro A. I., Rickard J., Roever L., Ronfani L., Rubagotti E., Rubino S., Saad A. M., Sabour S., Sadeghi E., Moghaddam S. S., Safari Y., Sagar R., Sahraian M. A., Mohammad Sajadi S., Salahshoor M. R., Salam N., Saleem A., Salem M. R., Salem H., Salimi Y., Salimzadeh H., Samy A. M., Sanabria J., Santos I. S., Santric-Milicevic M. M., Jose B. P. S., Saraswathy S. Y. I., Sarrafzadegan N., Sartorius B., Sathian B., Sathish T., Satpathy M., Sawhney M., Sayyah M., Sbarra A. N., Schaeffer L. E.,	

Appendix 2. Excluded reports with reason for exclusion

	Schwebel D. C., Senbeta A. M., Senthilkumaran S., Sepanlou S. G., Serván-Mori E., Shafieesabet A., Shaheen A. A., Shahid I., Shaikh M. A., Shalash A. S., Shams-Beyranvand M., Shamsi M., Shamsizadeh M., Shannawaz M., Sharafi K., Sharma R., Sheikh A., Shetty B. S. K., Shiferaw W. S., Shigematsu M., Shin J. I., Shiri R., Shirkoohi R., Shivakumar K. M., Si S., Siabani S., Siddiqi T. J., Silva D. A. S., Singh B. B., Singh A., Singh V., Singh N. P., Singh J. A., Sinha D. N., Sisay M. M., Skiadaresi E., Smith D. L., Filho A. M. S., Sobhiyeh M. R., Sokhan A., Soriano J. B., Sorrie M. B., Soyiri I. N., Spurlock E. E., Sreeramareddy C. T., Sudaryanto A., Sufiyan M. B., Suleria H. A. R., Sykes B. L., Tabarés-Seisdedos R., Tabuchi T., Tadesse D. B., Tarigan I. U., Taye B., Tefera Y. M., Tehrani-Banihashemi A., Tekelemedhin S. W., Tekle M. G., Temsah M. H., Tesfay F. H., Tesfay B. E., Tessema Z. T., Thankappan K. R., Thekkepurakkal A. S., Thomas N., Thompson R. L., Thomson A. J., Topor-Madry R., Tovani-Palone M. R., Traini E., Tran B. X., Tran K. B., Ullah I., Unnikrishnan B., Usman M. S., Uthman O. A., Uzochukwu B. S. C., Valdez P. R., Varughese S., Veisani Y., Violante F. S., Vollmer S., Whawariat F. G., Waheed Y., Wallin M. T., Wang Y. P., Wang Y., Wangdi K., Weiss D. J., Weldesamuel G. T., Werkneh A. A., Westerman R., Wiangkham T., Wiens K. E., Wijeratne T., Wiysonge C. S., Wolde H. F., Wondafrash D. Z., Wonde T. E., Worku G. T., Yadollahpour A., Jabbari S. H. Y., Yamada T., Yaseri M., Yatsuya H., Yeshaneh A., Yilma M. T., Yip P., Yisma E., Yonemoto N., Younis M. Z., Yousof H. A. S. A., Yu C., Yusefzadeh H., Zadey S., Moghadam T. Z., Zaidi Z., Zaman S. B., Zamani M., Zandian H., Zar H. J., Zerfu T. A., Zhang Y., Ziapour A., Zodpey S., Zuniga Y.	

Appendix 2. Excluded reports with reason for exclusion

	M. H., Hay S. I.,Reiner R. C.			
D_0882	Dickson K. S., Darteh E. K. M.,Kumi-Kyereme A.	2017	Providers of antenatal care services in Ghana: evidence from Ghana demographic and health surveys 1988-2014	Context
D_0896	Diouf M., Faye B. T., Diouf E. H., Dia A. K., Konate A., Fall F. B., Sene D., Diouf M. B., Gadiaga L., Konate L., Dione D. A., Tine R. C.,Faye O.	2022	Survival of eight LLIN brands 6, 12, 24 and 36 months after a mass distribution campaign in rural and urban settings in Senegal	Context
D_0908	Doctor H. V., Radovich E.,Benova L.	2019	Time trends in facility-based and private-sector childbirth care: analysis of Demographic and Health Surveys from 25 sub-Saharan African countries from 2000 to 2016	Context
D_0913	Doherty Jane,McInytre Di	2015	#FeesMustFall and the campaign for universal health coverage	Type of report
D_0920	Dossou J. P., Cresswell J. A., Makoutode P., De Brouwere V., Witter S., Filippi V., Kanhonou L. G., Goufodji S. B., Lange I. L., Lawin L., Affo F.,Marchal B.	2018	Rowing against the current': the policy process and effects of removing user fees for caesarean sections in Benin	Concept
D_0930	Ducray J. F., Kell C. M., Basdav J.,Haffejee F.	2021	Cervical cancer knowledge and screening uptake by marginalized population of women in inner-city Durban, South Africa: Insights into the need for increased health literacy	Context
D_0931	Duda S. N., Farr A. M., Lindegren M. L., Blevins M., Wester C. W., Wools-Kaloustian K., Ekouevi D. K., Egger M., Hemingway-Foday J., Cooper D. A., Moore R. D., McGowan C. C., Nash D., Saphonn V., Saramony S., Han N., Lee M. P., Zhang F., Bele V., Pujari S., Merati T., Ramadian O., Yuliana F., Yunihastuti E., Oka S., Takano M., Kajindran A., Kamarulzaman A., Low L. L., Sim B. L. H., Capistrano R., Ditangco R., Kuo L. H., Wong W. W., Chaiwarith R., Khongpattanayothin M., Kiertiburanakul S.,	2014	Characteristics and comprehensiveness of adult HIV care and treatment programmes in Asia-Pacific, sub-Saharan Africa and the Americas: Results of a site assessment conducted by the International epidemiologic Databases to Evaluate AIDS (IeDEA) Collaboration	Context

Appendix 2. Excluded reports with reason for exclusion

	Kotarathititum W., Phanuphak P., Piyavong B., Fou E., Ng O. T., Choi J. Y., Han S. H., Carr A., Chuah J., Dickson B., Hoy J., Ji J., Norris R., Templeton D., Watson K., Bendall C., Jiamsakul A., Law M. G., McManus H., Durier N., Petersen B., Sohn A. H., Rodriguez B., Gill M. J., Saag M. S., Mugavero M. J., Willig J., Eron J. J., Napravnik S., Kitahata M. M., Crane H. M., Drozd D. R., Sterling T. R., Haas D., Bebawy S., Turner M., Gange S. J., Althoff K. N., McKaig R. G., Justice A. C., Freeman A. M., Lent C., Van Rompaey S. E., Morton L., McReynolds J., Lober W. B., Abraham A. G., Lau B., Zhang J., Jing Y., Golub E., Modur S., Wong C., Mendes A., Masys D. R., Minor B., Wehbe F., Cahn P., Krolewiecki A., Cesar C., Schechter M., Faulhaber J. C., Wolff M., Cortes C., Pape J. W., Marcelin A., Padgett D., Sierra-Madero J., Vega Y. C., Gotuzzo E., Akam W., Urayeneza E., Uwingabiye R., Modeste K., Patou W., Coucou E., Kassamina K., Mpukela M. A., Mafutaming K., Munyungu N., Kabwe J., Mbaya M., Lufindusu S., Balimba A., Susan B., Molu B. M., Blessing B., Niyongabo T., Nindagiye E., Dusengamungu C., Manariyo M., Ayaya S., Diero L., Yiannoutsos C., Musick B. S., Lyamuya R., Mbaula M. F., Mtiro H., Wilfest W., Mhinga, Maruchu I. A., Ssali J., Ssemakadde M., Deo W., Mubiru M., Masaba J. P. M., Musaazi J., Bwana M., Musinguzi N., Mbidde M., Kalanzi H., Kambugu A. D., Orama R., Mboya E., Braitstein P., Sang E., Chebet J., Chelobei F., Josphat M., Ariya P., Namaemba J., Munyisi C., Ototo R., Caroline K., Busaka O., Bett L. M., Simatwa L., Hosea S., Nancy W., Boit L., Biyegon J., Peter C., Paron M., Nandi S., Wycliffe K. N., Otieno J., Omolo K., Mukhwana W., Koech J., Ndiege D. O., Owiti J.,	

Appendix 2. Excluded reports with reason for exclusion

	Wasing'a E., Owengah E., Juma E. N., Kulzer J., Odhiambo B., Tanser F., Hoffmann C., Chi B., Naniche D., Wood R., Stinson K., Fatti G., Phiri S., Giddy J., Chimbetete C., Malisita K., Eley B., Hobbins M., Kamenova K., Faturiyele O., Fox M., Prozesky H., Technau K., Sawry S., Bohlius J., Blaser N., Estill J., Keiser O., Wandeler G., Salazar-Vizcaya L., Haas A., Ballif M., Rohner E., Wyss N., Baranczuk Z., Goodwin K., Ostinelli C. H. D., Davies M. A., Boulle A., Campbell L., Cornell M., Johnson L., Maxwell N., Myer L., Schomaker M., Porter M., Dabis F., Bissagnene E., Arrivé E., Coffie P., Jaquet A., Leroy V., Lewden C., De Rekeneire N., Sasco A. J., Zannou D. M., Ahouada C., Akakpo J., Ahomadegbé C., Bashi J., Gougounon-Houéto A., Azon-Kouanou A., Houngbé F., Sehonou J., Drabo J., Bognounou R., Dienderé A., Traore E., Zoungrana L., Zerbo B., Sawadogo A. B., Zoungrana J., Héma A., Soré I., Bado G., Tapsoba A., Messou E., Gnokoro J. C., Koné M., Kouakou G. M., Bosse C. A., Brou K., Assi A. I., Chenal H., Hawerlander D., Soppi F., Minga A., Abo Y., Yoboue J. M., Eholié S. P., Amego M. D. N., Andavi V., Diallo Z., Ello F., Tanon A. K., Koule S. O., Anzan K. C., Guehi C., Wejse C., Da Silva Z. J., Paulo J., Rodrigues A., Da Silva D., Medina C., Oliviera-Souto I., Østergaard L., Laursen A., Sodemann M., Aaby P., Fomsgaard A., Erikstrup C., Eugen-Olsen J., Leuenberger D., Hebelamou J., Maïga M. Y., Diakité F. F., Kalle A., Katile D., Traore H. A., Minta D., Cissé T., Dembelé M., Doumbia M., Fomba M., Kaya A. S., Traoré A. M., Traoré H., Toure A. A., Charurat M., Kwaghe V., Ajayi S., Alim G., Dapiap S., Otu, Igbinoba F., Benson O., Adebamowo C., James J., Obaseki, Osakede P., Olasode J., Seydi M., Sow P. S., Diop B., Manga N. M., Tine J. M., Bassabi C. C.,	

Appendix 2. Excluded reports with reason for exclusion

	Patassi A., Kotosso A., Kariyare B. G., Gbadamassi G., Komi A., Mensah-Zukong K. E.,Pakpame P.			
D_0948	Ebener S., Stenberg K., Brun M., Monet J. P., Ray N., Sobel H. L., Roos N., Gault P., Conlon C. M., Bailey P., Moran A. C., Ouedraogo L., Kitong J. F., Ko E., Sanon D., Jega F. M., Azogu O., Ouedraogo B., Osakwe C., Chanza H. C., Steffen M., Ben Hamadi I., Tib H., Asaad A. H.,Torres T. T.	2019	Proposing standardised geographical indicators of physical access to emergency obstetric and newborn care in low-income and middle-income countries	Type of report
D_0968	Eisele T. R., Miller J. M., Moonga H. B., Hamainza B., Hutchinson P.,Keating J.	2011	Malaria infection and anemia prevalence in Zambia's Luangwa District: An area of near-universal insecticide-treated mosquito net coverage	Context
D_0972	Ekem N. N., Lawani L. O., Onoh R. C., Iyoke C. A., Ajah L. O., Onwe E. O., Onyebuchi A. K.,Okafor L. C.	2018	Utilisation of preconception care services and determinants of poor uptake among a cohort of women in Abakaliki Southeast Nigeria	Context
D_0983	Ellenga Mbolla B. F., Kouala Landa C. M., Bakekolo P. R., Makani Bassakouahou J. K., Bouithy S. N., Eyeni-Sinomono T., Bianza J. R., Ossou-Nguiet P. M., Bani A. M., Kimpamboudi A., Beaney T., Ster A. C., Poulter N. R., Xia X.,Kimbally Kaky S. G.	2020	May measurement month 2018: An analysis of blood pressure screening results from Republic of the Congo	Concept
D_1003	Endalamaw A., Gilks C. F., Ambaw F.,Assefa Y.	2022	Universality of Universal Health Coverage: a Scoping Review	Concept
D_1004	Endalamaw Aklilu, Gilks Charles F., Ambaw Fentie,Assefa Yibeltal	2022	Universality of universal health coverage: A scoping review	Concept
D_1020	Eregata G. T., Hailu A., Memirie S. T.,Norheim O. F.	2019	Measuring progress towards universal health coverage: national and subnational analysis in Ethiopia	Concept
D_1054	Falzon D., Jaramillo E., Wares F., Zignol M., Floyd K.,Raviglione M. C.	2013	Universal access to care for multidrug-resistant tuberculosis: An analysis of surveillance data	Concept
D_1056	Fares H.,Puig-Junoy J.	2021	Inequity and benefit incidence analysis in healthcare use among Syrian	Context

Appendix 2. Excluded reports with reason for exclusion

			refugees in Egypt	
D_1060	Fayorsey R. N., Saito S., Carter R. J., Gusmao E., Frederix K., Koech-Keter E., Tene G., Panya M.,Abrams E. J.	2013	Decentralization of pediatric HIV care and treatment in five sub-Saharan African countries	Concept
D_1075	Fitzpatrick C., Bangert M., Mbabazi P. S., Mikhailov A., Zouré H., Polo Rebollo M., Robalo Correia e Silva M.,Biswas G.	2018	Monitoring equity in universal health coverage with essential services for neglected tropical diseases: an analysis of data reported for five diseases in 123 countries over 9 years	Concept
D_1084	Floyd S., Shanaube K., Yang B., Schaap A., Griffith S., Phiri M., Macleod D., Sloot R., Sabapathy K., Bond V., Bock P., Ayles H., Fidler S., Hayes R.,Team Hptn PopART Study	2020	HIV testing and treatment coverage achieved after 4 years across 14 urban and peri-urban communities in Zambia and South Africa: An analysis of findings from the HPTN 071 (PopART) trial	Concept
D_1089	Folayan M. O., El Tantawi M., Virtanen J. I., Feldens C. A., Rashwan M., Kemoli A. M., Villena R., Al-Batayneh O. B., Amalia R., Gaffar B., Mohebbi S. Z., Arheiam A., Daryanavard H., Vukovic A., Schroth R. J.,Early Childhood Caries Advocacy Gr	2021	An ecological study on the association between universal health service coverage index, health expenditures, and early childhood caries	Concept
D_1098	Foster N., Vassal A., Cleary S., Cunnama L., Churchyard G.,Sinanovic E.	2015	The economic burden of TB diagnosis and treatment in South Africa	Context
D_1106	Fox M. P., Mazimba A., Seidenberg P., Crooks D., Sikateyo B.,Rosen S.	2010	Barriers to initiation of antiretroviral treatment in rural and urban areas of Zambia: A cross-sectional study of cost, stigma, and perceptions about ART	Context
D_1114	Friedman E. A., Gostin L. O., Kavanagh M. M., Periago M. R., Marmot M., Coates A., Binagwaho A., Mukherjee J., Chowdhury M., Robinson T., Veloso V. G., Wang C.,Were M.	2019	Putting health equity at heart of universal coverage-the need for national programmes of action	Type of report
D_1125	Fullman N., Barber R. M., Abajobir A. A., Abate K. H., Abbafati C., Abbas K. M., Abd-Allah F., Abdulkader R. S., Abdulle A. M., Abera S. F., Aboyans V., Abu-	2017	Measuring progress and projecting attainment on the basis of past trends of the health-related Sustainable	Concept

Appendix 2. Excluded reports with reason for exclusion

	Raddad L. J., Abu-Rmeileh N. M. E., Adedeji I. A., Adetokunboh O., Afshin A., Agrawal A., Agrawal S., Ahmad Kiadaliri A., Ahmadieh H., Ahmed M. B., Aichour M. T. E., Aichour A. N., Aichour I., Aiyar S., Akinyemi R. O., Akseer N., Al-Aly Z., Alam K., Alam N., Alasfoor D., Alene K. A., Alizadeh-Navaei R., Alkerwi A., Alla F., Allebeck P., Allen C., Al-Raddadi R., Alsharif U., Altirkawi K. A., Alvis-Guzman N., Amare A. T., Amini E., Ammar W., Ansari H., Antonio C. A. T., Anwari P., Arora M., Artaman A., Aryal K. K., Asayesh H., Asgedom S. W., Assadi R., Atey T. M., Atre S. R., Avila-Burgos L., Avokpaho E. F. G. A., Awasthi A., Azzopardi P., Bacha U., Badawi A., Balakrishnan K., Bannick M. S., Barac A., Barker-Collo S. L., Bärnighausen T., Barrero L. H., Basu S., Battle K. E., Baune B. T., Beardsley J., Bedi N., Beghi E., Béjot Y., Bell M. L., Bennett D. A., Bennett J. R., Bensenor I. M., Berhane A., Berhe D. F., Bernabé E., Betsu B. D., Beuran M., Beyene A. S., Bhala N., Bhansali A., Bhatt S., Bhutta Z. A., Bicer B. K., Bidgoli H. H., Bikbov B., Bilal A. I., Birungi C., Biryukov S., Bizuayehu H. M., Blosser C. D., Boneya D. J., Bose D., Bou-Orm I. R., Brauer M., Breitborde N. J. K., Brugha T. S., Bulto L. N. B., Butt Z. A., Cahuana-Hurtado L., Cameron E., Campuzano J. C., Carabin H., Cárdenas R., Carrero J. J., Carter A., Casey D. C., Castañeda-Orjuela C. A., Castro R. E., Catalá-López F., Cercy K., Chang H. Y., Chang J. C., Charlson F. J., Chew A., Chisumpa V. H., Chitheer A. A., Christensen H., Christopher D. J., Cirillo M., Cooper C., Criqui M. H., Cromwell E. A., Crump J. A., Dandona L., Dandona R., Dargan P. I., das Neves J., Davitoiu D. V., de Courten B., De Steur H., Defo B. K., Degenhardt L., Deiparine S., Deribe K., deVeber G. A., Ding E. L., Djalalinia S., Do H. P.,	Development Goals in 188 countries: an analysis from the Global Burden of Disease Study 2016	

Appendix 2. Excluded reports with reason for exclusion

	Dokova K., Doku D. T., Donkelaar A. V., Dorsey E. R., Driscoll T. R., Dubey M., Duncan B. B., Ebel B. E., Ebrahimi H., El-Khatib Z. Z., Enayati A., Endries A. Y., Ermakov S. P., Erskine H. E., Eshrati B., Eskandarieh S., Esteghamati A., Estep K., Faraon E. J. A., Farinha C. S. E. S., Faro A., Farzadfar F., Fazeli M. S., Feigin V. L., Feigl A. B., Fereshtehnejad S. M., Fernandes J. C., Ferrari A. J., Feyissa T. R., Filip I., Fischer F., Fitzmaurice C., Flaxman A. D., Foigt N., Foreman K. J., Frank T., Franklin R. C., Friedman J., Frostad J. J., Fürst T., Furtado J. M., Gakidou E., Garcia-Basteiro A. L., Gebrehiwot T. T., Geleijnse J. M., Geleto A., Gemechu B. L., Gething P. W., Gibney K. B., Gill P. S., Gillum R. F., Giref A. Z., Gishu M. D., Giussani G., Glenn S. D., Godwin W. W., Goldberg E. M., Gona P. N., Goodridge A., Gopalani S. V., Goryakin Y., Griswold M., Gugnani H. C., Gupta R., Gupta T., Gupta V., Hafezi-Nejad N., Hailu G. B., Hamadeh R. R., Hammami M., Hankey G. J., Harb H. L., Hareri H. A., Hassanvand M. S., Havmoeller R., Hawley C., Hay S. I., He J., Hendrie D., Henry N. J., Heredia-Pi I. B., Hoek H. W., Holmberg M., Horita N., Hosgood H. D., Hostiuc S., Hoy D. G., Hsairi M., Htet A. S., Huang J. J., Huang H., Huynh C., Iburg K. M., Ikeda C., Inoue M., Irvine C. M. S., Jacobsen K. H., Jahanmehr N., Jakovljevic M. B., Jauregui A., Javanbakht M., Jeemon P., Jha V., John D., Johnson C. O., Johnson S. C., Jonas J. B., Jürisson M., Kabir Z., Kadel R., Kahsay A., Kamal R., Karch A., Karema C. K., Kasaeian A., Kassebaum N. J., Kastor A., Katikireddi S. V., Kawakami N., Keiyoro P. N., Kelbore S. G., Kemmer L., Kengne A. P., Kesavachandran C. N., Khader Y. S., Khalil I. A., Khan E. A., Khang Y. H., Khosravi A., Khubchandani J., Kieling C., Kim J. Y., Kim Y. J., Kim D., Kimokoti R. W., Kinfu Y., Kisa A.,	

Appendix 2. Excluded reports with reason for exclusion

	Kissimova-Skarbek K. A., Kivimaki M., Kokubo Y., Kopec J. A., Kosen S., Koul P. A., Koyanagi A., Kravchenko M., Krohn K. J., Kulikoff X. R., Kumar G. A., Kumar Lal D., Kutz M. J., Kyu H. H., Lalloo R., Lansingh V. C., Larsson A., Lazarus J. V., Lee P. H., Leigh J., Leung J., Leung R., Levi M., Li Y., Liben M. L., Linn S., Liu P. Y., Liu S., Lodha R., Looker K. J., Lopez A. D., Lorkowski S., Lotufo P. A., Lozano R., Lucas T. C. D., Lunevicius R., Mackay M. T., Maddison E. R., Magdy Abd El Razek H., Magdy Abd El Razek M., Majdan M., Majdzadeh R., Majeed A., Malekzadeh R., Malhotra R., Malta D. C., Mamun A. A., Manguerra H., Mantovani L. G., Manyazewal T., Mapoma C. C., Marks G. B., Martin R. V., Martinez-Raga J., Martins-Melo F. R., Martopullo I., Mathur M. R., Mazidi M., McAlinden C., McGaughey M., McGrath J. J., McKee M., Mehata S., Mehndiratta M. M., Meier T., Meles K. G., Memish Z. A., Mendoza W., Mengesha M. M., Mengistie M. A., Mensah G. A., Mensink G. B. M., Mereta S. T., Meretoja T. J., Meretoja A., Mezgebe H. B., Micha R., Millear A., Miller T. R., Minnig S., Mirarefin M., Mirrakhimov E. M., Misganaw A., Mishra S. R., Mitchell P. B., Mohammad K. A., Mohammed K. E., Mohammed S., Mohan M. B. V., Mokdad A. H., Mollenkopf S. K., Monasta L., Montañez Hernandez J. C., Montico M., Moradi-Lakeh M., Moraga P., Morawska L., Morrison S. D., Moses M. W., Mountjoy-Venning C., Mueller U. O., Muller K., Murthy G. V. S., Musa K. I., Naghavi M., Naheed A., Naidoo K. S., Nangia V., Natarajan G., Negoi R. I., Negoi I., Nguyen C. T., Nguyen Q. L., Nguyen T. H., Nguyen G., Nguyen M., Nichols E., Ningrum D. N. A., Nomura M., Nong V. M., Norheim O. F., Noubiap J. J. N., Obermeyer C. M., Ogbo F. A.,	

Appendix 2. Excluded reports with reason for exclusion

	Oh I. H., Oladimeji O., Olagunju A. T., Olagunju T. O., Olivares P. R., Olsen H. E., Olusanya B. O., Olusanya J. O., Ong K., Oren E., Ortiz A., Owolabi M. O., Pa M., Pana A., Panda B. K., Panda-Jonas S., Papachristou C., Park E. K., Patton G. C., Paulson K., Pereira D. M., Perico D. N., Pesudovs K., Petzold M., Phillips M. R., Pigott D. M., Pillay J. D., Pinho C., Piradov M. A., Pishgar F., Poulton R. G., Pourmalek F., Qorbani M., Radfar A., Rafay A., Rahimi-Movaghar V., Rahman M. H. U., Rahman M. A., Rahman M., Rai R. K., Rajsic S., Ram U., Ranabhat C. L., Rao P. C., Rawaf S., Reidy P., Reiner R. C., Reinig N., Reitsma M. B., Remuzzi G., Renzaho A. M. N., Resnikoff S., Rezaei S., Rios Blancas M. J., Rivas J. C., Roba K. T., Rojas-Rueda D., Rokni M. B., Roshandel G., Roth G. A., Roy A., Rubagotti E., Sadat N., Safdarian M., Safi S., Safiri S., Sagar R., Salama J., Salomon J. A., Samy A. M., Sanabria J. R., Santomauro D., Santos I. S., Santos J. V., Santric Milicevic M. M., Sartorius B., Satpathy M., Sawhney M., Saxena S., Saylan M. I., Schmidt M. I., Schneider I. J. C., Schneider M. T., Schöttker B., Schutte A. E., Schwebel D. C., Schwendicke F., Seedat S., Sepanlou S. G., Servan-Mori E. E., Shackelford K. A., Shaheen A., Shahraz S., Shaikh M. A., Shamsipour M., Shamsizadeh M., Shariful Islam S. M., Sharma J., Sharma R., She J., Shi P., Shibuya K., Shields C., Shifa G. T., Shiferaw M. S., Shigematsu M., Shin M. J., Shiri R., Shirkoohi R., Shirude S., Shishani K., Shoman H., Shrime M. G., Silberberg D. H., Silva D. A. S., Silva J. P., Silveira D. G. A., Singh J. A., Singh V., Sinha D. N., Skiadaresi E., Slepak E. L., Sligar A., Smith D. L., Smith A., Smith M., Sobaih B. H. A., Sobngwi E., Soljak M., Soneji S., Sorensen R. J. D., Sposato L. A., Sreeramareddy C. T., Srinivasan V., Stanaway J. D.,	

Appendix 2. Excluded reports with reason for exclusion

	Stein D. J., Steiner C., Steinke S., Stokes M. A., Strub B., Sufiyan M. B., Sunguya B. F., Sur P. J., Swaminathan S., Sykes B. L., Sylte D. O., Szoeke C. E. I., Tabarés-Seisdedos R., Tadakamadla S. K., Tandon N., Tao T., Tarekegn Y. L., Tavakkoli M., Taveira N., Tegegne T. K., Terkawi A. S., Tessema G. A., Thakur J. S., Thankappan K. R., Thrift A. G., Tiruye T. Y., Tobe-Gai R., Topor-Madry R., Torre A., Tortajada M., Tran B. X., Troeger C., Truelsen T., Tsoi D., Tuem K. B., Tuzcu E. M., Tyrovolas S., Ukwaja K. N., Uneke C. J., Updike R., Uthman O. A., van Boven J. F. M., Varughese S., Vasankari T., Venketasubramanian N., Vidavalur R., Violante F. S., Vladimirov S. K., Vlassov V. V., Vollset S. E., Vos T., Wadilo F., Wakayo T., Wallin M. T., Wang Y. P., Weichenthal S., Weiderpass E., Weintraub R. G., Weiss D. J., Werdecker A., Westerman R., Whiteford H. A., Wijeratne T., Wiysonge C. S., Woldeyes B. G., Wolfe C. D. A., Woodbrook R., Xavier D., Xu G., Yadgir S., Yakob B., Yan L. L., Yano Y., Yaseri M., Ye P., Yimam H. H., Yip P., Yonemoto N., Yoon S. J., Yotebieng M., Younis M. Z., Zaidi Z., Zaki M. E. S., Zavala-Arciniega L., Zhang X., Zipkin B., Zodpey S., Lim S. S.,Murray C. J. L.			
D_1127	Fullman N., Yearwood J., Abay S. M., Abbafati C., Abd-Allah F., Abdela J., Abdelalim A., Abebe Z., Abebo T. A., Aboyans V., Abraha H. N., Abreu D. M. X., Abu-Raddad L. J., Adane A. A., Adedoyin R. A., Adetokunboh O., Adhikari T. B., Afarideh M., Afshin A., Agarwal G., Agius D., Agrawal A., Agrawal S., Ahmad Kiadaliri A., Aichour M. T. E., Akibu M., Akinyemi R. O., Akinyemiju T. F., Akseer N., Al Lami F. H., Alahdab F., Al-Aly Z., Alam K., Alam T., Alasfoor D., Albittar M. I., Alene K. A., Al-Eyadhy A., Ali S. D., Alijanzadeh M., Aljunid S. M., Alkerwi A., Alla F.,	2018	Measuring performance on the Healthcare Access and Quality Index for 195 countries and territories and selected subnational locations: A systematic analysis from the Global Burden of Disease Study 2016	Context

Appendix 2. Excluded reports with reason for exclusion

	Allebeck P., Allen C., Alomari M. A., Al-Raddadi R., Alsharif U., Altirkawi K. A., Alvis-Guzman N., Amare A. T., Amenu K., Ammar W., Amoako Y. A., Anber N., Andrei C. L., Androudi S., Antonio C. A. T., Araújo V. E. M., Aremu O., Ärnlöv J., Artaman A., Aryal K. K., Asayesh H., Asfaw E. T., Asgedom S. W., Asghar R. J., Ashebir M. M., Asseffa N. A., Atey T. M., Atre S. R., Atteraya M. S., Avila-Burgos L., Avokpaho E. F. G. A., Awasthi A., Ayala Quintanilla B. P., Ayalew A. A., Ayele H. T., Ayer R., Ayuk T. B., Azzopardi P., Azzopardi-Muscat N., Babalola T. K., Badali H., Badawi A., Banach M., Banerjee A., Banstola A., Barber R. M., Barboza M. A., Barker-Collo S. L., Bärnighausen T., Barquera S., Barrero L. H., Bassat Q., Basu S., Baune B. T., Bazargan-Hejazi S., Bedi N., Beghi E., Behzadifar M., Behzadifar M., Bekele B. B., Belachew A. B., Belay S. A., Belay Y. A., Bell M. L., Bello A. K., Bennett D. A., Bennett J. R., Bensenor I. M., Berhe D. F., Bernabé E., Bernstein R. S., Beuran M., Bhalla A., Bhatt P., Bhaumik S., Bhutta Z. A., Biadgo B., Bijani A., Bikbov B., Birungi C., Biryukov S., Bizuneh H., Bolliger I. W., Bolt K., Bou-Orm I. R., Bozorgmehr K., Brady O. J., Brazinova A., Breitborde N. J. K., Brenner H., Britton G., Brugha T. S., Butt Z. A., Cahuana-Hurtado L., Campos-Nonato I. R., Campuzano J. C., Car J., Car M., Cárdenas R., Carrero J. J., Carvalho F., Castañeda-Orjuela C. A., Castillo Rivas J., Catalá-López F., Cercy K., Chalek J., Chang H. Y., Chang J. C., Chattopadhyay A., Chaturvedi P., Chiang P. P. C., Chisumpa V. H., Choi J. Y. J., Christensen H., Christopher D. J., Chung S. C., Ciobanu L. G., Cirillo M., Colombara D., Conti S., Cooper C., Cornaby L., Cortesi P. A., Cortinovis M., Costa Pereira A., Cousin E., Criqui M. H., Cromwell E.	

Appendix 2. Excluded reports with reason for exclusion

	A., Crowe C. S., Crump J. A., Daba A. K., Dachew B. A., Dadi A. F., Dandona L., Dandona R., Dargan P. I., Daryani A., Daryani M., Das J., Das S. K., Das Neves J., Davis Weaver N., Davletov K., De Courten B., De Leo D., De Neve J. W., Dellavalle R. P., Demoz G., Deribe K., Des Jarlais D. C., Dey S., Dharmaratne S. D., Dhimal M., Djalalinia S., Doku D. T., Dolan K., Dorsey E. R., Dos Santos K. P. B., Doyle K. E., Driscoll T. R., Dubey M., Dubljanin E., Duncan B. B., Echko M., Edessa D., Edvardsson D., Ehrlich J. R., Eldrenkamp E., El-Khatib Z., Endres M., Endries A. Y., Eshrati B., Eskandarieh S., Esteghamati A., Fakhar M., Farag T., Faramarzi M., Faraon E. J. A., Faro A., Farzadfar F., Fatusi A., Fazeli M. S., Feigin V. L., Feigl A. B., Fentahun N., Fereshtehnejad S. M., Fernandes E., Fernandes J. C., Fijabi D. O., Filip I., Fischer F., Fitzmaurice C., Flaxman A. D., Flor L. S., Foigt N., Foreman K. J., Frostad J. J., Fürst T., Futran N. D., Gakidou E., Gallus S., Gambashidze K., Gamkrelidze A., Ganji M., Gebre A. K., Gebrehiwot T. T., Gebremedhin A. T., Gelaw Y. A., Geleijnse J. M., Geremew D., Gething P. W., Ghadimi R., Ghasemi Falavarjani K., Ghasemi-Kasman M., Gill P. S., Giref A. Z., Giroud M., Gishu M. D., Giussani G., Godwin W. W., Goli S., Gomez-Dantes H., Gona P. N., Goodridge A., Gopalani S. V., Goryakin Y., Goulart A. C., Grada A., Griswold M., Grosso G., Gugnani H. C., Guo Y., Gupta R., Gupta R., Gupta T., Gupta T., Gupta V., Haagsma J. A., Hachinski V., Hafezi-Nejad N., Hailu G. B., Hamadeh R. R., Hamidi S., Hankey G. J., Harb H. L., Harewood H. C., Harikrishnan S., Haro J. M., Hassen H. Y., Havmoeller R., Hawley C., Hay S. I., He J., Hearps S. J. C., Hegazy M. I., Heibati B., Heidari M., Hendrie D., Henry N. J., Herrera Ballesteros V. H.,	

Appendix 2. Excluded reports with reason for exclusion

	Herteliu C., Hibstu D. T., Hiluf M. K., Hoek H. W., Homaie Rad E., Horita N., Hosgood H. D., Hosseini M., Hosseini S. R., Hostiuc M., Hostiuc S., Hoy D. G., Hsairi M., Htet A. S., Hu G., Huang J. J., Iburg K. M., Idris F., Igumbor E. U., Ikeda C., Ileanu B. V., Ilesanmi O. S., Innos K., Irvani S. S. N., Irvine C. M. S., Islami F., Jacobs T. A., Jacobsen K. H., Jahanmehr N., Jain R., Jain S. K., Jakovljevic M. M., Jalu M. T., Jamal A. A., Javanbakht M., Jayatilleke A. U., Jeemon P., Jha R. P., Jha V., Józwiak J., John O., Johnson S. C., Jonas J. B., Joshua V., Jürisson M., Kabir Z., Kadel R., Kahsay A., Kalani R., Kar C., Karanikolos M., Karch A., Karema C. K., Karimi S. M., Kasaeian A., Kassa D. H., Kassa G. M., Kassa T. D., Kassebaum N. J., Katikireddi S. V., Kaul A., Kawakami N., Kazanjan K., Kebede S., Keiyoro P. N., Kemp G. R., Kengne A. P., Kereselidze M., Ketema E. B., Khader Y. S., Khafaie M. A., Khajavi A., Khalil I. A., Khan E. A., Khan G., Khan M. N., Khan M. A., Khanal M. N., Khang Y. H., Khater M. M., Khoja A. T. A., Khosravi A., Khubchandani J., Kibret G. D., Kiirithio D. N., Kim D., Kim Y. J., Kimokoti R. W., Kinfu Y., Kinra S., Kisa A., Kissoon N., Kochhar S., Kokubo Y., Kopec J. A., Kosen S., Koul P. A., Koyanagi A., Kravchenko M., Krishan K., Krohn K. J., Kuate Defo B., Kumar G. A., Kumar P., Kutz M., Kuzin I., Kyu H. H., Lad D. P., Lafranconi A., Lal D. K., Lalloo R., Lam H., Lan Q., Lang J. J., Lansingh V. C., Lansky S., Larsson A., Latifi A., Lazarus J. V., Leasher J. L., Lee P. H., Legesse Y., Leigh J., Leshargie C. T., Leta S., Leung J., Leung R., Levi M., Li Y., Liang J., Liben M. L., Lim L. L., Lim S. S., Lind M., Linn S., Listl S., Liu P. Y., Liu S., Lodha R., Lopez A. D., Lorch S. A., Lorkowski S., Lotufo P. A., Lucas T. C. D., Lunevicius R., Lurton G., Lyons R. A., Maalouf F., Macarayan E. R. K., Mackay M. T., Maddison E. R.,	

Appendix 2. Excluded reports with reason for exclusion

	Madotto F., Magdy Abd El Razek H., Magdy Abd El Razek M., Majdan M., Majdzadeh R., Majeed A., Malekzadeh R., Malhotra R., Malta D. C., Mamun A. A., Manguerra H., Manhertz T., Mansournia M. A., Mantovani L. G., Manyazewal T., Mapoma C. C., Margono C., Martinez-Raga J., Martins S. C. O., Martins-Melo F. R., Martopullo I., März W., Massenburg B. B., Mathur M. R., Maulik P. K., Mazidi M., McAlinden C., McGrath J. J., McKee M., Mehata S., Mehrotra R., Mehta K. M., Mehta V., Meier T., Mejia-Rodriguez F., Meles K. G., Melku M., Memiah P., Memish Z. A., Mendoza W., Mengiste D. A., Mengistu D. T., Menota B. G., Mensah G. A., Meretoja A., Meretoja T. J., Mezgebe H. B., Miazgowski T., Micha R., Milam R., Millear A., Miller T. R., Mini G. K., Minnig S., Mirica A., Mirrakhimov E. M., Misganaw A., Mitchell P. B., Mlashu F. W., Moazen B., Mohammad K. A., Mohammadibakhsh R., Mohammed E., Mohammed M. A., Mohammed S., Mokdad A. H., Mola G. L. D., Molokhia M., Momeniha F., Monasta L., Montañez Hernandez J. C., Moosazadeh M., Moradi-Lakeh M., Moraga P., Morawska L., Moreno Velasquez I., Mori R., Morrison S. D., Moses M., Mousavi S. M., Mueller U. O., Murhekar M., Murthy G. V. S., Murthy S., Musa J., Musa K. I., Mustafa G., Muthupandian S., Nagata C., Nagel G., Naghavi M., Naheed A., Naik G. A., Naik N., Najafi F., Naldi L., Nangia V., Nansseu J. R. N., Narayan K. M. V., Nascimento B. R., Negoi I., Negoi R. I., Newton C. R., Ngunjiri J. W., Nguyen G., Nguyen L., Nguyen T. H., Nichols E., Ningrum D. N. A., Nolte E., Nong V. M., Norheim O. F., Norrving B., Noubiap J. J. N., Nyandwi A., Obermeyer C. M., Ofori-Asenso R., Ogbo F. A., Oh I. H., Oladimeji O., Olagunju A. T.,	

Appendix 2. Excluded reports with reason for exclusion

	Olagunju T. O., Olivares P. R., De Oliveira P. P. V., Olsen H. E., Olusanya B. O., Olusanya J. O., Ong K., Opio J. N., Oren E., Ortega-Altamirano D. V., Ortiz A., Ozdemir R., Pa M., Pain A. W., Palone M. R. T., Pana A., Panda-Jonas S., Pandian J. D., Park E. K., Parsian H., Patel T., Pati S., Patil S. T., Patle A., Patton G. C., Paturi V. R., Paudel D., De Moares Pedroso M., Pedroza S. P., Pereira D. M., Perico N., Peterson H., Petzold M., Peykari N., Phillips M. R., Piel F. B., Pigott D. M., Pillay J. D., Piradov M. A., Polinder S., Pond C. D., Postma M. J., Pourmalek F., Prakash S., Prakash V., Prasad N., Prasad N. M., Purcell C., Qorbani M., Quintana H. K., Radfar A., Rafay A., Rafiei A., Rahimi K., Rahimi-Movaghar A., Rahimi-Movaghar V., Rahman M., Rahman M. A., Rahman S. U., Rai R. K., Raju S. B., Ram U., Rana S. M., Rankin Z., Rasella D., Rawaf D. L., Rawaf S., Ray S. E., Razo-García C. A., Reddy P., Reiner R. C., Reis C., Reitsma M. B., Remuzzi G., Renzaho A. M. N., Resnikoff S., Rezaei S., Rezai M. S., Ribeiro A. L., Rios Blancas M. J., Rivera J. A., Roever L., Ronfani L., Roshandel G., Rostami A., Roth G. A., Rothenbacher D., Roy A., Roy N., Ruhago G. M., Sabde Y. D., Sachdev P. S., Sadat N., Safdarian M., Safiri S., Sagar R., Sahebkar A., Sahraian M. A., Sajadi H. S., Salama J., Salamati P., De Freitas Saldanha R., Salimzadeh H., Salomon J. A., Samy A. M., Sanabria J. R., Sancheti P. K., Sanchez-Niño M. D., Santomauro D., Santos I. S., Santric Milicevic M. M., Sarker A. R., Sarrafzadegan N., Sartorius B., Satpathy M., Savic M., Sawhney M., Saxena S., Saylan M. I., Schaeffner E., Schmidhuber J., Schmidt M. I., Schneider I. J. C., Schumacher A. E., Schutte A. E., Schwebel D. C., Schwendicke F., Sekerija M., Sepanlou S. G., Servan-Mori E. E., Shafieesabet A.,	

Appendix 2. Excluded reports with reason for exclusion

	Shaikh M. A., Shakh-Nazarova M., Shams-Beyranvand M., Sharafi H., Sharif-Alhoseini M., Shariful Islam S. M., Sharma M., Sharma R., She J., Sheikh A., Shfare M. T., Shi P., Shields C., Shigematsu M., Shinohara Y., Shiri R., Shirkoohi R., Shiue I., Shrime M. G., Shukla S. R., Siabani S., Sigfusdottir I. D., Silberberg D. H., Silva D. A. S., Silva J. P., Silveira D. G. A., Singh J. A., Singh L., Singh N. P., Singh V., Sinha D. N., Sinke A. H., Sisay M., Skirbekk V., Sliwa K., Smith A., Soares Filho A. M., Sobaih B. H. A., Somai M., Soneji S., Soofi M., Sorensen R. J. D., Soriano J. B., Soyiri I. N., Sposato L. A., Sreeramareddy C. T., Srinivasan V., Stanaway J. D., Stathopoulou V., Steel N., Stein D. J., Stokes M. A., Sturua L., Sufiyan M. B., Suliankatchi R. A., Sunguya B. F., Sur P. J., Sykes B. L., Sylaja P. N., Szoeke C. E. I., Tabarés-Seisdedos R., Tadakamadla S. K., Tadesse A. H., Taffere G. R., Tandon N., Tariku A. T., Taveira N., Tehrani-Banihashemi A., Temam Shifa G., Temsah M. H., Terkawi A. S., Tesema A. G., Tesfaye D. J., Tessema B., Thakur J. S., Thomas N., Thompson M. J., Tillmann T., To Q. G., Tobe-Gai R., Tonelli M., Topor-Madry R., Topouzis F., Torre A., Tortajada M., Tran B. X., Tran K. B., Tripathi A., Tripathy S. P., Troeger C., Truelsen T., Tsoi D., Tudor Car L., Tuem K. B., Tyrovolas S., Uchendu U. S., Ukwaja K. N., Ullah I., Updike R., Uthman O. A., Uzochukwu B. S. C., Valdez P. R., Van Boven J. F. M., Varughese S., Vasankari T., Venketasubramanian N., Violante F. S., Vladimirov S. K., Vlassov V. V., Vollset S. E., Vos T., Wagnew F., Waheed Y., Wallin M. T., Walson J. L., Wang Y., Wang Y. P., Wassie M. M., Weaver M. R., Weiderpass E., Weintraub R. G., Weiss J., Weldegwergs K. G., Werdecker A., West T. E., Westerman R., White R. G., Whiteford H. A., Widecka J., Winkler A. S., Wiysonge	

Appendix 2. Excluded reports with reason for exclusion

	C. S., Wolfe C. D. A., Wondimkun Y. A., Workicho A., Wyper G. M. A., Xavier D., Xu G., Yan L. L., Yano Y., Yaseri M., Yimer N. B., Yin P., Yip P., Yirsaw B. D., Yonemoto N., Yonga G., Yoon S. J., Yotebieng M., Younis M. Z., Yu C., Zadnik V., Zaidi Z., El Sayed Zaki M., Zaman S. B., Zamani M., Zenebe Z. M., Zhou M., Zhu J., Zimsen S. R. M., Zipkin B., Zodpey S., Zuhlke L. J., Murray C. J. L.,Lozano R.			
D_1128	Furuoka F.,Hoque M. Z.	2015	Determinants of antiretroviral therapy coverage in Sub-Saharan Africa	Concept
D_1176	Geldsetzer P., Manne-Goehler J., Marcus M. E., Ebert C., Zhumadilov Z., Wesseh C. S., Tsabedze L., Supiyev A., Sturua L., Bahendeka S. K., Sibai A. M., Quesnel-Crooks S., Norov B., Mwangi K. J., Mwalim O., Wong-McClure R., Mayige M. T., Martins J. S., Lunet N., Labadarios D., Karki K. B., Kagaruki G. B., Jorgensen J. M. A., Hwalla N. C., Houinato D., Houehanou C., Msaidie M., Guwatudde D., Gurung M. S., Gathecha G., Dorobantu M., Damasceno A., Bovet P., Bicaba B. W., Aryal K. K., Andall-Brereton G., Agoudavi K., Stokes A., Davies J. I., Barnighausen T., Atun R., Vollmer S.,Jaacks L. M.	2019	The state of hypertension care in 44 low-income and middle-income countries: a cross-sectional study of nationally representative individual-level data from 1.1 million adults	Concept
D_1200	Gingrich C. D., Ricotta E., Kahwa A., Kahabuka C.,Koenker H.	2017	Demand and willingness-to-pay for bed nets in Tanzania: Results from a choice experiment	Context
D_1292	Hailemeskel H. S., Assebe T., Alemayehu T., Belay D. M., Teshome F., Baye A.,Bayih W. A.	2020	Determinants of short birth interval among ever married reproductive age women: A community based unmatched case control study at Dessie city administration, Northern Ethiopia	Concept
D_1297	Haji Y., Teshome M., Alemayehu A., Mekonnen M., Gebrieal F. W.,Tsasdik A. G.	2018	The Levels of Neonatal Care Practices at Health Facilities and Home Deliveries in Rural Sidama Zone,	Concept

Appendix 2. Excluded reports with reason for exclusion

			Southern Ethiopia	
D_1306	Hallett T. B., Gregson S., Dube S., Mapfeka E. S., Mugurungi O.,Garnett G. P.	2011	Estimating the resources required in the roll-out of universal access to antiretroviral treatment in Zimbabwe	Concept
D_1308	Hamainza B., Sikaala C. H., Moonga H. B., Chanda J., Chinula D., Mwenda M., Kamuliwo M., Bennett A., Seyoum A.,Killeen G. F.	2016	Incremental impact upon malaria transmission of supplementing pyrethroid-impregnated long-lasting insecticidal nets with indoor residual spraying using pyrethroids or the organophosphate, pirimiphos methyl	Concept
D_1315	Hamre K. E. S., Ayodo G., Hodges J. S.,John C. C.	2020	Amass insecticide-treated bed net distribution campaign reduced malaria risk on an individual but not population level in a highland epidemic-prone area of kenya	Concept
D_1327	Harris B., Goudge J., Ataguba J. E., McIntyre D., Nxumalo N., Jikwana S.,Chersich M.	2011	Inequities in access to health care in South Africa	Context
D_1339	Hasan M. M., Magalhaes R. J. S., Ahmed S., Ahmed S., Biswas T., Fatima Y., Islam M. S., Hossain M. S.,Mamun A. A.	2020	Meeting the Global Target in Reproductive, Maternal, Newborn, and Child Health Care Services in Low- and Middle-Income Countries	Context
D_1349	Hellwig F., Coll C. V. N., Ewerling F.,Barros A. J. D.	2019	Time trends in demand for family planning satisfied: analysis of 73 countries using national health surveys over a 24-year period	Concept
D_1366	Hollingworth S. A., Ankrah D., Uzochukwu B. S. C., Okeke C. C., Ruiz F.,Thacher E.	2022	Antihypertensive medicine use differs between Ghana and Nigeria	Concept
D_1407	Huchko M. J., Bukusi E. A.,Cohen C. R.	2011	Building capacity for cervical cancer screening in outpatient HIV clinics in the Nyanza province of western Kenya	Concept
D_1454	Ilinca S., Di Giorgio L., Salari P.,Chuma J.	2019	Socio-economic inequality and inequity in use of health care services in Kenya: Evidence from the fourth Kenya household health expenditure	Context

Appendix 2. Excluded reports with reason for exclusion

			and utilization survey	
D_1465	Iwuji C. C., Mayanja B. N., Weiss H. A., Atuhumuza E., Hughes P., Maher D.,Grosskurth H.	2011	Morbidity in HIV-1-infected individuals before and after the introduction of antiretroviral therapy: A longitudinal study of a population-based cohort in Uganda	Context
D_1549	Kantorova V., Wheldon M. C., Ueffing P.,Dasgupta A. N. Z.	2020	Estimating progress towards meeting women's contraceptive needs in 185 countries: A Bayesian hierarchical modelling study	Concept
D_1568	Karlsen S., Say L., Souza J. P., Hogue C. J., Calles D. L., Gulmezoglu A. M.,Raine R.	2011	The relationship between maternal education and mortality among women giving birth in health care institutions: Analysis of the cross sectional WHO Global Survey on Maternal and Perinatal Health	Context
D_1576	Kasaye H. K., Yilma M. T., Bobo F. T.,Fekadu G.	2020	Poor Universal Coverage of Immediate Essential Newborn Care at Hospitals of Wollega Zones, The Case of Western Ethiopia	Context
D_1589	Kayiwa Denis, Mugambe Richard K., Mselle Jane Sembuche, Isunju John Bosco, Ssempebwa John C., Wafula Solomon Tsebeni, Ndejjo Rawlance, Kansiime Winnie K., Nalugya Aisha, Wagaba Brenda, Zziwa Jude B., Bwire Constance, Buregyeya Esther, Radooli Martin Othieno, Kimbugwe Ceaser, Namanya Emily, Bateganya Najib Lukooya, McGriff Joanne A., Wang Yuke,Ssekamatte Tonny	2020	Assessment of water, sanitation and hygiene service availability in healthcare facilities in the greater Kampala metropolitan area, Uganda	Concept
D_1590	Kayom V. O., Kakuru A.,Kiguli S.	2015	Newborn Care Practices among Mother-Infant Dyads in Urban Uganda	Context
D_1629	Kibret G. D., Demant D.,Hayen A.	2022	Geographical accessibility of emergency neonatal care services in Ethiopia: analysis using the 2016 Ethiopian Emergency Obstetric and	Concept

Appendix 2. Excluded reports with reason for exclusion

			Neonatal Care Survey	
D_1638	Kieny Marie-Paule,Evans David B.	2013	Universal health coverage	Type of report
D_1642	Kilian A., Schnurr L. W., Matova T., Selby R. A., Lokko K., Blaufuss S., Gbanya M. Z., Allan R., Koenker H., Swaka M., Greer G., Fotheringham M., Gerberg L.,Lynch M.	2017	Evaluation of a continuous community-based ITN distribution pilot in Lainya County, South Sudan 2012-2013	Concept
D_1657	Kimario K. A., Muhanga M. I.,Kayunze K. A.	2022	Household Socio-demographic Characteristics and Progress towards Attainment of Universal Health Coverage in Kilimanjaro, Tanzania	Concept
D_1690	Koenker H., Arnold F., Ba F., Cisse M., Diouf L., Eckert E., Erskine M., Florey L., Fotheringham M., Gerberg L., Lengeler C., Lynch M., Mnzava A., Nasr S., Ndiop M., Poyer S., Renshaw M., Shargie E., Taylor C., Thwing J., Van Hulle S., Ye Y., Yukich J.,Kilian A.	2018	Assessing whether universal coverage with insecticide-treated nets has been achieved: Is the right indicator being used?	Concept
D_1708	Koss C. A., Havlir D. V., Ayieko J., Kwarisiima D., Kabami J., Chamie G., Atukunda M., Mwinike Y., Mwangwa F., Owaraganise A., Peng J., Olilo W., Snyman K., Awuonda B., Clark T. D., Black D., Nugent J., Brown L. B., Marquez C., Okochi H., Zhang K., Camlin C. S., Jain V., Gandhi M., Cohen C. R., Bukusi E. A., Charlebois E. D., Petersen M. L., Kamya M. R.,Balzer L. B.	2021	HIV incidence after pre-exposure prophylaxis initiation among women and men at elevated HIV risk: A population-based study in rural Kenya and Uganda	Context
D_1719	Kramer K., Mandike R., Nathan R., Mohamed A., Lynch M., Brown N., Mnzava A., Rimisho W.,Lengeler C.	2017	Effectiveness and equity of the Tanzania National Voucher Scheme for mosquito nets over 10 years of implementation	Concept
D_1774	Lalji S., Ngondi J. M., Thawer N. G., Tembo A., Mandike R., Mohamed A., Chacky F., Mwalimu C. D., Greer G., Kaspar N., Kramer K., Mlay B., Issa K., Lweikiza J., Mutafungwa A., Nzowa M., Willilo R. A., Nyoni W., Dadi D., Ramsan M. M., Reithinger R.,Magesa S. M.	2016	School Distribution as Keep-Up Strategy to Maintain Universal Coverage of Long-Lasting Insecticidal Nets: Implementation and Results of a Program in Southern Tanzania	Concept

Appendix 2. Excluded reports with reason for exclusion

D_1795	Lawani L. O., Iyoke C. A., Onoh R. C., Nkwo P. O., Ibrahim I. A., Ekwedigwe K. C.,Ekine A. A.	2016	Obstetric benefits of health insurance: A comparative analysis of obstetric indices and outcome of enrollees and non-enrollees in southeast Nigeria	Concept
D_1798	Lawn J. E., Kinney M. V., Belizan J. M., Mason E. M., McDougall L., Larson J., Lackritz E., Friberg I. K.,Howson C. P.	2013	Born Too Soon: Accelerating actions for prevention and care of 15 million newborns born too soon	Context
D_1814	Lee E. S., Vedanthan R., Jeemon P., Kamano J. H., Kudesia P., Rajan V., Engelgau M.,Moran A. E.	2016	Quality improvement for cardiovascular disease care in low- and middle-income countries: A systematic review	Context
D_1819	Leegwater A., Wong W.,Avila C.	2015	A concise, health service coverage index for monitoring progress towards universal health coverage	Concept
D_1830	Leroy Valeriane, Malateste Karen, Rabie Helena, Lumbiganon Pagakrong, Ayaya Samuel, Dicko Fatoumata, Davies Mary-Ann, Kariminia Azar, Wools-Kaloustian Kara, Aka Edmond, Phiri Samuel, Aurpibul Linda, Yiannoutsos Constantin, Signate-Sy Haby, Mofenson Lynne, Dabis Francois,International Ie D. E. A. Pediatric Working Group	2013	Outcomes of antiretroviral therapy in children in Asia and Africa: a comparative analysis of the IeDEA pediatric multiregional collaboration	Participants
D_1831	Leslie H. H., Malata A., Ndiaye Y.,Kruk M. E.	2017	Effective coverage of primary care services in eight high-mortality countries	Participants
D_1850	Lim S. S., Allen K., Dandona L., Forouzanfar M. H., Fullman N., Goldberg E. M., Hay S. I., Holmberg M., Kutz M. J., Larson H. J., Lopez A. D., McNellan C. R., Mokdad A. H., Mooney M. D., Naghavi M., Olsen H. E., Pigott D. M., Vos T., Wang H., Achoki T., Afshin A., Allen C., Anderson G. M., Barber R., Bienhoff K. A., Blore J., Brauer M., Carter A., Casey D., Charlson F. J., Chen A. Z., Coates M. M., Coggeshall M., Cohen A. J., Deshpande A., Erskine H. E., Ferrari A. J., Fitzmaurice C., Foreman K., Fraser M., Friedman J., Frostad J.,	2016	Measuring the health-related Sustainable Development Goals in 188 countries: a baseline analysis from the Global Burden of Disease Study 2015	Participants

Appendix 2. Excluded reports with reason for exclusion

	Godwin W., Graetz N., Griswold M., Haagsma J. A., Haakenstad A., Kassebaum N. J., Kemmer L., Kulikoff X. R., Kyu H. H., Leung J., Lind M., Liu P. Y., Masiye F., Mirarefin M., Misganaw A., Moradi-Lakeh M., Nguyen G., Pinho C., Rao P. C., Reitsma M. B., Roth G. A., Santomauro D. F., Shackelford K., Silpakit N., Sligar A., Sorensen R. J. D., Stanaway J. D., Steiner C., Sur P., Vollset S. E., Wanga V., Whiteford H. A., Zhao Y., Zhou M., Murray C. J. L., Kotsakis G. A., Mock C. N., Anderson B. O., Watkins D. A., Bhutta Z. A., Nisar M. I., Akseer N., deVeber G. A., Jeemon P., Dandona R., Kumar G. A., Gething P. W., Bisanzio D., Cooper C., Ali R., Bennett D. A., Jha V., Weiss D. J., Kinfu Y., Patel V., Langan S. M., McKee M., Murthy G. V. S., Roberts B., Stöckl H., Duan L., Jin Y., Li Y., Liu S., Wang L., Ye P., Liang X., Azzopardi P., Cowie B. C., Meretoja A., Patton G. C., Alam K., Weintraub R. G., Sawyer S. M., Szoeke C. E. I., Taylor H. R., Lozano R., Barrientos-Gutierrez T., Campos-Nonato I. R., Campuzano J. C., Heredia-Pi I. B., Montañez Hernandez J. C., Rios Blancas M. J., Servan-Mori E. E., Shamah Levy T., Salomon J. A., Binagwaho A., Bärnighausen T., Cahill L. E., Ding E. L., Farvid M. S., Wagner G. R., Thorne-Lyman A. L., James P., Fitchett J. R. A., Abajobir A. A., Knibbs L. D., Veerman J. L., Lalloo R., Gouda H. N., Guo Y., McGrath J. J., Abate K. H., Gebrehiwot T. T., Gebremedhin A. T., Abbafati C., Abbas K. M., Abd-Allah F., Abdulle A. M., Abraham B., Abubakar I., Aldridge R. W., Banerjee A., Benzian H., Tillmann T., Abu-Raddad L. J., Abu-Rmeileh N. M., Melaku Y. A., Abyu G. Y., Bayou T. A., Betsu B. D., Gebru A. A., Hailu G. B., Tekle D. Y., Yalew A. Z., Adebiyi A. O., Owolabi M. O., Akinyemi R. O., Adedeji I. A., Afanvi K. A., Micha R., Shi P., Singh G. M., Badawi A., Agarwal A.,	

Appendix 2. Excluded reports with reason for exclusion

	Agrawal A., Ahmad Kiadaliri A., Norrving B., Ahmadieh H., Yaseri M., Jahanmehr N., Ahmed K. Y., Alemu Z. A., Tegegne T. K., Akanda A. S., Akinyemiju T. F., Schwebel D. C., Singh J. A., Al-Aly Z., Driscoll T. R., Leigh J., Mekonnen A. B., Neal B., Alam U., Alasfoor D., AlBuhairan F. S., Alkhateeb M. A., Aldhahri S. F., Altirkawi K. A., Terkawi A. S., Alkerwi A., Alla F., Allebeck P., Rabiee R. H. S., Roy N., Kivipelto M., Carrero J. J., Fereshtehnejad S. M., Weiderpass E., Havmoeller R., Sindi S., Al-Raddadi R., Alvarez E., Alvis-Guzman N., Paternina Caicedo A. J., Amare A. T., Ciobanu L. G., Tessema G. A., Amberbir A., Amegah A. K., Amini H., Fürst T., Karema C. K., Ammar W., Harb H. L., Amrock S. M., Andersen H. H., Antonio C. A. T., Anwari P., Ärnlöv J., Larsson A., Artaman A., Asayesh H., Asghar R. J., Atique S., Avokpaho E. F. G. A., Awasthi A., Ayala Quintanilla B. P., Bacha U., Balakrishnan K., Barac A., Barker-Collo S. L., Mohammed S., Barrero L. H., Basu S., Del L. C., Bazargan-Hejazi S., Beardsley J., Bedi N., Beghi E., Béjot Y., Sheth K. N., Bell M. L., Bello A. K., Santos I. S., Bensenor I. M., Lotufo P. A., Berhane A., Wolfe C. D., Bernabé E., Wolfe I., Bernal O. A., Roba H. S., Beyene A. S., Mesfin Y. M., Bhala N., Bhatt S., Biadgilign S., Bikbov B., Soneji S., Bjertness E., Htet A. S., Bourne R. R. A., Brainin M., Krueger H., Gotay C. C., Kissoon N., Murthy S., Pourmalek F., Brazinova A., Majdan M., Shen J., Breitborde N. J. K., Broday D. M., Brugha T. S., Buchbinder R., Gabbe B., Thrift A. G., Butt Z. A., van Donkelaar A., Martin R. V., Carabin H., Cárdenas R., Caso V., Castañeda-Orjuela C. A., Castillo Rivas J., Catalá-López F., Cavalleri F., Cecílio P., das Neves J., Massano J., Pedro J. M., Chang H., Chang J., Che X., Chiang P. P., Chibalabala M., Chisumpa V. H.,	

Appendix 2. Excluded reports with reason for exclusion

	Mapoma C. C., Choi J. J., Chowdhury R., Christensen H., Cirillo M., Piel F. B., Rodriguez A., Cooke G. S., Majeed A., Cooper L. T., Crump J. A., Derrett S., Poulton R. G., Damtew S. A., Workie S. B., Deribe K., Tefera W., Giref A. Z., Haile D., Temam Shifa G., Dargan P. I., Davis A. C., Newton J. N., Steel N., Davletov K., de Castro E. F., De Leo D., Degenhardt L., Resnikoff S., Mitchell P. B., Des Jarlais D. C., Dey S., Dhillon P. K., Lal D. K., Zodpey S., Dharmaratne S. D., Dorsey E. R., Doyle K. E., Kemp A. H., Dubey M., Rahman M. H. U., Ram U., Singh A., Verma R. K., Yadav A. K., Duncan B. B., Kieling C., Schmidt M. I., Ebrahimi H., Esteghamati A., Farzadfar F., Hafezi-Nejad N., Kasaeian A., Parsaeian M., Pishgar F., Sheikhbahaei S., Fahimi S., Malekzadeh R., Roshandel G., Sepanlou S. G., Hassanvand M. S., Khosravi A., Rahimi-Movaghar V., Endries A. Y., Ermakov S. P., Soshnikov S., Eshrati B., Farid T. A., Khan A. R., Farinha C. S. E. S., Faro A., Feigin V. L., Felicio M. M., Fernandes J. G., Fischer F., Foigt N., Shiue I., Fowkes F. G. R., Franca E. B., Franklin R. C., Garcia-Basteiro A. L., Gebre T., Gessner B. D., Gillum R. F., Mehari A., Ginawi I. A., Giroud M., Gishu M. D., Tura A. K., Gona P., Goodridge A., Gopalani S. V., Goto A., Inoue M., Greenwell K. F., Gupta R., Gupta R., Gupta V., Gutiérrez R. A., Gyawali B., Iburg K. M., Halasa Y. A., Undurraga E. A., Hamadeh R. R., Hamidi S., Hammami M., Hankey G. J., Haro J. M., Haro J. M., Hoek H. W., Skirbekk V., Horino M., Horita N., Hosgood H. D., Hoy D. G., Hu G., Huang H., Idrisov B. T., Kwan G. F., Kawakami N., Shibuya K., Islami F., Jacobs T. A., Jacobsen K. H., Jakovljevic M. B., Jansen H. A. F., Javanbakht M., Jayatilleke A. U., Jee S. H., Jiang Y., Jibat T., Jonas J. B., Kabir Z., Kalkonde Y.,	

Appendix 2. Excluded reports with reason for exclusion

	Kamal R., Kesavachandran C. N., She J., Kan H., Kandel A., Karch A., Karimkhani C., Karunapema P., Kaul A., Kayibanda J. F., Keiyoro P. N., Matzopoulos R., Parry C. D., Kengne A. P., Wiysonge C. S., Stein D. J., Mayosi B. M., Shey M., Keren A., Khader Y. S., Khan E. A., Khan G., Khang Y. H., Won S., Khoja T. A. M., Khubchandani J., Kim C., Kim D., Kim S., Kim Y. J., Kimokoti R. W., Kokubo Y., Kolte D., Kosen S., Koul P. A., Koyanagi A., Kravchenko M., Varakin Y. Y., Kuate Defo B., Kuchenbecker R. S., Kuipers E. J., Kulkarni V. S., Lal A., Lucas R. M., Lam H., Lan Q., Laryea D. O., Latif A. A., Leasher J. L., Leinsalu M., Leung R., Levi M., Linn S., Lipshultz S. E., Wilkinson J. D., Simard E. P., Liu Y., Phillips M. R., Lloyd B. K., Lo L., Logroscino G., Lunevicius R., Magdy M., Magis-Rodriguez C., Mahdavi M., Malta D. C., Meaney P. A., Margolis D. J., Martinez-Raga J., Mason-Jones A. J., Tedla B. A., Memiah P., Memish Z. A., Mendoza W., Mensink G. B. M., Meretoja T. J., Mhimbira F. A., Miller T. R., Mills E. J., Mohammadi A., Monasta L., Montico M., Ronfani L., Monis J. D., Morawska L., Sun J., Mori R., Werdecker A., Mueller U. O., Westerman R., Murdoch M. E., Murimira B., Murray J., Musa K. I., Nachega J. B., Seedat S., Tran B. X., Nagel G., Rothenbacher D., Naidoo K. S., Oladimeji O., Sartorius B., Zegeye E. A., Naldi L., Remuzzi G., Nangia V., Nejjari C., Newton C. R., Ngalesoni F. N., Nguhiu P., Nguyen Q. L., Nkamedjie P. M., Nolte S., Osborne R. H., Nomura M., Norheim O. F., Obermeyer C. M., Ogbo F. A., Oh I., Olivares P. R., Olusanya B. O., Olusanya J. O., Opio J. N., Oren E., Ortiz A., Ota E., Mahesh P. A., Park E., Park H., Patel T., Patil S. T., Patten S. B., Tonelli M., Paudel D., Pereira D. M., Perico N., Pesudovs K., Petzold M.,	

Appendix 2. Excluded reports with reason for exclusion

	Pillay J. D., Polinder S., Qorbani M., Radfar A., Rahman M., Rahman S. U., Rai R. K., Rajsic S., Raju M., Rana S. M., Ranabhat C. L., Ranganathan K., Refaat A. H., Ribeiro A. L., Rojas-Rueda D., Roy A., Sagar R., Satpathy M., Sackey B. B., Saleh M. M., Sanabria J. R., Sarmiento-Suarez R., Savic M., Sawhney M., Schmidhuber J., Schneider I. J. C., Silva D. A. S., Schutte A. E., Shaheen A., Shaikh M. A., Sharma R., Shigematsu M., Shin M., Yoon S., Shiri R., Shishani K., Sigfusdottir I. D., Silverberg J. I., Yano Y., Singh O. P., Singh P. K., Søreide K., Soriano J. B., Sposato L. A., Sreeramareddy C. T., Stahl H., Stathopoulou V., Steckling N., Stranges S., Strong M., Sunguya B. F., Swaminathan S., Sykes B. L., Tabarés-Seisdedos R., Tabb K. M., Talongwa R. T., Tarawneh M. R., Tavakkoli M., Taye B., Tuzcu E. M., Thakur J., Thomson A. J., Thurston G. D., Tobe-Gai R., Topor-Madry R., Topouzis F., Tsala Dimbuene Z., Tyrovolas S., Ukwaja K. N., Uneke C. J., Uthman O. A., Vasankari T., Vasconcelos A. M. N., Venketasubramanian N., Violante F. S., Vlassov V. V., Volkow P., Wallin M. T., Weichenthal S., Woolf A. D., Wubshet M., Xu G., Yakob B., Yan L. L., Yip P., Yonemoto N., Younis M. Z., Yu C., Zaidi Z., Zaki M. E., Zambrana-Torrelio C., Zapata T.,Zonies D.			
D_1858	Liu K., Subramanian S. V.,Lu C. L.	2019	Assessing national and subnational inequalities in medical care utilization and financial risk protection in Rwanda	Context
D_1862	Local Burden of Disease Wa S. H. Collaborators	2020	Mapping geographical inequalities in access to drinking water and sanitation facilities in low-income and middle-income countries, 2000-17	Concept
D_1863	Lockman S., Holme M. P., Makhema J., Bachanas P., Moore J., Wirth K. E., Lebelonyane R.,Essex M.	2020	Implementation of Universal HIV Testing and Treatment to Reduce HIV	Participants

Appendix 2. Excluded reports with reason for exclusion

			Incidence in Botswana: the Ya Tsie Study	
D_1885	Lozano R., Fullman N., Mumford J. E., Knight M., Barthelemy C. M., Abbafati C., Abbastabar H., Abd-Allah F., Abdollahi M., Abedi A., Abolhassani H., Abosetugn A. E., Abreu L. G., Abrigo M. R. M., Abu Haimed A. K., Abushouk A. I., Adabi M., Adebayo O. M., Adekanmbi V., Adelson J., Adetokunboh O. O., Adham D., Advani S. M., Afshin A., Agarwal G., Agasthi P., Aghamir S. M. K., Agrawal A., Ahmad T., Akinyemi R. O., Alahdab F., Al-Aly Z., Alam K., Albertson S. B., Alemu Y. M., Alhassan R. K., Ali M., Ali S., Alipour V., Aljunid S. M., Alla F., Almadi M. A. H., Almasi A., Almasi-Hashiani A., Almasri N. A., Al-Mekhlafi H. M., Almulhim A. M., Alonso J., Al-Raddadi R. M., Altirkawi K. A., Alvis-Guzman N., Alvis-Zakzuk N. J., Amini S., Amini-Rarani M., Amiri F., Amit A. M. L., Amugsi D. A., Ancuceanu R., Anderlini D., Andrei C. L., Androudi S., Ansari F., Ansari-Moghaddam A., Antonio C. A. T., Antony C. M., Antriyandarti E., Anvari D., Anwer R., Arabloo J., Arab-Zozani M., Aravkin A. Y., Aremu O., Ärnlöv J., Asaad M., Asadi-Aliabadi M., Asadi-Pooya A. A., Athari S. S., Atout M. M. W., Ausloos M., Avila-Burgos L., Ayala Quintanilla B. P., Ayano G., Ayanore M. A., Aynalem Y. A., Aynalem G. L., Ayza M. A., Azari S., Azzopardi P. S., B D. B., Babaee E., Badiye A. D., Bahrami M. A., Baig A. A., Bakhshaei M. H., Bakhtiari A., Bakkannavar S. M., Balachandran A., Banach M., Banerjee S. K., Banik P. C., Bante A. B., Bante S. A., Barker-Collo S. L., Bärnighausen T. W., Barrero L. H., Bassat Q., Basu S., Baune B. T., Bayati M., Baye B. A., Bedi N., Beghi E., Behzadifar M., Bekuma T. T. T., Bell M. L., Bensenor I. M., Berman A. E., Bernabe E., Bernstein R. S.,	2020	Measuring universal health coverage based on an index of effective coverage of health services in 204 countries and territories, 1990–2019: a systematic analysis for the Global Burden of Disease Study 2019	Participants

Appendix 2. Excluded reports with reason for exclusion

	Bhagavathula A. S., Bhandari D., Bhardwaj P., Bhat A. G., Bhattacharyya K., Bhattarai S., Bhutta Z. A., Bijani A., Bikbov B., Bilano V., Biondi A., Birihane B. M., Bockarie M. J., Bohlouli S., Bojia H. A., Bolla S. R. R., Boloor A., Brady O. J., Braithwaite D., Briggs A. M., Briko N. I., Burugina Nagaraja S., Busse R., Butt Z. A., Caetano dos Santos F. L., Cahuana-Hurtado L., Cámera L. A., Cárdenas R., Carreras G., Carrero J. J., Carvalho F., Castaldelli-Maia J. M., Castañeda-Orjuela C. A., Castelpietra G., Castro F., Catalá-López F., Cederroth C. R., Cerin E., Chandan J. S., Chang A. Y., Charan J., Chattu V. K., Chaturvedi S., Chin K. L., Cho D. Y., Choi J. Y. J., Christensen H., Chu D. T., Chung M. T., Ciobanu L. G., Cirillo M., Compton K., Cortesi P. A., Costa V. M., Cousin E., Dahlawi S. M. A., Damiani G., Dandona L., Dandona R., Darega Gela J., Darwesh A. M., Daryani A., Dash A. P., Davey G., Dávila-Cervantes C. A., Davletov K., De Neve J. W., Denova-Gutiérrez E., Deribe K., Dervenis N., Desai R., Dharmaratne S. D., Dhungana G. P., Dianatinasab M., Dias da Silva D., Diaz D., Dippenaar I. N., Do H. T., Dorostkar F., Doshmangir L., Duncan B. B., Duraes A. R., Eagan A. W., Edvardsson D., El Sayed I., El Tantawi M., Elgendy I. Y., Elyazar I. R., Eskandari K., Eskandarieh S., Esmaeilnejad S., Esteghamati A., Ezekannagha O., Farag T., Farahmand M., Faraon E. J. A., Farinha C. S. E. S., Farioli A., Faris P. S., Faro A., Fazlzadeh M., Feigin V. L., Fernandes E., Ferrara P., Feyissa G. T., Filip I., Fischer F., Fisher J. L., Flor L. S., Foigt N. A., Folayan M. O., Fomenkov A. A., Foroutan M., Francis J. M., Fu W., Fukumoto T., Furtado J. M., Gad M. M., Gaidhane A. M., Gakidou E., Galles N. C., Gallus S., Gardner W. M., Geberemariyam B. S., Gebrehiwot A. M., Gebremeskel L. G., Gebremeskel G. G., Gesesew	

Appendix 2. Excluded reports with reason for exclusion

	H. A., Ghadiri K., Ghafourifard M., Ghashghaee A., Ghith N., Gholamian A., Gilani S. A., Gill P. S., Gill T. K., Ginindza T. G., Gitimoghaddam M., Giussani G., Glagn M., Gnedovskaya E. V., Godinho M. A., Goharinezhad S., Gopalani S. V., Goudarzian A. H., Goulart B. N. G., Gubari M. I. M., Guimarães R. A., Guled R. A., Gultie T., Guo Y., Gupta R., Hafezi-Nejad N., Hafiz A., Haile T. G., Hamadeh R. R., Hameed S., Hamidi S., Han C., Han H., Handiso D. W., Hanif A., Hankey G. J., Haro J. M., Hasaballah A. I., Hasan M. M., Hashi A., Hassan S., Hassan A., Hassanipour S., Hassankhani H., Havmoeller R. J., Hay S. I., Hayat K., Heidari G., Heidari-Soureshjani R., Hendrie D., Herteliu C., Hird T. R., Ho H. C., Hole M. K., Holla R., Hoogar P., Hopf K. P., Horita N., Hossain N., Hosseini M., Hosseinzadeh M., Hostiuc M., Hostiuc S., Househ M., Hsieh V. C. R., Hu G., Huda T. M., Humayun A., Hwang B. F., Iavicoli I., Ibitoye S. E., Ikeda N., Ilesanmi O. S., Ilic M. D., Inbaraj L. R., Iqbal U., Irvani S. S. N., Irvine C. M. S., Islam M. M., Islam S. M. S., Islami F., Iso H., Iwu C. J., Jaafari J., Jadidi-Niaragh F., Jafarinia M., Jahagirdar D., Jahani M. A., Jahanmehr N., Jakovljevic M., Janjani H., Javaheri T., Jayatilleke A. U., Jenabi E., Jha R. P., Jha V., Ji J. S., Jia P., John-Akinola Y. O., Jonas J. B., Joukar F., Jozwiak J. J., Jürisson M., Kabir Z., Kalankesh L. R., Kalhor R., Kamath A. M., Kanchan T., Kapoor N., Karami Matin B., Karanikolos M., Karimi S. M., Kassebaum N. J., Katikireddi S. V., Kayode G. A., Keiyoro P. N., Khader Y. S., Khammarnia M., Khan M., Khan E. A., Khang Y. H., Khatab K., Khater A. M., Khater M. M., Khatib M. N., Khayamzadeh M., Khubchandani J., Kianipour N., Kim Y. J., Kimokoti R. W., Kinfu Y., Kisa A., Kissimova-Skarbek K., Kivimäki M., Kneib C. J., Kocarnik J. M.,	

Appendix 2. Excluded reports with reason for exclusion

	Kochhar S., Kohler S., Kopec J. A., Korotkova A. V., Korshunov V. A., Kosen S., Kotlo A., Koul P. A., Koyanagi A., Krishan K., Krohn K. J., Kugbey N., Kulkarni V., Kumar G. A., Kumar N., Kumar M., Kurmi O. P., Kusuma D., Kyu H. H., La Vecchia C., Lacey B., Lal D. K., Lalloo R., Landires I., Lansingh V. C., Larsson A. O., Lasrado S., Lau K. M. M., Lauriola P., Lazarus J. V., Ledesma J. R., Lee P. H., Lee S. W. H., Leever A. T., LeGrand K. E., Leigh J., Leonardi M., Li S., Lim S. S., Lim L. L., Liu X., Logroscino G., Lopez A. D., Lopukhov P. D., Lotufo P. A., Lu A., Ma J., Madadin M., Mahasha P. W., Mahmoudi M., Majeed A., Malagón-Rojas J. N., Maleki S., Malta D. C., Mansouri B., Mansournia M. A., Martini S., Martins-Melo F. R., Martopullo I., Massenburg B. B., Mastrogiacomo C. I., Mathur M. R., McAlinden C., McKee M., Medina-Solís C. E., Meharie B. G., Mehndiratta M. M., Mehrabi Nasab E., Mehri F., Mehrotra R., Mekonnen T., Melese A., Memiah P. T. N., Mendoza W., Menezes R. G., Mensah G. A., Meretoja T. J., Meretoja A., Mestrovic T., Miazgowski B., Michalek I. M., Mirrakhimov E. M., Mirzaei M., Mirzaei-Alavijeh M., Mitchell P. B., Moazen B., Moghadaszadeh M., Mohamadi E., Mohammad Y., Mohammad D. K., Mohammad Gholi Mezerji N., Mohammadian-Hafshejani A., Mohammed S., Mohammed J. A., Mokdad A. H., Monasta L., Mondello S., Moradi M., Moradi-Lakeh M., Moradzadeh R., Moraga P., Morgado-da-Costa J., Morrison S. D., Mosapour A., Mosser J. F., Mousavi Khaneghah A., Muriithi M. K., Mustafa G., Nabhan A. F., Naderi M., Nagarajan A. J., Naghavi M., Naghshtabrizi B., Naimzada M. D., Nangia V., Nansseu J. R., Nayak V. C., Nazari J., Ndejjo R., Negoi I., Negoi R. I., Neupane S., Ngari K. N., Nguefack-	

Appendix 2. Excluded reports with reason for exclusion

	Tsague G., Ngunjiri J. W., Nguyen C. T., Nguyen D. N., Nguyen H. L. T., Nnaji C. A., Nomura S., Norheim O. F., Noubiap J. J., Nowak C., Nunez-Samudio V., Otoiu A., Ogbo F. A., Oghenetega O. B., Oh I. H., Okunga E. W., Oladnabi M., Olagunju A. T., Olusanya J. O., Olusanya B. O., Oluwasanu M. M., Omar Bali A., Omer M. O., Ong K. L., Onwujekwe O. E., Ortega-Altamirano D. V. V., Ortiz A., Ostojic S. M., Otstavnov N., Otstavnov S. S., Øverland S., Owolabi M. O., Padubidri J. R., Pakhale S., Palladino R., Pana A., Panda-Jonas S., Pangaribuan H. U., Pathak M., Patton G. C., Paudel S., Pazoki Toroudi H., Pease S. A., Peden A. E., Pennini A., Peprah E. K., Pereira J., Pigott D. M., Pilgrim T., Pilz T. M., Pinheiro M., Piradov M. A., Pirsaheb M., Pokhrel K. N., Postma M. J., Pourjafar H., Pourmalek F., Pourmirza Kalhori R., Pourshams A., Prada S. I., Pribadi D. R. A., Pupillo E., Quazi Syed Z., Radfar A., Rafiee A., Rafiei A., Raggi A., Rahim F., Rahman M. A., Rajabpour-Sanati A., Rana S. M., Ranabhat C. L., Rao S. J., Rasella D., Rashedi V., Rath G. K., Rathi P., Rawaf S., Rawaf D. L., Rawal L., Rawassizadeh R., Razo C., Renjith V., Renzaho A. M. N., Reshmi B., Rezaei N., Riahi S. M., Ribeiro D. C., Rickard J., Roberts N. L. S., Roever L., Romoli M., Ronfani L., Roshandel G., Rubagotti E., Rwegerera G. M., Sabour S., Sachdev P. S., Saddik B., Sadeghi M., Sadeghi E., Safari Y., Sagar R., Sahebkar A., Sahraian M. A., Sajadi S. M., Salahshoor M. R., Salem M. R. R., Salem H., Salomon J., Samadi Kafil H., Samy A. M., Sanabria J., Santric-Milicevic M. M., Saraswathy S. Y. I., Sarmiento-Suárez R., Sartorius B., Sarveazad A., Sathian B., Sathish T., Sattin D., Savic M., Sawyer S. M., Saxena D., Sbarra A. N., Schaeffer L. E., Schiavolin S., Schmidt M. I., Schutte A. E., Schwebel D. C.,	

Appendix 2. Excluded reports with reason for exclusion

	Schwendicke F., Seedat S., Sha F., Shahabi S., Shaheen A. A., Shaikh M. A., Shamsizadeh M., Shannawaz M., Sharafi K., Sharara F., Sharifi H., Shaw D. H., Sheikh A., Sheikhtaheri A., Shetty B. S. K., Shibuya K., Shiferaw W. S., Shigematsu M., Shin J. I., Shiri R., Shirkoohi R., Shivakumar K. M., Shrime M. G., Shuval K., Siabani S., Sierpinski R., Sigfusdottir I. D., Sigurvinsdottir R., Silva D. A. S., Silva J. P., Simonetti B., Simpson K. E., Singh J. A., Singh P., Sinha D. N., Skryabin V. Y., Smith E. U. R., Soheili A., Soltani S., Soofi M., Sorensen R. J. D., Soriano J. B., Sorrie M. B., Soyiri I. N., Spurlock E. E., Sreeramareddy C. T., Stanaway J. D., Steel N., Stein C., Stokes M. A., Sufiyan M. B., Suleria H. A. R., Sultan I., Szumowski L., Tabarés-Seisdedos R., Tabuchi T., Tadakamadla S. K., Taddele B. W., Tadesse D. B., Taherkhani A., Tamiru A. T., Tanser F. C., Tareque M. I., Tarigan I. U., Teagle W. L., Tediosi F., Tefera Y. G. G., Tela F. G., Tessema Z. T., Thakur B., Titova M. V., Tonelli M., Topor-Madry R., Topouzis F., Tovani-Palone M. R. R., Tran B. X., Travillian R., Troeger C. E., Tudor Car L., Uddin R., Ullah I., Umeokonkwo C. D., Unnikrishnan B., Upadhyay E., Uthman O. A., Vacante M., Valdez P. R., Varughese S., Vasankari T. J., Vasseghian Y., Venketasubramanian N., Violante F. S., Vlassov V., Vollset S. E., Vongpradith A., Vos T., Waheed Y., Walters M. K., Wamai R. G., Wang H., Wang Y. P., Weintraub R. G., Weiss J., Werdecker A., Westerman R., Wilner L. B., Woldu G., Wolfe C. D. A., Wu A. M., Wulf Hanson S., Xie Y., Yahyazadeh Jabbari S. H., Yamagishi K., Yano Y., Yaya S., Yazdi-Feyzabadi V., Yearwood J. A., Yeshitila Y. G., Yip P., Yonemoto N., Younis M. Z., Yousefi Z., Yousefinezhadi T., Yusefzadeh H., Zadey S., Zahirian Moghadam T., Zaidi	

Appendix 2. Excluded reports with reason for exclusion

	S. S., Zaki L., Zaman S. B., Zamanian M., Zandian H., Zastrozhin M. S., Zewdie K. A., Zhang Y., Zhao X. J. G., Zhao Y., Zheng P., Zhu C., Ziapour A., Zlavog B. S., Zodpey S.,Murray C. J. L.			
D_1886	Lu C., Chin B., Lewandowski J. L., Basinga P., Hirschhorn L. R., Hill K., Murray M.,Binagwaho A.	2012	Towards universal health coverage: An evaluation of Rwanda Mutuelles in its first eight years	Participants
D_1893	Luoga E.,Gamell A.	2019	Prevention of mother-to-child transmission of HIV - An update from rural Africa	Type of report
D_1902	Lyellu H. Y., Hussein T. H., Wandel M., Stray-Pedersen B., Mgongo M.,Msuya S. E.	2020	Prevalence and factors associated with early initiation of breastfeeding among women in Moshi municipal, northern Tanzania	Context
D_1932	Mafuta W., Zuwarimwe J.,Mwale M.	2021	Universal WASH coverage; What it takes for fragile states. Case of Jariban district in Somalia	Concept
D_1951	Makoae M. G.,Jubber K.	2008	Confidentiality or continuity? Family caregivers' experiences with care for HIV/AIDS patients in home-based care in Lesotho	Concept
D_1965	Mamba K. C., Muula A. S.,Stones W.	2017	Facility-imposed barriers to early utilization of focused antenatal care services in Mangochi District, Malawi - A mixed methods assessment	Participants
D_1966	Mamdani Masuma	2020	Universal Health Coverage: From aspirations to reality	Type of report
D_1972	Manoufi D., Kabore W. C., Yahannon C. N., Dumont A.,Ridde V.	2021	Improving provision of mother-and-child care in Chad at the community level: A quasi-experimental study	Concept
D_1973	Manzi A., Munyaneza F., Mujawase F., Banamwana L., Sayinzoga F., Thomson D. R., Ntaganira J.,Hedt-Gauthier B. L.	2014	Assessing predictors of delayed antenatal care visits in Rwanda: A secondary analysis of Rwanda demographic and health survey 2010	Context

Appendix 2. Excluded reports with reason for exclusion

D_1975	Mao W., Ogbuoji O., Watkins D., Bharali I., Boateng E., Diab M. M., Dwomoh D., Jamison D. T., Kumar P., McDade K. K., Nonvignon J., Ogundeji Y., Zeng F. G., Zimmerman A.,Yamey G.	2021	Achieving global mortality reduction targets and universal health coverage: The impact of COVID-19	Context
D_1979	Marbán-Castro E., Sacoor C., Nhacolo A., Augusto O., Jamisse E., López-Varela E., Casellas A., Aponte J. J., Bassat Q., Sigauque B., Macete E.,Garcia-Basteiro A. L.	2018	BCG vaccination in southern rural Mozambique: An overview of coverage and its determinants based on data from the demographic and health surveillance system in the district of Manhiça	Context
D_1996	Marukutira T., Stoove M., Lockman S., Mills L. A., Gaolathe T., Lebelonyane R., Jarvis J. N., Kelly S. L., Wilson D. P., Luchters S., Crowe S. M.,Hellard M.	2018	A tale of two countries: progress towards UNAIDS 90-90-90 targets in Botswana and Australia	Type of report
D_1999	Mary M., Diop A., Sheldon W. R., Yenikoye A.,Winikoff B.	2019	Scaling up interventions: Findings and lessons learned from an external evaluation of Niger's National Initiative to reduce postpartum hemorrhage	Context
D_2001	Masaninga F., Mukumbuta N., Ndhlovu K., Hamainza B., Wamulume P., Chanda E., Banda J., Mwanza-Ingwe M., Miller J. M., Ameneshewa B., Mnzava A.,Kawesha-Chizema E.	2018	Insecticide-treated nets mass distribution campaign: Benefits and lessons in Zambia	Context
D_2008	Masong M. C., Ozano K., Tagne M. S., Tchoffo M. N., Ngang S., Thomson R., Theobald S., Tchuente L. A. T.,Kouokam E.	2021	Achieving equity in UHC interventions: who is left behind by neglected tropical disease programmes in Cameroon?	Context
D_2022	Mbachu C. O., Onwujekwe O. E., Uzochukwu B. S., Uchegbu E., Oranuba J.,Ilika A. L.	2012	Examining equity in access to long-lasting insecticide nets and artemisinin-based combination therapy in Anambra State, Nigeria	Concept
D_2062	McIntyre D.,Meheus F.	2013	Achieving sustainable universal health coverage in low- and middle-income countries	Type of report
D_2076	Mebratie Anagaw D., Sparrow Robert, Yilma Zelalem, Abebaw Degnet, Alemu Getnet,Bedi Arjun S.	2019	The impact of Ethiopia's pilot community based health insurance	Context

Appendix 2. Excluded reports with reason for exclusion

			scheme on healthcare utilization and cost of care	
D_2120	Mihigo Richard, Okeibunor Joseph, Masresha Balcha, Mkanda Pascal, Poy Alain, Zawaira Felicitas,Cabore Joseph	2018	IMMUNIZATION AND VACCINE DEVELOPMENT: Progress towards High and Equitable Immunization Coverage in the Africa Region	Type of report
D_2127	Mills A., Ataguba J. E., Akazili J., Borghi J., Garshong B., Makawia S., Mtei G., Harris B., MacHa J., Meheus F.,McIntyre D.	2012	Equity in financing and use of health care in Ghana, South Africa, and Tanzania: Implications for paths to universal coverage	Context
D_2128	Mimiko O.	2017	Experiences with Universal Health Coverage of Maternal Health Care in Ondo State, Nigeria, 2009-2017	Type of report
D_2147	Mkandawire Paul, Kangmennaang Joseph, Walker Chad, Antabe Roger, Atuoye Kilian,Luginaah Isaac	2021	Pregnancy intention and gestational age at first antenatal care visit in Lesotho	Context
D_2150	Mladovsky P.,Ba M.	2017	Removing user fees for health services: A multi-epistemological perspective on access inequities in Senegal	Context
D_2152	Mlangeni N., Du Preez K., Mokone M., Malotle M., Kisting S., Ramodike J.,Zungu M.	2022	HIV and TB Workplace Program for Street Vendors: A Situational Analysis	Context
D_2161	Mogeni P., Williams T. N., Fegan G., Nyundo C., Bauni E., Mwai K., Omedo I., Njuguna P., Newton C. R., Osier F., Berkley J. A., Hammitt L. L., Lowe B., Mwambingu G., Awuondo K., Mturi N., Peshu N., Snow R. W., Noor A., Marsh K.,Bejon P.	2016	Age, Spatial, and Temporal Variations in Hospital Admissions with Malaria in Kilifi County, Kenya: A 25-Year Longitudinal Observational Study	Context
D_2166	Mohammed A., Agwu P.,Okoye U.	2020	When Primary Healthcare Facilities are Available but Mothers Look the Other Way: Maternal Mortality in Northern Nigeria	Concept
D_2172	Molla S., Tsehay C. T.,Gebremedhin T.	2020	Urban health extension program and health services utilization in northwest Ethiopia: A community-based study	Concept
D_2183	Montgomery M. A., Bartram J.,Elimelech M.	2009	Increasing functional sustainability of	Context

Appendix 2. Excluded reports with reason for exclusion

			water and sanitation supplies in rural sub-saharan Africa	
D_2210	Mtei G., Makawia S.,Masanja H.	2014	Monitoring and Evaluating Progress towards Universal Health Coverage in Tanzania	Context
D_2224	Mukumbang Ferdinand C., Knight Lucia, Masquillier Caroline, Delport Anton, Sematlane Neo, Dube Lorraine Tanyaradzwa, Lembani Martina,Wouters Edwin	2019	Household-focused interventions to enhance the treatment and management of HIV in low- and middle-income countries: a scoping review	Concept
D_2226	Mulat A. K., Mao W., Bharali I., Balkew R. B.,Yamey G.	2022	Scaling up community-based health insurance in Ethiopia: a qualitative study of the benefits and challenges	Context
D_2241	Munthali A. C., Mannan H., MacLachlan M., Swartz L., Makupe C. M.,Chilimampunga C.	2014	Non-use of Formal Health Services in Malawi: Perceptions from Non-users	Context
D_2264	Mwase T., Lohmann J., Hamadou S., Brenner S., Somda S. M. A., Hien H., Hillebrecht M.,De Allegri M.	2022	Can Combining Performance-Based Financing With Equity Measures Result in Greater Equity in Utilization of Maternal Care Services? Evidence From Burkina Faso	Context
D_2272	Nabyonga Orem J., Mugisha F., Kirunga C., Macq J.,Criel B.	2011	Abolition of user fees: the Uganda paradox	Concept
D_2276	Nachega J. B., Adetokunboh O., Uthman O. A., Knowlton A. W., Altice F. L., Schechter M., Galarraga O., Geng E., Peltzer K., Chang L. W., Van Cutsem G., Jaffar S. S., Ford N., Mellins C. A., Remien R. H.,Mills E. J.	2016	Community-Based Interventions to Improve and Sustain Antiretroviral Therapy Adherence, Retention in HIV Care and Clinical Outcomes in Low- and Middle-Income Countries for Achieving the UNAIDS 90-90-90 Targets	Context
D_2288	Nakovics M. I., Brenner S., Bongololo G., Chinkhumba J., Kalmus O., Leppert G.,De Allegri M.	2020	Determinants of healthcare seeking and out-of-pocket expenditures in a "free" healthcare system: Evidence from rural Malawi	Context
D_2311	Navarrete Lucia Fiestas, Ghislandi Simone, Stuckler	2019	Inequalities in the benefits of national	Context

Appendix 2. Excluded reports with reason for exclusion

	David, Tediosi Fabrizio,Fiestas Navarrete Lucia		health insurance on financial protection from out-of-pocket payments and access to health services: cross-sectional evidence from Ghana	
D_2341	Newell R., Spillman I.,Newell M. L.	2017	The use of facilities for labor and delivery: The views of women in rural Uganda	Context
D_2345	Ng'ambi W., Mangal T., Phillips A., Colbourn T., Mfutso-Bengo J., Revill P.,Hallett T. B.	2020	Factors associated with healthcare seeking behaviour for children in Malawi: 2016	Context
D_2349	Ngcobo S. J., Makhado L.,Sehularo L. A.	2022	HIV care and support services offered in mobile health clinics: a systematic review	Context
D_2363	Nilsen K., Tejedor-Garavito N., Leasure D. R., Utazi C. E., Ruktanonchai C. W., Wigley A. S., Dooley C. A., Matthews Z.,Tatem A. J.	2021	A review of geospatial methods for population estimation and their use in constructing reproductive, maternal, newborn, child and adolescent health service indicators	Context
D_2365	Njagi P., Arsenijevic J.,Groot W.	2020	Cost-related unmet need for healthcare services in Kenya	Context
D_2366	Njagi P., Groot W.,Arsenijevic J.	2021	Impact of household shocks on access to healthcare services in Kenya: A propensity score matching analysis	Context
D_2373	Njue C., Sharmin S.,Dawson A.	2022	Models of Maternal Healthcare for African refugee women in High-Income Countries: A Systematic Review	Participants
D_2378	Nketsia W., Mprah W. K., Opoku M. P., Juventus D.,Amponteng M.	2022	Achieving universal reproductive health coverage for deaf women in Ghana: an explanatory study of knowledge of contraceptive methods, pregnancy and safe abortion practices	Context
D_2394	Nonaka D., Maazou A., Yamagata S., Oumarou I., Uchida T., Yacouba H. J. G., Kobayashi J., Takeuchi	2012	Distribution of subsidized insecticide-treated bed nets through a community	Concept

Appendix 2. Excluded reports with reason for exclusion

	T.,Mizoue T.		health committee in Boboye Health District, Niger	
D_2398	Nove A., Friberg I. K., de Bernis L., McConville F., Moran A. C., Najjemba M., ten Hoope-Bender P., Tracy S.,Homer C. S. E.	2021	Potential impact of midwives in preventing and reducing maternal and neonatal mortality and stillbirths: a Lives Saved Tool modelling study	Context
D_2422	Nundoochan A.	2021	Improving equity in the distribution and financing of health services in Mauritius, a small island state with deeply rooted welfare state standards	Context
D_2431	Nyamugira A. B., Richter A., Furaha G.,Flessa S.	2022	Towards the achievement of universal health coverage in the Democratic Republic of Congo: does the Country walk its talk?	Concept
D_2432	Nyandekwe M., Kakoma J. B.,Nzayirambaho M.	2018	The health-related millennium development goals (MDGs) 2015: Rwanda performance and contributing factors	Context
D_2435	Nyandekwe Medard, Nzayirambaho Manasse,Baptiste Kakoma Jean	2014	Universal health coverage in Rwanda: dream or reality	Context
D_2439	Nyonator F., Ofosu A., Segbafah M.,d'Almeida S.	2014	Monitoring and Evaluating Progress towards Universal Health Coverage in Ghana	Concept
D_2440	O’Connell Thomas S., Bedford K. Juliet A., Thiede Michael,McIntyre Di	2015	Synthesizing qualitative and quantitative evidence on non-financial access barriers: implications for assessment at the district level	Context
D_2452	Obisie-Nmehielle N., Kalule-Sabiti I.,Palamuleni M.	2022	Factors associated with knowledge about family planning and access to sexual and reproductive health services by sexually active immigrant youths in Hillbrow, South Africa: a cross-sectional study	Context
D_2454	O'Brien Daniel P., Mills Clair, Hamel Catherine, Ford	2009	Universal access: the benefits and	Concept

Appendix 2. Excluded reports with reason for exclusion

	Nathan,Pottie Kevin		challenges in bringing integrated HIV care to isolated and conflict affected populations in the Republic of Congo	
D_2458	Ochalek J., Manthalu G.,Smith P. C.	2020	Squaring the cube: Towards an operational model of optimal universal health coverage	Context
D_2466	Odeyemi I. A.,Nixon J.	2013	Assessing equity in health care through the national health insurance schemes of Nigeria and Ghana: A review-based comparative analysis	Context
D_2472	Odufuwa O. G., Ross A., Mlacha Y. P., Juma O., Mmbaga S., Msellemu D.,Moore S.	2020	Household factors associated with access to insecticide-treated nets and house modification in Bagamoyo and Ulanga districts, Tanzania	Context
D_2487	Ojikutu B., Jack C.,Ramjee G.	2007	Provision of antiretroviral therapy in South Africa: Unique challenges and remaining obstacles	Type of report
D_2493	Okech T. C.,Lelegwe S. L.	2015	Analysis of Universal Health Coverage and Equity on Health Care in Kenya	Context
D_2499	Okoli C. I.,Cleary S. M.	2011	Socioeconomic status and barriers to the use of free antiretroviral treatment for HIV/AIDS in Enugu State, south-eastern Nigeria	Context
D_2502	Okonofua F., Ntoimo L., Ogungbangbe J., Anjorin S., Imongan W.,Yaya S.	2018	Predictors of women's utilization of primary health care for skilled pregnancy care in rural Nigeria	Context
D_2512	Okoronkwo I. L., Onwujekwe O. E.,Ani F. O.	2014	The long walk to universal health coverage: patterns of inequities in the use of primary healthcare services in Enugu, Southeast Nigeria	Context
D_2523	Oladele E. A., Badejo O. A., Obanubi C., Okechukwu E. F., James E., Owhonda G., Omeh O. I., Abass M., Negedu-Momoh O. R., Ojehomon N., Oqua D., Raj-Pandey S., Khamofu H.,Torpey K.	2018	Bridging the HIV treatment gap in Nigeria: Examining community antiretroviral treatment models	Concept

Appendix 2. Excluded reports with reason for exclusion

D_2525	Olakunde B. O., Adeyinka D. A., Olakunde O. A., Ozigbu C. E., Ndukwe C. D., Oladele T., Wakdok S., Udemezue S.,Ezeanolue E. E.	2019	Correlates of antiretroviral coverage for prevention of mother-to-child transmission of HIV in sub-Saharan Africa	Concept
D_2527	Olaleye A., Ogwumike F.,Olaniyan O.	2013	Inequalities in access to healthcare services among people living with HIV/AIDS in Nigeria	Context
D_2542	Olyaeemanesh A., Woldemichael A., Takian A.,Sari A. A.	2019	Availability and inequality in accessibility of health centre-based primary healthcare in Ethiopia	Context
D_2543	Omoniyi O. S.,Williams I.	2020	Realist Synthesis of the International Theory and Evidence on Strategies to Improve Childhood Vaccination in Low- and Middle-Income Countries: Developing Strategies for the Nigerian Healthcare System	Concept
D_2560	Onwujekwe O., Obi F., Ichoku H., Ezumah N., Okeke C., Ezenwaka U., Uzochukwu B.,Wang H.	2019	Assessment of a free maternal and child health program and the prospects for program re-activation and scale-up using a new health fund in Nigeria	Context
D_2562	Onwujekwe O., Uzochukwu B., Dike N., Okoli C., Eze S.,Chukwuogo O.	2009	Are there geographic and socio-economic differences in incidence, burden and prevention of malaria? A study in southeast Nigeria	Context
D_2567	Onyemaechi S.,Ezenwaka U.	2022	Influence of sub-national social health insurance scheme on enrollees' health seeking behaviour in Anambra state, Nigeria: a pre and post study	Context
D_2581	Orem J. N., Mugisha F., Kirunga C., Macq J.,Criel B.	2011	Abolition of user fees: the Uganda paradox	Context
D_2597	Otieno P. O., Wambiya E. O. A., Mohamed S. M., Mutua M. K., Kibe P. M., Mwangi B.,Donfouet H. P. P.	2020	Access to primary healthcare services and associated factors in urban slums in Nairobi-Kenya	Context

Appendix 2. Excluded reports with reason for exclusion

D_2623	Ozano Kim, Dean Laura, Yoshimura Mami, MacPherson Eleanor, Linou Natalia, Otmani Del Barrio Mariam, Halleux Christine M., Ogundahunsi Olumide,Theobald Sally	2020	A call to action for universal health coverage: Why we need to address gender inequities in the neglected tropical diseases community	Type of report
D_2643	Pariente W.	2017	URBANIZATION IN SUB-SAHARAN AFRICA AND THE CHALLENGE OF ACCESS TO BASIC SERVICES	Type of report
D_2646	Parmar D., De Allegri M., Savadogo G.,Sauerborn R.	2014	Do community-based health insurance schemes fulfil the promise of equity? A study from Burkina Faso	Context
D_2665	Peltzer K., Williams J. S., Kowal P., Negin J., Snodgrass J. J., Yawson A., Minicuci N., Thiele L., Phaswana-Mafuya N., Biritwum R. B., Naidoo N.,Chatterji S.	2014	Universal health coverage in emerging economies: findings on health care utilization by older adults in China, Ghana, India, Mexico, the Russian Federation, and South Africa	Context
D_2710	Plazy M., Dabis F., Naidu K., Orne-Gliemann J., Barnighausen T.,Dray-Spira R.	2015	Change of treatment guidelines and evolution of ART initiation in rural South Africa: data of a large HIV care and treatment programme	Concept
D_2715	Plucinski M. M., Chicuecue S., Macete E., Chambe G. A., Muguande O., Matsinhe G., Colborn J., Yoon S. S., Doyle T. J., Kachur S. P., Aide P., Alonso P. L., Guinovart C.,Morgan J.	2015	Sleeping arrangements and mass distribution of bed nets in six districts in central and northern Mozambique	Context
D_2716	Plucinski M. M., Chicuecue S., Macete E., Colborn J., Yoon S. S., Patrick Kachur S., Aide P., Alonso P., Guinovart C.,Morgan J.	2014	Evaluation of a universal coverage bed net distribution campaign in four districts in Sofala Province, Mozambique	Context
D_2721	Pons-Duran C., Llach M., Sacoor C., Sanz S., Macete E., Arikpo I., Ramírez M., Meremikwu M., Mbombo Ndombe D., Méndez S., Manun'Ebo M. F., Ramananjato R., Rabeza V. R., Tholandi M., Roman E., Pagnoni F., González R.,Menéndez C.	2021	Coverage of intermittent preventive treatment of malaria in pregnancy in four sub-Saharan countries: findings from household surveys	Context
D_2741	Quigley P., Green C., Soyoola M., Kureya T., Barber C.,Mubuyaeta K.	2018	Empowering women and communities to promote universal health coverage	Concept

Appendix 2. Excluded reports with reason for exclusion

			in rural Zambia	
D_2748	Radin M., Wong B., McManus C., Sinha S., Jeuland M., Larbi E., Tuffuor B., Biscoff N. K.,Whittington D.	2020	Benefits and costs of rural sanitation interventions in Ghana	Concept
D_2750	Radwan Ghada,Adawy Adel	2019	The Egyptian health map: a guide for evidence-based decisionmaking	Concept
D_2788	Ravindran T. K. S.,Fonn S.	2011	Are social franchises contributing to universal access to reproductive health services in low-income countries?	Concept
D_2789	Ravit M., Ravalihasy A., Audibert M., Ridde V., Bonnet E., Raffalli B., Roy F. A., N'Landu A.,Dumont A.	2020	The impact of the obstetrical risk insurance scheme in Mauritania on maternal healthcare utilization: a propensity score matching analysis	Concept
D_2804	Reid M. J. A., Arinaminpathy N., Bloom A., Bloom B. R., Boehm C., Chaisson R., Chin D. P., Churchyard G., Cox H., Ditiu L., Dybul M., Farrar J., Fauci A. S., Fekodu E., Fujiwara P. I., Hallett T. B., Hanson C. L., Harrington M., Herbert N., Hopewell P. C., Ikeda C., Jamison D. T., Khan A. J., Koek I., Krishnan N., Motsoaledi A., Pai M., Raviglione M. C., Sharman A., Small P. M., Swaminathan S., Temesgen Z., Vassall A., Venkatesan N., van Weezenbeek K., Yamey G., Agins B. D., Arexandru S., Andrews J. R., Beyeler N., Bivol S., Brigden G., Cattamanchi A., Cazabon D., Crudu V., Daftary A., Dewan P., Doepel L. K., Eisinger R. W., Fan V., Fewer S., Furin J., Goldhaber-Fiebert J. D., Gomez G. B., Graham S. M., Gupta D., Kamene M., Khaparde S., Mailu E. W., Masini E. O., McHugh L., Mitchell E., Moon S., Osberg M., Pande T., Prince L., Rade K., Rao R., Remme M., Seddon J. A., Selwyn C., Shete P., Sachdeva K. S., Stallworthy G., Vesga J. F., Vilc V.,Goosby E. P.	2019	Building a tuberculosis-free world: The Lancet Commission on tuberculosis	Type of report
D_2831	Ricotta E. E., Boulay M., Ainslie R., Babalola S., Fotheringham M., Koenker H.,Lynch M.	2015	The use of mediation analysis to assess the effects of a behaviour change communication strategy on bed net	Concept

Appendix 2. Excluded reports with reason for exclusion

			ideation and household universal coverage in Tanzania	
D_2832	Ricotta E., Koenker H., Kilian A.,Lynch M.	2014	Are pregnant women prioritized for bed nets? An assessment using survey data from 10 African countries	Concept
D_2835	Ridde V., Belaid L., Samb O. M.,Faye A.	2014	Health system revenue collection in Burkina Faso from 1980 to 2012	Context
D_2836	Ridde V., Gautier L., Turcotte-Tremblay A. M., Sieleunou I.,Paul E.	2018	Performance-based Financing in Africa: Time to Test Measures for Equity	Type of report
D_2840	Ridde V.,Hane F.	2021	Universal health coverage: The roof has been leaking for far too long	Type of report
D_2853	Roa L., Jumbam D. T., Makasa E.,Meara J. G.	2019	Global surgery and the sustainable development goals	Type of report
D_2856	Roberts D. A., Ng M., Ikilezi G., Gasasira A., Dwyer-Lindgren L., Fullman N., Nalugwa T., Kamya M.,Gakidou E.	2015	Benchmarking health system performance across regions in Uganda: A systematic analysis of levels and trends in key maternal and child health interventions, 1990-2011	Concept
D_2866	Rohde J., Cousens S., Chopra M., Tangcharoensathien V., Black R., Bhutta Z. A.,Lawn J. E.	2008	30 years after Alma-Ata: has primary health care worked in countries?	Context
D_2871	Rombosia K., Oele E., Rangara N., Mwaura J., Mitto B., Ondura E., Onyango D.,Akoth C.	2019	EFFECT OF THE DISTANCES OF PUBLIC HEALTH FACILITIES FROM THE NEAREST MAJOR ROADS ON SKILLED DELIVERIES CONDUCTED IN KISUMU COUNTY, KENYA	Concept
D_2900	Rudasingwa M., De Allegri M., Mphuka C., Chansa C., Yeboah E., Bonnet E., Ridde V.,Chitah B. M.	2022	Universal health coverage and the poor: to what extent are health financing policies making a difference? Evidence from a benefit incidence analysis in Zambia	Context
D_2904	Ruhago G. M., Ngalesoni F. N.,Norheim O. F.	2012	Addressing inequity to achieve the maternal and child health millennium development goals: looking beyond averages	Context

Appendix 2. Excluded reports with reason for exclusion

D_2924	Sackou-Kouakou Julie-Ghislaine, Kouame Jerome, Tiade Marie-Laure, Desquith Aka Angele, Dagnogo Aissata, Hounsa-Alla Annita, Attia-Konan Regine, Coulibaly Madikiny,Kouadio Kouakou Luc	2021	Renunciation of care for financial reasons among women in a peri-urban neighborhood in Abidjan-Cote d'Ivoire	Context
D_2937	Salim A. M. A.,Hamed F. H. M.	2018	Exploring health insurance services in Sudan from the perspectives of insurers	Context
D_2949	Sanders David, Nandi Sulakshana, Labonte Ronald, Vance Carina,Van Damme Wim	2019	From primary health care to universal health coverage-one step forward and two steps back	Type of report
D_2958	Sanogo N'doh Ashken, Fantaye Arone Wondwossen,Yaya Sanni	2019	Universal Health Coverage and Facilitation of Equitable Access to Care in Africa	Context
D_2963	Sarkar A.	2019	Can shared standpipes fulfil the Sustainable Development Goal of universal access to safe water for urban poor in Kenya?	Concept
D_2967	Sato R.	2020	Vaccine stockouts and vaccination rate: Examination of unique administrative data from Nigeria	Concept
D_2969	Sayinzoga F., Tetui M., Van Der Velden K., Van Dillen J.,Bijlmakers L.	2019	Understanding variation in health service coverage and maternal health outcomes among districts in Rwanda - A qualitative study of local health workers' perceptions	Context
D_2973	Scheil-Adlung X., Behrendt T.,Wong L.	2015	Health sector employment: a tracer indicator for universal health coverage in national Social Protection Floors	Context
D_2975	Scherf T.,Ieee	2006	Policies for universal access to telecommunications in rural areas of developing countries - An institutional economics approach	Context
D_2986	Schutte A. E., Venkateshmurthy N. S., Mohan S.,Prabhakaran D.	2021	Hypertension in Low- and Middle-Income Countries	Concept

Appendix 2. Excluded reports with reason for exclusion

D_3030	Sharma J., Leslie H. H., Kundu F.,Kruk M. E.	2017	Poor Quality for Poor Women? Inequities in the Quality of Antenatal and Delivery Care in Kenya	Context
D_3034	Shawky S.	2010	Could the employment-based targeting approach serve egypt in moving towards a social health insurance model?	Context
D_3035	Sheff Mallory C., Bawah Ayaga A., Asuming Patrick O., Kyei Pearl, Kushitor Mawuli, Phillips James F.,Kachur S. Patrick	2020	Evaluating health service coverage in Ghana's Volta Region using a modified Tanahashi model	Concept
D_3041	Sherr K., Pfeiffer J., Mussa A., Vio F., Gimbel S., Micek M.,Gloyd S.	2009	The role of nonphysician clinicians in the rapid expansion of HIV care in mozambique	Context
D_3046	Shiferaw F.,Zolfo M.	2012	The role of information communication technology (ICT) towards universal health coverage: the first steps of a telemedicine project in Ethiopia	Context
D_3047	Shikuku D. N., Muganda M., Amunga S. O., Obwanda E. O., Muga A., Matete T.,Kisia P.	2019	Door - to - door immunization strategy for improving access and utilization of immunization Services in Hard-to-Reach Areas: a case of Migori County, Kenya	Concept
D_3115	Some E. R.,Meda N.	2014	Does the national program of prevention of mother to child transmission of HIV (PMTCT) reach its target in Ouagadougou, Burkina Faso?	Context
D_3123	Souteyrand Y. P., Collard V., Moatti J. P., Grubb I.,Guerma T.	2008	Free care at the point of service delivery: a key component for reaching universal access to HIV/AIDS treatment in developing countries	Type of report
D_3124	Souza J. P., Gülmezoglu A. M., Vogel J., Carroli G., Lumbiganon P., Qureshi Z., Costa M. J., Fawole B., Mugerwa Y., Nafiou I., Neves I., Wolomby-Molondo J.	2013	Moving beyond essential interventions for reduction of maternal mortality (the WHO Multicountry Survey on	Context

Appendix 2. Excluded reports with reason for exclusion

	J., Bang H. T., Cheang K., Chuyun K., Jayaratne K., Jayathilaka C. A., Mazhar S. B., Mori R., Mustafa M. L., Pathak L. R., Perera D., Rathavy T., Recidoro Z., Roy M., Ruyan P., Shrestha N., Taneepanichsku S., Tien N. V., Ganchimeg T., Wehbe M., Yadamsuren B., Yan W., Yunis K., Bataglia V., Cecatti J. G., Hernandez-Prado B., Nardin J. M., Narváez A., Ortiz-Panozo E., Pérez-Cuevas R., Valladares E., Zavaleta N., Armson A., Crowther C., Hogue C., Lindmark G., Mittal S., Pattinson R., Stanton M. E., Campodonico L., Cuesta C., Giordano D., Intarut N., Laopaiboon M., Bahl R., Martines J., Mathai M., Merialdi M.,Say L.		Maternal and Newborn Health): A cross-sectional study	
D_3127	Spaan E., Mathijssen J., Tromp N., McBain F., ten Have A.,Baltusser R.	2012	The impact of health insurance in Africa and Asia: a systematic review	Context
D_3147	Stanton Michelle, Molineux Andrew, Mackenzie Charles,Kelly-Hope Louise	2016	Mobile Technology for Empowering Health Workers in Underserved Communities: New Approaches to Facilitate the Elimination of Neglected Tropical Diseases	Concept
D_3148	Starrs A. M., Ezeh A. C., Barker G., Basu A., Bertrand J. T., Blum R., Coll-Seck A. M., Grover A., Laski L., Roa M., Sathar Z. A., Say L., Serour G. I., Singh S., Stenberg K., Temmerman M., Biddlecom A., Popinchalk A., Summers C.,Ashford L. S.	2018	Accelerate progress-sexual and reproductive health and rights for all: report of the Guttmacher-Lancet Commission	Concept
D_3161	Strachan C. E., Nuwa A., Muhangi D., Okui A. P., Helinski M. E. H.,Tibenderana J. K.	2016	What drives the consistent use of long-lasting insecticidal nets over time? A multi-method qualitative study in mid-western Uganda	Concept
D_3184	Sundewall J.,Poku N. K.	2018	Achieving sexual and reproductive health and rights through universal health coverage	Type of report
D_3219	Tafere T. E., Afework M. F.,Yalew A. W.	2018	Does antenatal care service quality influence essential newborn care (ENC) practices? in Bahir Dar City	Context

Appendix 2. Excluded reports with reason for exclusion

			Administration, North West Ethiopia: A prospective follow up study	
D_3233	Tamirat K. S., Tessema Z. T.,Kebede F. B.	2020	Factors associated with the perceived barriers of health care access among reproductive-age women in Ethiopia: a secondary data analysis of 2016 Ethiopian demographic and health survey	Context
D_3245	Tariku A., Alemu K., Gizaw Z., Muchie K. F., Derso T., Abebe S. M., Yitayal M., Fekadu A., Ayele T. A., Alemayehu G. A., Tsegaye A. T., Shimeka A.,Biks G. A.	2017	Mothers' education and ANC visit improved exclusive breastfeeding in Dabat health and Demographic surveillance system site, northwest Ethiopia	Context
D_3249	Taverne Bernard, Desclaux Alice, Delaporte Eric, Ndoye Ibra, Coll Seck Awa M.,Barre-Sinoussi Francoise	2013	Universal health coverage and HIV in resource-constrained countries: a critical juncture for research and action	Type of report
D_3275	Tessema Z. T., Worku M. G., Tesema G. A., Alamneh T. S., Teshale A. B., Yeshaw Y., Alem A. Z., Ayalew H. G.,Liyew A. M.	2022	Determinants of accessing healthcare in Sub-Saharan Africa: A mixed-effect analysis of recent Demographic and Health Surveys from 36 countries	Context
D_3297	Tinyami Erick Tandi, YongMin Cho, Akam Aba Jean-Cluade, Afoh Chick Ofilia, Seung Hun Ryu, Min Seok Choi, KyungHee Kim,Jae Wook Choi	2015	Cameroon public health sector: shortage and inequalities in geographic distribution of health personnel	Context
D_3319	Toyin Saraki H. E.	2015	From 12 candles to universal health care: transforming social determinants for maternal, newborn and child health	Type of report
D_3331	Tshililo A. R., Mangena-Netshikweta L., Nemathaga L. H.,Maluleke M.	2019	Challenges of primary healthcare nurses regarding the integration of HIV and AIDS services into primary healthcare in Vhembe district of Limpopo province, South Africa	Context

Appendix 2. Excluded reports with reason for exclusion

D_3352	Uebel K. E., Timmerman V., Ingle S. M., van Rensburg D. H. C. J.,Mollentze W. F.	2010	Towards universal ARV access: Achievements and challenges in Free State province, South Africa	Context
D_3364	Umeh C. A.	2018	Challenges toward achieving universal health coverage in Ghana, Kenya, Nigeria, and Tanzania	Context
D_3379	Valiani S.	2020	Structuring Sustainable Universal Health Care in South Africa	Type of report
D_3392	van Hees Suzanne G. M., O'Fallon Timothy, Hofker Miranda, Dekker Marleen, Polack Sarah, Banks Lena Morgon,Spaan Ernst J. A. M.	2019	Leaving no one behind? Social inclusion of health insurance in low- and middle-income countries: a systematic review	Context
D_3406	van Schalkwyk Elanie A.,Gerbe Berna	2021	Vulnerable mothers' experience of feeding their preterm infant in neonatal care	Context
D_3423	Vella S., Schwartländer B., Sow S. P., Eholie S. P.,Murphy R. L.	2012	The history of antiretroviral therapy and of its implementation in resource-limited areas of the world	Concept
D_3430	Verguet S., Olson Z. D., Babigumira J. B., Desalegn D., Johansson K. A., Kruk M. E., Levin C. E., Nugent R. A., Pecenka C., Shrime M. G., Memirie S. T., Watkins D. A.,Jamison D. T.	2015	Health gains and financial risk protection afforded by public financing of selected interventions in ethiopia: An extended cost-effectiveness analysis	Context
D_3434	Victora C. G., Rubens C. E.,Grp Gapps Review	2010	Global report on preterm birth and stillbirth (4 of 7): delivery of interventions	Type of report
D_3467	Wagstaff A.,Neelsen S.	2020	A comprehensive assessment of universal health coverage in 111 countries: a retrospective observational study	Concept
D_3469	Waiswa P., Peterson S., Tomson G.,Pariyo G. W.	2010	Poor newborn care practices - a population based survey in eastern Uganda	Context
D_3475	Wang H., Juma M. A., Rosemberg N.,Ulisubisya M. M.	2018	Progressive Pathway to Universal	Type of

Appendix 2. Excluded reports with reason for exclusion

			Health Coverage in Tanzania: A Call for Preferential Resource Allocation Targeting the Poor	report
D_3490	Wares F.,Falzon D.	2014	Progress in achieving universal access to care for multidrug-resistant tuberculosis (MDR-TB)	Not retrieved
D_3501	Weld K. K., Padden D., Ricciardi R.,Bibb S. C. G.	2009	Health Literacy Rates in a Sample of Active Duty Military Personnel	Context
D_3504	Were L. P. O., Were E., Wamai R., Hogan J.,Galarraga O.	2017	The Association of Health Insurance with institutional delivery and access to skilled birth attendants: evidence from the Kenya Demographic and health survey 2008-09	Concept
D_3506	Were L. P. O., Were E., Wamai R., Hogan J.,Galarraga O.	2020	Effects of social health insurance on access and utilization of obstetric health services: results from HIV plus pregnant women in Kenya	Concept
D_3510	West P. A., Protopopoff N., Rowland M., Cumming E., Rand A., Drakeley C., Wright A., Kivaju Z., Kirby M. J., Mosha F. W., Kisinza W.,Kleinschmidt I.	2013	Malaria Risk Factors in North West Tanzania: The Effect of Spraying, Nets and Wealth	Context
D_3511	Wharton-Smith A., Rassi C., Batisso E., Ortu G., King R., Endriyas M., Counihan H., Hamade P.,Getachew D.	2019	Gender-related factors affecting health seeking for neglected tropical diseases: findings from a qualitative study in Ethiopia	Context
D_3515	White J. A.,Rispel L. C.	2021	Policy exclusion or confusion? Perspectives on universal health coverage for migrants and refugees in South Africa	Context
D_3519	Wigley A. S., Tejedor-Garavito N., Alegana V., Carioli A., Ruktanonchai C. W., Pezzulo C., Matthews Z., Tatem A. J.,Nilsen K.	2020	Measuring the availability and geographical accessibility of maternal health services across sub-Saharan Africa	Context
D_3545	Woldemariam A. T.	2016	The administrator's perspective	Type of report

Appendix 2. Excluded reports with reason for exclusion

D_3554	Wolfe W. R., Weiser S. D., Steward W. T., Iacopino V.,Heisler M.	2009	Wolfe et al respond...Gamper A. Universal access to antiretroviral therapy and HIV stigma in Botswana. Am J Public Health 2009;99:968-9	Type of report
D_3557	Wondimu A., Cao Q., Asuman D., Almansa J., Postma M. J.,van Hulst M.	2020	Understanding the Improvement in Full Childhood Vaccination Coverage in Ethiopia Using Oaxaca-Blinder Decomposition Analysis	Concept
D_3563	Woog V.,Pembe A. B.	2013	Unsafe abortion in Tanzania: a review of the evidence	Type of report
D_3566	Worrall E., Were V., Matope A., Gama E., Olewe J., Mwambi D., Desai M., Kariuki S., Buff A. M.,Niessen L. W.	2020	Coverage outcomes (effects), costs, cost-effectiveness, and equity of two combinations of long-lasting insecticidal net (LLIN) distribution channels in Kenya: a two-arm study under operational conditions	Concept
D_3567	Woskie L. R.,Fallah M. P.	2019	Overcoming distrust to deliver universal health coverage: Lessons from Ebola	Context
D_3571	Wringe A., Cawley C., Szumilin E., Salumu L., Quiles I. A., Pasquier E., Masiku C.,Nicholas S.	2018	Retention in care among clinically stable antiretroviral therapy patients following a six-monthly clinical consultation schedule: findings from a cohort study in rural Malawi	Context
D_3576	Yaméogo W. M. E., Ouédraogo T. M.,Kouanda S.	2016	Local initiatives to access emergency obstetric and neonatal care in Burkina Faso	Context
D_3578	Yamson P., Tetteh J., DeGraft-Amoah D., Quansah H., Mensah G., Biritwum R.,Yawson A. E.	2021	Unmet Needs of Healthcare Services and Associated Factors among a Cohort of Ghanaian Adults: A Nationally Stratified Cross-Sectional Study Design	Context
D_3586	Yaya S., Bishwajit G., Ekholuenetale M., Shah V., Kadio B.,Udenigwe O.	2018	Factors associated with maternal utilization of health facilities for	Context

Appendix 2. Excluded reports with reason for exclusion

			delivery in Ethiopia	
D_3591	Yekabong R. C., Ebile W. A., Fon P. N.,Asongalem E. A.	2017	The impact of mass distribution of long lasting insecticide-treated bed-nets on the malaria parasite burden in the Buea Health District in South-West Cameroon: a hospital based chart review of patient's laboratory records	Context
D_3596	Ying Roger, Barnabas Ruanne V.,Williams Brian G.	2014	Modeling the implementation of universal coverage for HIV treatment as prevention and its impact on the HIV epidemic	Context
D_3611	Zakumumpa H., Bennett S.,Ssengooba F.	2019	Leveraging the lessons learned from financing HIV programs to advance the universal health coverage (UHC) agenda in the East African Community	Context
D_3612	Zamawe C. O. F., Nakamura K., Shibanuma A.,Jimba M.	2016	The effectiveness of a nationwide universal coverage campaign of insecticide-treated bed nets on childhood malaria in Malawi	Context
D_3615	Zegers De Beyl C., Koenker H., Acosta A., Onyefunafoa E. O., Adegbe E., McCartney-Melstad A., Selby R. A.,Kilian A.	2016	Multi-country comparison of delivery strategies for mass campaigns to achieve universal coverage with insecticide-treated nets: What works best?	Context
D_3626	Zhou G. F., Yewhalaw D., Lo E., Zhong D. B., Wang X. M., Degefa T., Zemene E., Lee M. C., Kebede E., Tushune K.,Yan G. Y.	2016	Analysis of asymptomatic and clinical malaria in urban and suburban settings of southwestern Ethiopia in the context of sustaining malaria control and approaching elimination	Context
D_3629	Zöllner C., De Allegri M., Louis V. R., Yé M., Sié A., Tiendrebéogo J., Jahn A.,Müller O.	2015	Insecticide-treated mosquito nets in rural Burkina Faso: assessment of coverage and equity in the wake of a universal distribution campaign	Context
D_3630	Zon H., Pavlova M.,Groot W.	2021	Factors associated with access to	Context

Appendix 2. Excluded reports with reason for exclusion

			healthcare in Burkina Faso: evidence from a national household survey	
D_3633	The Improving Health in Slums Collaborative	2021	Inequity of healthcare access and use and catastrophic health spending in slum communities: A retrospective, cross-sectional survey in four countries	Context
D_3639	GBD 2016 SDG Collaborators	2017	Measuring progress and projecting attainment on the basis of past trends of the health-related Sustainable Development Goals in 188 countries: an analysis from the Global Burden of Disease Study 2016	Concept
D_3641	GBD 2015 SDG Collaborators	2016	Measuring the health-related Sustainable Development Goals in 188 countries: a baseline analysis from the Global Burden of Disease Study 2015	Concept
