# Supplementary material for: Mapping inequalities in health service coverage in Africa: a scoping review
Source: BMJ Open. 2024 Nov 24;14(11):e082918. doi: 10.1136/bmjopen-2023-082918 (PMC11590813; doi:10.1136/bmjopen-2023-082918)
Supplement: online supplemental file 3 [file bmjopen-14-11-s003.pdf]

0. IDENTIFICATION	1. BIBLIOGRAPHIC SOURCE				2. STUDY CHARACTERISTICS					3. INEQUITIES IN HEALTH	
	**Title**	**Year**	**First author**	**UHL**	**Study design**	**Target units**	**Sample coverage**	**African countries or regions involved**	**Types of funding**	**Place of residence**	**Name (acronym)**
D_0062	Reproductive Plans And Utilization of Contraceptives Among Women Living With HIV	2019	Adeleye et al.	https://dx.doi.org/10.21106/ijma.277	Cross-sectional	Women living with HIV	Organizational	Nigeria	No funding	No	
D_0065	Provision of immediate postpartum contraception to women living with HIV in the Eastern Cape, South Africa; a cross-sectional analysis	2020	Adeniyi et al.	https://doi.org/10.1186/s12978-020-01049-9	Cross-sectional	Parturient women with HIV	Sub-national	South Africa	Non-commercial	Yes	Place of residence
D_0081	Moving Up the Sanitation Ladder: A Study of the Coverage and Utilization of Improved Sanitation Facilities and Associated Factors Among Households in Southern Ethiopia	2022	Afework et al.	https://doi.org/10.1177/11786302221080825	Cross-sectional	Households	Sub-national	Ethiopia	No funding	No	

0. IDENTIFICATION	1. BIBLIOGRAPHIC SOURCE				2. STUDY CHARACTERISTICS					3. INEQUITIES IN HEALTH	
	Title	Year	First author	UHL	Study design	Target units	Sample coverage	African countries or regions involved	Types of funding	Place of residence	Name (acronym)
D_0117	Maternal health care services utilisation in the context of 'Abiye' (safe motherhood) programme in Ondo State, Nigeria	2020	Ajayi et al.	https://doi.org/10.1186/s12889-020-08512-z	Cross-sectional	Women within the reproductive age (15-49 years) that gave birth to at least one child over the 5 years post-implementation of the Abiye programme	National	Nigeria	No funding	Yes	Place of residence
D_0135	Slums, women and sanitary living in South-South Nigeria	2021	Akpabio et al.	https://doi.org/10.1007/s10901-020-09802-z	Qualitative	Women	Sub-national	Nigeria	Non-commercial	No	
D_0145	Leaving no child behind: Decomposing socioeconomic inequalities in child health for india and south africa	2021	Alaba et al.	https://doi.org/10.3390/ijerph1813711 4	Cross-sectional	Children 0 to 12 months	National	South Africa	Non-commercial	Yes	Residence status

0. IDENTIFICATION	1. BIBLIOGRAPHIC SOURCE				2. STUDY CHARACTERISTICS					3. INEQUITIES IN HEALTH	
	**Title**	**Year**	**First author**	**UHL**	**Study design**	**Target units**	**Sample coverage**	**African countries or regions involved**	**Types of funding**	**Place of residence**	**Name (acronym)**
D_0159	Access to skilled attendant at birth and the coverage of the third dose of diphtheria-tetanus-pertussis vaccine across 14 West African countries - An equity analysis	2020	Alhassan et al.	https://doi.org/10.1186/s12939-020-01204-5	Cross-sectional	Women and children	National	Benin, Burkina Faso, Cote d' Ivoire, The Gambia, Ghana, Guinea, Guinea Bissau, Liberia, Mali, Niger, Nigeria, Senegal, Sierra Leone and [illegible]	No funding	Yes	Residence status, region of region
D_0166	Trends and correlates of maternal, newborn and child health services utilization in primary healthcare facilities: An explorative ecological study using DHIMSII data from one district in the Volta region of [illegible]	2020	Alhassan et al.	https://doi.org/10.1186/s12884-020-03195-1	Cross-sectional	Health facilities	Sub-national	Ghana	No funding	Yes	Residence

0. IDENTIFICATION	1. BIBLIOGRAPHIC SOURCE				2. STUDY CHARACTERISTICS					3. INEQUITIES IN HEALTH	
	**Title**	**Year**	**First author**	**UHL**	**Study design**	**Target units**	**Sample coverage**	**African countries or regions involved**	**Types of funding**	**Place of residence**	**Name (acronym)**
D_0172	Determinants of equity in utilization of maternal health services in Butajira, Southern Ethiopia	2012	Aliy & Mariam	https://www.ajol.info/index.php/ejhd/article/view/116114	Cross-sectional	Households with pregnant mothers	Sub-national	Ethiopia	Unclear	Yes	Residence
D_0195	Determinants of insecticide treated nets use among youth corp members in Edo State, Nigeria	2011	Amoran O. E. et al	http://www.biomedcentral.com/1471-2458/11/728	Cross-sectional	University leavers serving compulsory national service (youth corpers)	Sub-national	Nigeria	Unclear	Yes	Location of Domicile

0. IDENTIFICATION	1. BIBLIOGRAPHIC SOURCE					2. STUDY CHARACTERISTICS						3. INEQUITIES IN HEALTH	
	Title	Year	First author	UHL		Study design	Target units	Sample coverage	African countries or regions involved	Types of funding		Place of residence	Name (acronym)
D_0203	Towards achievement of Sustainable Development Goal 3: multilevel analyses of demographic and health survey data on health insurance coverage and maternal healthcare utilisation in sub-Saharan Africa	2022	Amu et al.	https://doi.org/10.1093/inthealth/ihac017		Cross-sectional	Women aged 15-49	National	28; Angola, Burkina Faso, Burundi, Benin, DRC, Congo, Cote d'Ivoire, Cameroon, Ethiopia, Gabon, Ghana, Gambia, Kenya, Comoros, Liberia, Lesotho, Mali, Malawi, Nigeria, Namibia, Sierra Leone, Senegal, Chad, Togo, Uganda, Zambia,	Unclear		Yes	Residence, Subregions

0. IDENTIFICATION	1. BIBLIOGRAPHIC SOURCE					2. STUDY CHARACTERISTICS						3. INEQUITIES IN HEALTH	
	Title	Year	First author	UHL		Study design	Target units	Sample coverage	African countries or regions involved	Types of funding		Place of residence	Name (acronym)
D_0215	Equity and access to maternal and child health services in Ghana a cross-sectional study	2021	Anarwat et al.	https://doi.org/10.1186/s12913-021-06872-9		Cross-sectional	Women of reproductive age 15–49 years	National	Ghana	Non-commercial		Yes	Residence
D_0219	A subnational profiling analysis reveals regional differences as the main predictor of ITN ownership and use in Nigeria	2019	Andrada et al.	https://doi.org/10.1186/s12936-019-2816-9		Cross-sectional	General population in Nigera	National	Nigeria	Non-commercial		Yes	Residence, Region
D_0264	Inequality trends in maternal health services for young Ghanaian women with childbirth history between 2003 and 2014	2017	Asamoah & Agardh	https://doi.org/10.1136/bmjopen-2016-011663		Cross-sectional	Young women aged 15–24 years with at least one previous birth experience in the past 5 years prior to the surveys.	National	Ghana	Unclear		Yes	Residence

0. IDENTIFICATION	1. BIBLIOGRAPHIC SOURCE				2. STUDY CHARACTERISTICS					3. INEQUITIES IN HEALTH	
	**Title**	**Year**	**First author**	**UHL**	**Study design**	**Target units**	**Sample coverage**	**African countries or regions involved**	**Types of funding**	**Place of residence**	**Name (acronym)**
D_0290	Leaving no one behind: Lessons from implementation of policies for universal HIV treatment to universal health coverage	2020	Assefa et al.	https://doi.org/10.1186/s12992-020-00549-4	Mixted methods design	General population	National	South-east Africa, West-central Africa, Middle East and North Africa	No funding	Yes	Region
D_0297	Inequalities in child immunization coverage in Ghana: evidence from a decomposition analysis	2018	Asuman et al.	https://doi.org/10.1186/s13561-018-0193-7	Cross-sectional	Children between 12 and 59 months	National	Ghana	No funding	Yes	Location
D_0313	A reassessment of global antenatal care coverage for improving maternal health using sub-Saharan Africa as a case study	2018	Ataguba	https://dx.doi.org/10.1371/journal.pone.0204822	Cross-sectional	Women aged 15-49	National	Uganda	Non-commercial	Yes	Location of household

0. IDENTIFICATION	1. BIBLIOGRAPHIC SOURCE				2. STUDY CHARACTERISTICS					3. INEQUITIES IN HEALTH	
	**Title**	**Year**	**First author**	**UHL**	**Study design**	**Target units**	**Sample coverage**	**African countries or regions involved**	**Types of funding**	**Place of residence**	**Name (acronym)**
D_0315	Socio-economic inequality in maternal health care utilization in Sub-Saharan Africa: Evidence from Togo	2021	Atake	http://dx.doi.org/10.1002/hpm.3083	Cross-sectional	Women aged 15-49	National	Togo	Unclear	Yes	Area of residence
D_0336	Poor prenatal service utilization and pregnancy outcome in a tertiary health facility in southwest Nigeria	2020	Awoleke & Olofinbiyi	https://doi.org/10.11604/pamj.2020.35.28.20426	Cross-sectional	Consecutive pregnant mothers who had their deliveries at the Teaching Hospital	Organizational	Nigeria	Unclear	No	
D_0361	Correlates of intra-household ITN use in Liberia: A multilevel analysis of household survey data	2016	Babalola et al.	http://dx.doi.org/10.1371/journal.pone.0158331	Cross-sectional	Households with at least one child under the age of five years	Sub-national	Liberia	Non-commercial	Yes	County of residence

0. IDENTIFICATION	1. BIBLIOGRAPHIC SOURCE					2. STUDY CHARACTERISTICS						3. INEQUITIES IN HEALTH	
	Title	Year	First author	UHL		Study design	Target units	Sample coverage	African countries or regions involved	Types of funding		Place of residence	Name (acronym)
D_0369	Prevalence and determinants of maternal healthcare utilisation among young women in sub-Saharan Africa: cross-sectional analyses of demographic and health survey data	2022	Bain et al.	https://doi.org/10.1186/s12889-022-13037-8		Cross-sectional	Women aged 15-24	National	28; Angola, Burkina Faso, Burundi, Benin, DRC, Congo, Cote d'Ivoire, Cameroon, Ethiopia, Gabon, Ghana, Gambia, Kenya, Comoros, Liberia, Lesotho, Mali, Malawi, Nigeria, Namibia, Sierra Leone, Senegal, Chad, Togo, Uganda, Zambia,	Unclear		Yes	Residence

0. IDENTIFICATION	1. BIBLIOGRAPHIC SOURCE					2. STUDY CHARACTERISTICS						3. INEQUITIES IN HEALTH	
	Title	Year	First author	UHL		Study design	Target units	Sample coverage	African countries or regions involved	Types of funding		Place of residence	Name (acronym)
D_0423	Factors influencing timing and frequency of antenatal care in Uganda	2011	Bbaale	https://www.ncbi.nlm.nih.gov/pmc/articles/PMC3562883/pdf/AMJ-04-431.pdf		Cross-sectional	Women aged 15-49	National	Uganda	No funding		Yes	Residence, Region
D_0424	Maternal education and childbirth care in Uganda	2011	Bbaale & Guloba	https://www.ncbi.nlm.nih.gov/pmc/articles/PMC3562941/pdf/AMJ-04-389.pdf		Cross-sectional	Women aged 15-49	National	Uganda	No funding		Yes	Residence, Region

0. IDENTIFICATION	1. BIBLIOGRAPHIC SOURCE				2. STUDY CHARACTERISTICS					3. INEQUITIES IN HEALTH	
	Title	Year	First author	UHL	Study design	Target units	Sample coverage	African countries or regions involved	Types of funding	Place of residence	Name (acronym)
D_0432	A quantitative analysis of food insecurity and other barriers associated with ART nonadherence among women in rural communities of Eswatini	2021	Becker et al.	https://doi.org/10.1371/journal.pone.0256277	Cross-sectional	Women living with HIV in rural communities	National	Eswatini	Non-commercial	No	
D_0453	Two decades of antenatal and delivery care in Uganda: a cross-sectional study using Demographic and Health Surveys	2018	Benova et al.	https://doi.org/10.1186/s12913-018-3546-3	Cross-sectional	Women aged 15-49	National	Uganda	Non-commercial	Yes	Residence, Geographic zone
D_0493	Utilization of sexual and reproductive health services in ethiopia - Does it affect sexual activity among high school students?	2015	Bilal et al.	https://doi.org/10.1016/j.srhc.2014.09.009	Cross-sectional	Students from grade 9 up to grade 12 who were attending secondary and preparatory schools	Sub-national	Ethiopia	Unclear	No	

0. IDENTIFICATION	1. BIBLIOGRAPHIC SOURCE				2. STUDY CHARACTERISTICS					3. INEQUITIES IN HEALTH	
	Title	Year	First author	UHL	Study design	Target units	Sample coverage	African countries or regions involved	Types of funding	Place of residence	Name (acronym)
D_0497	Addressing the huge poor–rich gap of inequalities in accessing safe childbirth care: A first step to achieving universal maternal health coverage in Tanzania	2021	Bintabara	https://doi.org/10.1371/journal.pone.0246995	Cross-sectional	Women aged 15-49	National	Tanzania	No funding	Yes	Residence
D_0500	Twelve-year persistence of inequalities in antenatal care utilisation among women in Tanzania: A decomposition analysis of population-based cross-sectional surveys	2021	Bintabara & Basinda	https://doi.org/10.1136/bmjopen-2020-040450	Cross-sectional	Women aged 15-49	National	Tanzania	No funding	Yes	Residence
D_0520	Socio-demographic and economic inequalities in modern contraception in 11 low- And middle-income countries: An analysis of the PMA2020 surveys	2020	Blumenberg et al.	https://doi.org/10.1186/s12978-020-00931-w	Cross-sectional	Women in reproductive age (15–49 years) that were sexually active.	Multiple levels	9; Burkina Faso, Cote d'Ivoire, Congo DR, Ethiopia, Ghana, Niger, Nigeria, Kenya, Uganda	Non-commercial	No	

0. IDENTIFICATION	1. BIBLIOGRAPHIC SOURCE				2. STUDY CHARACTERISTICS					3. INEQUITIES IN HEALTH	
	**Title**	**Year**	**First author**	**UHL**	**Study design**	**Target units**	**Sample coverage**	**African countries or regions involved**	**Types of funding**	**Place of residence**	**Name (acronym)**
D_0550	A decomposition analysis of change in skilled birth attendants, 2003 to 2008, Ghana demographic and health surveys	2014	Bosomprah et al.	https://doi.org/10.1186/s12884-014-0415-x	Cross-sectional	live births in the five years preceding the survey,	National	Ghana	Non-commercial	No	
D_0593	Slow and Steady can Still Win the Race': Childhood Vaccination Experience of Migrant Ebira Women Within the Health System in Ekiti State, Nigeria	2021	Olakanmi-Falade & Awoleke	https://www.ojhas.org/issue79/2021-3-3.html	Mixted methods design	Migrant Ebira mothers who had children under 2 years, who had lived in the community for at least 12 months before the time of the [illegible]	Sub-national	Nigeria	Unclear	No	
D_0612	Antiretroviral therapy in Walvis Bay, Namibia	2016	Callaghan	https://hdl.handle.net/1807/70825	Cross-sectional	Kuisebmond Population	Organizational	Namibia	Unclear	No	

0. IDENTIFICATION	1. BIBLIOGRAPHIC SOURCE				2. STUDY CHARACTERISTICS					3. INEQUITIES IN HEALTH	
	**Title**	**Year**	**First author**	**UHL**	**Study design**	**Target units**	**Sample coverage**	**African countries or regions involved**	**Types of funding**	**Place of residence**	**Name (acronym)**
D_0621	Family planning, antenatal and delivery care: Cross-sectional survey evidence on levels of coverage and inequalities by public and private sector in 57 low- and middle-income countries	2016	Campbell et al.	https://doi.org/10.1111/tmi.12681	Cross-sectional	Women ever-married or all women of reproductive age	National	30-Benin, Burkina Faso, Burundi, Cameroon, Chad, Congo, DR Congo, Ethiopia, Gabon, Ghana, Guinea, Kenya, Lesotho, Liberia, Madagascar, Malawi, Mali, Mozambique, Namibia, Niger, Nigeria, Rwanda, Sao Tome & Principe, Senegal, Sierra Leona, Swaziland, Tanzania, Uganda, Zambia	Unclear	No	

0. IDENTIFICATION	1. BIBLIOGRAPHIC SOURCE				2. STUDY CHARACTERISTICS					3. INEQUITIES IN HEALTH	
	Title	Year	First author	UHL	Study design	Target units	Sample coverage	African countries or regions involved	Types of funding	Place of residence	Name (acronym)
D_0648	Use of family planning and child health services in the private sector: An equity analysis of 12 DHS surveys	2018	Chakraborty & Sprockett	https://doi.org/10.1186/s12939-018-0763-7	Cross-sectional	Women of reproductive age (15–49 years),	National	8-DRC, Ghana, Kenya, Liberia, Mali, Nigeria, Senegal, Zambia	Non-commercial	Yes	Residence
D_0697	Meeting demand for family planning within a generation: prospects and implications at country level	2015	Choi et al.	https://dx.doi.org/10.3402/gha.v8.29734	Cross-sectional	Women between 15 and 49 years of age who are married or in a union	National	Ethiopia, Burkina Faso, Nigeria	Unclear	Yes	Residence
D_0716	A Longitudinal Analysis of Mosquito Net Ownership and Use in an Indigenous Batwa Population after a Targeted Distribution	2016	Clark et al.	https://doi.org/10.1371/journal.pone.0154808	Longitudinal	Indigenous Batwa population	Sub-national	Uganda	Non-commercial	No	

0. IDENTIFICATION	1. BIBLIOGRAPHIC SOURCE				2. STUDY CHARACTERISTICS					3. INEQUITIES IN HEALTH	
	**Title**	**Year**	**First author**	**UHL**	**Study design**	**Target units**	**Sample coverage**	**African countries or regions involved**	**Types of funding**	**Place of residence**	**Name (acronym)**
D_0746	Malaria prevalence and long-lasting insecticidal net use in rural western Uganda: results of a cross-sectional survey conducted in an area of highly variable malaria transmission intensity	2021	Cote et al.	https://doi.org/10.1186/s12936-021-03835-7	Cross-sectional	Chilren households in Bugoye sub-county	Sub-national	Uganda	Non-commercial	Yes	Elevation quartile
D_0761	Women's Sexual Empowerment and Contraceptive Use in Ghana	2012	Crissman et al.	https://doi.org/10.1111/j.1728-4465.2012.00318.x	Cross-sectional	Nonpregnant married and partnered women not desiring to conceive in the next three months.	National	Ghana	Unclear	Yes	Setting; Region
D_0771	Socio-economic and demographic factors associated with reproductive and child health preventive care in Mozambique: a cross-sectional study	2020	Daca et al.	https://doi.org/10.1186/s12939-020-01303-3	Cross-sectional	Women aged 15 to 49 years. Children aged 12 to 59 months	National	Mozambique	Non-commercial	Yes	Residence, Region

0. IDENTIFICATION	1. BIBLIOGRAPHIC SOURCE				2. STUDY CHARACTERISTICS					3. INEQUITIES IN HEALTH	
	**Title**	**Year**	**First author**	**UHL**	**Study design**	**Target units**	**Sample coverage**	**African countries or regions involved**	**Types of funding**	**Place of residence**	**Name (acronym)**
D_0800	Assessing the contextual effect of community in the utilization of postnatal care services in Ghana	2021	Dankwah et al.	https://doi.org/10.1186/s12913-020-06028-1	Cross-sectional	Women aged 15–49 years	National	Ghana	Non-commercial	Yes	Regional zone
D_0812	Is South Africa closing the health gaps between districts? Monitoring progress towards universal health service coverage with routine facility data	2021	Day et al.	https://doi.org/10.1186/s12913-021-06171-3	Cross-sectional	Districts in South Africa	Sub-national	South Africa	Non-commercial	No	
D_0828	Neglected tropical diseases as a 'litmus test' for universal health coverage? understanding who is left behind and why in mass drug administration: Lessons from four country contexts	2019	Dean et al.	https://doi.org/10.1371/journal.pntd.0007847	Qualitative	Communities and Health implementers	Sub-national	Ghana, Nigeria, Liberia, Cameroon	Non-commercial	No	

0. IDENTIFICATION	1. BIBLIOGRAPHIC SOURCE					2. STUDY CHARACTERISTICS						3. INEQUITIES IN HEALTH	
	**Title**	**Year**	**First author**	**UHL**		**Study design**	**Target units**	**Sample coverage**	**African countries or regions involved**	**Types of funding**		**Place of residence**	**Name (acronym)**
D_0830	Preventive Health Service Coverage Among Infants and Children at Six Maternal-Child Health Clinics in Western Kenya: A Cross-Sectional Assessment	2022	Deathe et al.	https://doi.org/10.1007/s10995-021-03271-8		Cross-sectional	Children under five	Sub-national	Kenya	Non-commercial		No	
D_0838	Distance, difference in altitude and socioeconomic determinants of utilisation of maternal and child health services in Ethiopia: A geographic and multilevel modelling analysis	2021	Defar et al.	https://doi.org/10.1136/bmjopen-2020-042095		Cross-sectional	Women aged 13–49 with a live birth within the last 12 months and children aged 12–23 months	Sub-national	Ethiopia	Non-commercial		Yes	Region
D_0839	Geographic differences in maternal and child health care utilization in four Ethiopian regions; A cross-sectional study	2019	Defar et al.	https://doi.org/10.1186/s12939-019-1079-y		Cross-sectional	Women aged 13–49 with a live birth within the last 12 months and children aged 12–23 months	Sub-national	Ethiopia	Non-commercial		No	

0. IDENTIFICATION	1. BIBLIOGRAPHIC SOURCE				2. STUDY CHARACTERISTICS					3. INEQUITIES IN HEALTH	
	**Title**	**Year**	**First author**	**UHL**	**Study design**	**Target units**	**Sample coverage**	**African countries or regions involved**	**Types of funding**	**Place of residence**	**Name (acronym)**
D_0863	Ownership and use of insecticide-treated nets in Oromia and Amhara Regional States of Ethiopia twoyears after a nationwide campaign	2011	Deressa et al.	https://doi.org/10.1111/j.1365-3156.2011.02875.x	Cross-sectional	Households	Sub-national	Ethiopia	Non-commercial	Yes	Area of residence; Region
D_0867	Effectiveness of post-campaign, door-to-door, hang-up, and communication interventions to increase long-lasting, insecticidal bed net utilization in Togo (2011-2012): A cluster randomized, control trial	2014	Desrochers et al.	https://doi.org/10.1186/1475-2875-13-260	Randomized controlled trial	Communities	Sub-national	Togo	Non-commercial	No	
D_0869	Immediate postnatal care following childbirth in Ugandan health facilities: An analysis of Demographic and Health Surveys between 2001 and 2016	2021	Dey et al.	https://doi.org/10.1136/bmjgh-2020-004230	Cross-sectional	Women 15–49 years	National	Uganda	Non-commercial	Yes	Residence

0. IDENTIFICATION	1. BIBLIOGRAPHIC SOURCE				2. STUDY CHARACTERISTICS					3. INEQUITIES IN HEALTH	
	**Title**	**Year**	**First author**	**UHL**	**Study design**	**Target units**	**Sample coverage**	**African countries or regions involved**	**Types of funding**	**Place of residence**	**Name (acronym)**
D_0870	Inequalities in non-communicable diseases and effective responses	2013	Di Cesare et al.	https://doi.org/10.1016/s0140-6736(12)61851-0	Cross-sectional	General population	National	Malawi	Non-commercial	No	
D_0871	Insecticide-treated nets ownership and utilization among under-five children following the 2010 mass distribution in Burkina Faso	2014	Diabate et al.	https://dx.doi.org/10.1186/1475-2875-13-353	Cross-sectional	Households	Sub-national	Burkina Faso	Unclear	Yes	Place of residence

0. IDENTIFICATION	1. BIBLIOGRAPHIC SOURCE				2. STUDY CHARACTERISTICS					3. INEQUITIES IN HEALTH	
	Title	Year	First author	UHL	Study design	Target units	Sample coverage	African countries or regions involved	Types of funding	Place of residence	Name (acronym)
D_0892	Impact of mining projects on water and sanitation infrastructures and associated child health outcomes: a multi-country analysis of Demographic and Health Surveys (DHS) in sub-Saharan Africa	2021	Dietler et al.	https://doi.org/10.1186/s12992-021-00723-2	Cross-sectional	Households	National	34; Angola, Burkina Faso, Benin, Burundi, Cameroon, Central African Republic, Chad, Comoros, Côte d'Ivoire, Democratic Republic of the Congo, Eswatini, Ethiopia, Gambia,Ghana, Guinea, Kenya, Liberia, Lesotho, Madagascar, Malawi, Mali, Mozambique, Namibia, Niger, Nigeria, Rwanda, [illegible]	Non-commercial	No	

0. IDENTIFICATION	1. BIBLIOGRAPHIC SOURCE				2. STUDY CHARACTERISTICS					3. INEQUITIES IN HEALTH	
	Title	Year	First author	UHL	Study design	Target units	Sample coverage	African countries or regions involved	Types of funding	Place of residence	Name (acronym)
D_0970	Predictors of insecticidal net use among internally displaced persons aged 6-59 months in Abuja, Nigeria	2018	Ejembi et al.	http://dx.doi.org/10.11604/pamj.2018.29.136.13322	Cross-sectional	Children aged 6-59 months at internal displaced persons camps	Sub-national	Nigeria	Unclear	No	
D_0979	Changes in Inequality in Use of Maternal Health Care Services: Evidence from Skilled Birth Attendance in Mauritania for the Period 2007-2015	2022	Taleb El Hassen et al.	https://doi.org/10.3390/ijerph19063566	Cross-sectional	Women aged 15-49 years; households, women of childbearing age, children under five, men aged 15 to 49	National	Mauritania	Non-commercial	Yes	1) Place of residence; 2) 13 regions of the country

0. IDENTIFICATION	1. BIBLIOGRAPHIC SOURCE				2. STUDY CHARACTERISTICS					3. INEQUITIES IN HEALTH	
	Title	Year	First author	UHL	Study design	Target units	Sample coverage	African countries or regions involved	Types of funding	Place of residence	Name (acronym)
D_0999	Trend in the use of modern contraception in sub-Saharan Africa: Does women's education matter?	2014	Emina et al.	http://dx.doi.org/10.1016/j.contraception.2014.02.001	Cross-sectional	Women	National	27; Angola, Benin, Botswana,Burkina Faso, Burundi, Cameroon, Cape Verde, Central African Republic, Chad, Comoros, Congo, DRC, Cote d'Ivoire, Djibouti, Equatorial Guinea, Ethiopia, Gabon, Gambia, Ghana, Guinea, Guinea Bissau, Kenya, Lesotho, Liberia, Madagascar, Malawi, Mali	Unclear	No	

0. IDENTIFICATION	1. BIBLIOGRAPHIC SOURCE				2. STUDY CHARACTERISTICS					3. INEQUITIES IN HEALTH	
	**Title**	**Year**	**First author**	**UHL**	**Study design**	**Target units**	**Sample coverage**	**African countries or regions involved**	**Types of funding**	**Place of residence**	**Name (acronym)**
D_1013	Utilization, Predictors and Gaps in the Continuum of Care for Maternal and Newborn Health in Ghana	2021	Enos et al.	https://dx.doi.org/10.21106/ijma.425	Cross-sectional	Women aged 15-49 years	Sub-national	Ghana	No funding	Yes	Residence
D_1036	ART use and associated factors among HIV positive caregivers of orphans and vulnerable children in Tanzania	2020	Exavery et al.	https://doi.org/10.1186/s12889-020-09361-6	Cross-sectional	Caregivers of orphans and vulnerable children living with HIV	Sub-national	Tanzania	No funding	Yes	Residence
D_1063	Trends and causes of socioeconomic inequalities in maternal healthcare in Ghana, 2003-2014	2019	Fenny et al.	https://doi.org/10.1108/ijse-03-2018-0148	Cross-sectional	Women aged 15–49 years	National	Ghana	Unclear	Yes	Residence

0. IDENTIFICATION	1. BIBLIOGRAPHIC SOURCE					2. STUDY CHARACTERISTICS						3. INEQUITIES IN HEALTH	
	Title	Year	First author	UHL		Study design	Target units	Sample coverage	African countries or regions involved	Types of funding		Place of residence	Name (acronym)
D_1088	Determination of the predictive factors of long-lasting insecticide-treated net ownership and utilisation in the Bamenda Health District of Cameroon	2017	Fokam et al.	https://doi.org/10.1186/s12889-017-4155-5		Cross-sectional	Households	Sub-national	Cameroon	Non-commercial		No	
D_1103	The free caesareans policy in low-income settings: An interrupted time series analysis in Mali (2003-2012)	2014	Fournier et al.	https://doi.org/10.1371/journal.pone.0105130		Interrupted time series	Women	Sub-national	Mali	Non-commercial		Yes	Residence

0. IDENTIFICATION	1. BIBLIOGRAPHIC SOURCE					2. STUDY CHARACTERISTICS						3. INEQUITIES IN HEALTH	
	Title	Year	First author	UHL		Study design	Target units	Sample coverage	African countries or regions involved	Types of funding		Place of residence	Name (acronym)
D_1108	DO BETTER INSTITUTIONS BROADEN ACCESS TO SANITATION IN SUB-SAHARA AFRICA?	2021	Francois et al.	https://doi.org/10.1111/coep.12512		Cross-sectional	General population in countries	National	44; Angola, Benin, Botswana,Burkina Faso, Burundi, Cameroon, Cape Verde, Central African Republic, Chad, Comoros, Congo, DRC, Cote d'Ivoire, Djibouti, Equatorial Guinea, Ethiopia, Eritrea,Eswatini, Gabon, Gambia, Ghana, Guinea, Guinea Bissau, Kenya, Lesotho, Liberia, Madagasc[illegible]	Non-commercial		Yes	Residence

0. IDENTIFICATION	1. BIBLIOGRAPHIC SOURCE				2. STUDY CHARACTERISTICS					3. INEQUITIES IN HEALTH	
	Title	Year	First author	UHL	Study design	Target units	Sample coverage	African countries or regions involved	Types of funding	Place of residence	Name (acronym)
D_1144	Ethnic disparities in utilisation of maternal health care services in Ghana: evidence from the 2007 Ghana Maternal Health Survey	2016	Ganle	https://doi.org/10.1080/13557858.2015.1015499	Cross-sectional	Women aged 15-49	National	Ghana	Non-commercial	No	
D_1145	Risky sexual behaviour and contraceptive use in contexts of displacement: Insights from a cross-sectional survey of female adolescent refugees in Ghana	2019	Ganle et al.	https://doi.org/10.1186/s12939-019-1031-1	Cross-sectional	Female adolescent refugees aged 14–19 years.	Sub-national	Ghana	No funding	No	
D_1147	Understanding how distance to facility and quality of care affect maternal health service utilization in Kenya and Haiti: A comparative geographic information system study	2019	Gao & Kelley	https://doi.org/10.4081/gh.2019.690	Cross-sectional	Women aged 15-49	National	Kenya	No funding	No	

0. IDENTIFICATION	1. BIBLIOGRAPHIC SOURCE					2. STUDY CHARACTERISTICS						3. INEQUITIES IN HEALTH	
	Title	Year	First author	UHL		Study design	Target units	Sample coverage	African countries or regions involved	Types of funding		Place of residence	Name (acronym)
D_1161	Gender differences in the use of insecticide-treated nets after a universal free distribution campaign in Kano State, Nigeria: Post-campaign survey results	2013	Garley et al.	https://doi.org/10.1186/1475-2875-12-119		Cross-sectional	Households in areas which were covered by two independent campaign waves.	Sub-national	Nigeria	Unclear		Yes	Residence
D_1190	Demand satisfied by modern contraceptive among married women of reproductive age in Kenya	2021	Gichangi et al.	https://doi.org/10.1371/journal.pone.0248393		Cross-sectional	Married women of reproductive age	National	Kenya	Non-commercial		Yes	Area of residence; County of residence
D_1226	LLIN Evaluation in Uganda Project (LLINEUP): Factors associated with ownership and use of long-lasting insecticidal nets in Uganda: A cross-sectional survey of 48 districts ISRCTN17516395 ISRCTN	2018	Gonahasa et al.	https://doi.org/10.1186/s12936-018-2571-3		Cross-sectional	Households	National	Uganda	Non-commercial		Yes	Region

0. IDENTIFICATION	1. BIBLIOGRAPHIC SOURCE				2. STUDY CHARACTERISTICS					3. INEQUITIES IN HEALTH	
	**Title**	**Year**	**First author**	**UHL**	**Study design**	**Target units**	**Sample coverage**	**African countries or regions involved**	**Types of funding**	**Place of residence**	**Name (acronym)**
D_1287	ART adherence and viral suppression are high among most non-pregnant individuals with early-stage, asymptomatic HIV infection: an observational study from Uganda and South Africa	2019	Haberer et al.	https://doi.org/10.1002/jia2.25232	Longitudinal	Men and non-pregnant women with early-stage HIV infection	Sub-national	2; Uganda, South Africa	Non-commercial	No	
D_1296	Low immunization coverage in Wonago district, southern Ethiopia: A community-based cross-sectional study	2019	Hailu et al.	https://doi.org/10.1371/journal.pone.0220144	Cross-sectional	Children aged 6–36 months	Sub-national	Ethiopia	Non-commercial	No	

0. IDENTIFICATION	1. BIBLIOGRAPHIC SOURCE				2. STUDY CHARACTERISTICS					3. INEQUITIES IN HEALTH	
	Title	Year	First author	UHL	Study design	Target units	Sample coverage	African countries or regions involved	Types of funding	Place of residence	Name (acronym)
D_1393	Towards universal health coverage: The role of within-country wealth-related inequality in 28 countries in sub-Saharan Africa	2011	Hosseinpoor et al.	https://doi.org/10.2471/BLT.11.087536	Cross-sectional	Women and children	National	28; Benin, Burkina Faso, Cameroon, Congo, DRC, Cote d'Ivoire, Djibouti, Equatorial Guinea, Ethiopia, Gabon, Ghana, Guinea, Kenya, Lesotho, Liberia, Madagascar, Malawi, Mali, Mozambique, Namibia, Niger, Nigeria, Rwanda, Senegal, Sierra Leone, Swaziland, Tanzania, Uganda, Zambia	Unclear	No	

0. IDENTIFICATION	1. BIBLIOGRAPHIC SOURCE					2. STUDY CHARACTERISTICS						3. INEQUITIES IN HEALTH	
	**Title**	**Year**	**First author**	**UHL**		**Study design**	**Target units**	**Sample coverage**	**African countries or regions involved**	**Types of funding**		**Place of residence**	**Name (acronym)**
D_1396	Patterns and trends of contraceptive use among sexually active adolescents in Burkina Faso, Ethiopia, and Nigeria: evidence from cross-sectional studies	2015	Hounton et al.	https://doi.org/10.3402/gha.v8.29737		Cross-sectional	Sexually active adolescents (15- to 19-year age group)	National	3; Burkina Faso, Ethiopia, Nigeria	No funding		Yes	Residence
D_1419	Towards universal health coverage for reproductive health services in Ethiopia: two policy recommendations	2015	Onarheim et al.	https://doi.org/10.1186/s12939-015-0218-3		Cross-sectional	Women aged 15-49 years, Households	National	Ethiopia	Non-commercial		Yes	1) Location; 2) Administrative region (nine regions and two cities)

0. IDENTIFICATION	1. BIBLIOGRAPHIC SOURCE				2. STUDY CHARACTERISTICS					3. INEQUITIES IN HEALTH	
	**Title**	**Year**	**First author**	**UHL**	**Study design**	**Target units**	**Sample coverage**	**African countries or regions involved**	**Types of funding**	**Place of residence**	**Name (acronym)**
D_1443	Modern Contraception: Uptake and Correlates among Women of Reproductive Age-Group in a Rural Community of Osun State, Nigeria	2020	Idowu et al.	https://doi.org/10.4314/ejhs.v30i4.8	Cross-sectional	Women between 15-49 years of age,	Sub-national	Nigeria	Unclear	No	
D_1452	Determinants of geographical inequalities for DTP3 vaccine coverage in sub-Saharan Africa	2020	Ikilezi et al.	https://doi.org/10.1016/j.vaccine.2020.03.005	Cross-sectional	Children 12–23 months of age	National		Unclear	Yes	Rural population
D_1456	Influence of women's decision-making autonomy on antenatal care utilisation and institutional delivery services in Nigeria: evidence from the Nigeria Demographic and Health Survey 2018	2022	Imo	https://doi.org/10.1186/s12884-022-04478-5	Cross-sectional	Childbearing women (15–49 years) who were ever-married/living with partners.	National	Nigeria	No funding	Yes	Place of residence; Region

0. IDENTIFICATION	1. BIBLIOGRAPHIC SOURCE					2. STUDY CHARACTERISTICS						3. INEQUITIES IN HEALTH	
	Title	Year	First author	UHL		Study design	Target units	Sample coverage	African countries or regions involved	Types of funding		Place of residence	Name (acronym)
D_1463	Individual and community-level determinants of cervical cancer screening in Zimbabwe: a multi-level analyses of a nationwide survey	2022	Isabirye et al.	https://doi.org/10.1186/s12905-022-01881-0		Cross-sectional	Women aged 15–49 years.	National	Zimbabwe	No funding		Yes	Residence
D_1504	Two decades of maternity care fee exemption policies in Ghana: have they benefited the poor?	2016	Johnson et al.	https://doi.org/10.1093/heapol/czv017		Cross-sectional	Women who gave birth 5 years preceding the survey; 1993 it was 3 years	National	Ghana	Unclear		Yes	Place of residence; Region

0. IDENTIFICATION	1. BIBLIOGRAPHIC SOURCE					2. STUDY CHARACTERISTICS						3. INEQUITIES IN HEALTH	
	**Title**	**Year**	**First author**	**UHL**		**Study design**	**Target units**	**Sample coverage**	**African countries or regions involved**	**Types of funding**		**Place of residence**	**Name (acronym)**
D_1516	Socioeconomic inequalities in access to skilled birth attendance among urban and rural women in low-income and middle-income countries	2018	Joseph et al.	https://doi.org/10.1136/bmjgh-2018-000898		Cross-sectional	Participants were women in reproductive age from 15 to 49 years old with information on birth attendance in the 3 (DHS) or 2 years (MICS) preceding the survey.	National	Benin, Burkina Faso, Burundi, Central African Republic, Chad, Comoros, DRC, Ethiopia, Ghana, Gabon, Gambia, Guinea-Bissau, Guinea, Kenya, Lesotho, Liberia, Malawi, Mali, Mauritania, Mozambique, Namibia, Niger, Nigeria, Rwanda, Sao Tome & Principe, Senegal, Sierra	Non-commercial		Yes	Residence

0. IDENTIFICATION	1. BIBLIOGRAPHIC SOURCE					2. STUDY CHARACTERISTICS						3. INEQUITIES IN HEALTH	
	Title	Year	First author	UHL		Study design	Target units	Sample coverage	African countries or regions involved	Types of funding		Place of residence	Name (acronym)
D_1542	Investigating the disparities in cervical cancer screening among Namibian women	2015	Kangmennaang et al.	https://doi.org/10.1016/j.ygyno.2015.05.036		Cross-sectional	Women who reported hearing about cervical cancer.	National	Namibia	Unclear		Yes	Residence
D_1548	Contraceptive use and needs among adolescent women aged 15-19: Regional and global estimates and projections from 1990 to 2030 from a Bayesian hierarchical modelling study	2021	Kantorová et al.	https://doi.org/10.1371/journal.pone.0247479		Longitudinal	Adolescents aged 15–19 years from 754 nationally; Women aged 15–49 years	Multiple levels	Sub-Saharan Africa and Northern Africa	Non-commercial		No	
D_1566	Changes in equity of maternal, newborn, and child health care practices in 115 districts of rural Ethiopia: Implications for the health extension program	2015	Karim et al.	https://doi.org/10.1186/s12884-015-0668-z		Cross-sectional	Women age 15 to 49 years	Sub-national	Ethiopia	Unclear		Yes	Distance to health facility

0. IDENTIFICATION	1. BIBLIOGRAPHIC SOURCE				2. STUDY CHARACTERISTICS					3. INEQUITIES IN HEALTH	
	**Title**	**Year**	**First author**	**UHL**	**Study design**	**Target units**	**Sample coverage**	**African countries or regions involved**	**Types of funding**	**Place of residence**	**Name (acronym)**
D_1571	Contraceptive dynamics during COVID-19 in sub-Saharan Africa: Longitudinal evidence from Burkina Faso and Kenya	2021	Karp et al.	https://doi.org/10.1136/bmjsrh-2020-200944	Pre-Post	Women at risk of unintended pregnancy (15 -49 years)	Multiple levels	Kenya and Burkina Faso	Non-commercial	Yes	1) Residence; 2) Countries
D_1583	Long-lasting insecticidal net source, ownership and use in the context of universal coverage: A household survey in eastern Rwanda	2015	Kateera et al.	https://doi.org/10.1186/s12936-015-0915-9	Cross-sectional	Households	Sub-national	Rwanda	Non-commercial	No	
D_1595	A quasi-experimental evaluation of an interpersonal communication intervention to increase insecticide-treated net use among children in Zambia	2012	Keating et al.	https://doi.org/10.1186/1475-2875-11-313	Non-Randomized controlled trial	Households	Sub-national	Zambia	No funding	No	

0. IDENTIFICATION	1. BIBLIOGRAPHIC SOURCE				2. STUDY CHARACTERISTICS					3. INEQUITIES IN HEALTH	
	Title	Year	First author	UHL	Study design	Target units	Sample coverage	African countries or regions involved	Types of funding	Place of residence	Name (acronym)
D_1596	Assessment of Inequalities in Coverage of Essential Reproductive, Maternal, Newborn, Child, and Adolescent Health Interventions in Kenya	2018	Keats et al.	https://doi.org/10.1001/jamanetworkopen.2018.5152	Cross-sectional	Women of repoductive age (15-49 years) and children younger than years, with national, regional, county, and subcounty level representati	National	Kenya	Non-commercial	Yes	1) Residence; 2) region
D_1609	Charting health system reconstruction in post-war Liberia: a comparison of rural vs. remote healthcare utilization	2016	Kentoffioet al.	https://doi.org/10.1186/s12913-016-1709-7	Cross-sectional	Konobo district	National	Liberia	Non-commercial	Yes	Region

0. IDENTIFICATION	1. BIBLIOGRAPHIC SOURCE					2. STUDY CHARACTERISTICS						3. INEQUITIES IN HEALTH	
	Title	Year	First author	UHL		Study design	Target units	Sample coverage	African countries or regions involved	Types of funding		Place of residence	Name (acronym)
D_1618	A Transparent Universal Health Coverage Index with Decomposition by Socioeconomic Groups: Application in Asian and African Settings	2019	Khan et al.	https://doi.org/10.1007/s40258-019-00464-9		Cross-sectional	UHC social-economic groups	National	15 African Countries Chad and Namibia	No funding		Yes	Countries
D_1691	Effect of user preferences on ITN use: a review of literature and data	2017	Koenker & Yukich	https://doi.org/10.1186/s12936-017-1879-8		Other type of review	Household	National	Burkina Faso, Gambia, Malawi, Mali, Nigeria, Rwanda, Senegal	Non-commercial		Yes	1) Residence; Countries

0. IDENTIFICATION	1. BIBLIOGRAPHIC SOURCE				2. STUDY CHARACTERISTICS					3. INEQUITIES IN HEALTH	
	**Title**	**Year**	**First author**	**UHL**	**Study design**	**Target units**	**Sample coverage**	**African countries or regions involved**	**Types of funding**	**Place of residence**	**Name (acronym)**
D_1787	Insecticide-treated net use before and after mass distribution in a fishing community along Lake Victoria, Kenya: successes and unavoidable pitfalls	2014	Larson et al.	http://www.malariajournal.com/content/13/1/466	Pre-Post	Household representatives	National	Kenya	Non-commercial	No	
D_1822	Lifetime Prevalence of Cervical Cancer Screening in 55 Low- and Middle-Income Countries	2020	Lemp et al.	https://doi.org/10.1001/jama.2020.16244	Cross-sectional	Women aged 15 or older	National	14; Ghana, Côte d'Ivoire, Namibia, Botswana, Eswatini, Lesotho, Benin, Kenya, Zimbabwe, South Africa, Sudan, Morocco, Algeria, [illegible]	Non-commercial	Yes	Place of residence

0. IDENTIFICATION	1. BIBLIOGRAPHIC SOURCE				2. STUDY CHARACTERISTICS					3. INEQUITIES IN HEALTH	
	**Title**	**Year**	**First author**	**UHL**	**Study design**	**Target units**	**Sample coverage**	**African countries or regions involved**	**Types of funding**	**Place of residence**	**Name (acronym)**
D_1825	Financial accessibility and user fee reforms for maternal healthcare in five sub-Saharan countries: a quasi-experimental analysis	2016	Leone et al.	https://dx.doi.org/10.1136/bmjopen-2015-009692	Cross-sectional	Women	National	5; Burkina Faso, Cameroon, Ghana, Nigeria, Zambia	Non-commercial	Yes	Residence
D_1838	Antiretroviral treatment coverage in a rural district in Tanzania--a modeling study using empirical data	2015	Levira et al.	https://doi.org/10.1186/s12889-015-1460-8	Longitudinal	PLHIV in the Rufiji district of Tanzania	Sub-national	Tanzania	Unclear	No	
D_1854	The extent of universal health coverage for maternal health services in eastern uganda: A cross sectional study	2021	Lindberg et al.	https://dx.doi.org/10.1007/s10995-021-03357-3	Cross-sectional	Resident women giving birth in 2017	Sub-national	Uganda	Non-commercial	Yes	Place of residence

0. IDENTIFICATION	1. BIBLIOGRAPHIC SOURCE				2. STUDY CHARACTERISTICS					3. INEQUITIES IN HEALTH	
	**Title**	**Year**	**First author**	**UHL**	**Study design**	**Target units**	**Sample coverage**	**African countries or regions involved**	**Types of funding**	**Place of residence**	**Name (acronym)**
D_1868	Freely distributed bed-net use among Chano Mille residents, south Ethiopia: A longitudinal study	2013	Loha et al.	https://doi.org/10.1186/1475-2875-12-23	Longitudinal	Household	Sub-national	Ethiopia	Non-commercial	No	
D_1915	Determinants of hanging and use of ITNs in the context of near universal coverage in Zambia	2012	Macintyre et al.	https://doi.org/10.1093/heapol/czr042	Cross-sectional	Households (Mothers, children under 5)	Sub-national	Zambia	Non-commercial	No	

0. IDENTIFICATION	1. BIBLIOGRAPHIC SOURCE				2. STUDY CHARACTERISTICS					3. INEQUITIES IN HEALTH	
	**Title**	**Year**	**First author**	**UHL**	**Study design**	**Target units**	**Sample coverage**	**African countries or regions involved**	**Types of funding**	**Place of residence**	**Name (acronym)**
D_1919	Determinants and Consequences of Failure of Linkage to Antiretroviral Therapy at Primary Care Level in Blantyre, Malawi: A Prospective Cohort Study	2012	MacPherson et al.	https://doi.org/10.1371/journal.pone.0044794	Longitudinal	>15 years old with confirmed HIV infection	Organizational	Malawi	Non-commercial	No	
D_1921	Disability and sexual and reproductive health service utilisation in Uganda: an intersectional analysis of demographic and health surveys between 2006 and 2016	2022	Mac-Seing et al.	https://doi.org/10.1186/s12889-022-12708-w	Cross-sectional	People with disabilities (women and men, aged 18 to 49)	National	Uganda	Non-commercial	Yes	1) Place of residence; 2) Region

0. IDENTIFICATION	1. BIBLIOGRAPHIC SOURCE				2. STUDY CHARACTERISTICS					3. INEQUITIES IN HEALTH	
	Title	Year	First author	UHL	Study design	Target units	Sample coverage	African countries or regions involved	Types of funding	Place of residence	Name (acronym)
D_1931	Insight into Nigeria's progress towards the universal coverage of reproductive, maternal, newborn and child health services: a secondary data analysis	2022	Mafiana et al.	http://dx.doi.org/10.1136/bmjopen-2022-061595	Interrupted time series	Women aged 15–49, children under 5	National	Nigeria	Non-commercial	Yes	Place of residence
D_1947	Implementation of Urban Health Equity Assessment and Response Tool: a Case of Matsapha, Swaziland	2018	Makadzange et al.	https://doi.org/10.1007/s11524-018-0241-y	Case study	Community members, health workers, police, local leaders	Sub-national	Swaziland	No funding	Yes	Area
D_1970	Effects of door-to-door hang-up visits on the use of long-lasting insecticide-treated mosquito nets in the democratic republic of the congo: A cluster randomized controlled trial	2021	Mankadi and Jin	https://doi.org/10.3390/ijerph18179048	Randomized controlled trial	Household members	Sub-national	Democratic Republic of the Congo	Non-commercial	No	

0. IDENTIFICATION	1. BIBLIOGRAPHIC SOURCE				2. STUDY CHARACTERISTICS					3. INEQUITIES IN HEALTH	
	**Title**	**Year**	**First author**	**UHL**	**Study design**	**Target units**	**Sample coverage**	**African countries or regions involved**	**Types of funding**	**Place of residence**	**Name (acronym)**
D_2010	Effect of bed net colour and shape preferences on bed net usage: a secondary data analysis of the 2017 Malawi Malaria Indicator Survey	2020	Mategula et al.	https://doi.org/10.1186/s12936-020-03499-9	Cross-sectional	Households	National	Malawi	No funding	Yes	1) Residence; 2) Region
D_2015	HIV care coverage among HIV-positive adolescent girls and young women in South Africa: Results from the HERStory Study	2021	Mathews et al.	https://doi.org/10.7196/SAMJ.2021.v111i5.15351	Cross-sectional	Adolescent girls and young women (AGYW) aged 15 - 24 years	Sub-national	South Africa	Non-commercial	No	
D_2029	Facilitators and barriers to retention in care under universal antiretroviral therapy (Option B+) for the Prevention of Mother to Child Transmission of HIV (PMTCT): A narrative review	2021	Mbeya Munkhondya et al.	https://doi.org/10.1016/j.ijans.2021.100372	Narrative review	HIV positive mothers	Multiple levels	sub-Saharan Africa	Non-commercial	No	

0. IDENTIFICATION	1. BIBLIOGRAPHIC SOURCE					2. STUDY CHARACTERISTICS						3. INEQUITIES IN HEALTH	
	Title	Year	First author	UHL		Study design	Target units	Sample coverage	African countries or regions involved	Types of funding		Place of residence	Name (acronym)
D_2034	The consequences of declining population access to insecticide-treated nets (ITNs) on net use patterns and physical degradation of nets after 22 months of ownership	2021	Mboma et al.	https://doi.org/10.1186/s12936-021-03686-2		Cross-sectional	Households	Sub-national	Tanzania	Non-commercial		Yes	District (eight)
D_2036	Mosquito net coverage in years between mass distributions: a case study of Tanzania, 2013	2018	Mboma et al.	https://doi.org/10.1186/s12936-018-2247-z		Cross-sectional	Household members	Sub-national	Tanzania	Non-commercial		Yes	District (eight)
D_2078	Factors associated with contraceptive use in Tigray, North Ethiopia	2017	Medhanyie et al.	https://doi.org/10.1186/s12978-017-0281-x		Cross-sectional	Women of reproductive age group (15–49)	Sub-national	Ethiopia	Non-commercial		Yes	1) Residence; 2) Zone

0. IDENTIFICATION	1. BIBLIOGRAPHIC SOURCE					2. STUDY CHARACTERISTICS						3. INEQUITIES IN HEALTH	
	**Title**	**Year**	**First author**	**UHL**		**Study design**	**Target units**	**Sample coverage**	**African countries or regions involved**	**Types of funding**		**Place of residence**	**Name (acronym)**
D_2097	Adolescent sexual and reproductive health in sub-Saharan Africa: who is left behind?	2020	Melesse et al.	https://doi.org/10.1136/bmjgh-2019-002231		Multi-pronged study design	Women	National	33 countries in sub-Saharan Africa	Non-commercial		Yes	1) Residence; 2) Region; 3) 33 Countries;
D_2105	Sub-national levels and trends in contraceptive prevalence, unmet need, and demand for family planning in Nigeria with survey uncertainty	2019	Mercer et al.	https://doi.org/10.1186/s12889-019-8043-z		Cross-sectional	Women	National	Nigeria	Non-commercial		Yes	State (map)

0. IDENTIFICATION	1. BIBLIOGRAPHIC SOURCE					2. STUDY CHARACTERISTICS						3. INEQUITIES IN HEALTH	
	**Title**	**Year**	**First author**	**UHL**		**Study design**	**Target units**	**Sample coverage**	**African countries or regions involved**	**Types of funding**		**Place of residence**	**Name (acronym)**
D_2133	Exploring inequities in skilled care at birth among migrant population in a metropolitan city Addis Ababa, Ethiopia; A qualitative study	2014	Mirkuzie	http://www.equityhealthj.com/content/13/1/110		Qualitative	Migrant women	Organizational	Ethiopia	Non-commercial		No	
D_2187	Factors associated with the use of mosquito bed nets: results from two cross-sectional household surveys in Zambezia Province, Mozambique	2016	Moon et al.	https://doi.org/10.1186/s12936-016-1250-5		Pre-Post	Households	Sub-national	Mozambique	Unclear		Yes	District
D_2236	Sociocultural and Institutional Constraints to Family Planning Uptake Among Migrant Female Head Porters in Madina, a Suburb of Accra, Ghana	2021	Munemo et al.	https://doi.org/10.1177/0886109920954419		Qualitative	Migrant women	Sub-national	Ghana	No funding		No	

0. IDENTIFICATION	1. BIBLIOGRAPHIC SOURCE				2. STUDY CHARACTERISTICS					3. INEQUITIES IN HEALTH	
	**Title**	**Year**	**First author**	**UHL**	**Study design**	**Target units**	**Sample coverage**	**African countries or regions involved**	**Types of funding**	**Place of residence**	**Name (acronym)**
D_2261	Reframing non-communicable diseases and injuries for equity in the era of universal health coverage: Findings and recommendations from the Kenya NCDI poverty commission	2021	Mwangi et al.	https://doi.org/10.5334/aogh.3085	Multi-pronged study design	All	National	Kenya	Unclear	Yes	Location

0. IDENTIFICATION	1. BIBLIOGRAPHIC SOURCE					2. STUDY CHARACTERISTICS						3. INEQUITIES IN HEALTH	
	Title	Year	First author	UHL		Study design	Target units	Sample coverage	African countries or regions involved	Types of funding		Place of residence	Name (acronym)
D_2263	Inequities and their determinants in coverage of maternal health services in Burkina Faso	2018	Mwase et al.	https://doi.org/10.1186/s12939-018-0770-8		Cross-sectional	Pregnancy women	Sub-national	Burkina Faso	Non-commercial		Yes	Region (District)

0. IDENTIFICATION	1. BIBLIOGRAPHIC SOURCE				2. STUDY CHARACTERISTICS					3. INEQUITIES IN HEALTH	
	**Title**	**Year**	**First author**	**UHL**	**Study design**	**Target units**	**Sample coverage**	**African countries or regions involved**	**Types of funding**	**Place of residence**	**Name (acronym)**
D_2289	A Cross-Sectional Study on Hypertension Medication Adherence in a High-Burden Region in Namibia: Exploring Hypertension Interventions and Validation of the Namibia Hill-Bone Compliance Scale	2022	Nakwafila et al.	https://doi.org/10.3390/ijerph19074416	Cross-sectional	Blood pressure patients	Sub-national	Namibia	No funding	No	
D_2305	Assessing Adherence to Antihypertensive Therapy in Primary Health Care in Namibia: Findings and Implications	2017	Nashilongo et al.	https://doi.org/10.1007/s10557-017-6756-8	Cross-sectional	Patients with hypertension	Organizational	Namibia	Unclear	No	

0. IDENTIFICATION	1. BIBLIOGRAPHIC SOURCE				2. STUDY CHARACTERISTICS					3. INEQUITIES IN HEALTH	
	**Title**	**Year**	**First author**	**UHL**	**Study design**	**Target units**	**Sample coverage**	**African countries or regions involved**	**Types of funding**	**Place of residence**	**Name (acronym)**
D_2329	Incomplete vaccination and associated factors among children aged 12–23 months in South Africa: an analysis of the South African demographic and health survey 2016	2021	Ndwandwe et al.	https://doi.org/10.1080/21645515.2020.1791509	Cross-sectional	Children aged 12–23 months	National	South Africa	Non-commercial	Yes	1) Residence; 2) Province of residence (nine)
D_2353	Determining the effective coverage of maternal and child health services in Kenya, using demographic and health survey data sets: tracking progress towards universal health coverage	2017	Nguhiu et al.	https://doi.org/10.1111/tmi.12841	Cross-sectional	Women, children under 5 years	National	Kenya	Non-commercial	No	

0. IDENTIFICATION	1. BIBLIOGRAPHIC SOURCE					2. STUDY CHARACTERISTICS						3. INEQUITIES IN HEALTH	
	**Title**	**Year**	**First author**	**UHL**		**Study design**	**Target units**	**Sample coverage**	**African countries or regions involved**	**Types of funding**		**Place of residence**	**Name (acronym)**
D_2375	Coverage and usage of insecticide treated nets (ITNs) within households: associated factors and effect on the prevalance of malaria parasitemia in the Mount Cameroon area	2019	Njumkeng et al.	https://doi.org/10.1186/s12889-019-7555-x		Cross-sectional	Households	Sub-national	Cameroon	No funding		Yes	Six major settlements
D_2403	Socioeconomic inequalities in maternal health care utilization in Ghana	2019	Novignon et al.	https://doi.org/10.1186/s12939-019-1043-x		Cross-sectional	Household women aged 15 to 49	National	Ghana	No funding		Yes	1) Place of residence; 2) Region of residence

0. IDENTIFICATION	1. BIBLIOGRAPHIC SOURCE				2. STUDY CHARACTERISTICS					3. INEQUITIES IN HEALTH	
	**Title**	**Year**	**First author**	**UHL**	**Study design**	**Target units**	**Sample coverage**	**African countries or regions involved**	**Types of funding**	**Place of residence**	**Name (acronym)**
D_2419	Why rural women do not use primary health centres for pregnancy care: Evidence from a qualitative study in Nigeria	2019	Ntoimo et al.	https://doi.org/10.1186/s12884-019-2433-1	Qualitative	Women and men in marital union	Sub-national	Nigeria	Non-commercial	No	
D_2421	Long-lasting insecticidal net (LLIN) ownership, use and cost of implementation after a mass distribution campaign in Kasaï Occidental Province, Democratic Republic of Congo	2017	Ntuku et al.	https://doi.org/10.1186/s12936-016-1671-1	Pre-Post	Household members	Sub-national	Democratic Republic of the Congo	Non-commercial	No	
D_2457	Explaining socioeconomic disparities and gaps in the use of antenatal care services in 36 countries in sub-Saharan Africa	2021	Obse & Ataguba	https://doi.org/10.1093/heapol/czab036	Cross-sectional	Women	National	36 countries in sub-Saharan Africa	Unclear	Yes	1) Location; 2) Region of residence (e.g., subnational); 3) 36 sub-sharan countries

0. IDENTIFICATION	1. BIBLIOGRAPHIC SOURCE					2. STUDY CHARACTERISTICS						3. INEQUITIES IN HEALTH	
	**Title**	**Year**	**First author**	**UHL**		**Study design**	**Target units**	**Sample coverage**	**African countries or regions involved**	**Types of funding**		**Place of residence**	**Name (acronym)**
D_2491	Sociodemographic factors associated with the use of insecticide treated nets among under-fives in Nigeria: Evidence from a national survey	2022	Ojo et al.	https://doi.org/10.1177/00494755221110374		Cross-sectional	Children under-fives	National	Nigeria	Non-commercial		Yes	1) Residence; 2) Region
D_2556	Towards making efficient use of household resources for appropriate prevention of malaria: investigating households' ownership, use and expenditures on ITNs and other preventive tools in Southeast Nigeria	2014	Onwujekwe et al.	http://www.biomedcentral.com/1471-2458/14/315		Cross-sectional	Households with children under 5 years old	Sub-national	Nigeria	Non-commercial		No	

0. IDENTIFICATION	1. BIBLIOGRAPHIC SOURCE				2. STUDY CHARACTERISTICS					3. INEQUITIES IN HEALTH	
	**Title**	**Year**	**First author**	**UHL**	**Study design**	**Target units**	**Sample coverage**	**African countries or regions involved**	**Types of funding**	**Place of residence**	**Name (acronym)**
D_2578	Demographic disparities in unimproved drinking water and sanitation in Ghana: A nationally representative cross-sectional study	2022	Oppong et al.	http://dx.doi.org/10.1136/bmjopen-2021-060595	Cross-sectional	Occupied households, household heads (an adult man or woman recognised as the head of the unit by the other members of the household unit or by herself if living alone)	National	Ghana	No funding	Yes	1) Place of residence; 2) Region of residence; 3) Zone
D_2630	Inequities in Access to Maternal Health Care in Enugu State: Implications for Universal Health Coverage to Meet Vision 2030 in Nigeria	2019	Ozumba et al.	https://doi.org/10.1177/0272684X18819977	Multi-pronged study design	Women who had babies	Sub-national	Nigeria	No funding	Yes	Locality

0. IDENTIFICATION	1. BIBLIOGRAPHIC SOURCE					2. STUDY CHARACTERISTICS						3. INEQUITIES IN HEALTH	
	**Title**	**Year**	**First author**	**UHL**		**Study design**	**Target units**	**Sample coverage**	**African countries or regions involved**	**Types of funding**		**Place of residence**	**Name (acronym)**
D_2649	How do supply- and demand-side interventions influence equity in healthcare utilisation? Evidence from maternal healthcare in Senegal	2019	Parmar & Banerjee	https://doi.org/10.1016/j.socscimed.2019.112582		Cross-sectional	Women (15–49 years)	National	Senegal	Unclear		Yes	Location
D_2737	Temporal and regional variations in use, equity and quality of antenatal care in Egypt: A repeat cross-sectional analysis using Demographic and Health Surveys	2019	Pugliese-Garcia et al.	https://doi.org/10.1186/s12884-019-2409-1		Cross-sectional	All ever-married women aged 15–49 with a live birth	National	Egypt	Unclear		Yes	1) Residence; 2) Administrative regions

0. IDENTIFICATION	1. BIBLIOGRAPHIC SOURCE				2. STUDY CHARACTERISTICS					3. INEQUITIES IN HEALTH	
	Title	Year	First author	UHL	Study design	Target units	Sample coverage	African countries or regions involved	Types of funding	Place of residence	Name (acronym)
D_2739	Geographical Inequalities in Use of Improved Drinking Water Supply and Sanitation across Sub-Saharan Africa: Mapping and Spatial Analysis of Cross-sectional Survey Data	2014	Pullan et al.	https://doi.org/10.1371/journal.pmed.1001626	Cross-sectional	Households	National	Sub-Saharan Africa (SSA)	Non-commercial	Yes	1) Residence; 2) Sub-Saharan Africa (SSA) countries
D_2763	Individual and Network Factors Associated With HIV Care Continuum Outcomes Among Nigerian MSM Accessing Health Care Services	2018	Ramadhani et al.	https://dx.doi.org/10.1097/QAI.0000000000001756	Cross-sectional	Men who have sex with men (MSM)	Organizational	Nigeria	Non-commercial	No	
D_2808	Non-adherence to long-lasting insecticide treated bednet use following successful malaria control in Tororo, Uganda	2020	Rek et al.	https://doi.org/10.1371/journal.pone.0243303	Longitudinal	Households	Sub-national	Uganda	Non-commercial	No	

0. IDENTIFICATION	1. BIBLIOGRAPHIC SOURCE					2. STUDY CHARACTERISTICS						3. INEQUITIES IN HEALTH	
	**Title**	**Year**	**First author**	**UHL**		**Study design**	**Target units**	**Sample coverage**	**African countries or regions involved**	**Types of funding**		**Place of residence**	**Name (acronym)**
D_2813	Design, implementation and evaluation of a national campaign to deliver 18 million free long-lasting insecticidal nets to uncovered sleeping spaces in Tanzania	2013	Renggli et al.	https://doi.org/10.1186/1475-2875-12-85		Cross-sectional	Children under 5	Sub-national	Tanzania	Unclear		Yes	Geographic areas
D_2861	A long way to go - Estimates of combined water, sanitation and hygiene coverage for 25 sub-Saharan African countries	2017	Roche et al.	https://doi.org/10.1371/journal.pone.0171783		Cross-sectional	Household members	National	Sub-Saharan Africa (25 countries)	No funding		Yes	1) Residence; 2) 25 Sub-Saharan African (SSA) Countries

0. IDENTIFICATION	1. BIBLIOGRAPHIC SOURCE					2. STUDY CHARACTERISTICS						3. INEQUITIES IN HEALTH	
	Title	Year	First author	UHL		Study design	Target units	Sample coverage	African countries or regions involved	Types of funding		Place of residence	Name (acronym)
D_2911	Determinants of bed net use in southeast Nigeria following mass distribution of LLINs: Implications for social behavior change interventions	2015	Russell et al.	https://doi.org/10.1371/journal.pone.0139447		Cross-sectional	Households	Sub-national	Nigeria	Non-commercial		No	
D_2917	Factors associated with the non-use of insecticide-treated nets in Rwandan children	2016	Ruyange et al.	https://doi.org/10.1186/s12936-016-1403-6		Cross-sectional	Women of reproductive age, men aged 15–59 years, Children	National	Rwanda	Non-commercial		Yes	Residence

0. IDENTIFICATION	1. BIBLIOGRAPHIC SOURCE					2. STUDY CHARACTERISTICS						3. INEQUITIES IN HEALTH	
	**Title**	**Year**	**First author**	**UHL**		**Study design**	**Target units**	**Sample coverage**	**African countries or regions involved**	**Types of funding**		**Place of residence**	**Name (acronym)**
D_2959	Wealth Status, Health Insurance, and Maternal Health Care Utilization in Africa: Evidence from Gabon	2020	Sanogo & Yaya	https://dx.doi.org/10.1155/2020/4036830		Cross-sectional	Reproductive aged women	National	Gabon	Unclear		Yes	1) Residency; 2) Region

0. IDENTIFICATION	1. BIBLIOGRAPHIC SOURCE				2. STUDY CHARACTERISTICS					3. INEQUITIES IN HEALTH	
	Title	Year	First author	UHL	Study design	Target units	Sample coverage	African countries or regions involved	Types of funding	Place of residence	Name (acronym)
D_2991	Factors associated with use of insecticide-treated net for malaria prevention in Manica District, Mozambique: a community-based cross-sectional survey	2021	Scott et al.	https://doi.org/10.1186/s12936-021-03738-7	Cross-sectional	Household members (Children less than 10 years of age were excluded as previous studies suggest children particularly those under the age of 5 years often accompany caregivers on travel for family-related	Sub-national	Mozambique	Non-commercial	No	
D_3028	Healthcare utilisation, cancer screening and potential barriers to accessing cancer care in rural South West Nigeria: a cross-sectional study	2021	Sharma et al.	https://doi.org/10.1136/bmjopen-2020-040352	Cross-sectional	Adults >18 years	Sub-national	Nigeria	Non-commercial	No	

0. IDENTIFICATION	1. BIBLIOGRAPHIC SOURCE				2. STUDY CHARACTERISTICS					3. INEQUITIES IN HEALTH	
	Title	Year	First author	UHL	Study design	Target units	Sample coverage	African countries or regions involved	Types of funding	Place of residence	Name (acronym)
D_3044	Socio-economic inequalities in ANC attendance among mothers who gave birth in the past 12 months in Debre Brehan town and surrounding rural areas, North East Ethiopia: A community-based survey	2019	Shibre & Mekonnen	https://doi.org/10.1186/s12978-019-0768-8	Cross-sectional	Recently-delivered women (mothers who gave birth in the 12 months prior to the study)	Sub-national	Ethiopia	Non-commercial	No	

0. IDENTIFICATION	1. BIBLIOGRAPHIC SOURCE					2. STUDY CHARACTERISTICS						3. INEQUITIES IN HEALTH	
	Title	Year	First author	UHL		Study design	Target units	Sample coverage	African countries or regions involved	Types of funding		Place of residence	Name (acronym)
D_3065	Individual and environmental characteristics associated with immunization of children in rural areas of Burkina Faso: A multi-level analysis	2007	Sia et al.	https://pubmed.ncbi.nlm.nih.gov/18299262/		Cross-sectional	Children aged 12-23 months	Sub-national	Burkina Faso	Unclear		No	
D_3073	Inequalities in access and utilization of maternal, newborn and child health services in sub-saharan africa: A special focus on urban settings	2021	Sidze et al.	https://dx.doi.org/10.1007/s10995-021-03250-z		Narrative review	Women, children	Multiple levels	sub-Saharan Africa	Non-commercial		Yes	Sub-Saharan Countries

0. IDENTIFICATION	1. BIBLIOGRAPHIC SOURCE				2. STUDY CHARACTERISTICS					3. INEQUITIES IN HEALTH	
	**Title**	**Year**	**First author**	**UHL**	**Study design**	**Target units**	**Sample coverage**	**African countries or regions involved**	**Types of funding**	**Place of residence**	**Name (acronym)**
D_3076	Insecticide-treated bed net access and use among preschool children in Nouna District, Burkina Faso	2020	Sié et al.	https://dx.doi.org/10.1093/inthealth/ihaa003	Cross-sectional	Heads of households	Sub-national	Burkina Faso	Non-commercial	No	
D_3100	Evidence of improving antiretroviral therapy treatment delays: an analysis of eight years of programmatic outcomes in Blantyre, Malawi	2013	Sloan et al.	http://www.biomedcentral.com/1471-2458/13/49[illegible]	Longitudinal	Patients with HIV	Organizational	Malawi	Non-commercial	No	
D_3113	Low use of long-lasting insecticidal nets for malaria prevention in south-central Ethiopia: A community-based cohort study	2019	Solomon et al.	https://doi.org/10.1371/journal.pone.0210578	Longitudinal	Households	Sub-national	Ethiopia	Non-commercial	No	

0. IDENTIFICATION	1. BIBLIOGRAPHIC SOURCE				2. STUDY CHARACTERISTICS					3. INEQUITIES IN HEALTH	
	**Title**	**Year**	**First author**	**UHL**	**Study design**	**Target units**	**Sample coverage**	**African countries or regions involved**	**Types of funding**	**Place of residence**	**Name (acronym)**
D_3118	Empowerment and use of modern contraceptive methods among married women in Burkina Faso: a multilevel analysis	2021	Some et al.	https://dx.doi.org/10.1186/s12889-021-11541-x	Cross-sectional	Married women of reproductive age (15–49 years)	National	Burkina Faso	No funding	Yes	Residence
D_3156	Evaluation of the 2011 long-lasting, insecticide-treated net distribution for universal coverage in Togo	2013	Stevens et al.	http://www.malariajournal.com/content/12/1/162	Cross-sectional	Households	Sub-national	Togo	Non-commercial	Yes	Region
D_3162	On the way to universal coverage of maternal services in Iringa rural District in Tanzania. Who is yet to be reached?	2016	Straneo et al.	https://doi.org/10.4314/ahs.v16i2.10	Cross-sectional	Households	Sub-national	Tanzania	Non-commercial	No	

0. IDENTIFICATION	1. BIBLIOGRAPHIC SOURCE				2. STUDY CHARACTERISTICS					3. INEQUITIES IN HEALTH	
	**Title**	**Year**	**First author**	**UHL**	**Study design**	**Target units**	**Sample coverage**	**African countries or regions involved**	**Types of funding**	**Place of residence**	**Name (acronym)**
D_3164	Evaluation of long-lasting insecticidal net distribution through schools in Southern Tanzania	2022	Stuck et al.	https://doi.org/10.1093/heapol/czab140	Non-Randomized controlled trial	Households	Sub-national	Tanzania	Non-commercial	Yes	Districts
D_3174	Not all inequalities are equal: differences in coverage across the continuum of reproductive health services	2019	Sully et al.	https://dx.doi.org/10.1136/bmjgh-2019-001695	Cross-sectional	Women aged 15-49 years	National	Eastern, Middle, Western and Northern Africa	Non-commercial	Yes	1) Residence, 2) African regions

0. IDENTIFICATION	1. BIBLIOGRAPHIC SOURCE				2. STUDY CHARACTERISTICS					3. INEQUITIES IN HEALTH	
	**Title**	**Year**	**First author**	**UHL**	**Study design**	**Target units**	**Sample coverage**	**African countries or regions involved**	**Types of funding**	**Place of residence**	**Name (acronym)**
D_3241	Determinants of long-lasting insecticidal net ownership and utilization in malaria transmission regions: Evidence from Zimbabwe Demographic and Health Surveys	2019	Tapera	https://doi.org/10.1186/s12936-019-2912-x	Cross-sectional	Household members	National	Zimbabwe	No funding	Yes	1) Place of residence; 2) Region/province
D_3243	Sociodemographic inequities in cervical cancer screening, treatment and care amongst women aged at least 25 years: evidence from surveys in Harare, Zimbabwe	2019	Tapera et al.	https://doi.org/10.1186/s12889-019-6749-6	Cross-sectional	Women aged at least 25 years	Sub-national	Zimbabwe	Non-commercial	Yes	Province of residence

0. IDENTIFICATION	1. BIBLIOGRAPHIC SOURCE					2. STUDY CHARACTERISTICS						3. INEQUITIES IN HEALTH	
	Title	Year	First author	UHL		Study design	Target units	Sample coverage	African countries or regions involved	Types of funding		Place of residence	Name (acronym)
D_3254	Factors associated to bed net use in Cameroon: a retrospective study in Mfou health district in the Centre Region	2012	Tchinda et al.	http://www.panafrican-med-journal.com/content/article/12/112/full/		Cross-sectional	Households	Sub-national	Cameroon	Non-commercial		Yes	Area of residence
D_3256	Cervical cancer screening uptake and correlates among HIV-infected women: a cross-sectional survey in Cote d'Ivoire, West Africa	2019	Tchounga et al.	https://dx.doi.org/10.1136/bmjopen-2019-029882		Cross-sectional	WLHIV aged 25-55 years	Sub-national	Ivory Coast	Non-commercial		No	

0. IDENTIFICATION	1. BIBLIOGRAPHIC SOURCE					2. STUDY CHARACTERISTICS						3. INEQUITIES IN HEALTH	
	**Title**	**Year**	**First author**	**UHL**		**Study design**	**Target units**	**Sample coverage**	**African countries or regions involved**	**Types of funding**		**Place of residence**	**Name (acronym)**
D_3268	Duration and determinants of delayed tuberculosis diagnosis and treatment in high-burden countries: a mixed-methods systematic review and meta-analysis	2021	Teo et al.	https://doi.org/10.1186/s12931-021-01841-6		Systematic review	Presumptive TB and ppl with TB	National	16: Angola, CAR, Congo, RDC, Ethiopia, Kenya, Lesotho, Liberia, Mozambique, Namibia, Nigeria, Sierra Leone, South Africa, Tanzania, Zambia, Zimbabwe	No funding		Yes	Residence
D_3272	Skilled delivery inequality in Ethiopia: To what extent are the poorest and uneducated mothers benefiting?	2017	Tesfaye et al.	https://doi.org/10.1186/s12939-017-0579-x		Longitudinal	Women in a reproductive age group	National	Ethiopia	No funding		No	

0. IDENTIFICATION	1. BIBLIOGRAPHIC SOURCE				2. STUDY CHARACTERISTICS					3. INEQUITIES IN HEALTH	
	Title	Year	First author	UHL	Study design	Target units	Sample coverage	African countries or regions involved	Types of funding	Place of residence	Name (acronym)
D_3284	Investigating the association between pregnancy intention and insecticide-treated bed net (ITN) use: A cross-sectional study of pregnant women in Rwanda	2015	Thogarapalli et al.	https://doi.org/10.1007/s10389-015-0676-5	Cross-sectional	Sample of pregnant women owning an ITN	National	Rwanda	Unclear	Yes	Residence
D_3291	Success of Senegal's first nationwide distribution of long-lasting insecticide-treated nets to children under five - Contribution toward universal coverage	2011	Thwing et al.	http://www.malariajournal.com/content/10/1/86	Cross-sectional	A household (all individuals who eat out of one pot, including guests)	Sub-national	Senegal	Unclear	No	
D_3292	Declines in Malaria Burden and all-cause child mortality following increases in control interventions in Senegal, 2005-2010	2017	Thwing et al.	https://doi.org/10.4269/ajtmh.16-0953	Cross-sectional	Children under five	National	Senegal	Unclear	Yes	Epidemiologic zone

0. IDENTIFICATION	1. BIBLIOGRAPHIC SOURCE				2. STUDY CHARACTERISTICS					3. INEQUITIES IN HEALTH	
	Title	Year	First author	UHL	Study design	Target units	Sample coverage	African countries or regions involved	Types of funding	Place of residence	Name (acronym)
D_3301	Individual-level and community-level determinants of cervical cancer screening among Kenyan women: a multilevel analysis of a Nationwide survey	2017	Tiruneh et al.	https://doi.org/10.1186/s12905-017-0469-9	Cross-sectional	Married women of reproductive age	National	Kenya	No funding	Yes	Place of residence
D_3304	Evaluation of the coverage and effective use rate of long-lasting insecticidal nets after nation-wide scale up of their distribution in Benin	2013	Tokponnon et al.	https://doi.org/10.1186/1756-3305-6-265	Cross-sectional	Households	National	Benin	Non-commercial	Yes	Place of residence
D_3330	Factors associated with the upsurge in the use of delivery care services in Sierra Leone	2020	Tsawe & Susuman	https://doi.org/10.1016/j.puhe.2019.11.002	Cross-sectional	Women of reproductive age (15–49 years)	National	Sierra Leone	Non-commercial	Yes	Place of residence

0. IDENTIFICATION	1. BIBLIOGRAPHIC SOURCE				2. STUDY CHARACTERISTICS					3. INEQUITIES IN HEALTH	
	**Title**	**Year**	**First author**	**UHL**	**Study design**	**Target units**	**Sample coverage**	**African countries or regions involved**	**Types of funding**	**Place of residence**	**Name (acronym)**
D_3333	Which family members use the best nets? An analysis of the condition of mosquito nets and their distribution within households in Tanzania	2010	Tsuang et al.	http://www.malariajournal.com/content/9/1/211	Cross-sectional	Household	National	Tanzania	Non-commercial	No	
D_3357	Utilization of insecticide treated nets among pregnant women in enugu, South Eastern Nigeria	2013	Ugwu et al.	https://pubmed.ncbi.nlm.nih.gov/23771448/	Cross-sectional	Pregnant women	Sub-national	Nigeria	Unclear	No	

0. IDENTIFICATION	1. BIBLIOGRAPHIC SOURCE				2. STUDY CHARACTERISTICS					3. INEQUITIES IN HEALTH	
	Title	Year	First author	UHL	Study design	Target units	Sample coverage	African countries or regions involved	Types of funding	Place of residence	Name (acronym)
D_3377	Finding the gap: Revealing local disparities in coverage of maternal, newborn and child health services in South Sudan using lot quality assurance sampling	2015	Valadez et al.	https://doi.org/10.1111/tmi.12613	Cross-sectional	Household	National	South Sudan	Non-commercial	Yes	County of residence
D_3419	Group Medical Visit and Microfinance Intervention for Patients With Diabetes or Hypertension in Kenya	2021	Vedanthan et al.	https://doi.org/10.1016/j.jacc.2021.03.002	Randomized controlled trial	Adults ≥ 35 years of age having either diabetes or hypertension	National	Kenya	Non-commercial	No	

0. IDENTIFICATION	1. BIBLIOGRAPHIC SOURCE					2. STUDY CHARACTERISTICS						3. INEQUITIES IN HEALTH	
	**Title**	**Year**	**First author**	**UHL**		**Study design**	**Target units**	**Sample coverage**	**African countries or regions involved**	**Types of funding**		**Place of residence**	**Name (acronym)**
D_3460	Equity in Maternal Health in South Africa: Analysis of Health Service Access and Health Status in a National Household Survey	2013	Wabiri et al.	https://doi.org/10.1371/journal.pone.0073864		Cross-sectional	2 groups of women aged 15–55: those who had been pregnant in the past two years and those interviewed as the parent or guardian of a child under 2	National	South Africa	Non-commercial		Yes	Place of residence
D_3474	Persisting Regional Disparities in Modern Contraceptive Use and Unmet Need for Contraception among Nigerian Women	2019	Wang & Cao	https://doi.org/10.1155/2019/9103928		Cross-sectional	Women (15-49 yr)	National	Nigeria	Non-commercial		Yes	Place of residece and Geopolitical region

0. IDENTIFICATION	1. BIBLIOGRAPHIC SOURCE				2. STUDY CHARACTERISTICS					3. INEQUITIES IN HEALTH	
	**Title**	**Year**	**First author**	**UHL**	**Study design**	**Target units**	**Sample coverage**	**African countries or regions involved**	**Types of funding**	**Place of residence**	**Name (acronym)**
D_3479	Effective coverage of facility delivery in Bangladesh, Haiti, Malawi, Nepal, Senegal, and Tanzania	2019	Wang et al.	https://doi.org/10.1371/journal.pone.0217853	Cross-sectional	Women (15-49 yr) with a birth in the 5yr before the survey	National	Senegal, Tanzania, Malawi	Non-commercial	Yes	Delivery by region
D_3486	Use of long-lasting insecticide-treated bed nets in a population with universal coverage following a mass distribution campaign in Uganda	2016	Wanzira et al.	https://doi.org/10.1186/s12936-016-1360-0	Cross-sectional	Households	National	Uganda	No funding	No	

0. IDENTIFICATION	1. BIBLIOGRAPHIC SOURCE				2. STUDY CHARACTERISTICS					3. INEQUITIES IN HEALTH	
	**Title**	**Year**	**First author**	**UHL**	**Study design**	**Target units**	**Sample coverage**	**African countries or regions involved**	**Types of funding**	**Place of residence**	**Name (acronym)**
D_3487	Long-lasting insecticide-treated bed net ownership and use among children under five years of age following a targeted distribution in central Uganda	2014	Wanzira et al.	https://doi.org/10.1186/1475-2875-13-185	Cross-sectional	Households	Sub-national	Uganda	Non-commercial	No	
D_3509	Evaluation of a national universal coverage campaign of long-lasting insecticidal nets in a rural district in north-west Tanzania	2012	West et al.	https://doi.org/10.1186/1475-2875-11-273	Cross-sectional	Households	Sub-national	Tanzania	Non-commercial	No	
D_3568	The impact of renewing long-lasting insecticide-treated nets in the event of malaria resurgence: Lessons from 10 years of net use in dielmo, Senegal	2021	Wotodjo et al.	https://doi.org/10.4269/AJTMH.20-0127	Longitudinal		Sub-national	Senegal	Non-commercial	No	

0. IDENTIFICATION	1. BIBLIOGRAPHIC SOURCE					2. STUDY CHARACTERISTICS						3. INEQUITIES IN HEALTH	
	**Title**	**Year**	**First author**	**UHL**		**Study design**	**Target units**	**Sample coverage**	**African countries or regions involved**	**Types of funding**		**Place of residence**	**Name (acronym)**
D_3573	Wealth and Education Inequities in Maternal and Child Health Services Utilization in Rural Ethiopia	2022	Wuneh et al.	https://doi.org/10.3390/ijerph19095421		Cross-sectional	Households, women in the reproductive age of 15 to 49 years, children aged 12–23 months	Sub-national	Ethiopia	Non-commercial		No	
D_3588	Inequalities in maternal health care utilization in Benin: A population based cross-sectional study	2018	Yaya et al.	https://doi.org/10.1186/s12884-018-1846-6		Cross-sectional	Women aged between 15–49 years	National	Benin	No funding		Yes	1) Type of residence; 2) Geographic Region

0. IDENTIFICATION	1. BIBLIOGRAPHIC SOURCE				2. STUDY CHARACTERISTICS					3. INEQUITIES IN HEALTH	
	**Title**	**Year**	**First author**	**UHL**	**Study design**	**Target units**	**Sample coverage**	**African countries or regions involved**	**Types of funding**	**Place of residence**	**Name (acronym)**
D_3621	Long-lasting insecticide-treated bed net ownership, utilization and associated factors among school-age children in Dara Mallo and Uba Debretsehay districts, Southern Ethiopia	2020	Zerdo et al.	https://dx.doi.org/10.1186/s12936-020-03437-9	Cross-sectional	Households, children	Sub-national	Ethiopia	Non-commercial	No	
D_3625	Trends and projections of universal health coverage indicators in Ghana, 1995-2030: A national and subnational study	2019	Zhang et al.	https://doi.org/10.1371/journal.pone.0209126	Cross-sectional	Households, women, children	National	Ghana	Non-commercial	Yes	10 Regions in Ghana

0. IDENTIFICATION	1. BIBLIOGRAPHIC SOURCE					2. STUDY CHARACTERISTICS						3. INEQUITIES IN HEALTH	
	**Title**	**Year**	**First author**	**UHL**		**Study design**	**Target units**	**Sample coverage**	**African countries or regions involved**	**Types of funding**		**Place of residence**	**Name (acronym)**
G_58	Developing Malawi's Universal Health Coverage Index	2022	Mchenga et al.	https://doi.org/10.3389/frhs.2021.786186		Cross-sectional	Households, women, children	National	Malawi	Unclear		No	
W_03	Socioeconomic Factors Associated with Compliance with Mass Drug Administration for Lymphatic Filariasis Elimination in Kenya: Descriptive Study Results	2012	Njomo et al.	https://www.proquest.com/docview/1115911778?pq-origsite=gscholar&fromopenview=true		Cross-sectional	Households	National	Kenya	Non-commercial		No	

0. IDENTIFICATION	1. BIBLIOGRAPHIC SOURCE				2. STUDY CHARACTERISTICS					3. INEQUITIES IN HEALTH	
	**Title**	**Year**	**First author**	**UHL**	**Study design**	**Target units**	**Sample coverage**	**African countries or regions involved**	**Types of funding**	**Place of residence**	**Name (acronym)**
W_16	Equality analysis of main health indicators among children under 5 years in Uganda	2019	Elduma	http://dx.doi.org/10.4314/ejhs.v29i2.8	Cross-sectional	Children	National	Uganda, Central Republic of Africa, Tanzania, Ethiopia, Democratic republic of Congo, Rwanda, Burundi	Unclear	Yes	Area of residence

1. BIBLIOGRAPHIC SOURCE												
**Title**	**Year**	**First author**	**UHL**	**Measure**	**Race, ethnicity, culture, or language**	**Name (acronym)**	**Measure**	**Occupation**	**Name (acronym)**	**Measure**	**Gender or sex**	**Name (acronym)**
Reproductive Plans And Utilization of Contraceptives Among Women Living With HIV	2019	Adeleye et al.	https://dx.doi.org/10.21106/ijma.277		No			No			No	
Provision of immediate postpartum contraception to women living with HIV in the Eastern Cape, South Africa; a cross-sectional analysis	2020	Adeniyi et al.	https://doi.org/10.1186/s12978-020-01049-9	Rural/Semi-urban/Urban	No			Yes	Employment status	Employed/Unemployed	No	
Moving Up the Sanitation Ladder: A Study of the Coverage and Utilization of Improved Sanitation Facilities and Associated Factors Among Households in Southern Ethiopia	2022	Afework et al.	https://doi.org/10.1177/11786302221080825		No			Yes	Occupation of household head	Farmer/Governmental office/Merchant/Daily laborer and other	No	

1. BIBLIOGRAPHIC SOURCE												
**Title**	**Year**	**First author**	**UHL**	**Measure**	**Race, ethnicity, culture, or language**	**Name (acronym)**	**Measure**	**Occupation**	**Name (acronym)**	**Measure**	**Gender or sex**	**Name (acronym)**
Maternal health care services utilisation in the context of 'Abiye' (safe motherhood) programme in Ondo State, Nigeria	2020	Ajayi et al.	https://doi.org/10.1186/s12889-020-08512-z	Rural/Urban	Yes	Ethnic groups	Yoruba/Others	No			No	
Slums, women and sanitary living in South-South Nigeria	2021	Akpabio et al.	https://doi.org/10.1007/s10901-020-09802-z		No			No			Yes	Gender
Leaving no child behind: Decomposing socioeconomic inequalities in child health for india and south africa	2021	Alaba et al.	https://doi.org/10.3390/ijerph18137114	Urban/Rural	No			No			Yes	Sex of household head

1. BIBLIOGRAPHIC SOURCE												
Title	Year	First author	UHL	Measure	Race, ethnicity, culture, or language	Name (acronym)	Measure	Occupation	Name (acronym)	Measure	Gender or sex	Name (acronym)
Access to skilled attendant at birth and the coverage of the third dose of diphtheria-tetanus-pertussis vaccine across 14 West African countries - An equity analysis	2020	Alhassan et al.	https://doi.org/10.1186/s12939-020-01204-5	Urban/Rural, Region of Residence	No			No			No	
Trends and correlates of maternal, newborn and child health services utilization in primary healthcare facilities: An explorative ecological study using DHIMSII data from one district in the Volta region of Ghana	2020	Alhassan et al.	https://doi.org/10.1186/s12884-020-03195-1	Urban/Rural	No			No			No	

1. BIBLIOGRAPHIC SOURCE												
Title	Year	First author	UHL	Measure	Race, ethnicity, culture, or language	Name (acronym)	Measure	Occupation	Name (acronym)	Measure	Gender or sex	Name (acronym)
Determinants of equity in utilization of maternal health services in Butajira, Southern Ethiopia	2012	Aliy & Mariam	https://www.ajol.info/index.php/ejhd/article/view/116114	Urban/Rural	No			Yes	Farmer/Non-farmer		Yes	Sex of household head
Determinants of insecticide treated nets use among youth corp members in Edo State, Nigeria	2011	Amoran O. E. et al	http://www.biomedcentral.com/1471-2458/11/728	South-West/South-East/South-South/North-West/North-East/North Central	No			No			Yes	Sex

1. BIBLIOGRAPHIC SOURCE													
**Title**	**Year**	**First author**	**UHL**	**Measure**	**Race, ethnicity, culture, or language**	**Name (acronym)**	**Measure**	**Occupation**	**Name (acronym)**	**Measure**	**Gender or sex**	**Name (acronym)**	
Towards achievement of Sustainable Development Goal 3: multilevel analyses of demographic and health survey data on health insurance coverage and maternal healthcare utilisation in sub-Saharan Africa	2022	Amu et al.	https://doi.org/10.1093/inthealth/ihac017	Urban/Rural	No			Yes	Current working status	Yes/No	No		

1. BIBLIOGRAPHIC SOURCE													
**Title**	**Year**	**First author**	**UHL**	**Measure**	**Race, ethnicity, culture, or language**	**Name (acronym)**	**Measure**	**Occupation**	**Name (acronym)**	**Measure**	**Gender or sex**	**Name (acronym)**	
Equity and access to maternal and child health services in Ghana a cross-sectional study	2021	Anarwat et al.	https://doi.org/10.1186/s12913-021-06872-9	Urban/Rural	No			No			No		
A subnational profiling analysis reveals regional differences as the main predictor of ITN ownership and use in Nigeria	2019	Andrada et al.	https://doi.org/10.1186/s12936-019-2816-9	Urban/Rural, South-West/South-East/South-South/North-West/North-East/North Central	No			No			No		
Inequality trends in maternal health services for young Ghanaian women with childbirth history between 2003 and 2014	2017	Asamoah & Agardh	https://doi.org/10.1136/bmjopen-2016-011663	Urban/Rural	No			No			No		

1. BIBLIOGRAPHIC SOURCE												
**Title**	**Year**	**First author**	**UHL**	**Measure**	**Race, ethnicity, culture, or language**	**Name (acronym)**	**Measure**	**Occupation**	**Name (acronym)**	**Measure**	**Gender or sex**	**Name (acronym)**
Leaving no one behind: Lessons from implementation of policies for universal HIV treatment to universal health coverage	2020	Assefa et al.	https://doi.org/10.1186/s12992-020-00549-4	Region	No			No			Yes	Gender
Inequalities in child immunization coverage in Ghana: evidence from a decomposition analysis	2018	Asuman et al.	https://doi.org/10.1186/s13561-018-0193-7	Urban/Rural	No			Yes	Employment	No employment/Family or other employee/self-employed	Yes	Gender of child
A reassessment of global antenatal care coverage for improving maternal health using sub-Saharan Africa as a case study	2018	Ataguba	https://dx.doi.org/10.1371/journal.pone.0204822	Urban/Rural	No			No			No	

1. BIBLIOGRAPHIC SOURCE												
**Title**	**Year**	**First author**	**UHL**	**Measure**	**Race, ethnicity, culture, or language**	**Name (acronym)**	**Measure**	**Occupation**	**Name (acronym)**	**Measure**	**Gender or sex**	**Name (acronym)**
Socio-economic inequality in maternal health care utilization in Sub-Saharan Africa: Evidence from Togo	2021	Atake	http://dx.doi.org/10.1002/hpm.3083	Urban/Rural	No			No			No	
Poor prenatal service utilization and pregnancy outcome in a tertiary health facility in southwest Nigeria	2020	Awoleke & Olofinbiyi	https://doi.org/10.11604/pamj.2020.35.28.20426		Yes	Ethnicity	Igbo/Yoruba/Hausa/Others	Yes	Employment of woman and husband	Unemployed/Employed	No	
Correlates of intra-household ITN use in Liberia: A multilevel analysis of household survey data	2016	Babalola et al.	http://dx.doi.org/10.1371/journal.pone.0158331	Bong, Cape Mount, Grand Kru or Rivercess.	No			No			Yes	Sex of household member

1. BIBLIOGRAPHIC SOURCE												
**Title**	**Year**	**First author**	**UHL**	**Measure**	**Race, ethnicity, culture, or language**	**Name (acronym)**	**Measure**	**Occupation**	**Name (acronym)**	**Measure**	**Gender or sex**	**Name (acronym)**
Prevalence and determinants of maternal healthcare utilisation among young women in sub-Saharan Africa: cross-sectional analyses of demographic and health survey data	2022	Bain et al.	https://doi.org/10.1186/s12889-022-13037-8	Urban/Rural	No			Yes	Maternal current working status	Not working/Working	Yes	Sex of household head

1. BIBLIOGRAPHIC SOURCE												
Title	Year	First author	UHL	Measure	Race, ethnicity, culture, or language	Name (acronym)	Measure	Occupation	Name (acronym)	Measure	Gender or sex	Name (acronym)
Factors influencing timing and frequency of antenatal care in Uganda	2011	Bbaale	https://www.ncbi.nlm.nih.gov/pmc/articles/PMC3562883/pdf/AMJ-04-431.pdf	Urban/Rural, East/North/West	No			Yes	Maternal and Partner's occupation	Agriculture/Services/Blue collar/White collar	No	
Maternal education and childbirth care in Uganda	2011	Bbaale & Guloba	https://www.ncbi.nlm.nih.gov/pmc/articles/PMC3562941/pdf/AMJ-04-389.pdf	Urban/Rural, East/North/West	No			Yes	Maternal and Partner's occupation	Agriculture/Services/Blue collar/White collar	No	

1. BIBLIOGRAPHIC SOURCE												
**Title**	**Year**	**First author**	**UHL**	**Measure**	**Race, ethnicity, culture, or language**	**Name (acronym)**	**Measure**	**Occupation**	**Name (acronym)**	**Measure**	**Gender or sex**	**Name (acronym)**
A quantitative analysis of food insecurity and other barriers associated with ART nonadherence among women in rural communities of Eswatini	2021	Becker et al.	https://doi.org/10.1371/journal.pone.0256277		No			Yes	Employment status	Employed/Unemployed	No	
Two decades of antenatal and delivery care in Uganda: a cross-sectional study using Demographic and Health Surveys	2018	Benova et al.	https://doi.org/10.1186/s12913-018-3546-3	Urban/Rural, Western/Eastern/North/Central	No			No			No	
Utilization of sexual and reproductive health services in ethiopia - Does it affect sexual activity among high school students?	2015	Bilal et al.	https://doi.org/10.1016/j.srhc.2014.09.009		No			No			Yes	Gender

1. BIBLIOGRAPHIC SOURCE													
**Title**	**Year**	**First author**	**UHL**	**Measure**	**Race, ethnicity, culture, or language**	**Name (acronym)**	**Measure**	**Occupation**	**Name (acronym)**	**Measure**	**Gender or sex**	**Name (acronym)**	
Addressing the huge poor–rich gap of inequalities in accessing safe childbirth care: A first step to achieving universal maternal health coverage in Tanzania	2021	Bintabara	https://doi.org/10.1371/journal.pone.0246995	Urban/Rural	No			Yes	Employment	Not employed/ Employed	No		
Twelve-year persistence of inequalities in antenatal care utilisation among women in Tanzania: A decomposition analysis of population-based cross-sectional surveys	2021	Bintabara & Basinda	https://doi.org/10.1136/bmjopen-2020-040450	Urban/Rural	No			Yes	Employment	Not employed/ Employed	No		
Socio-demographic and economic inequalities in modern contraception in 11 low- And middle-income countries: An analysis of the PMA2020 surveys	2020	Blumenberg et al.	https://doi.org/10.1186/s12978-020-00931-w		No			No			No		

1. BIBLIOGRAPHIC SOURCE												
**Title**	**Year**	**First author**	**UHL**	**Measure**	**Race, ethnicity, culture, or language**	**Name (acronym)**	**Measure**	**Occupation**	**Name (acronym)**	**Measure**	**Gender or sex**	**Name (acronym)**
A decomposition analysis of change in skilled birth attendants, 2003 to 2008, Ghana demographic and health surveys	2014	Bosomprah et al.	https://doi.org/10.1186/s12884-014-0415-x		No			No			No	
Slow and Steady can Still Win the Race': Childhood Vaccination Experience of Migrant Ebira Women Within the Health System in Ekiti State, Nigeria	2021	Olakanmi-Falade & Awoleke	https://www.ojhas.org/issue79/2021-3-3.html		No			Yes	Occupation	Unemployed/Unskilled/Semi-skilled/Skilled/	No	
Antiretroviral therapy in Walvis Bay, Namibia	2016	Callaghan	https://hdl.handle.net/1807/70825		No			No			Yes	Gender

1. BIBLIOGRAPHIC SOURCE												
**Title**	**Year**	**First author**	**UHL**	**Measure**	**Race, ethnicity, culture, or language**	**Name (acronym)**	**Measure**	**Occupation**	**Name (acronym)**	**Measure**	**Gender or sex**	**Name (acronym)**
Family planning, antenatal and delivery care: Cross-sectional survey evidence on levels of coverage and inequalities by public and private sector in 57 low- and middle-income countries	2016	Campbell et al.	https://doi.org/10.1111/tmi.12681		No			No			No	

1. BIBLIOGRAPHIC SOURCE												
**Title**	**Year**	**First author**	**UHL**	**Measure**	**Race, ethnicity, culture, or language**	**Name (acronym)**	**Measure**	**Occupation**	**Name (acronym)**	**Measure**	**Gender or sex**	**Name (acronym)**
Use of family planning and child health services in the private sector: An equity analysis of 12 DHS surveys	2018	Chakraborty & Sprockett	https://doi.org/10.1186/s12939-018-0763-7	Urban/Rural	No			No			No	
Meeting demand for family planning within a generation: prospects and implications at country level	2015	Choi et al.	https://dx.doi.org/10.3402/gha.v8.29734	Urban/Rural	No			No			No	
A Longitudinal Analysis of Mosquito Net Ownership and Use in an Indigenous Batwa Population after a Targeted Distribution	2016	Clark et al.	https://doi.org/10.1371/journal.pone.0154808		No			No			No	

1. BIBLIOGRAPHIC SOURCE												
**Title**	**Year**	**First author**	**UHL**	**Measure**	**Race, ethnicity, culture, or language**	**Name (acronym)**	**Measure**	**Occupation**	**Name (acronym)**	**Measure**	**Gender or sex**	**Name (acronym)**
Malaria prevalence and long-lasting insecticidal net use in rural western Uganda: results of a cross-sectional survey conducted in an area of highly variable malaria transmission intensity	2021	Cote et al.	https://doi.org/10.1186/s12936-021-03835-7	Quartile 1/2/3/4	No			No			No	
Women's Sexual Empowerment and Contraceptive Use in Ghana	2012	Crissman et al.	https://doi.org/10.1111/j.1728-4465.2012.00318.x	Urban/Rural, North/South	No			Yes	Employment	Unemployment/Employment	No	
Socio-economic and demographic factors associated with reproductive and child health preventive care in Mozambique: a cross-sectional study	2020	Daca et al.	https://doi.org/10.1186/s12939-020-01303-3	Urban/Rural, Northern/Southern/Central	No			Yes	Occupation class	Non-manual/Farmers/Manual/Not working	No	

1. BIBLIOGRAPHIC SOURCE												
**Title**	**Year**	**First author**	**UHL**	**Measure**	**Race, ethnicity, culture, or language**	**Name (acronym)**	**Measure**	**Occupation**	**Name (acronym)**	**Measure**	**Gender or sex**	**Name (acronym)**
Assessing the contextual effect of community in the utilization of postnatal care services in Ghana	2021	Dankwah et al.	https://doi.org/10.1186/s12913-020-06028-1	Regions in Ghana	Yes	Ethnicity	Akan/ Northern tribes/ Ewe/ /Ga/Other	Yes	Occupation	Working/ Not Working	No	
Is South Africa closing the health gaps between districts? Monitoring progress towards universal health service coverage with routine facility data	2021	Day et al.	https://doi.org/10.1186/s12913-021-06171-3		No			No			No	
Neglected tropical diseases as a 'litmus test' for universal health coverage? understanding who is left behind and why in mass drug administration: Lessons from four country contexts	2019	Dean et al.	https://doi.org/10.1371/journal.pntd.0007847		No			No			Yes	Gender

1. BIBLIOGRAPHIC SOURCE												
**Title**	**Year**	**First author**	**UHL**	**Measure**	**Race, ethnicity, culture, or language**	**Name (acronym)**	**Measure**	**Occupation**	**Name (acronym)**	**Measure**	**Gender or sex**	**Name (acronym)**
Preventive Health Service Coverage Among Infants and Children at Six Maternal-Child Health Clinics in Western Kenya: A Cross-Sectional Assessment	2022	Deathe et al.	https://doi.org/10.1007/s10995-021-03271-8		No			No			Yes	Gender
Distance, difference in altitude and socioeconomic determinants of utilisation of maternal and child health services in Ethiopia: A geographic and multilevel modelling analysis	2021	Defar et al.	https://doi.org/10.1136/bmjopen-2020-042095	Region	No			No			Yes	Gender of child
Geographic differences in maternal and child health care utilization in four Ethiopian regions; A cross-sectional study	2019	Defar et al.	https://doi.org/10.1186/s12939-019-1079-y		No			No			Yes	Gender of child

1. BIBLIOGRAPHIC SOURCE													
**Title**	**Year**	**First author**	**UHL**	**Measure**	**Race, ethnicity, culture, or language**	**Name (acronym)**	**Measure**	**Occupation**	**Name (acronym)**	**Measure**	**Gender or sex**	**Name (acronym)**
Ownership and use of insecticide-treated nets in Oromia and Amhara Regional States of Ethiopia twoyears after a nationwide campaign	2011	Deressa et al.	https://doi.org/10.1111/j.1365-3156.2011.02875.x	Urban/Rural; Region	No			No			No	
Effectiveness of post-campaign, door-to-door, hang-up, and communication interventions to increase long-lasting, insecticidal bed net utilization in Togo (2011-2012): A cluster randomized, control trial	2014	Desrochers et al.	https://doi.org/10.1186/1475-2875-13-260		No			No			No	
Immediate postnatal care following childbirth in Ugandan health facilities: An analysis of Demographic and Health Surveys between 2001 and 2016	2021	Dey et al.	https://doi.org/10.1136/bmjgh-2020-004230	Urban/Rural, Geographical zone	No			No			No	

1. BIBLIOGRAPHIC SOURCE												
**Title**	**Year**	**First author**	**UHL**	**Measure**	**Race, ethnicity, culture, or language**	**Name (acronym)**	**Measure**	**Occupation**	**Name (acronym)**	**Measure**	**Gender or sex**	**Name (acronym)**
Inequalities in non-communicable diseases and effective responses	2013	Di Cesare et al.	https://doi.org/10.1016/s0140-6736(12)61851-0		No			No			Yes	Gender
Insecticide-treated nets ownership and utilization among under-five children following the 2010 mass distribution in Burkina Faso	2014	Diabate et al.	https://dx.doi.org/10.1186/1475-2875-13-353	Urban/Peri-urban/Rural	No			No			No	

1. BIBLIOGRAPHIC SOURCE												
Title	Year	First author	UHL	Measure	Race, ethnicity, culture, or language	Name (acronym)	Measure	Occupation	Name (acronym)	Measure	Gender or sex	Name (acronym)
Impact of mining projects on water and sanitation infrastructures and associated child health outcomes: a multi-country analysis of Demographic and Health Surveys (DHS) in sub-Saharan Africa	2021	Dietler et al.	https://doi.org/10.1186/s12992-021-00723-2		No			No			No	

1. BIBLIOGRAPHIC SOURCE												
**Title**	**Year**	**First author**	**UHL**	**Measure**	**Race, ethnicity, culture, or language**	**Name (acronym)**	**Measure**	**Occupation**	**Name (acronym)**	**Measure**	**Gender or sex**	**Name (acronym)**
Predictors of insecticidal net use among internally displaced persons aged 6-59 months in Abuja, Nigeria	2018	Ejembi et al.	http://dx.doi.org/10.11604/pamj.2018.29.136.133 22		No			No			Yes	Gender of child
Changes in Inequality in Use of Maternal Health Care Services: Evidence from Skilled Birth Attendance in Mauritania for the Period 2007-2015	2022	Taleb El Hassen et al.	https://doi.org/10.3390/ijerph19063566	1) Rural, Urban; 2) Nouakchott, Hodh Charghy, and so forth	No			No			No	

1. BIBLIOGRAPHIC SOURCE												
**Title**	**Year**	**First author**	**UHL**	**Measure**	**Race, ethnicity, culture, or language**	**Name (acronym)**	**Measure**	**Occupation**	**Name (acronym)**	**Measure**	**Gender or sex**	**Name (acronym)**
Trend in the use of modern contraception in sub-Saharan Africa: Does women's education matter?	2014	Emina et al.	http://dx.doi.org/10.1016/j.contraception.2014.02.001		No			No			No	

1. BIBLIOGRAPHIC SOURCE												
**Title**	**Year**	**First author**	**UHL**	**Measure**	**Race, ethnicity, culture, or language**	**Name (acronym)**	**Measure**	**Occupation**	**Name (acronym)**	**Measure**	**Gender or sex**	**Name (acronym)**
Utilization, Predictors and Gaps in the Continuum of Care for Maternal and Newborn Health in Ghana	2021	Enos et al.	https://dx.doi.org/10.21106/ijma.425	Urban/Rural	No			No			No	
ART use and associated factors among HIV positive caregivers of orphans and vulnerable children in Tanzania	2020	Exavery et al.	https://doi.org/10.1186/s12889-020-09361-6	Urban/Rural	No			No			Yes	Gender
Trends and causes of socioeconomic inequalities in maternal healthcare in Ghana, 2003-2014	2019	Fenny et al.	https://doi.org/10.1108/ijse-03-2018-0148	Urban/Rural	No			Yes	Labor market status	Unemployed/Employed-family or other/Self employed	No	

1. BIBLIOGRAPHIC SOURCE												
**Title**	**Year**	**First author**	**UHL**	**Measure**	**Race, ethnicity, culture, or language**	**Name (acronym)**	**Measure**	**Occupation**	**Name (acronym)**	**Measure**	**Gender or sex**	**Name (acronym)**
Determination of the predictive factors of long-lasting insecticide-treated net ownership and utilisation in the Bamenda Health District of Cameroon	2017	Fokam et al.	https://doi.org/10.1186/s12889-017-4155-5		No			No			No	
The free caesareans policy in low-income settings: An interrupted time series analysis in Mali (2003-2012)	2014	Fournier et al.	https://doi.org/10.1371/journal.pone.0105130	Cities with district hospital/Villages with health centre/Villages with no health care facility	No			No			No	

1. BIBLIOGRAPHIC SOURCE												
**Title**	**Year**	**First author**	**UHL**	**Measure**	**Race, ethnicity, culture, or language**	**Name (acronym)**	**Measure**	**Occupation**	**Name (acronym)**	**Measure**	**Gender or sex**	**Name (acronym)**
DO BETTER INSTITUTIONS BROADEN ACCESS TO SANITATION IN SUB-SAHARA AFRICA?	2021	Francois et al.	https://doi.org/10.1111/coep.12512	Urban/Rural	No			No			No	

1. BIBLIOGRAPHIC SOURCE													
Title	Year	First author	UHL	Measure	Race, ethnicity, culture, or language	Name (acronym)	Measure	Occupation	Name (acronym)	Measure	Gender or sex	Name (acronym)
Ethnic disparities in utilisation of maternal health care services in Ghana: evidence from the 2007 Ghana Maternal Health Survey	2016	Ganle	https://doi.org/10.1080/13557858.2015.1015499		Yes	Ethnicity	Akan/Ewe/Ga-Dangme/Gruma/Grusi/Guan/Hausa/Mole-Dagbani/Other	No			No	
Risky sexual behaviour and contraceptive use in contexts of displacement: Insights from a cross-sectional survey of female adolescent refugees in Ghana	2019	Ganle et al.	https://doi.org/10.1186/s12939-019-1031-1		No			No			No	
Understanding how distance to facility and quality of care affect maternal health service utilization in Kenya and Haiti: A comparative geographic information system study	2019	Gao & Kelley	https://doi.org/10.4081/gh.2019.690		No			Yes	Respondent's current employment status	Unclear	No	

1. BIBLIOGRAPHIC SOURCE												
**Title**	**Year**	**First author**	**UHL**	**Measure**	**Race, ethnicity, culture, or language**	**Name (acronym)**	**Measure**	**Occupation**	**Name (acronym)**	**Measure**	**Gender or sex**	**Name (acronym)**
Gender differences in the use of insecticide-treated nets after a universal free distribution campaign in Kano State, Nigeria: Post-campaign survey results	2013	Garley et al.	https://doi.org/10.1186/1475-2875-12-119	Urban/Rural	No			No			No	
Demand satisfied by modern contraceptive among married women of reproductive age in Kenya	2021	Gichangi et al.	https://doi.org/10.1371/journal.pone.0248393	Urban/Rural; Counties in Kenya	No			No			No	
LLIN Evaluation in Uganda Project (LLINEUP): Factors associated with ownership and use of long-lasting insecticidal nets in Uganda: A cross-sectional survey of 48 districts ISRCTN17516395 [illegible]	2018	Gonahasa et al.	https://doi.org/10.1186/s12936-018-2571-3	Region	No			No			Yes	Gender of resident who slept under LLN

1. BIBLIOGRAPHIC SOURCE												
**Title**	**Year**	**First author**	**UHL**	**Measure**	**Race, ethnicity, culture, or language**	**Name (acronym)**	**Measure**	**Occupation**	**Name (acronym)**	**Measure**	**Gender or sex**	**Name (acronym)**
ART adherence and viral suppression are high among most non-pregnant individuals with early-stage, asymptomatic HIV infection: an observational study from Uganda and South Africa	2019	Haberer et al.	https://doi.org/10.1002/jia2.25232		No			Yes	Employed	Yes/No	Yes	Gender
Low immunization coverage in Wonago district, southern Ethiopia: A community-based cross-sectional study	2019	Hailu et al.	https://doi.org/10.1371/journal.pone.0220144		No			Yes	Occupation	Housewife/ Merchant/ Other	No	

1. BIBLIOGRAPHIC SOURCE												
**Title**	**Year**	**First author**	**UHL**	**Measure**	**Race, ethnicity, culture, or language**	**Name (acronym)**	**Measure**	**Occupation**	**Name (acronym)**	**Measure**	**Gender or sex**	**Name (acronym)**
Towards universal health coverage: The role of within-country wealth-related inequality in 28 countries in sub-Saharan Africa	2011	Hosseinpoor et al.	https://doi.org/10.2471/BLT.11.087536		No			No			No	

1. BIBLIOGRAPHIC SOURCE												
**Title**	**Year**	**First author**	**UHL**	**Measure**	**Race, ethnicity, culture, or language**	**Name (acronym)**	**Measure**	**Occupation**	**Name (acronym)**	**Measure**	**Gender or sex**	**Name (acronym)**
Patterns and trends of contraceptive use among sexually active adolescents in Burkina Faso, Ethiopia, and Nigeria: evidence from cross-sectional studies	2015	Hounton et al.	https://doi.org/10.3402/gha.v8.29737	Urban/Rural	No			No			No	
Towards universal health coverage for reproductive health services in Ethiopia: two policy recommendations	2015	Onarheim et al.	https://doi.org/10.1186/s12939-015-0218-3	1) Rural, Urban; 2) Tigray, Affar, Amhara, Oromiya, Somali, Benishangul-Gumuz, SNNPR, Gambela, Harari, Addis Ababa, [illegible]	No			Yes	Employment status	Employed, Not employed	Yes	Sex of household head

1. BIBLIOGRAPHIC SOURCE												
Title	Year	First author	UHL	Measure	Race, ethnicity, culture, or language	Name (acronym)	Measure	Occupation	Name (acronym)	Measure	Gender or sex	Name (acronym)
Modern Contraception: Uptake and Correlates among Women of Reproductive Age-Group in a Rural Community of Osun State, Nigeria	2020	Idowu et al.	https://doi.org/10.4314/ejhs.v30i4.8		No			No			No	
Determinants of geographical inequalities for DTP3 vaccine coverage in sub-Saharan Africa	2020	Ikilezi et al.	https://doi.org/10.1016/j.vaccine.2020.03.005	% rural residence at mid-year	No			No			No	
Influence of women's decision-making autonomy on antenatal care utilisation and institutional delivery services in Nigeria: evidence from the Nigeria Demographic and Health Survey 2018	2022	Imo	https://doi.org/10.1186/s12884-022-04478-5	Urban/Rural; Regions in Nigeria	No			Yes	Employment	Current working/Not working	No	

1. BIBLIOGRAPHIC SOURCE												
Title	Year	First author	UHL	Measure	Race, ethnicity, culture, or language	Name (acronym)	Measure	Occupation	Name (acronym)	Measure	Gender or sex	Name (acronym)
Individual and community-level determinants of cervical cancer screening in Zimbabwe: a multi-level analyses of a nationwide survey	2022	Isabirye et al.	https://doi.org/10.1186/s12905-022-01881-0	Urban/Rural	No			Yes	Employment status	Yes/No	No	
Two decades of maternity care fee exemption policies in Ghana: have they benefited the poor?	2016	Johnson et al.	https://doi.org/10.1093/heapol/czv017	Urban/Rural; Regions in Ghana	No			No			No	

1. BIBLIOGRAPHIC SOURCE												
**Title**	**Year**	**First author**	**UHL**	**Measure**	**Race, ethnicity, culture, or language**	**Name (acronym)**	**Measure**	**Occupation**	**Name (acronym)**	**Measure**	**Gender or sex**	**Name (acronym)**
Socioeconomic inequalities in access to skilled birth attendance among urban and rural women in low-income and middle-income countries	2018	Joseph et al.	https://doi.org/10.1136/bmjgh-2018-000898	Urban/Rural	No			No			No	

1. BIBLIOGRAPHIC SOURCE												
**Title**	**Year**	**First author**	**UHL**	**Measure**	**Race, ethnicity, culture, or language**	**Name (acronym)**	**Measure**	**Occupation**	**Name (acronym)**	**Measure**	**Gender or sex**	**Name (acronym)**
Investigating the disparities in cervical cancer screening among Namibian women	2015	Kangmennaang et al.	https://doi.org/10.1016/j.ygyno.2015.05.036	Urban/Rural	No			Yes	Employment status	No employment/Fully employed/Seasonal/Occasional	No	
Contraceptive use and needs among adolescent women aged 15-19: Regional and global estimates and projections from 1990 to 2030 from a Bayesian hierarchical modelling study	2021	Kantorová et al.	https://doi.org/10.1371/journal.pone.0247479		No			No			No	
Changes in equity of maternal, newborn, and child health care practices in 115 districts of rural Ethiopia: Implications for the health extension program	2015	Karim et al.	https://doi.org/10.1186/s12884-015-0668-z	Distance to health facility	No			No			No	

1. BIBLIOGRAPHIC SOURCE												
**Title**	**Year**	**First author**	**UHL**	**Measure**	**Race, ethnicity, culture, or language**	**Name (acronym)**	**Measure**	**Occupation**	**Name (acronym)**	**Measure**	**Gender or sex**	**Name (acronym)**
Contraceptive dynamics during COVID-19 in sub-Saharan Africa: Longitudinal evidence from Burkina Faso and Kenya	2021	Karp et al.	https://doi.org/10.1136/bmjsrh-2020-200944	1) rural, urban; 2) Kenya, Burkina Faso	No			No			No	
Long-lasting insecticidal net source, ownership and use in the context of universal coverage: A household survey in eastern Rwanda	2015	Kateera et al.	https://doi.org/10.1186/s12936-015-0915-9		No			No			Yes	Sex of heald of household
A quasi-experimental evaluation of an interpersonal communication intervention to increase insecticide-treated net use among children in Zambia	2012	Keating et al.	https://doi.org/10.1186/1475-2875-11-313		No			No			Yes	Sex

1. BIBLIOGRAPHIC SOURCE												
**Title**	**Year**	**First author**	**UHL**	**Measure**	**Race, ethnicity, culture, or language**	**Name (acronym)**	**Measure**	**Occupation**	**Name (acronym)**	**Measure**	**Gender or sex**	**Name (acronym)**
Assessment of Inequalities in Coverage of Essential Reproductive, Maternal, Newborn, Child, and Adolescent Health Interventions in Kenya	2018	Keats et al.	https://doi.org/10.1001/jamanetworkopen.2018.5152	1) Urban, rural ; 2) 8 regions (central, coast, eastern, Nairobi, north eastern, Nyanza, Rift Valley, western)	No			No			No	
Charting health system reconstruction in post-war Liberia: a comparison of rural vs. remote healthcare utilization	2016	Kentoffioet al.	https://doi.org/10.1186/s12913-016-1709-7	Konobo District (remote region), Liberian rural	No			No			No	

1. BIBLIOGRAPHIC SOURCE												
**Title**	**Year**	**First author**	**UHL**	**Measure**	**Race, ethnicity, culture, or language**	**Name (acronym)**	**Measure**	**Occupation**	**Name (acronym)**	**Measure**	**Gender or sex**	**Name (acronym)**
A Transparent Universal Health Coverage Index with Decomposition by Socioeconomic Groups: Application in Asian and African Settings	2019	Khan et al.	https://doi.org/10.1007/s40258-019-00464-9	Burkina Faso, Chad, Comoros, Cote D'Ivoire, Ethiopia, Ghana, Kenya, Malawi, Mali, Morocco, Namibia, Congo Rep, Senegal, Zambia, Zimbabwe	No			No			No	
Effect of user preferences on ITN use: a review of literature and data	2017	Koenker & Yukich	https://doi.org/10.1186/s12936-017-1879-8	1) Urban, Rural; 2) Burkina Faso, Gambia, Malawi, Mali, Nigeria, Rwanda, Senegal	No			No			Yes	Household head is female

1. BIBLIOGRAPHIC SOURCE												
Title	Year	First author	UHL	Measure	Race, ethnicity, culture, or language	Name (acronym)	Measure	Occupation	Name (acronym)	Measure	Gender or sex	Name (acronym)
Insecticide-treated net use before and after mass distribution in a fishing community along Lake Victoria, Kenya: successes and unavoidable pitfalls	2014	Larson et al.	http://www.malariajournal.com/content/13/1/466		No			Yes	1) Husband Occupation; 2) Wife Occupation	1-2) Farming, Fishing, Merchant, Teacher, None, Other	Yes	Gender
Lifetime Prevalence of Cervical Cancer Screening in 55 Low- and Middle-Income Countries	2020	Lemp et al.	https://doi.org/10.1001/jama.2020.16244	Urban/Rural	No			No			No	

1. BIBLIOGRAPHIC SOURCE													
**Title**	**Year**	**First author**	**UHL**	**Measure**	**Race, ethnicity, culture, or language**	**Name (acronym)**	**Measure**	**Occupation**	**Name (acronym)**	**Measure**	**Gender or sex**	**Name (acronym)**	
Financial accessibility and user fee reforms for maternal healthcare in five sub-Saharan countries: a quasi-experimental analysis	2016	Leone et al.	https://dx.doi.org/10.1136/bmjopen-2015-009692	Urban/Rural	No			No			No		
Antiretroviral treatment coverage in a rural district in Tanzania--a modeling study using empirical data	2015	Levira et al.	https://doi.org/10.1186/s12889-015-1460-8		No			No			Yes	Gender	
The extent of universal health coverage for maternal health services in eastern uganda: A cross sectional study	2021	Lindberg et al.	https://dx.doi.org/10.1007/s10995-021-03357-3	Peri-urban/Rural	No			No			No		

1. BIBLIOGRAPHIC SOURCE												
**Title**	**Year**	**First author**	**UHL**	**Measure**	**Race, ethnicity, culture, or language**	**Name (acronym)**	**Measure**	**Occupation**	**Name (acronym)**	**Measure**	**Gender or sex**	**Name (acronym)**
Freely distributed bed-net use among Chano Mille residents, south Ethiopia: A longitudinal study	2013	Loha et al.	https://doi.org/10.1186/1475-2875-12-23		No			No			Yes	Gender
Determinants of hanging and use of ITNs in the context of near universal coverage in Zambia	2012	Macintyre et al.	https://doi.org/10.1093/heapol/czr042		No			No			Yes	Child sex

1. BIBLIOGRAPHIC SOURCE												
**Title**	**Year**	**First author**	**UHL**	**Measure**	**Race, ethnicity, culture, or language**	**Name (acronym)**	**Measure**	**Occupation**	**Name (acronym)**	**Measure**	**Gender or sex**	**Name (acronym)**
Determinants and Consequences of Failure of Linkage to Antiretroviral Therapy at Primary Care Level in Blantyre, Malawi: A Prospective Cohort Study	2012	MacPherson et al.	https://doi.org/10.1371/journal.pone.0044794		No			Yes	Employment status	Not in formal employment, In formal employment	Yes	Sex and pregnancy
Disability and sexual and reproductive health service utilisation in Uganda: an intersectional analysis of demographic and health surveys between 2006 and 2016	2022	Mac-Seing et al.	https://doi.org/10.1186/s12889-022-12708-w	1) Rural, Urban; 2) Kampala, North, Central, Central, East Central, Eastern, West Nile, Western, Southeast	No			No			Yes	Sex

1. BIBLIOGRAPHIC SOURCE													
**Title**	**Year**	**First author**	**UHL**	**Measure**	**Race, ethnicity, culture, or language**	**Name (acronym)**	**Measure**	**Occupation**	**Name (acronym)**	**Measure**	**Gender or sex**	**Name (acronym)**
Insight into Nigeria's progress towards the universal coverage of reproductive, maternal, newborn and child health services: a secondary data analysis	2022	Mafiana et al.	http://dx.doi.org/10.1136/bmjopen-2022-061595	Rural, Urban	No			No			No	
Implementation of Urban Health Equity Assessment and Response Tool: a Case of Matsapha, Swaziland	2018	Makadzange et al.	https://doi.org/10.1007/s11524-018-0241-y	Urban, Peri-Urban	No			No			No	
Effects of door-to-door hang-up visits on the use of long-lasting insecticide-treated mosquito nets in the democratic republic of the congo: A cluster randomized controlled trial	2021	Mankadi and Jin	https://doi.org/10.3390/ijerph18179048		No			No			Yes	Sex

1. BIBLIOGRAPHIC SOURCE												
**Title**	**Year**	**First author**	**UHL**	**Measure**	**Race, ethnicity, culture, or language**	**Name (acronym)**	**Measure**	**Occupation**	**Name (acronym)**	**Measure**	**Gender or sex**	**Name (acronym)**
Effect of bed net colour and shape preferences on bed net usage: a secondary data analysis of the 2017 Malawi Malaria Indicator Survey	2020	Mategula et al.	https://doi.org/10.1186/s12936-020-03499-9	1) Rural, Urban; 2) Northern, Central, South	No			No			Yes	Gender of household head
HIV care coverage among HIV-positive adolescent girls and young women in South Africa: Results from the HERStory Study	2021	Mathews et al.	https://doi.org/10.7196/SAMJ.2021.v111i5.15351		No			No			No	
Facilitators and barriers to retention in care under universal antiretroviral therapy (Option B+) for the Prevention of Mother to Child Transmission of HIV (PMTCT): A narrative review	2021	Mbeya Munkhondya et al.	https://doi.org/10.1016/j.ijans.2021.100372		No			No			No	

1. BIBLIOGRAPHIC SOURCE												
**Title**	**Year**	**First author**	**UHL**	**Measure**	**Race, ethnicity, culture, or language**	**Name (acronym)**	**Measure**	**Occupation**	**Name (acronym)**	**Measure**	**Gender or sex**	**Name (acronym)**
The consequences of declining population access to insecticide-treated nets (ITNs) on net use patterns and physical degradation of nets after 22 months of ownership	2021	Mboma et al.	https://doi.org/10.1186/s12936-021-03686-2	Bagamoyo (R), Kinondoni (U), Kilosa (R), Iringa (U), Mbozi (R), Kahama (R), Geita (R), Musoma (R)	No			No			No	
Mosquito net coverage in years between mass distributions: a case study of Tanzania, 2013	2018	Mboma et al.	https://doi.org/10.1186/s12936-018-2247-z	Bagamoyo (R), Kinondoni (U), Kilosa (R), Iringa (U), Mbozi (R), Kahama (R), Geita (R), Musoma (R)	No			No			No	
Factors associated with contraceptive use in Tigray, North Ethiopia	2017	Medhanyie et al.	https://doi.org/10.1186/s12978-017-0281-x	1) Rural, Urban; 2) Western, North West, Central, Eastern, South East, Mekelle, Southern	No			Yes	Occupation status of women	Housewife, Others (farmer, employee, merchant and student)	No	

1. BIBLIOGRAPHIC SOURCE												
**Title**	**Year**	**First author**	**UHL**	**Measure**	**Race, ethnicity, culture, or language**	**Name (acronym)**	**Measure**	**Occupation**	**Name (acronym)**	**Measure**	**Gender or sex**	**Name (acronym)**
Adolescent sexual and reproductive health in sub-Saharan Africa: who is left behind?	2020	Melesse et al.	https://doi.org/10.1136/bmjgh-2019-002231	1) Rural, Urban; 2) Eastern and Souther, West and Central Africa; 3) 33 countries in sub-Saharan Africa (14 countries in Eastern and Southern and 19 countries in West and Central Africa)	No			No			Yes	Gender
Sub-national levels and trends in contraceptive prevalence, unmet need, and demand for family planning in Nigeria with survey uncertainty	2019	Mercer et al.	https://doi.org/10.1186/s12889-019-8043-z	Not reported	No			No			No	

1. BIBLIOGRAPHIC SOURCE												
**Title**	**Year**	**First author**	**UHL**	**Measure**	**Race, ethnicity, culture, or language**	**Name (acronym)**	**Measure**	**Occupation**	**Name (acronym)**	**Measure**	**Gender or sex**	**Name (acronym)**
Exploring inequities in skilled care at birth among migrant population in a metropolitan city Addis Ababa, Ethiopia; A qualitative study	2014	Mirkuzie	http://www.equityhealthj.com/content/13/1/110		No			No			No	
Factors associated with the use of mosquito bed nets: results from two cross-sectional household surveys in Zambezia Province, Mozambique	2016	Moon et al.	https://doi.org/10.1186/s12936-016-1250-5	Alto Molócuè, Morrumbala, Namacurra	Yes	Primary language of household	Cinyanja, Cisena, Echuabo, Elomwe, Emakhuwa, Nharinga, Portuguese	No			No	
Sociocultural and Institutional Constraints to Family Planning Uptake Among Migrant Female Head Porters in Madina, a Suburb of Accra, Ghana	2021	Munemo et al.	https://doi.org/10.1177/0886109920954419		No			No			No	

1. BIBLIOGRAPHIC SOURCE													
**Title**	**Year**	**First author**	**UHL**	**Measure**	**Race, ethnicity, culture, or language**	**Name (acronym)**	**Measure**	**Occupation**	**Name (acronym)**	**Measure**	**Gender or sex**	**Name (acronym)**	
Reframing non-communicable diseases and injuries for equity in the era of universal health coverage: Findings and recommendations from the Kenya NCDI poverty commission	2021	Mwangi et al.	https://doi.org/10.5334/aogh.3085	Rural, Urban	No			No			No		

1. BIBLIOGRAPHIC SOURCE												
**Title**	**Year**	**First author**	**UHL**	**Measure**	**Race, ethnicity, culture, or language**	**Name (acronym)**	**Measure**	**Occupation**	**Name (acronym)**	**Measure**	**Gender or sex**	**Name (acronym)**
Inequities and their determinants in coverage of maternal health services in Burkina Faso	2018	Mwase et al.	https://doi.org/10.1186/s12939-018-0770-8	Boucle du Mouhoun (Boromo, Nouna, Solenzo, Toma), Centre-Sud (Manga), Centre-Est (Ouargaye, Tenkodogo, Zabré), Centre-Nord (Barsalogho, Kongoussi, Plateau Central, Ziniaré, Boussé), Centre-Ouest (Koudougou, Nanoro, Réo, Sapouy), Nord (Gourcy, Ouahigouya, Yako), Sud-Ouest (Batié	No			No			No	

1. BIBLIOGRAPHIC SOURCE													
**Title**	**Year**	**First author**	**UHL**	**Measure**	**Race, ethnicity, culture, or language**	**Name (acronym)**	**Measure**	**Occupation**	**Name (acronym)**	**Measure**	**Gender or sex**	**Name (acronym)**
A Cross-Sectional Study on Hypertension Medication Adherence in a High-Burden Region in Namibia: Exploring Hypertension Interventions and Validation of the Namibia Hill-Bone Compliance Scale	2022	Nakwafila et al.	https://doi.org/10.3390/ijerph19074416		No			Yes	Employment	None/Student, Employed, Self-employed, Retired	Yes	Sex
Assessing Adherence to Antihypertensive Therapy in Primary Health Care in Namibia: Findings and Implications	2017	Nashilongo et al.	https://doi.org/10.1007/s10557-017-6756-8		No			Yes	Employment status	Employed,Unemployed	Yes	Patient's sex (years)

1. BIBLIOGRAPHIC SOURCE												
**Title**	**Year**	**First author**	**UHL**	**Measure**	**Race, ethnicity, culture, or language**	**Name (acronym)**	**Measure**	**Occupation**	**Name (acronym)**	**Measure**	**Gender or sex**	**Name (acronym)**
Incomplete vaccination and associated factors among children aged 12–23 months in South Africa: an analysis of the South African demographic and health survey 2016	2021	Ndwandwe et al.	https://doi.org/10.1080/21645515.2020.1791509	1) Rural, Urban; 2) Eastern Cape, Free State, Gauteng, KwaZulu-Natal, Limpopo, Mpumalanga, Northern Cape, North West, Western Cape	No			Yes	Maternal occupation	Working, Not working	Yes	Sex of child
Determining the effective coverage of maternal and child health services in Kenya, using demographic and health survey data sets: tracking progress towards universal health coverage	2017	Nguhiu et al.	https://doi.org/10.1111/tmi.12841		No			No			No	

1. BIBLIOGRAPHIC SOURCE												
**Title**	**Year**	**First author**	**UHL**	**Measure**	**Race, ethnicity, culture, or language**	**Name (acronym)**	**Measure**	**Occupation**	**Name (acronym)**	**Measure**	**Gender or sex**	**Name (acronym)**
Coverage and usage of insecticide treated nets (ITNs) within households: associated factors and effect on the prevalance of malaria parasitemia in the Mount Cameroon area	2019	Njumkeng et al.	https://doi.org/10.1186/s12889-019-7555-x	Ombe, Mutengene, Dibanda, Bolifamba, Muea, Tole)	No			No			Yes	Gender
Socioeconomic inequalities in maternal health care utilization in Ghana	2019	Novignon et al.	https://doi.org/10.1186/s12939-019-1043-x	1) Rural, Urban; 2) Western, Central, Greater Accra, Volta, Eastern, Ashanti, Brong Ahafo, Northern, Upper East, Upper West	No			No			No	

1. BIBLIOGRAPHIC SOURCE												
**Title**	**Year**	**First author**	**UHL**	**Measure**	**Race, ethnicity, culture, or language**	**Name (acronym)**	**Measure**	**Occupation**	**Name (acronym)**	**Measure**	**Gender or sex**	**Name (acronym)**
Why rural women do not use primary health centres for pregnancy care: Evidence from a qualitative study in Nigeria	2019	Ntoimo et al.	https://doi.org/10.1186/s12884-019-2433-1		No			No			No	
Long-lasting insecticidal net (LLIN) ownership, use and cost of implementation after a mass distribution campaign in Kasaï Occidental Province, Democratic Republic of Congo	2017	Ntuku et al.	https://doi.org/10.1186/s12936-016-1671-1		No			Yes	Occupation of the head of the household	Without occupation, Farmer, Merchant, Employed	Yes	Sex
Explaining socioeconomic disparities and gaps in the use of antenatal care services in 36 countries in sub-Saharan Africa	2021	Obse & Ataguba	https://doi.org/10.1093/heapol/czab036	1) Rural, Urban; 2) Yes, No	No			No			Yes	Proportion of women in the country

1. BIBLIOGRAPHIC SOURCE												
**Title**	**Year**	**First author**	**UHL**	**Measure**	**Race, ethnicity, culture, or language**	**Name (acronym)**	**Measure**	**Occupation**	**Name (acronym)**	**Measure**	**Gender or sex**	**Name (acronym)**
Sociodemographic factors associated with the use of insecticide treated nets among under-fives in Nigeria: Evidence from a national survey	2022	Ojo et al.	https://doi.org/10.1177/0049475522111 0374	1) Rural, Urban; 2) North East, North Central, North West, South, South South, South West	No			No			Yes	1) Child's gender; 2) Gender of head of household
Towards making efficient use of household resources for appropriate prevention of malaria: investigating households' ownership, use and expenditures on ITNs and other preventive tools in Southeast Nigeria	2014	Onwujekwe et al.	http://www.biomedcentral.com/1471-2458/14/315		No			No			No	

1. BIBLIOGRAPHIC SOURCE											
**Title**	**Year**	**First author**	**UHL**	**Measure**	**Race, ethnicity, culture, or language**	**Name (acronym)**	**Measure**	**Occupation**	**Name (acronym)**	**Measure**	**Gender or sex**	**Name (acronym)**
Demographic disparities in unimproved drinking water and sanitation in Ghana: A nationally representative cross-sectional study	2022	Oppong et al.	http://dx.doi.org/10.1136/bmjopen-2021-060595	1) Rural, Urban; 2) Household's regional location from the 10 administrative regions in Ghana (Western, Central, Greater Accra, Volta, Eastern, Ashanti, Brong Ahafo, Northern, Upper East, Upper West); 3) Coastal, Middle,	No			No			Yes	Sex of household head
Inequities in Access to Maternal Health Care in Enugu State: Implications for Universal Health Coverage to Meet Vision 2030 in Nigeria	2019	Ozumba et al.	https://doi.org/10.1177/0272684X18819977	Rural, Urban	No			Yes	Employed	Employed, Not employed	No	

1. BIBLIOGRAPHIC SOURCE													
**Title**	**Year**	**First author**	**UHL**	**Measure**	**Race, ethnicity, culture, or language**	**Name (acronym)**	**Measure**	**Occupation**	**Name (acronym)**	**Measure**	**Gender or sex**	**Name (acronym)**	
How do supply- and demand-side interventions influence equity in healthcare utilisation? Evidence from maternal healthcare in Senegal	2019	Parmar & Banerjee	https://doi.org/10.1016/j.socscimed.2019.112582	Rural, Urban	No			No			No		
Temporal and regional variations in use, equity and quality of antenatal care in Egypt: A repeat cross-sectional analysis using Demographic and Health Surveys	2019	Pugliese-Garcia et al.	https://doi.org/10.1186/s12884-019-2409-1	1) Rural, Urban; 2) Urban Governorates, Urban Lower, Urban Upper, Rural Lower, Rural Upper, Frontier Governorates	No			No			No		

1. BIBLIOGRAPHIC SOURCE												
**Title**	**Year**	**First author**	**UHL**	**Measure**	**Race, ethnicity, culture, or language**	**Name (acronym)**	**Measure**	**Occupation**	**Name (acronym)**	**Measure**	**Gender or sex**	**Name (acronym)**
Geographical Inequalities in Use of Improved Drinking Water Supply and Sanitation across Sub-Saharan Africa: Mapping and Spatial Analysis of Cross-sectional Survey Data	2014	Pullan et al.	https://doi.org/10.1371/journal.pmed.1001626	1) Rural, Urban	No			No			No	
Individual and Network Factors Associated With HIV Care Continuum Outcomes Among Nigerian MSM Accessing Health Care Services	2018	Ramadhani et al.	https://dx.doi.org/10.1097/QAI.0000000000001756		No			No			No	
Non-adherence to long-lasting insecticide treated bednet use following successful malaria control in Tororo, Uganda	2020	Rek et al.	https://doi.org/10.1371/journal.pone.0243303		No			No			Yes	Gender of the individual

1. BIBLIOGRAPHIC SOURCE												
**Title**	**Year**	**First author**	**UHL**	**Measure**	**Race, ethnicity, culture, or language**	**Name (acronym)**	**Measure**	**Occupation**	**Name (acronym)**	**Measure**	**Gender or sex**	**Name (acronym)**
Design, implementation and evaluation of a national campaign to deliver 18 million free long-lasting insecticidal nets to uncovered sleeping spaces in Tanzania	2013	Renggli et al.	https://doi.org/10.1186/1475-2875-12-85	Southern, Southern Highlands, Central, West Lake, Lake, Coastal and Northern Zones	No			No			No	
A long way to go - Estimates of combined water, sanitation and hygiene coverage for 25 sub-Saharan African countries	2017	Roche et al.	https://doi.org/10.1371/journal.pone.0171783	1) Rural, Urban	No			No			No	

1. BIBLIOGRAPHIC SOURCE												
**Title**	**Year**	**First author**	**UHL**	**Measure**	**Race, ethnicity, culture, or language**	**Name (acronym)**	**Measure**	**Occupation**	**Name (acronym)**	**Measure**	**Gender or sex**	**Name (acronym)**
Determinants of bed net use in southeast Nigeria following mass distribution of LLINs: Implications for social behavior change interventions	2015	Russell et al.	https://doi.org/10.1371/journal.pone.0139447		No			Yes	Occupation	Farmer (fisherman, animal rearer), Hand-work (self-employed), Housewife, Civil Servant, Trader (commerce/sales), Student, Other	Yes	Sex
Factors associated with the non-use of insecticide-treated nets in Rwandan children	2016	Ruyange et al.	https://doi.org/10.1186/s12936-016-1403-6	Rural, Urban	No			Yes	Mother occupation	Not employed or agricultural, Employed	Yes	Children sex

1. BIBLIOGRAPHIC SOURCE												
**Title**	**Year**	**First author**	**UHL**	**Measure**	**Race, ethnicity, culture, or language**	**Name (acronym)**	**Measure**	**Occupation**	**Name (acronym)**	**Measure**	**Gender or sex**	**Name (acronym)**
Wealth Status, Health Insurance, and Maternal Health Care Utilization in Africa: Evidence from Gabon	2020	Sanogo & Yaya	https://dx.doi.org/10.1155/2020/4036830	1) Rural, Urban; 2) Libreville-Port-Gentil, Estuaire, Haut-Ogooue, Moyen-Ogooue, Ngounie, Nyanga, Ogooue Maritime, Ogooue-Ivindo, Ogooue-Lolo, and Woleu-[illegible]	No			No			No	

1. BIBLIOGRAPHIC SOURCE											
**Title**	**Year**	**First author**	**UHL**	**Measure**	**Race, ethnicity, culture, or language**	**Name (acronym)**	**Measure**	**Occupation**	**Name (acronym)**	**Measure**	**Gender or sex**	**Name (acronym)**
Factors associated with use of insecticide-treated net for malaria prevention in Manica District, Mozambique: a community-based cross-sectional survey	2021	Scott et al.	https://doi.org/10.1186/s12936-021-03738-7		No			No			Yes	Sex
Healthcare utilisation, cancer screening and potential barriers to accessing cancer care in rural South West Nigeria: a cross-sectional study	2021	Sharma et al.	https://doi.org/10.1136/bmjopen-2020-040352		No			No			Yes	Sex

1. BIBLIOGRAPHIC SOURCE												
**Title**	**Year**	**First author**	**UHL**	**Measure**	**Race, ethnicity, culture, or language**	**Name (acronym)**	**Measure**	**Occupation**	**Name (acronym)**	**Measure**	**Gender or sex**	**Name (acronym)**
Socio-economic inequalities in ANC attendance among mothers who gave birth in the past 12 months in Debre Brehan town and surrounding rural areas, North East Ethiopia: A community-based survey	2019	Shibre & Mekonnen	https://doi.org/10.1186/s12978-019-0768-8		No			Yes	1) Maternal occupation; 2) husband's occupation	1) Housewife, government employee, private employee, merchant, daily worker/student; 2) government employee, private employee, merchant, daily worker/driv	No	

1. BIBLIOGRAPHIC SOURCE												
**Title**	**Year**	**First author**	**UHL**	**Measure**	**Race, ethnicity, culture, or language**	**Name (acronym)**	**Measure**	**Occupation**	**Name (acronym)**	**Measure**	**Gender or sex**	**Name (acronym)**
Individual and environmental characteristics associated with immunization of children in rural areas of Burkina Faso: A multi-level analysis	2007	Sia et al.	https://pubmed.ncbi.nlm.nih.gov/18299262/		No			Yes	1) Occupation de la mère [Mother's Occupation]; 2) Occupation du partenaire de la mère [Occupation of the mother's partner]	1) Ne travaille pas [Not working], Agriculture [Agriculture], Autre travail [Other work]; 2) Agriculture [Agriculture], Autre travail [Other work]	Yes	Child's sex
Inequalities in access and utilization of maternal, newborn and child health services in sub-saharan africa: A special focus on urban settings	2021	Sidze et al.	https://dx.doi.org/10.1007/s10995-021-03250-z	Countries	No			Yes	Employment status		No	

1. BIBLIOGRAPHIC SOURCE												
**Title**	**Year**	**First author**	**UHL**	**Measure**	**Race, ethnicity, culture, or language**	**Name (acronym)**	**Measure**	**Occupation**	**Name (acronym)**	**Measure**	**Gender or sex**	**Name (acronym)**
Insecticide-treated bed net access and use among preschool children in Nouna District, Burkina Faso	2020	Sié et al.	https://dx.doi.org/10.1093/inthealth/ihaa003		Yes	Ethnicity	Dafing/Marka, Bwaba, Mossi, Samo, Peulh, Other	No			Yes	Child’s sex
Evidence of improving antiretroviral therapy treatment delays: an analysis of eight years of programmatic outcomes in Blantyre, Malawi	2013	Sloan et al.	http://www.biomedcentral.com/1471-2458/13/490		No			No			Yes	Sex
Low use of long-lasting insecticidal nets for malaria prevention in south-central Ethiopia: A community-based cohort study	2019	Solomon et al.	https://doi.org/10.1371/journal.pone.0210578		No			Yes	Occupational status of head of household	Farmer, Other	Yes	Gender

1. BIBLIOGRAPHIC SOURCE													
**Title**	**Year**	**First author**	**UHL**	**Measure**	**Race, ethnicity, culture, or language**	**Name (acronym)**	**Measure**	**Occupation**	**Name (acronym)**	**Measure**	**Gender or sex**	**Name (acronym)**
Empowerment and use of modern contraceptive methods among married women in Burkina Faso: a multilevel analysis	2021	Some et al.	https://dx.doi.org/10.1186/s12889-021-11541-x	Rural, Urban	No			No			No	
Evaluation of the 2011 long-lasting, insecticide-treated net distribution for universal coverage in Togo	2013	Stevens et al.	http://www.malariajournal.com/content/12/1/162	Maritime, Plateaux, Centrale, Kara, Savanes	No			No			Yes	Sex
On the way to universal coverage of maternal services in Iringa rural District in Tanzania. Who is yet to be reached?	2016	Straneo et al.	https://doi.org/10.4314/ahs.v16i2.10		No			No			Yes	Sex of household head

1. BIBLIOGRAPHIC SOURCE												
**Title**	**Year**	**First author**	**UHL**	**Measure**	**Race, ethnicity, culture, or language**	**Name (acronym)**	**Measure**	**Occupation**	**Name (acronym)**	**Measure**	**Gender or sex**	**Name (acronym)**
Evaluation of long-lasting insecticidal net distribution through schools in Southern Tanzania	2022	Stuck et al.	https://doi.org/10.1093/heapol/czab140	Intervention districts (Lindi, Mtwara), non-intervention districts (Geita, Mwanza)	No			No			No	
Not all inequalities are equal: differences in coverage across the continuum of reproductive health services	2019	Sully et al.	https://dx.doi.org/10.1136/bmjgh-2019-001695	1) Rural, Urban; 2) Eastern Africa, Middle Africa, Western Africa, Northern Africa	No			No			No	

1. BIBLIOGRAPHIC SOURCE												
**Title**	**Year**	**First author**	**UHL**	**Measure**	**Race, ethnicity, culture, or language**	**Name (acronym)**	**Measure**	**Occupation**	**Name (acronym)**	**Measure**	**Gender or sex**	**Name (acronym)**
Determinants of long-lasting insecticidal net ownership and utilization in malaria transmission regions: Evidence from Zimbabwe Demographic and Health Surveys	2019	Tapera	https://doi.org/10.1186/s12936-019-2912-x	1) Rural, Urban; 2) Bulawayo, Harare, Manicaland, Mashonaland Central, Mashonaland East, Mashonaland West, Masvingo, Matebeleland North, Matebeleland South, [illegible]	No			No			Yes	Sex of head of household
Sociodemographic inequities in cervical cancer screening, treatment and care amongst women aged at least 25 years: evidence from surveys in Harare, Zimbabwe	2019	Tapera et al.	https://doi.org/10.1186/s12889-019-6749-6	Manicaland, Masvingo, Midlands, Mashonaland Central, Mashonaland East, Mashonaland West, Harare	No			Yes	Occupation	Unemployed, professional, selfemployed, other	No	

1. BIBLIOGRAPHIC SOURCE												
**Title**	**Year**	**First author**	**UHL**	**Measure**	**Race, ethnicity, culture, or language**	**Name (acronym)**	**Measure**	**Occupation**	**Name (acronym)**	**Measure**	**Gender or sex**	**Name (acronym)**
Factors associated to bed net use in Cameroon: a retrospective study in Mfou health district in the Centre Region	2012	Tchinda et al.	http://www.panafrican-med-journal.com/content/article/12/112/full/	Rural, Urban	No			Yes	Main occupation	Students/unemployed/Farmers/Employed	Yes	Sex
Cervical cancer screening uptake and correlates among HIV-infected women: a cross-sectional survey in Cote d'Ivoire, West Africa	2019	Tchounga et al.	https://dx.doi.org/10.1136/bmjopen-2019-029882		No			No			No	

1. BIBLIOGRAPHIC SOURCE												
**Title**	**Year**	**First author**	**UHL**	**Measure**	**Race, ethnicity, culture, or language**	**Name (acronym)**	**Measure**	**Occupation**	**Name (acronym)**	**Measure**	**Gender or sex**	**Name (acronym)**
Duration and determinants of delayed tuberculosis diagnosis and treatment in high-burden countries: a mixed-methods systematic review and meta-analysis	2021	Teo et al.	https://doi.org/10.1186/s12931-021-01841-6	Urban, sub-urban, in areas without health centers	No			Yes	Occupation	More working days per week, unemployed	Yes	Sex
Skilled delivery inequality in Ethiopia: To what extent are the poorest and uneducated mothers benefiting?	2017	Tesfaye et al.	https://doi.org/10.1186/s12939-017-0579-x		No			No			No	

1. BIBLIOGRAPHIC SOURCE												
**Title**	**Year**	**First author**	**UHL**	**Measure**	**Race, ethnicity, culture, or language**	**Name (acronym)**	**Measure**	**Occupation**	**Name (acronym)**	**Measure**	**Gender or sex**	**Name (acronym)**
Investigating the association between pregnancy intention and insecticide-treated bed net (ITN) use: A cross-sectional study of pregnant women in Rwanda	2015	Thogarapalli et al.	https://doi.org/10.1007/s10389-015-0676-5	Rural, Urban	No			No			No	
Success of Senegal's first nationwide distribution of long-lasting insecticide-treated nets to children under five - Contribution toward universal coverage	2011	Thwing et al.	http://www.malariajournal.com/content/10/1/86		No			No			No	
Declines in Malaria Burden and all-cause child mortality following increases in control interventions in Senegal, 2005-2010	2017	Thwing et al.	https://doi.org/10.4269/ajtmh.16-0953	Dakar, North, Center, South	No			No			No	

1. BIBLIOGRAPHIC SOURCE												
**Title**	**Year**	**First author**	**UHL**	**Measure**	**Race, ethnicity, culture, or language**	**Name (acronym)**	**Measure**	**Occupation**	**Name (acronym)**	**Measure**	**Gender or sex**	**Name (acronym)**
Individual-level and community-level determinants of cervical cancer screening among Kenyan women: a multilevel analysis of a Nationwide survey	2017	Tiruneh et al.	https://doi.org/10.1186/s12905-017-0469-9	Urban, Rural	No			Yes	Occupation	Yes, No	No	
Evaluation of the coverage and effective use rate of long-lasting insecticidal nets after nation-wide scale up of their distribution in Benin	2013	Tokponnon et al.	https://doi.org/10.1186/1756-3305-6-265	Urban, Rural	No			No			Yes	Gender of the head of the family
Factors associated with the upsurge in the use of delivery care services in Sierra Leone	2020	Tsawe & Susuman	https://doi.org/10.1016/j.puhe.2019.11.002	Urban, Rural	No			No			No	

1. BIBLIOGRAPHIC SOURCE												
**Title**	**Year**	**First author**	**UHL**	**Measure**	**Race, ethnicity, culture, or language**	**Name (acronym)**	**Measure**	**Occupation**	**Name (acronym)**	**Measure**	**Gender or sex**	**Name (acronym)**
Which family members use the best nets? An analysis of the condition of mosquito nets and their distribution within households in Tanzania	2010	Tsuang et al.	http://www.malariajournal.com/content/9/1/211		No			No			Yes	Gender
Utilization of insecticide treated nets among pregnant women in enugu, South Eastern Nigeria	2013	Ugwu et al.	https://pubmed.ncbi.nlm.nih.gov/23771448/		No			No			No	

1. BIBLIOGRAPHIC SOURCE												
**Title**	**Year**	**First author**	**UHL**	**Measure**	**Race, ethnicity, culture, or language**	**Name (acronym)**	**Measure**	**Occupation**	**Name (acronym)**	**Measure**	**Gender or sex**	**Name (acronym)**
Finding the gap: Revealing local disparities in coverage of maternal, newborn and child health services in South Sudan using lot quality assurance sampling	2015	Valadez et al.	https://doi.org/10.1111/tmi.12613	UN, Upper Nile; Jong, Jonglei; Unity, Unity; Warr, Warrap; NBeG, Northern Bahr el Ghazal; WBeG, Western Bahr el Ghazal; Lakes, Lakes; WES, Western Equatoria State; CES, Central Equatoria State; EES, Eastern Equatoria	No			No			No	
Group Medical Visit and Microfinance Intervention for Patients With Diabetes or Hypertension in Kenya	2021	Vedanthan et al.	https://doi.org/10.1016/j.jacc.2021.03.002		No			Yes	Baseline monthly earnings	No job - <1,000, kshs - 1,000-4,999 - Kshs >=5,000	Yes	Gender

1. BIBLIOGRAPHIC SOURCE												
**Title**	**Year**	**First author**	**UHL**	**Measure**	**Race, ethnicity, culture, or language**	**Name (acronym)**	**Measure**	**Occupation**	**Name (acronym)**	**Measure**	**Gender or sex**	**Name (acronym)**
Equity in Maternal Health in South Africa: Analysis of Health Service Access and Health Status in a National Household Survey	2013	Wabiri et al.	https://doi.org/10.1371/journal.pone.0073864	Urban formal, urban informal, rural formal, urban informal	Yes	Race	African, White, Mixed ancestry, indian	Yes	Employment	Housewife or homemaker, unemployed/seeking work, unemployed/not seeking work, informal sector/self employed, student/learner	No	
Persisting Regional Disparities in Modern Contraceptive Use and Unmet Need for Contraception among Nigerian Women	2019	Wang & Cao	https://doi.org/10.1155/2019/9103928	Urban/rural and north-central/north-east/north-west/south-east/south-south/south-west	No			No			No	

1. BIBLIOGRAPHIC SOURCE												
**Title**	**Year**	**First author**	**UHL**	**Measure**	**Race, ethnicity, culture, or language**	**Name (acronym)**	**Measure**	**Occupation**	**Name (acronym)**	**Measure**	**Gender or sex**	**Name (acronym)**
Effective coverage of facility delivery in Bangladesh, Haiti, Malawi, Nepal, Senegal, and Tanzania	2019	Wang et al.	https://doi.org/10.1371/journal.pone.0217853	North, Central, South (Malawi) - North, Centra, East, South, Thies (Senegal) - Lake, Nothern, Southern, Eastern, South West Highlands, [illegible]	No			No			No	
Use of long-lasting insecticide-treated bed nets in a population with universal coverage following a mass distribution campaign in Uganda	2016	Wanzira et al.	https://doi.org/10.1186/s12936-016-1360-0		No			No			No	

1. BIBLIOGRAPHIC SOURCE												
**Title**	**Year**	**First author**	**UHL**	**Measure**	**Race, ethnicity, culture, or language**	**Name (acronym)**	**Measure**	**Occupation**	**Name (acronym)**	**Measure**	**Gender or sex**	**Name (acronym)**
Long-lasting insecticide-treated bed net ownership and use among children under five years of age following a targeted distribution in central Uganda	2014	Wanzira et al.	https://doi.org/10.1186/1475-2875-13-185		No			No			No	
Evaluation of a national universal coverage campaign of long-lasting insecticidal nets in a rural district in north-west Tanzania	2012	West et al.	https://doi.org/10.1186/1475-2875-11-273		No			No			No	
The impact of renewing long-lasting insecticide-treated nets in the event of malaria resurgence: Lessons from 10 years of net use in dielmo, Senegal	2021	Wotodjo et al.	https://doi.org/10.4269/AJTMH.20-0127		No			No			No	

1. BIBLIOGRAPHIC SOURCE												
**Title**	**Year**	**First author**	**UHL**	**Measure**	**Race, ethnicity, culture, or language**	**Name (acronym)**	**Measure**	**Occupation**	**Name (acronym)**	**Measure**	**Gender or sex**	**Name (acronym)**
Wealth and Education Inequities in Maternal and Child Health Services Utilization in Rural Ethiopia	2022	Wuneh et al.	https://doi.org/10.3390/ijerph19095421		No			No			No	
Inequalities in maternal health care utilization in Benin: A population based cross-sectional study	2018	Yaya et al.	https://doi.org/10.1186/s12884-018-1846-6	1) Rural, Urban; 2) Alibori, Atacora, Atlantique, Borgou, Collines, Couffo, Donga, Littoral,Mono, Queme, Plateau, Zou	No			Yes	Currently working	Yes, No	Yes	Sex of household head

1. BIBLIOGRAPHIC SOURCE												
**Title**	**Year**	**First author**	**UHL**	**Measure**	**Race, ethnicity, culture, or language**	**Name (acronym)**	**Measure**	**Occupation**	**Name (acronym)**	**Measure**	**Gender or sex**	**Name (acronym)**
Long-lasting insecticide-treated bed net ownership, utilization and associated factors among school-age children in Dara Mallo and Uba Debretsehay districts, Southern Ethiopia	2020	Zerdo et al.	https://dx.doi.org/10.1186/s12936-020-03437-9		No			Yes	1) Occupation of household head; 2) Occupation of mother	1) Occupation of household head (Farmer/Civil servant/Merchant/Others); 2) Occupation of mother (Housewife/Employee/Others)	Yes	1) Gender of school-age children (SAC); 2) Gender of household head
Trends and projections of universal health coverage indicators in Ghana, 1995-2030: A national and subnational study	2019	Zhang et al.	https://doi.org/10.1371/journal.pone.0209126	Ashanti, Brong-Ahafo, Central, Eastern, Greater Accra, Northern, Upper East, Upper West, Volta, Western	No			No			No	

1. BIBLIOGRAPHIC SOURCE												
**Title**	**Year**	**First author**	**UHL**	**Measure**	**Race, ethnicity, culture, or language**	**Name (acronym)**	**Measure**	**Occupation**	**Name (acronym)**	**Measure**	**Gender or sex**	**Name (acronym)**
Developing Malawi's Universal Health Coverage Index	2022	Mchenga et al.	https://doi.org/10.3389/frhs.2021.786186		No			No			No	
Socioeconomic Factors Associated with Compliance with Mass Drug Administration for Lymphatic Filariasis Elimination in Kenya: Descriptive Study Results	2012	Njomo et al.	https://www.proquest.com/docview/1115911778?pq-origsite=gscholar&fromopenview=true		No			Yes	Main occupation	Peasant Farming, Business, Fishing, Casual Labor, Housewife, Others	No	

1. BIBLIOGRAPHIC SOURCE												
**Title**	**Year**	**First author**	**UHL**	**Measure**	**Race, ethnicity, culture, or language**	**Name (acronym)**	**Measure**	**Occupation**	**Name (acronym)**	**Measure**	**Gender or sex**	**Name (acronym)**
Equality analysis of main health indicators among children under 5 years in Uganda	2019	Elduma	http://dx.doi.org/10.4314/ejhs.v29i2.8	Rural, Urban	No			No			Yes	Sex

1. BIBLIOGRAPHIC SOURCE												
**Title**	**Year**	**First author**	**UHL**	**Measure**	**Religion**	**Name (acronym)**	**Measure**	**Education**	**Name (acronym)**	**Measure**	**Socioeconomic status (SES)**	**Name (acronym)**
Reproductive Plans And Utilization of Contraceptives Among Women Living With HIV	2019	Adeleye et al.	https://dx.doi.org/10.21106/ijma.277		No			Yes	Level of Education	Primary/Secondary/Tertiary/Others	No	
Provision of immediate postpartum contraception to women living with HIV in the Eastern Cape, South Africa; a cross-sectional analysis	2020	Adeniyi et al.	https://doi.org/10.1186/s12978-020-01049-9		No			Yes	Education level	Tertiary/Grade 7-12/Grade 6 or less	No	
Moving Up the Sanitation Ladder: A Study of the Coverage and Utilization of Improved Sanitation Facilities and Associated Factors Among Households in Southern Ethiopia	2022	Afework et al.	https://doi.org/10.1177/11786302221080825		No			Yes	Educational status of head	Illiterate/Literate	Yes	Wealth quintile

1. BIBLIOGRAPHIC SOURCE												
**Title**	**Year**	**First author**	**UHL**	**Measure**	**Religion**	**Name (acronym)**	**Measure**	**Education**	**Name (acronym)**	**Measure**	**Socioeconomic status (SES)**	**Name (acronym)**
Maternal health care services utilisation in the context of 'Abiye' (safe motherhood) programme in Ondo State, Nigeria	2020	Ajayi et al.	https://doi.org/10.1186/s12889-020-08512-z		Yes	Religion	Christians/ Muslims	Yes	Levels of education	Primary or less/Secondary/Higher	Yes	Socioeconomic status; Wealth quinitles
Slums, women and sanitary living in South-South Nigeria	2021	Akpabio et al.	https://doi.org/10.1007/s10901-020-09802-z	Women/Men	No			No			No	
Leaving no child behind: Decomposing socioeconomic inequalities in child health for india and south africa	2021	Alaba et al.	https://doi.org/10.3390/ijerph18137114	Male/Female	No			Yes	Mother's education, Father's education	No/Primary/Secondary/Tertiary	Yes	Wealth quintile

1. BIBLIOGRAPHIC SOURCE												
**Title**	**Year**	**First author**	**UHL**	**Measure**	**Religion**	**Name (acronym)**	**Measure**	**Education**	**Name (acronym)**	**Measure**	**Socioeconomic status (SES)**	**Name (acronym)**
Access to skilled attendant at birth and the coverage of the third dose of diphtheria-tetanus-pertussis vaccine across 14 West African countries - An equity analysis	2020	Alhassan et al.	https://doi.org/10.1186/s12939-020-01204-5		No			Yes	Mother's education	No education-Secondary education +	Yes	Wealth quintile
Trends and correlates of maternal, newborn and child health services utilization in primary healthcare facilities: An explorative ecological study using DHIMSII data from one district in the Volta region of Ghana	2020	Alhassan et al.	https://doi.org/10.1186/s12884-020-03195-1		No			No			No	

1. BIBLIOGRAPHIC SOURCE											
Title	Year	First author	UHL	Measure	Religion	Name (acronym)	Measure	Education	Name (acronym)	Measure	Socioeconomic status (SES)	Name (acronym)
Determinants of equity in utilization of maternal health services in Butajira, Southern Ethiopia	2012	Aliy & Mariam	https://www.ajol.info/index.php/ejhd/article/view/116114	Male/Female	No			Yes	Mother and father's educational status	Formal/No formal	Yes	Average annual income
Determinants of insecticide treated nets use among youth corp members in Edo State, Nigeria	2011	Amoran O. E. et al	http://www.biomedcentral.com/1471-2458/11/728	Male/Female	No			No			No	

1. BIBLIOGRAPHIC SOURCE												
**Title**	**Year**	**First author**	**UHL**	**Measure**	**Religion**	**Name (acronym)**	**Measure**	**Education**	**Name (acronym)**	**Measure**	**Socioeconomic status (SES)**	**Name (acronym)**
Towards achievement of Sustainable Development Goal 3: multilevel analyses of demographic and health survey data on health insurance coverage and maternal healthcare utilisation in sub-Saharan Africa	2022	Amu et al.	https://doi.org/10.1093/inthealth/ihac017		Yes	Religion	Chrisitianity/Islamic/African traditional/No Religio/Others	Yes	Maternal educational status	None/Primary/Secondary/Higher	Yes	Wealth quintile

1. BIBLIOGRAPHIC SOURCE												
**Title**	**Year**	**First author**	**UHL**	**Measure**	**Religion**	**Name (acronym)**	**Measure**	**Education**	**Name (acronym)**	**Measure**	**Socioeconomic status (SES)**	**Name (acronym)**
Equity and access to maternal and child health services in Ghana a cross-sectional study	2021	Anarwat et al.	https://doi.org/10.1186/s12913-021-06872-9		No			Yes	Education level	Secondary or higher/Lower education	Yes	Wealth quintile
A subnational profiling analysis reveals regional differences as the main predictor of ITN ownership and use in Nigeria	2019	Andrada et al.	https://doi.org/10.1186/s12936-019-2816-9		No			Yes	Educational level of household head	None/Primary/Secondary or higher	Yes	Wealth quintile
Inequality trends in maternal health services for young Ghanaian women with childbirth history between 2003 and 2014	2017	Asamoah & Agardh	https://doi.org/10.1136/bmjopen-2016-011663		No			Yes	Educational level of household head	None/Basic/Secondary or higher	Yes	Wealth quintile

1. BIBLIOGRAPHIC SOURCE												
**Title**	**Year**	**First author**	**UHL**	**Measure**	**Religion**	**Name (acronym)**	**Measure**	**Education**	**Name (acronym)**	**Measure**	**Socioeconomic status (SES)**	**Name (acronym)**
Leaving no one behind: Lessons from implementation of policies for universal HIV treatment to universal health coverage	2020	Assefa et al.	https://doi.org/10.1186/s12992-020-00549-4	Male/Female	No			No			No	
Inequalities in child immunization coverage in Ghana: evidence from a decomposition analysis	2018	Asuman et al.	https://doi.org/10.1186/s13561-018-0193-7	Female/Male	Yes	Religion	Christian/Moslem/No religion	Yes	Education	Years of schooling	Yes	Household wealth quintile
A reassessment of global antenatal care coverage for improving maternal health using sub-Saharan Africa as a case study	2018	Ataguba	https://dx.doi.org/10.1371/journal.pone.0204822		No			Yes	Education level of women	No formal/Primary/At least secondary	Yes	Household wealth quintile

1. BIBLIOGRAPHIC SOURCE												
**Title**	**Year**	**First author**	**UHL**	**Measure**	**Religion**	**Name (acronym)**	**Measure**	**Education**	**Name (acronym)**	**Measure**	**Socioeconomic status (SES)**	**Name (acronym)**
Socio-economic inequality in maternal health care utilization in Sub-Saharan Africa: Evidence from Togo	2021	Atake	http://dx.doi.org/10.1002/hpm.3083		Yes	Traditional/Christian/Muslim/No religion		Yes	Woman & Partner's education	Uneducated/Primary/Secondary/Higher	Yes	Wealth quintile
Poor prenatal service utilization and pregnancy outcome in a tertiary health facility in southwest Nigeria	2020	Awoleke & Olofinbiyi	https://doi.org/10.11604/pamj.2020.35.28.20426		No			Yes	Maternal education	No formal/Primary/Secondary/Tertiary	No	
Correlates of intra-household ITN use in Liberia: A multilevel analysis of household survey data	2016	Babalola et al.	http://dx.doi.org/10.1371/journal.pone.0158331	Male/Female	No			Yes	Education level of the female caregiver	None/Primary/Post-primary	Yes	Wealth index

1. BIBLIOGRAPHIC SOURCE													
**Title**	**Year**	**First author**	**UHL**	**Measure**	**Religion**	**Name (acronym)**	**Measure**	**Education**	**Name (acronym)**	**Measure**	**Socioeconomic status (SES)**	**Name (acronym)**
Prevalence and determinants of maternal healthcare utilisation among young women in sub-Saharan Africa: cross-sectional analyses of demographic and health survey data	2022	Bain et al.	https://doi.org/10.1186/s12889-022-13037-8	Male/Female	Yes	Religion	African traditional/Christian/Muslim/No religion	Yes	Woman & Partner's education	None/Primary/Secondary/Higher	Yes	Wealth index

1. BIBLIOGRAPHIC SOURCE												
**Title**	**Year**	**First author**	**UHL**	**Measure**	**Religion**	**Name (acronym)**	**Measure**	**Education**	**Name (acronym)**	**Measure**	**Socioeconomic status (SES)**	**Name (acronym)**
Factors influencing timing and frequency of antenatal care in Uganda	2011	Bbaale	https://www.ncbi.nlm.nih.gov/pmc/articles/PMC3562883/pdf/AMJ-04-431.pdf		Yes	Religion	Catholics/Protest/Muslim/Other	Yes	Woman & Partner's education	No formal/Primary/Secondary/Post-secondary	Yes	Wealth quintile
Maternal education and childbirth care in Uganda	2011	Bbaale & Guloba	https://www.ncbi.nlm.nih.gov/pmc/articles/PMC3562941/pdf/AMJ-04-389.pdf		Yes	Religion	Catholics/Protest/Muslim/Other	Yes	Woman & Partner's education	No formal/Primary/Secondary/Post-secondary	Yes	Wealth quintile

1. BIBLIOGRAPHIC SOURCE													
**Title**	**Year**	**First author**	**UHL**	**Measure**	**Religion**	**Name (acronym)**	**Measure**	**Education**	**Name (acronym)**	**Measure**	**Socioeconomic status (SES)**	**Name (acronym)**	
A quantitative analysis of food insecurity and other barriers associated with ART nonadherence among women in rural communities of Eswatini	2021	Becker et al.	https://doi.org/10.1371/journal.pone.0256277		No			Yes	Educational level	None/Primary/Secondary or Higher	Yes	Household income	
Two decades of antenatal and delivery care in Uganda: a cross-sectional study using Demographic and Health Surveys	2018	Benova et al.	https://doi.org/10.1186/s12913-018-3546-3		No			Yes	Education	No education/Primary/Secondary+	Yes	Wealth quintile	
Utilization of sexual and reproductive health services in ethiopia - Does it affect sexual activity among high school students?	2015	Bilal et al.	https://doi.org/10.1016/j.srhc.2014.09.009	Male/Female	No			Yes	Education of mother and father	illiterate/can read and write/Grade 1-8/Grade 9-12/Grade 12+	Yes	Family income	

1. BIBLIOGRAPHIC SOURCE												
**Title**	**Year**	**First author**	**UHL**	**Measure**	**Religion**	**Name (acronym)**	**Measure**	**Education**	**Name (acronym)**	**Measure**	**Socioeconomic status (SES)**	**Name (acronym)**
Addressing the huge poor–rich gap of inequalities in accessing safe childbirth care: A first step to achieving universal maternal health coverage in Tanzania	2021	Bintabara	https://doi.org/10.1371/journal.pone.0246995		No			Yes	Maternal education	None/Primary/Secondary/Higher	Yes	Wealth quintile
Twelve-year persistence of inequalities in antenatal care utilisation among women in Tanzania: A decomposition analysis of population-based cross-sectional surveys	2021	Bintabara & Basinda	https://doi.org/10.1136/bmjopen-2020-040450		No			Yes	Maternal education	None/Primary/Secondary/Higher	Yes	Wealth quintile
Socio-demographic and economic inequalities in modern contraception in 11 low- And middle-income countries: An analysis of the PMA2020 surveys	2020	Blumenberg et al.	https://doi.org/10.1186/s12978-020-00931-w		No			Yes	Education	None/Primary/Secondary+	Yes	Wealth quintile

1. BIBLIOGRAPHIC SOURCE												
**Title**	**Year**	**First author**	**UHL**	**Measure**	**Religion**	**Name (acronym)**	**Measure**	**Education**	**Name (acronym)**	**Measure**	**Socioeconomic status (SES)**	**Name (acronym)**
A decomposition analysis of change in skilled birth attendants, 2003 to 2008, Ghana demographic and health surveys	2014	Bosomprah et al.	https://doi.org/10.1186/s12884-014-0415-x		No			Yes	Mother's education	None/Primary/Middle or Junior High School/Secondary+	Yes	Wealth quintile
Slow and Steady can Still Win the Race': Childhood Vaccination Experience of Migrant Ebira Women Within the Health System in Ekiti State, Nigeria	2021	Olakanmi-Falade & Awoleke	https://www.ojhas.org/issue79/2021-3-3.html		Yes	Religion	Christian/Muslim	Yes	Education level	No formal/Primary/Secondary/Tertiary	Yes	Income
Antiretroviral therapy in Walvis Bay, Namibia	2016	Callaghan	https://hdl.handle.net/1807/70825	Male/Female	No			No			No	

1. BIBLIOGRAPHIC SOURCE												
**Title**	**Year**	**First author**	**UHL**	**Measure**	**Religion**	**Name (acronym)**	**Measure**	**Education**	**Name (acronym)**	**Measure**	**Socioeconomic status (SES)**	**Name (acronym)**
Family planning, antenatal and delivery care: Cross-sectional survey evidence on levels of coverage and inequalities by public and private sector in 57 low- and middle-income countries	2016	Campbell et al.	https://doi.org/10.1111/tmi.12681		No			No			Yes	Wealth quintile

1. BIBLIOGRAPHIC SOURCE												
**Title**	**Year**	**First author**	**UHL**	**Measure**	**Religion**	**Name (acronym)**	**Measure**	**Education**	**Name (acronym)**	**Measure**	**Socioeconomic status (SES)**	**Name (acronym)**
Use of family planning and child health services in the private sector: An equity analysis of 12 DHS surveys	2018	Chakraborty & Sprockett	https://doi.org/10.1186/s12939-018-0763-7		No			No			Yes	Wealth quintile
Meeting demand for family planning within a generation: prospects and implications at country level	2015	Choi et al.	https://dx.doi.org/10.3402/gha.v8.29734		No			No			Yes	Wealth quintile
A Longitudinal Analysis of Mosquito Net Ownership and Use in an Indigenous Batwa Population after a Targeted Distribution	2016	Clark et al.	https://doi.org/10.1371/journal.pone.0154808		No			No			No	

1. BIBLIOGRAPHIC SOURCE												
Title	Year	First author	UHL	Measure	Religion	Name (acronym)	Measure	Education	Name (acronym)	Measure	Socioeconomic status (SES)	Name (acronym)
Malaria prevalence and long-lasting insecticidal net use in rural western Uganda: results of a cross-sectional survey conducted in an area of highly variable malaria transmission intensity	2021	Cote et al.	https://doi.org/10.1186/s12936-021-03835-7		No			No			No	
Women's Sexual Empowerment and Contraceptive Use in Ghana	2012	Crissman et al.	https://doi.org/10.1111/j.1728-4465.2012.00318.x		Yes	Religion	Christian/Muslim/Other	Yes	Education	None/Some	Yes	Wealth index
Socio-economic and demographic factors associated with reproductive and child health preventive care in Mozambique: a cross-sectional study	2020	Daca et al.	https://doi.org/10.1186/s12939-020-01303-3		No			Yes	Education	No education/Primary/Secondary	Yes	Wealth quintile

1. BIBLIOGRAPHIC SOURCE												
**Title**	**Year**	**First author**	**UHL**	**Measure**	**Religion**	**Name (acronym)**	**Measure**	**Education**	**Name (acronym)**	**Measure**	**Socioeconomic status (SES)**	**Name (acronym)**
Assessing the contextual effect of community in the utilization of postnatal care services in Ghana	2021	Dankwah et al.	https://doi.org/10.1186/s12913-020-06028-1		Yes	Religion	Traditional/Other, Muslim/Christian	Yes	Education	None/Primary/Secondary or higher	Yes	Wealth status
Is South Africa closing the health gaps between districts? Monitoring progress towards universal health service coverage with routine facility data	2021	Day et al.	https://doi.org/10.1186/s12913-021-06171-3		No			No			Yes	Socioeconomic quintile
Neglected tropical diseases as a 'litmus test' for universal health coverage? understanding who is left behind and why in mass drug administration: Lessons from four country contexts	2019	Dean et al.	https://doi.org/10.1371/journal.pntd.0007847	Male, Female	No			No			Yes	Poverty

1. BIBLIOGRAPHIC SOURCE												
**Title**	**Year**	**First author**	**UHL**	**Measure**	**Religion**	**Name (acronym)**	**Measure**	**Education**	**Name (acronym)**	**Measure**	**Socioeconomic status (SES)**	**Name (acronym)**
Preventive Health Service Coverage Among Infants and Children at Six Maternal-Child Health Clinics in Western Kenya: A Cross-Sectional Assessment	2022	Deathe et al.	https://doi.org/10.1007/s10995-021-03271-8	Male/Female	No			No			No	
Distance, difference in altitude and socioeconomic determinants of utilisation of maternal and child health services in Ethiopia: A geographic and multilevel modelling analysis	2021	Defar et al.	https://doi.org/10.1136/bmjopen-2020-042095	Male/Female	No			Yes	Educational level	No schooling/Schooling	Yes	Wealth tertiles
Geographic differences in maternal and child health care utilization in four Ethiopian regions; A cross-sectional study	2019	Defar et al.	https://doi.org/10.1186/s12939-019-1079-y	Proportion of men	No			Yes	Educational level of women	Mean educational level of women	Yes	Wealth

1. BIBLIOGRAPHIC SOURCE												
**Title**	**Year**	**First author**	**UHL**	**Measure**	**Religion**	**Name (acronym)**	**Measure**	**Education**	**Name (acronym)**	**Measure**	**Socioeconomic status (SES)**	**Name (acronym)**
Ownership and use of insecticide-treated nets in Oromia and Amhara Regional States of Ethiopia twoyears after a nationwide campaign	2011	Deressa et al.	https://doi.org/10.1111/j.1365-3156.2011.02875.x		No			No			No	
Effectiveness of post-campaign, door-to-door, hang-up, and communication interventions to increase long-lasting, insecticidal bed net utilization in Togo (2011-2012): A cluster randomized, control trial	2014	Desrochers et al.	https://doi.org/10.1186/1475-2875-13-260		No			No			Yes	Wealth quintile
Immediate postnatal care following childbirth in Ugandan health facilities: An analysis of Demographic and Health Surveys between 2001 and 2016	2021	Dey et al.	https://doi.org/10.1136/bmjgh-2020-004230		Yes	Religion	Christian/Other	Yes	Highest level of education	No education/Primary/Secondary/Higher	Yes	Wealth quintile

1. BIBLIOGRAPHIC SOURCE												
**Title**	**Year**	**First author**	**UHL**	**Measure**	**Religion**	**Name (acronym)**	**Measure**	**Education**	**Name (acronym)**	**Measure**	**Socioeconomic status (SES)**	**Name (acronym)**
Inequalities in non-communicable diseases and effective responses	2013	Di Cesare et al.	https://doi.org/10.1016/s0140-6736(12)61851-0	Male/Female	No			Yes	no formal schooling 22·3%; less than primary school 34·4%; primary school completed 27·7%; secondary school or higher [illegible]		No	
Insecticide-treated nets ownership and utilization among under-five children following the 2010 mass distribution in Burkina Faso	2014	Diabate et al.	https://dx.doi.org/10.1186/1475-2875-13-353		No			No			Yes	Household socioeconomic characteristics

1. BIBLIOGRAPHIC SOURCE												
**Title**	**Year**	**First author**	**UHL**	**Measure**	**Religion**	**Name (acronym)**	**Measure**	**Education**	**Name (acronym)**	**Measure**	**Socioeconomic status (SES)**	**Name (acronym)**
Impact of mining projects on water and sanitation infrastructures and associated child health outcomes: a multi-country analysis of Demographic and Health Surveys (DHS) in sub-Saharan Africa	2021	Dietler et al.	https://doi.org/10.1186/s12992-021-00723-2		No			No			Yes	Wealth quintile

1. BIBLIOGRAPHIC SOURCE												
**Title**	**Year**	**First author**	**UHL**	**Measure**	**Religion**	**Name (acronym)**	**Measure**	**Education**	**Name (acronym)**	**Measure**	**Socioeconomic status (SES)**	**Name (acronym)**
Predictors of insecticidal net use among internally displaced persons aged 6-59 months in Abuja, Nigeria	2018	Ejembi et al.	http://dx.doi.org/10.11604/pamj.2018.29.136.13322	Male/Female	Yes	Religion	Christian/Muslim	Yes	Education of mother	Formal/Non-formal	No	
Changes in Inequality in Use of Maternal Health Care Services: Evidence from Skilled Birth Attendance in Mauritania for the Period 2007-2015	2022	Taleb El Hassen et al.	https://doi.org/10.3390/ijerph19063566		No			Yes	Maternal level of education	No education, primary, secondary, higher	Yes	Wealth quintile

1. BIBLIOGRAPHIC SOURCE												
**Title**	**Year**	**First author**	**UHL**	**Measure**	**Religion**	**Name (acronym)**	**Measure**	**Education**	**Name (acronym)**	**Measure**	**Socioeconomic status (SES)**	**Name (acronym)**
Trend in the use of modern contraception in sub-Saharan Africa: Does women's education matter?	2014	Emina et al.	http://dx.doi.org/10.1016/j.contraception.2014.02.001		No			Yes	Female education	non-educated, primary education and secondary education or higher.	No	

1. BIBLIOGRAPHIC SOURCE												
**Title**	**Year**	**First author**	**UHL**	**Measure**	**Religion**	**Name (acronym)**	**Measure**	**Education**	**Name (acronym)**	**Measure**	**Socioeconomic status (SES)**	**Name (acronym)**
Utilization, Predictors and Gaps in the Continuum of Care for Maternal and Newborn Health in Ghana	2021	Enos et al.	https://dx.doi.org/10.21106/ijma.425		No			Yes	Education	No education/ Primary or Junior High Schooll/Secondary or higher	Yes	Income
ART use and associated factors among HIV positive caregivers of orphans and vulnerable children in Tanzania	2020	Exavery et al.	https://doi.org/10.1186/s12889-020-09361-6	Male/Female	No			Yes	Education	Never been to school/Primary/Secondary or higher	Yes	Wealth quintile
Trends and causes of socioeconomic inequalities in maternal healthcare in Ghana, 2003-2014	2019	Fenny et al.	https://doi.org/10.1108/ijse-03-2018-0148		Yes	Religion	Christian/Moslem/No or other	Yes	Schooling	Years of schooling	Yes	Wealth quintile

1. BIBLIOGRAPHIC SOURCE												
**Title**	**Year**	**First author**	**UHL**	**Measure**	**Religion**	**Name (acronym)**	**Measure**	**Education**	**Name (acronym)**	**Measure**	**Socioeconomic status (SES)**	**Name (acronym)**
Determination of the predictive factors of long-lasting insecticide-treated net ownership and utilisation in the Bamenda Health District of Cameroon	2017	Fokam et al.	https://doi.org/10.1186/s12889-017-4155-5		No			Yes	Education level of household head	No formal education/ Primary level/Secondary level/Tertiary level	No	
The free caesareans policy in low-income settings: An interrupted time series analysis in Mali (2003-2012)	2014	Fournier et al.	https://doi.org/10.1371/journal.pone.0105130		No			No			No	

1. BIBLIOGRAPHIC SOURCE												
**Title**	**Year**	**First author**	**UHL**	**Measure**	**Religion**	**Name (acronym)**	**Measure**	**Education**	**Name (acronym)**	**Measure**	**Socioeconomic status (SES)**	**Name (acronym)**
DO BETTER INSTITUTIONS BROADEN ACCESS TO SANITATION IN SUB-SAHARA AFRICA?	2021	Francois et al.	https://doi.org/10.1111/coep.12512		No			No			No	

1. BIBLIOGRAPHIC SOURCE												
**Title**	**Year**	**First author**	**UHL**	**Measure**	**Religion**	**Name (acronym)**	**Measure**	**Education**	**Name (acronym)**	**Measure**	**Socioeconomic status (SES)**	**Name (acronym)**
Ethnic disparities in utilisation of maternal health care services in Ghana: evidence from the 2007 Ghana Maternal Health Survey	2016	Ganle	https://doi.org/10.1080/13557858.2015.1015499		No			No			No	
Risky sexual behaviour and contraceptive use in contexts of displacement: Insights from a cross-sectional survey of female adolescent refugees in Ghana	2019	Ganle et al.	https://doi.org/10.1186/s12939-019-1031-1		No			No			No	
Understanding how distance to facility and quality of care affect maternal health service utilization in Kenya and Haiti: A comparative geographic information system study	2019	Gao & Kelley	https://doi.org/10.4081/gh.2019.690		No			Yes	Education level	No education/ primary education/ secondary education, or higher education	No	

1. BIBLIOGRAPHIC SOURCE												
**Title**	**Year**	**First author**	**UHL**	**Measure**	**Religion**	**Name (acronym)**	**Measure**	**Education**	**Name (acronym)**	**Measure**	**Socioeconomic status (SES)**	**Name (acronym)**
Gender differences in the use of insecticide-treated nets after a universal free distribution campaign in Kano State, Nigeria: Post-campaign survey results	2013	Garley et al.	https://doi.org/10.1186/1475-2875-12-119		No			No			Yes	Wealth quintile
Demand satisfied by modern contraceptive among married women of reproductive age in Kenya	2021	Gichangi et al.	https://doi.org/10.1371/journal.pone.0248393		No			Yes	Level of education	None/primary/ secondary or higher	Yes	Wealth quintile
LLIN Evaluation in Uganda Project (LLINEUP): Factors associated with ownership and use of long-lasting insecticidal nets in Uganda: A cross-sectional survey of 48 districts ISRCTN17516395 ISRCTN	2018	Gonahasa et al.	https://doi.org/10.1186/s12936-018-2571-3	Male/Female	No			No			Yes	Wealth terciles

1. BIBLIOGRAPHIC SOURCE												
**Title**	**Year**	**First author**	**UHL**	**Measure**	**Religion**	**Name (acronym)**	**Measure**	**Education**	**Name (acronym)**	**Measure**	**Socioeconomic status (SES)**	**Name (acronym)**
ART adherence and viral suppression are high among most non-pregnant individuals with early-stage, asymptomatic HIV infection: an observational study from Uganda and South Africa	2019	Haberer et al.	https://doi.org/10.1002/jia2.25232	Male/Female	No			Yes	Education	None or primary/Secondary	Yes	Regular income
Low immunization coverage in Wonago district, southern Ethiopia: A community-based cross-sectional study	2019	Hailu et al.	https://doi.org/10.1371/journal.pone.0220144		No			Yes	Education	no formal education/ primary/secondary and college or university)	No	

1. BIBLIOGRAPHIC SOURCE												
**Title**	**Year**	**First author**	**UHL**	**Measure**	**Religion**	**Name (acronym)**	**Measure**	**Education**	**Name (acronym)**	**Measure**	**Socioeconomic status (SES)**	**Name (acronym)**
Towards universal health coverage: The role of within-country wealth-related inequality in 28 countries in sub-Saharan Africa	2011	Hosseinpoor et al.	https://doi.org/10.2471/BLT.11.087536		No			No			Yes	Wealth quintile

1. BIBLIOGRAPHIC SOURCE												
**Title**	**Year**	**First author**	**UHL**	**Measure**	**Religion**	**Name (acronym)**	**Measure**	**Education**	**Name (acronym)**	**Measure**	**Socioeconomic status (SES)**	**Name (acronym)**
Patterns and trends of contraceptive use among sexually active adolescents in Burkina Faso, Ethiopia, and Nigeria: evidence from cross-sectional studies	2015	Hounton et al.	https://doi.org/10.3402/gha.v8.29737		No			Yes	Education	None/Primary/Secondary or higher	Yes	Wealth quintile
Towards universal health coverage for reproductive health services in Ethiopia: two policy recommendations	2015	Onarheim et al.	https://doi.org/10.1186/s12939-015-0218-3	Male, Female	Yes	Religious view	Islam, Orthodox Christianity, Protestant Christianity, Other religions	Yes	Education	Education, No education	Yes	Wealth

1. BIBLIOGRAPHIC SOURCE												
**Title**	**Year**	**First author**	**UHL**	**Measure**	**Religion**	**Name (acronym)**	**Measure**	**Education**	**Name (acronym)**	**Measure**	**Socioeconomic status (SES)**	**Name (acronym)**
Modern Contraception: Uptake and Correlates among Women of Reproductive Age-Group in a Rural Community of Osun State, Nigeria	2020	Idowu et al.	https://doi.org/10.4314/ejhs.v30i4.8		No			Yes	levels of education	Educated/ Uneducated	Yes	Monthly income
Determinants of geographical inequalities for DTP3 vaccine coverage in sub-Saharan Africa	2020	Ikilezi et al.	https://doi.org/10.1016/j.vaccine.2020.03.005		No			Yes	Maternal education	Unclear	No	
Influence of women's decision-making autonomy on antenatal care utilisation and institutional delivery services in Nigeria: evidence from the Nigeria Demographic and Health Survey 2018	2022	Imo	https://doi.org/10.1186/s12884-022-04478-5		No			Yes	Education	No eduation/Primary/Secondary or higher	Yes	Wealth quintile

1. BIBLIOGRAPHIC SOURCE												
**Title**	**Year**	**First author**	**UHL**	**Measure**	**Religion**	**Name (acronym)**	**Measure**	**Education**	**Name (acronym)**	**Measure**	**Socioeconomic status (SES)**	**Name (acronym)**
Individual and community-level determinants of cervical cancer screening in Zimbabwe: a multi-level analyses of a nationwide survey	2022	Isabirye et al.	https://doi.org/10.1186/s12905-022-01881-0		Yes	Religion	Christians/Non-christians	Yes	Education	<=Primary/Secondary/Postsecondary	Yes	Wealth quintile
Two decades of maternity care fee exemption policies in Ghana: have they benefited the poor?	2016	Johnson et al.	https://doi.org/10.1093/heapol/czv017		Yes	Religion	Christian/Moslem/Other	Yes	Mother's and Partner's education	No formal education/Primary/Secondary or higher	Yes	Wealth quintile

1. BIBLIOGRAPHIC SOURCE												
**Title**	**Year**	**First author**	**UHL**	**Measure**	**Religion**	**Name (acronym)**	**Measure**	**Education**	**Name (acronym)**	**Measure**	**Socioeconomic status (SES)**	**Name (acronym)**
Socioeconomic inequalities in access to skilled birth attendance among urban and rural women in low-income and middle-income countries	2018	Joseph et al.	https://doi.org/10.1136/bmjgh-2018-000898		No			No			Yes	Wealth quintile

1. BIBLIOGRAPHIC SOURCE												
**Title**	**Year**	**First author**	**UHL**	**Measure**	**Religion**	**Name (acronym)**	**Measure**	**Education**	**Name (acronym)**	**Measure**	**Socioeconomic status (SES)**	**Name (acronym)**
Investigating the disparities in cervical cancer screening among Namibian women	2015	Kangmennaang et al.	https://doi.org/10.1016/j.ygyno.2015.05.036		Yes	Religion	Catholic/Protestant/Elcin/Others	Yes	Education	No education/Primary/Secondary/Tertiary or higher	Yes	Wealth
Contraceptive use and needs among adolescent women aged 15-19: Regional and global estimates and projections from 1990 to 2030 from a Bayesian hierarchical modelling study	2021	Kantorová et al.	https://doi.org/10.1371/journal.pone.0247479		No			No			No	
Changes in equity of maternal, newborn, and child health care practices in 115 districts of rural Ethiopia: Implications for the health extension program	2015	Karim et al.	https://doi.org/10.1186/s12884-015-0668-z		No			Yes	Educational level	None, primary, higher	Yes	Wealth tertiles

1. BIBLIOGRAPHIC SOURCE												
**Title**	**Year**	**First author**	**UHL**	**Measure**	**Religion**	**Name (acronym)**	**Measure**	**Education**	**Name (acronym)**	**Measure**	**Socioeconomic status (SES)**	**Name (acronym)**
Contraceptive dynamics during COVID-19 in sub-Saharan Africa: Longitudinal evidence from Burkina Faso and Kenya	2021	Karp et al.	https://doi.org/10.1136/bmjsrh-2020-200944		No			Yes	Educational level	Lower, higher	Yes	Wealth tertiles
Long-lasting insecticidal net source, ownership and use in the context of universal coverage: A household survey in eastern Rwanda	2015	Kateera et al.	https://doi.org/10.1186/s12936-015-0915-9	Male, female	No			Yes	Highest educational level attained by heald of household	None, Primary school, Post primary/vocational, Secondary or higher	Yes	Household socio-economic status (SES) score
A quasi-experimental evaluation of an interpersonal communication intervention to increase insecticide-treated net use among children in Zambia	2012	Keating et al.	https://doi.org/10.1186/1475-2875-11-313	Male, female	No			Yes	Mother's education	None – Primary 6, Primary 7 - Higher	Yes	Household wealth

1. BIBLIOGRAPHIC SOURCE												
**Title**	**Year**	**First author**	**UHL**	**Measure**	**Religion**	**Name (acronym)**	**Measure**	**Education**	**Name (acronym)**	**Measure**	**Socioeconomic status (SES)**	**Name (acronym)**
Assessment of Inequalities in Coverage of Essential Reproductive, Maternal, Newborn, Child, and Adolescent Health Interventions in Kenya	2018	Keats et al.	https://doi.org/10.1001/jamanetworkopen.2018.5152		No			No			Yes	wealth quintiles
Charting health system reconstruction in post-war Liberia: a comparison of rural vs. remote healthcare utilization	2016	Kentoffioet al.	https://doi.org/10.1186/s12913-016-1709-7		No			No			No	

1. BIBLIOGRAPHIC SOURCE											
**Title**	**Year**	**First author**	**UHL**	**Measure**	**Religion**	**Name (acronym)**	**Measure**	**Education**	**Name (acronym)**	**Measure**	**Socioeconomic status (SES)**	**Name (acronym)**
A Transparent Universal Health Coverage Index with Decomposition by Socioeconomic Groups: Application in Asian and African Settings	2019	Khan et al.	https://doi.org/10.1007/s40258-019-00464-9		No			No			Yes	Socioeconomic quintiles
Effect of user preferences on ITN use: a review of literature and data	2017	Koenker & Yukich	https://doi.org/10.1186/s12936-017-1879-8	Yes, No	No			No			Yes	Wealth quintiles

1. BIBLIOGRAPHIC SOURCE												
**Title**	**Year**	**First author**	**UHL**	**Measure**	**Religion**	**Name (acronym)**	**Measure**	**Education**	**Name (acronym)**	**Measure**	**Socioeconomic status (SES)**	**Name (acronym)**
Insecticide-treated net use before and after mass distribution in a fishing community along Lake Victoria, Kenya: successes and unavoidable pitfalls	2014	Larson et al.	http://www.malariajournal.com/content/13/1/466	Male, Female	No			Yes	1) Husband Education; 2) Wife Education	1-2) Never, Primary, Secondary, College	Yes	Wealth Quintiles
Lifetime Prevalence of Cervical Cancer Screening in 55 Low- and Middle-Income Countries	2020	Lemp et al.	https://doi.org/10.1001/jama.2020.16244		No			Yes	Education	Secondary education/ primary school	Yes	Wealth

1. BIBLIOGRAPHIC SOURCE												
**Title**	**Year**	**First author**	**UHL**	**Measure**	**Religion**	**Name (acronym)**	**Measure**	**Education**	**Name (acronym)**	**Measure**	**Socioeconomic status (SES)**	**Name (acronym)**
Financial accessibility and user fee reforms for maternal healthcare in five sub-Saharan countries: a quasi-experimental analysis	2016	Leone et al.	https://dx.doi.org/10.1136/bmjopen-2015-009692		No			Yes	level of education	No education, Primary, Secondary, Higher	Yes	Wealth
Antiretroviral treatment coverage in a rural district in Tanzania--a modeling study using empirical data	2015	Levira et al.	https://doi.org/10.1186/s12889-015-1460-8	Male/Female	No			No			No	
The extent of universal health coverage for maternal health services in eastern uganda: A cross sectional study	2021	Lindberg et al.	https://dx.doi.org/10.1007/s10995-021-03357-3		No			Yes	Level of education	None, primary, secondary, vocational/diploma, higher	Yes	Socio-economic status

1. BIBLIOGRAPHIC SOURCE												
**Title**	**Year**	**First author**	**UHL**	**Measure**	**Religion**	**Name (acronym)**	**Measure**	**Education**	**Name (acronym)**	**Measure**	**Socioeconomic status (SES)**	**Name (acronym)**
Freely distributed bed-net use among Chano Mille residents, south Ethiopia: A longitudinal study	2013	Loha et al.	https://doi.org/10.1186/1475-2875-12-23	Male/Female	No			No			Yes	Wealth index
Determinants of hanging and use of ITNs in the context of near universal coverage in Zambia	2012	Macintyre et al.	https://doi.org/10.1093/heapol/czr042	Infant, Female	No			Yes	Mother's education	No education, Some primary, Primary plus	Yes	Household wealth

1. BIBLIOGRAPHIC SOURCE												
**Title**	**Year**	**First author**	**UHL**	**Measure**	**Religion**	**Name (acronym)**	**Measure**	**Education**	**Name (acronym)**	**Measure**	**Socioeconomic status (SES)**	**Name (acronym)**
Determinants and Consequences of Failure of Linkage to Antiretroviral Therapy at Primary Care Level in Blantyre, Malawi: A Prospective Cohort Study	2012	MacPherson et al.	https://doi.org/10.1371/journal.pone.0044794	Female non-pregnant, Male, Female pregnant	No			Yes	Literacy	Able to read a newspaper, Illiterate	Yes	Wealth quartile
Disability and sexual and reproductive health service utilisation in Uganda: an intersectional analysis of demographic and health surveys between 2006 and 2016	2022	Mac-Seing et al.	https://doi.org/10.1186/s12889-022-12708-w	Men, Women	Yes	Religion	Anglican, Catholic, Muslim, Seven Day Adventist / Pentecostal / Born Again / Evangelical, Other	Yes	Highest education level attained	No education, Primary, Secondary, Higher	Yes	Wealth index

1. BIBLIOGRAPHIC SOURCE												
Title	Year	First author	UHL	Measure	Religion	Name (acronym)	Measure	Education	Name (acronym)	Measure	Socioeconomic status (SES)	Name (acronym)
Insight into Nigeria's progress towards the universal coverage of reproductive, maternal, newborn and child health services: a secondary data analysis	2022	Mafiana et al.	http://dx.doi.org/10.1136/bmjopen-2022-061595		No			Yes	Maternal education	No education, Primary school, Secondary school or above	Yes	Socioeconomic status (wealth quintile)
Implementation of Urban Health Equity Assessment and Response Tool: a Case of Matsapha, Swaziland	2018	Makadzange et al.	https://doi.org/10.1007/s11524-018-0241-y		No			No			No	
Effects of door-to-door hang-up visits on the use of long-lasting insecticide-treated mosquito nets in the democratic republic of the congo: A cluster randomized controlled trial	2021	Mankadi and Jin	https://doi.org/10.3390/ijerph18179048	Male, Female	No			No			No	

1. BIBLIOGRAPHIC SOURCE												
**Title**	**Year**	**First author**	**UHL**	**Measure**	**Religion**	**Name (acronym)**	**Measure**	**Education**	**Name (acronym)**	**Measure**	**Socioeconomic status (SES)**	**Name (acronym)**
Effect of bed net colour and shape preferences on bed net usage: a secondary data analysis of the 2017 Malawi Malaria Indicator Survey	2020	Mategula et al.	https://doi.org/10.1186/s12936-020-03499-9	Male, Female	No			No			Yes	Wealth index
HIV care coverage among HIV-positive adolescent girls and young women in South Africa: Results from the HERStory Study	2021	Mathews et al.	https://doi.org/10.7196/SAMJ.2021.v111i5.15351		No			Yes	Currently in school	Yes, No	Yes	Socioeconomic status
Facilitators and barriers to retention in care under universal antiretroviral therapy (Option B+) for the Prevention of Mother to Child Transmission of HIV (PMTCT): A narrative review	2021	Mbeya Munkhondya et al.	https://doi.org/10.1016/j.ijans.2021.100372		No			No			No	

1. BIBLIOGRAPHIC SOURCE												
**Title**	**Year**	**First author**	**UHL**	**Measure**	**Religion**	**Name (acronym)**	**Measure**	**Education**	**Name (acronym)**	**Measure**	**Socioeconomic status (SES)**	**Name (acronym)**
The consequences of declining population access to insecticide-treated nets (ITNs) on net use patterns and physical degradation of nets after 22 months of ownership	2021	Mboma et al.	https://doi.org/10.1186/s12936-021-03686-2		No			No			No	
Mosquito net coverage in years between mass distributions: a case study of Tanzania, 2013	2018	Mboma et al.	https://doi.org/10.1186/s12936-018-2247-z		No			No			Yes	Socio-economic quintile
Factors associated with contraceptive use in Tigray, North Ethiopia	2017	Medhanyie et al.	https://doi.org/10.1186/s12978-017-0281-x		No			Yes	1) Women education; 2) Husband education	1-2) No formal education, Primary education, Secondary education, More than secondary	No	

1. BIBLIOGRAPHIC SOURCE												
**Title**	**Year**	**First author**	**UHL**	**Measure**	**Religion**	**Name (acronym)**	**Measure**	**Education**	**Name (acronym)**	**Measure**	**Socioeconomic status (SES)**	**Name (acronym)**
Adolescent sexual and reproductive health in sub-Saharan Africa: who is left behind?	2020	Melesse et al.	https://doi.org/10.1136/bmjgh-2019-002231	Girls, Boys, Women, Men	No			Yes	Education level	Primary or lower, Secondary or higher	Yes	Wealth tertile
Sub-national levels and trends in contraceptive prevalence, unmet need, and demand for family planning in Nigeria with survey uncertainty	2019	Mercer et al.	https://doi.org/10.1186/s12889-019-8043-z		No			No			No	

1. BIBLIOGRAPHIC SOURCE												
**Title**	**Year**	**First author**	**UHL**	**Measure**	**Religion**	**Name (acronym)**	**Measure**	**Education**	**Name (acronym)**	**Measure**	**Socioeconomic status (SES)**	**Name (acronym)**
Exploring inequities in skilled care at birth among migrant population in a metropolitan city Addis Ababa, Ethiopia; A qualitative study	2014	Mirkuzie	http://www.equityhealthj.com/content/13/1/110		No			Yes	Education	No education, some education	No	
Factors associated with the use of mosquito bed nets: results from two cross-sectional household surveys in Zambezia Province, Mozambique	2016	Moon et al.	https://doi.org/10.1186/s12936-016-1250-5		No			Yes	Education category	0-5, 6-10, >10	Yes	Household income
Sociocultural and Institutional Constraints to Family Planning Uptake Among Migrant Female Head Porters in Madina, a Suburb of Accra, Ghana	2021	Munemo et al.	https://doi.org/10.1177/088610992095441 9		No			No			No	

1. BIBLIOGRAPHIC SOURCE												
**Title**	**Year**	**First author**	**UHL**	**Measure**	**Religion**	**Name (acronym)**	**Measure**	**Education**	**Name (acronym)**	**Measure**	**Socioeconomic status (SES)**	**Name (acronym)**
Reframing non-communicable diseases and injuries for equity in the era of universal health coverage: Findings and recommendations from the Kenya NCDI poverty commission	2021	Mwangi et al.	https://doi.org/10.5334/aogh.3085		No			No			Yes	Wealth quintile

1. BIBLIOGRAPHIC SOURCE												
**Title**	**Year**	**First author**	**UHL**	**Measure**	**Religion**	**Name (acronym)**	**Measure**	**Education**	**Name (acronym)**	**Measure**	**Socioeconomic status (SES)**	**Name (acronym)**
Inequities and their determinants in coverage of maternal health services in Burkina Faso	2018	Mwase et al.	https://doi.org/10.1186/s12939-018-0770-8		Yes	Religion	Christian, Moslem, Other	Yes	Literacy	illiterate, iLterate	Yes	Household Wealth Quintiles

1. BIBLIOGRAPHIC SOURCE												
**Title**	**Year**	**First author**	**UHL**	**Measure**	**Religion**	**Name (acronym)**	**Measure**	**Education**	**Name (acronym)**	**Measure**	**Socioeconomic status (SES)**	**Name (acronym)**
A Cross-Sectional Study on Hypertension Medication Adherence in a High-Burden Region in Namibia: Exploring Hypertension Interventions and Validation of the Namibia Hill-Bone Compliance Scale	2022	Nakwafila et al.	https://doi.org/10.3390/ijerph19074416	Male, Female	No			Yes	Education	None, Primary, Secondary, Tertiary	Yes	Income
Assessing Adherence to Antihypertensive Therapy in Primary Health Care in Namibia: Findings and Implications	2017	Nashilongo et al.	https://doi.org/10.1007/s10557-017-6756-8	Male, Female	No			Yes	1) Education level; 2) Secondary education level	1) At least primary, No education; 2) Yes, No	No	

1. BIBLIOGRAPHIC SOURCE												
**Title**	**Year**	**First author**	**UHL**	**Measure**	**Religion**	**Name (acronym)**	**Measure**	**Education**	**Name (acronym)**	**Measure**	**Socioeconomic status (SES)**	**Name (acronym)**
Incomplete vaccination and associated factors among children aged 12–23 months in South Africa: an analysis of the South African demographic and health survey 2016	2021	Ndwandwe et al.	https://doi.org/10.1080/21645515.2020.1791509	Male, Female	No			Yes	Maternal education	Less than secondary, Secondary/ higher	Yes	Wealth index
Determining the effective coverage of maternal and child health services in Kenya, using demographic and health survey data sets: tracking progress towards universal health coverage	2017	Nguhiu et al.	https://doi.org/10.1111/tmi.12841		No			No			Yes	Wealth index

1. BIBLIOGRAPHIC SOURCE												
**Title**	**Year**	**First author**	**UHL**	**Measure**	**Religion**	**Name (acronym)**	**Measure**	**Education**	**Name (acronym)**	**Measure**	**Socioeconomic status (SES)**	**Name (acronym)**
Coverage and usage of insecticide treated nets (ITNs) within households: associated factors and effect on the prevalance of malaria parasitemia in the Mount Cameroon area	2019	Njumkeng et al.	https://doi.org/10.1186/s12889-019-7555-x	Male, Female	No			Yes	Level of education	At least Higher, Secondary, At most Primary	No	
Socioeconomic inequalities in maternal health care utilization in Ghana	2019	Novignon et al.	https://doi.org/10.1186/s12939-019-1043-x		No			Yes	Education	No education, Primary, Secondary, Higher	Yes	Wealth status

1. BIBLIOGRAPHIC SOURCE												
**Title**	**Year**	**First author**	**UHL**	**Measure**	**Religion**	**Name (acronym)**	**Measure**	**Education**	**Name (acronym)**	**Measure**	**Socioeconomic status (SES)**	**Name (acronym)**
Why rural women do not use primary health centres for pregnancy care: Evidence from a qualitative study in Nigeria	2019	Ntoimo et al.	https://doi.org/10.1186/s12884-019-2433-1		No			No			No	
Long-lasting insecticidal net (LLIN) ownership, use and cost of implementation after a mass distribution campaign in Kasaï Occidental Province, Democratic Republic of Congo	2017	Ntuku et al.	https://doi.org/10.1186/s12936-016-1671-1	Male, Female	No			Yes	Education of the head of the household	No education, Primary, Secondary, Superior/Above	Yes	Wealth quintile
Explaining socioeconomic disparities and gaps in the use of antenatal care services in 36 countries in sub-Saharan Africa	2021	Obse & Ataguba	https://doi.org/10.1093/heapol/czab036	Percentage	No			Yes	Education attainment levels		Yes	Wealth index

1. BIBLIOGRAPHIC SOURCE												
Title	Year	First author	UHL	Measure	Religion	Name (acronym)	Measure	Education	Name (acronym)	Measure	Socioeconomic status (SES)	Name (acronym)
Sociodemographic factors associated with the use of insecticide treated nets among under-fives in Nigeria: Evidence from a national survey	2022	Ojo et al.	https://doi.org/10.1177/0049475522 1110374	1-2) Male, Female	No			No			Yes	Wealth Index
Towards making efficient use of household resources for appropriate prevention of malaria: investigating households' ownership, use and expenditures on ITNs and other preventive tools in Southeast Nigeria	2014	Onwujekwe et al.	http://www.biomedcentral.com/1471-2458/14/315		No			No			Yes	Socioeconomic status (SES) quintiles

1. BIBLIOGRAPHIC SOURCE												
**Title**	**Year**	**First author**	**UHL**	**Measure**	**Religion**	**Name (acronym)**	**Measure**	**Education**	**Name (acronym)**	**Measure**	**Socioeconomic status (SES)**	**Name (acronym)**
Demographic disparities in unimproved drinking water and sanitation in Ghana: A nationally representative cross-sectional study	2022	Oppong et al.	http://dx.doi.org/10.1136/bmjopen-2021-060595	Male, Female	No			Yes	Level of education	no education, primary education, middle/junior or secondary school (JSS)/junior high school (JHS), secondary/senior secondary school (SSS)/senior high school ((SHS), more than secondary	No	
Inequities in Access to Maternal Health Care in Enugu State: Implications for Universal Health Coverage to Meet Vision 2030 in Nigeria	2019	Ozumba et al.	https://doi.org/10.1177/0272684X188199777		No			Yes	1) Level of Education; 2) Ever went to school	1) Low, High; 2) No, Yes	Yes	Wealth Index

1. BIBLIOGRAPHIC SOURCE												
**Title**	**Year**	**First author**	**UHL**	**Measure**	**Religion**	**Name (acronym)**	**Measure**	**Education**	**Name (acronym)**	**Measure**	**Socioeconomic status (SES)**	**Name (acronym)**
How do supply- and demand-side interventions influence equity in healthcare utilisation? Evidence from maternal healthcare in Senegal	2019	Parmar & Banerjee	https://doi.org/10.1016/j.socscimed.2019.112582		No			Yes	Mothers' education	No education, Some education	Yes	Household wealth index
Temporal and regional variations in use, equity and quality of antenatal care in Egypt: A repeat cross-sectional analysis using Demographic and Health Surveys	2019	Pugliese-Garcia et al.	https://doi.org/10.1186/s12884-019-2409-1		No			No			Yes	Wealth quintile

1. BIBLIOGRAPHIC SOURCE												
**Title**	**Year**	**First author**	**UHL**	**Measure**	**Religion**	**Name (acronym)**	**Measure**	**Education**	**Name (acronym)**	**Measure**	**Socioeconomic status (SES)**	**Name (acronym)**
Geographical Inequalities in Use of Improved Drinking Water Supply and Sanitation across Sub-Saharan Africa: Mapping and Spatial Analysis of Cross-sectional Survey Data	2014	Pullan et al.	https://doi.org/10.1371/journal.pmed.1001626		No			No			No	
Individual and Network Factors Associated With HIV Care Continuum Outcomes Among Nigerian MSM Accessing Health Care Services	2018	Ramadhani et al.	https://dx.doi.org/10.1097/QAI.0000000000001756		Yes	Religion	Christian, Moslem	Yes	Education	< Senior secondary school, Senior secondary school, > Senior secondary school	No	
Non-adherence to long-lasting insecticide treated bednet use following successful malaria control in Tororo, Uganda	2020	Rek et al.	https://doi.org/10.1371/journal.pone.0243303	Male, Female	No			No			Yes	Household wealth index (in tertiles)

1. BIBLIOGRAPHIC SOURCE												
**Title**	**Year**	**First author**	**UHL**	**Measure**	**Religion**	**Name (acronym)**	**Measure**	**Education**	**Name (acronym)**	**Measure**	**Socioeconomic status (SES)**	**Name (acronym)**
Design, implementation and evaluation of a national campaign to deliver 18 million free long-lasting insecticidal nets to uncovered sleeping spaces in Tanzania	2013	Renggli et al.	https://doi.org/10.1186/1475-2875-12-85		No			No			Yes	Wealth quintiles
A long way to go - Estimates of combined water, sanitation and hygiene coverage for 25 sub-Saharan African countries	2017	Roche et al.	https://doi.org/10.1371/journal.pone.0171783		No			No			Yes	Wealth inequalities

1. BIBLIOGRAPHIC SOURCE											
**Title**	**Year**	**First author**	**UHL**	**Measure**	**Religion**	**Name (acronym)**	**Measure**	**Education**	**Name (acronym)**	**Measure**	**Socioeconomic status (SES)**	**Name (acronym)**
Determinants of bed net use in southeast Nigeria following mass distribution of LLINs: Implications for social behavior change interventions	2015	Russell et al.	https://doi.org/10.1371/journal.pone.0139447	Male, Female	No			Yes	Education level	None, Primary, >=Secondary	Yes	Wealth index
Factors associated with the non-use of insecticide-treated nets in Rwandan children	2016	Ruyange et al.	https://doi.org/10.1186/s12936-016-1403-6	Male, Female	No			Yes	1) Mother education; 2) Community education level;	1-2) No education, Any education	Yes	1) Household wealth index; 2) Community wealth index

1. BIBLIOGRAPHIC SOURCE												
**Title**	**Year**	**First author**	**UHL**	**Measure**	**Religion**	**Name (acronym)**	**Measure**	**Education**	**Name (acronym)**	**Measure**	**Socioeconomic status (SES)**	**Name (acronym)**
Wealth Status, Health Insurance, and Maternal Health Care Utilization in Africa: Evidence from Gabon	2020	Sanogo & Yaya	https://dx.doi.org/10.1155/2020/4036830		Yes	Religion	Christianity, Islam, Other religions, No religion	Yes	Educational status	No education, primary, secondary, higher	Yes	Wealth status

1. BIBLIOGRAPHIC SOURCE												
**Title**	**Year**	**First author**	**UHL**	**Measure**	**Religion**	**Name (acronym)**	**Measure**	**Education**	**Name (acronym)**	**Measure**	**Socioeconomic status (SES)**	**Name (acronym)**
Factors associated with use of insecticide-treated net for malaria prevention in Manica District, Mozambique: a community-based cross-sectional survey	2021	Scott et al.	https://doi.org/10.1186/s12936-021-03738-7	Male, Female	No			Yes	Highest level of education completed by the head of the household	Never attended, primary school, secondary school or higher	Yes	Household wealth index
Healthcare utilisation, cancer screening and potential barriers to accessing cancer care in rural South West Nigeria: a cross-sectional study	2021	Sharma et al.	https://doi.org/10.1136/bmjopen-2020-040352	Man, Woman	Yes	Religion	Christian, Muslim, Others	Yes	Education	No formal education, Primary, Secondary, Higher	Yes	1) Personal income; 2) Family income

1. BIBLIOGRAPHIC SOURCE												
**Title**	**Year**	**First author**	**UHL**	**Measure**	**Religion**	**Name (acronym)**	**Measure**	**Education**	**Name (acronym)**	**Measure**	**Socioeconomic status (SES)**	**Name (acronym)**
Socio-economic inequalities in ANC attendance among mothers who gave birth in the past 12 months in Debre Brehan town and surrounding rural areas, North East Ethiopia: A community-based survey	2019	Shibre & Mekonnen	https://doi.org/10.1186/s12978-019-0768-8		No			Yes	1) Maternal education; 2) Husband's education	1-2) Primary, secondary, vocational, higher	Yes	Wealth index (household level SES)

1. BIBLIOGRAPHIC SOURCE												
**Title**	**Year**	**First author**	**UHL**	**Measure**	**Religion**	**Name (acronym)**	**Measure**	**Education**	**Name (acronym)**	**Measure**	**Socioeconomic status (SES)**	**Name (acronym)**
Individual and environmental characteristics associated with immunization of children in rural areas of Burkina Faso: A multi-level analysis	2007	Sia et al.	https://pubmed.ncbi.nlm.nih.gov/18299262/		Yes	Religion of the mother		Yes	Proportion de femmes éduquées [Proportion of educated women]		Yes	Niveau de vie [Level of household wealth]
Inequalities in access and utilization of maternal, newborn and child health services in sub-saharan africa: A special focus on urban settings	2021	Sidze et al.	https://dx.doi.org/10.1007/s10995-021-03250-z		Yes	Religion		Yes	Education		Yes	Wealth status

1. BIBLIOGRAPHIC SOURCE												
**Title**	**Year**	**First author**	**UHL**	**Measure**	**Religion**	**Name (acronym)**	**Measure**	**Education**	**Name (acronym)**	**Measure**	**Socioeconomic status (SES)**	**Name (acronym)**
Insecticide-treated bed net access and use among preschool children in Nouna District, Burkina Faso	2020	Sié et al.	https://dx.doi.org/10.1093/inthealth/ihaa003	Male, Female	Yes	Religion	Muslim, Catholic, Protestant, Animist, Other	No			Yes	Household resources score
Evidence of improving antiretroviral therapy treatment delays: an analysis of eight years of programmatic outcomes in Blantyre, Malawi	2013	Sloan et al.	http://www.biomedcentral.com/1471-2458/13/490	Men, Women	No			No			No	
Low use of long-lasting insecticidal nets for malaria prevention in south-central Ethiopia: A community-based cohort study	2019	Solomon et al.	https://doi.org/10.1371/journal.pone.0210578	Male, Female	No			Yes	Educational status of head of household	Illiterate, Read and write, Primary, Secondary and above	Yes	Household wealth index

1. BIBLIOGRAPHIC SOURCE												
**Title**	**Year**	**First author**	**UHL**	**Measure**	**Religion**	**Name (acronym)**	**Measure**	**Education**	**Name (acronym)**	**Measure**	**Socioeconomic status (SES)**	**Name (acronym)**
Empowerment and use of modern contraceptive methods among married women in Burkina Faso: a multilevel analysis	2021	Some et al.	https://dx.doi.org/10.1186/s12889-021-11541-x		No			Yes	Women's education level	No education, Primary, Secondary/ higher	Yes	Household wealth
Evaluation of the 2011 long-lasting, insecticide-treated net distribution for universal coverage in Togo	2013	Stevens et al.	http://www.malariajournal.com/content/12/1/162	Male, female	No			No			No	
On the way to universal coverage of maternal services in Iringa rural District in Tanzania. Who is yet to be reached?	2016	Straneo et al.	https://doi.org/10.4314/ahs.v16i2.10	Female, male	No			Yes	1) Maternal education (years); 2) Household's head education (years)	0-6, ≥7	Yes	Socio-economic status/quintiles

1. BIBLIOGRAPHIC SOURCE												
**Title**	**Year**	**First author**	**UHL**	**Measure**	**Religion**	**Name (acronym)**	**Measure**	**Education**	**Name (acronym)**	**Measure**	**Socioeconomic status (SES)**	**Name (acronym)**
Evaluation of long-lasting insecticidal net distribution through schools in Southern Tanzania	2022	Stuck et al.	https://doi.org/10.1093/heapol/czab140		No			No			No	
Not all inequalities are equal: differences in coverage across the continuum of reproductive health services	2019	Sully et al.	https://dx.doi.org/10.1136/bmjgh-2019-001695		No			No			Yes	Household wealth

1. BIBLIOGRAPHIC SOURCE												
**Title**	**Year**	**First author**	**UHL**	**Measure**	**Religion**	**Name (acronym)**	**Measure**	**Education**	**Name (acronym)**	**Measure**	**Socioeconomic status (SES)**	**Name (acronym)**
Determinants of long-lasting insecticidal net ownership and utilization in malaria transmission regions: Evidence from Zimbabwe Demographic and Health Surveys	2019	Tapera	https://doi.org/10.1186/s12936-019-2912-x	Male, Female	No			Yes	Education level	No education, primary, secondary, Higher	Yes	Wealth index
Sociodemographic inequities in cervical cancer screening, treatment and care amongst women aged at least 25 years: evidence from surveys in Harare, Zimbabwe	2019	Tapera et al.	https://doi.org/10.1186/s12889-019-6749-6		Yes	Religion	Roman Catholic, Protestant, Pentecostal, Apostolic sect, Other	Yes	Education	Primary, secondary, higher, none	Yes	1) Personal income; 2) household income; 3) wealth (poor, middle and rich).

1. BIBLIOGRAPHIC SOURCE												
**Title**	**Year**	**First author**	**UHL**	**Measure**	**Religion**	**Name (acronym)**	**Measure**	**Education**	**Name (acronym)**	**Measure**	**Socioeconomic status (SES)**	**Name (acronym)**
Factors associated to bed net use in Cameroon: a retrospective study in Mfou health district in the Centre Region	2012	Tchinda et al.	http://www.panafrican-med-journal.com/content/article/12/112/full/	Male, Female	No			Yes	Education level	Primary/none, Secondary, University	No	
Cervical cancer screening uptake and correlates among HIV-infected women: a cross-sectional survey in Cote d'Ivoire, West Africa	2019	Tchounga et al.	https://dx.doi.org/10.1136/bmjopen-2019-029882		No			Yes	Level of Eductaion	No formal education, primary level, secondary education, university level	No	

1. BIBLIOGRAPHIC SOURCE												
**Title**	**Year**	**First author**	**UHL**	**Measure**	**Religion**	**Name (acronym)**	**Measure**	**Education**	**Name (acronym)**	**Measure**	**Socioeconomic status (SES)**	**Name (acronym)**
Duration and determinants of delayed tuberculosis diagnosis and treatment in high-burden countries: a mixed-methods systematic review and meta-analysis	2021	Teo et al.	https://doi.org/10.1186/s12931-021-01841-6	Male, Female	No			Yes	Level of education	Low education	Yes	Finances
Skilled delivery inequality in Ethiopia: To what extent are the poorest and uneducated mothers benefiting?	2017	Tesfaye et al.	https://doi.org/10.1186/s12939-017-0579-x		No			Yes	Educational level	No education, primary and secondary and above	Yes	Wealth index

1. BIBLIOGRAPHIC SOURCE												
**Title**	**Year**	**First author**	**UHL**	**Measure**	**Religion**	**Name (acronym)**	**Measure**	**Education**	**Name (acronym)**	**Measure**	**Socioeconomic status (SES)**	**Name (acronym)**
Investigating the association between pregnancy intention and insecticide-treated bed net (ITN) use: A cross-sectional study of pregnant women in Rwanda	2015	Thogarapalli et al.	https://doi.org/10.1007/s10389-015-0676-5		No			Yes	Education level	No formal education, primary education, secondary education and abobe	Yes	LDHS wealth index
Success of Senegal's first nationwide distribution of long-lasting insecticide-treated nets to children under five - Contribution toward universal coverage	2011	Thwing et al.	http://www.malariajournal.com/content/10/1/86		No			No			No	
Declines in Malaria Burden and all-cause child mortality following increases in control interventions in Senegal, 2005-2010	2017	Thwing et al.	https://doi.org/10.4269/ajtmh.16-0953		No			No			Yes	Socio economiic quintile

1. BIBLIOGRAPHIC SOURCE												
**Title**	**Year**	**First author**	**UHL**	**Measure**	**Religion**	**Name (acronym)**	**Measure**	**Education**	**Name (acronym)**	**Measure**	**Socioeconomic status (SES)**	**Name (acronym)**
Individual-level and community-level determinants of cervical cancer screening among Kenyan women: a multilevel analysis of a Nationwide survey	2017	Tiruneh et al.	https://doi.org/10.1186/s12905-017-0469-9		No			Yes	Education level	No education, primary, secondary and higher	Yes	Household assets
Evaluation of the coverage and effective use rate of long-lasting insecticidal nets after nation-wide scale up of their distribution in Benin	2013	Tokponnon et al.	https://doi.org/10.1186/1756-3305-6-265	Male, Female	No			No			No	
Factors associated with the upsurge in the use of delivery care services in Sierra Leone	2020	Tsawe & Susuman	https://doi.org/10.1016/j.puhe.2019.11.002		No			Yes	Education level	No education, primary, secondary+	Yes	Household wealth

1. BIBLIOGRAPHIC SOURCE												
**Title**	**Year**	**First author**	**UHL**	**Measure**	**Religion**	**Name (acronym)**	**Measure**	**Education**	**Name (acronym)**	**Measure**	**Socioeconomic status (SES)**	**Name (acronym)**
Which family members use the best nets? An analysis of the condition of mosquito nets and their distribution within households in Tanzania	2010	Tsuang et al.	http://www.malariajournal.com/content/9/1/211	Male, Female	No			No			No	
Utilization of insecticide treated nets among pregnant women in enugu, South Eastern Nigeria	2013	Ugwu et al.	https://pubmed.ncbi.nlm.nih.gov/23771448/		No			Yes	Educational lecel	Tertiary education, secondary education, primary education and no formal education	Yes	Social class

1. BIBLIOGRAPHIC SOURCE												
**Title**	**Year**	**First author**	**UHL**	**Measure**	**Religion**	**Name (acronym)**	**Measure**	**Education**	**Name (acronym)**	**Measure**	**Socioeconomic status (SES)**	**Name (acronym)**
Finding the gap: Revealing local disparities in coverage of maternal, newborn and child health services in South Sudan using lot quality assurance sampling	2015	Valadez et al.	https://doi.org/10.1111/tmi.12613		No			No			No	
Group Medical Visit and Microfinance Intervention for Patients With Diabetes or Hypertension in Kenya	2021	Vedanthan et al.	https://doi.org/10.1016/j.jacc.2021.03.002	Women/Male	No			No			Yes	Baseline wealth category

1. BIBLIOGRAPHIC SOURCE												
**Title**	**Year**	**First author**	**UHL**	**Measure**	**Religion**	**Name (acronym)**	**Measure**	**Education**	**Name (acronym)**	**Measure**	**Socioeconomic status (SES)**	**Name (acronym)**
Equity in Maternal Health in South Africa: Analysis of Health Service Access and Health Status in a National Household Survey	2013	Wabiri et al.	https://doi.org/10.1371/journal.pone.0073864		No			Yes	Highest education level	None or Grade 0-3, Gr4-7, Gr8-11, Gr12, Tertially	Yes	Socio economic quintile
Persisting Regional Disparities in Modern Contraceptive Use and Unmet Need for Contraception among Nigerian Women	2019	Wang & Cao	https://doi.org/10.1155/2019/9103928		Yes	Religious affiliation	Christian/Islam, others	No			Yes	Wealth index

1. BIBLIOGRAPHIC SOURCE												
**Title**	**Year**	**First author**	**UHL**	**Measure**	**Religion**	**Name (acronym)**	**Measure**	**Education**	**Name (acronym)**	**Measure**	**Socioeconomic status (SES)**	**Name (acronym)**
Effective coverage of facility delivery in Bangladesh, Haiti, Malawi, Nepal, Senegal, and Tanzania	2019	Wang et al.	https://doi.org/10.1371/journal.pone.0217853		No			No			No	
Use of long-lasting insecticide-treated bed nets in a population with universal coverage following a mass distribution campaign in Uganda	2016	Wanzira et al.	https://doi.org/10.1186/s12936-016-1360-0		No			No			No	

1. BIBLIOGRAPHIC SOURCE												
**Title**	**Year**	**First author**	**UHL**	**Measure**	**Religion**	**Name (acronym)**	**Measure**	**Education**	**Name (acronym)**	**Measure**	**Socioeconomic status (SES)**	**Name (acronym)**
Long-lasting insecticide-treated bed net ownership and use among children under five years of age following a targeted distribution in central Uganda	2014	Wanzira et al.	https://doi.org/10.1186/1475-2875-13-185		No			No			Yes	Wealth quintile
Evaluation of a national universal coverage campaign of long-lasting insecticidal nets in a rural district in north-west Tanzania	2012	West et al.	https://doi.org/10.1186/1475-2875-11-273		No			No			Yes	Percentile of SEs
The impact of renewing long-lasting insecticide-treated nets in the event of malaria resurgence: Lessons from 10 years of net use in dielmo, Senegal	2021	Wotodjo et al.	https://doi.org/10.4269/AJTMH.20-0127		No			No			No	

1. BIBLIOGRAPHIC SOURCE												
**Title**	**Year**	**First author**	**UHL**	**Measure**	**Religion**	**Name (acronym)**	**Measure**	**Education**	**Name (acronym)**	**Measure**	**Socioeconomic status (SES)**	**Name (acronym)**
Wealth and Education Inequities in Maternal and Child Health Services Utilization in Rural Ethiopia	2022	Wuneh et al.	https://doi.org/10.3390/ijerph19095421		No			Yes	Women's educational level	No education (not attended formal education), educated (primary or above)	Yes	Household wealth (created by dividing the household wealth index into three equal tertiles (Tertile 1, Tertile 2, and Tertile 3)
Inequalities in maternal health care utilization in Benin: A population based cross-sectional study	2018	Yaya et al.	https://doi.org/10.1186/s12884-018-1846-6	Male, Female	Yes	Religious beliefs	Christianity, Islam, traditional, Other religion	Yes	Educational attainment	No formal education, primary, secondary, higher education	Yes	Wealth quintiles

1. BIBLIOGRAPHIC SOURCE												
**Title**	**Year**	**First author**	**UHL**	**Measure**	**Religion**	**Name (acronym)**	**Measure**	**Education**	**Name (acronym)**	**Measure**	**Socioeconomic status (SES)**	**Name (acronym)**
Long-lasting insecticide-treated bed net ownership, utilization and associated factors among school-age children in Dara Mallo and Uba Debretsehay districts, Southern Ethiopia	2020	Zerdo et al.	https://dx.doi.org/10.1186/s12936-020-03437-9	1-2) Male, Female	No			Yes	1) Educational status of household head; 2) Educational status of mother; 3) Education level of mother	1-2) Illiterate, Literate; 3) < Grade 7, ≥ Grade 7	Yes	Wealth index of the household in quartile
Trends and projections of universal health coverage indicators in Ghana, 1995-2030: A national and subnational study	2019	Zhang et al.	https://doi.org/10.1371/journal.pone.0209126		No			No			Yes	Wealth quintiles

1. BIBLIOGRAPHIC SOURCE												
**Title**	**Year**	**First author**	**UHL**	**Measure**	**Religion**	**Name (acronym)**	**Measure**	**Education**	**Name (acronym)**	**Measure**	**Socioeconomic status (SES)**	**Name (acronym)**
Developing Malawi's Universal Health Coverage Index	2022	Mchenga et al.	https://doi.org/10.3389/frhs.2021.786186		No			No			Yes	Wealth index
Socioeconomic Factors Associated with Compliance with Mass Drug Administration for Lymphatic Filariasis Elimination in Kenya: Descriptive Study Results	2012	Njomo et al.	https://www.proquest.com/docview/1115911778?pq-origsite=gscholar&fromopenview=true		Yes	Religion	Christian, Islam, Non-practicing	No			No	

1. BIBLIOGRAPHIC SOURCE												
Title	Year	First author	UHL	Measure	Religion	Name (acronym)	Measure	Education	Name (acronym)	Measure	Socioeconomic status (SES)	Name (acronym)
Equality analysis of main health indicators among children under 5 years in Uganda	2019	Elduma	http://dx.doi.org/10.4314/ejhs.v29i2.8	Male, Female	No			Yes	Parent education level	No education/ Primary education/ Secondary education	Yes	Economic status

1. BIBLIOGRAPHIC SOURCE												
**Title**	**Year**	**First author**	**UHL**	**Measure**	**Social capital or ressources**	**Name (acronym)**	**Measure**	**Age**	**Name (acronym)**	**Measure**	**Disability**	**Name (acronym)**
Reproductive Plans And Utilization of Contraceptives Among Women Living With HIV	2019	Adeleye et al.	https://dx.doi.org/10.21106/ijma.277		No			No			No	
Provision of immediate postpartum contraception to women living with HIV in the Eastern Cape, South Africa; a cross-sectional analysis	2020	Adeniyi et al.	https://doi.org/10.1186/s12978-020-01049-9		Yes	Marital status	Single/Married/Cohabiting	Yes	Age	14/-24/25-34/35-44	No	
Moving Up the Sanitation Ladder: A Study of the Coverage and Utilization of Improved Sanitation Facilities and Associated Factors Among Households in Southern Ethiopia	2022	Afework et al.	https://doi.org/10.1177/11786302221080825	Lower/Middle/Upper	No			Yes	Age of household head	20-35/36-49/>50	No	

1. BIBLIOGRAPHIC SOURCE												
**Title**	**Year**	**First author**	**UHL**	**Measure**	**Social capital or ressources**	**Name (acronym)**	**Measure**	**Age**	**Name (acronym)**	**Measure**	**Disability**	**Name (acronym)**
Maternal health care services utilisation in the context of 'Abiye' (safe motherhood) programme in Ondo State, Nigeria	2020	Ajayi et al.	https://doi.org/10.1186/s12889-020-08512-z	Low/Middle/High; Poorer/Middle/Richer/Richest	No			Yes	Age	15-24/25-34/35-39	No	
Slums, women and sanitary living in South-South Nigeria	2021	Akpabio et al.	https://doi.org/10.1007/s10901-020-09802-z		No			No			No	
Leaving no child behind: Decomposing socioeconomic inequalities in child health for india and south africa	2021	Alaba et al.	https://doi.org/10.3390/ijerph1813714	Poorest/Poorer/Middle/Richer/Richest	No			Yes	Mean age of mother	Continuous variable	No	

1. BIBLIOGRAPHIC SOURCE												
**Title**	**Year**	**First author**	**UHL**	**Measure**	**Social capital or ressources**	**Name (acronym)**	**Measure**	**Age**	**Name (acronym)**	**Measure**	**Disability**	**Name (acronym)**
Access to skilled attendant at birth and the coverage of the third dose of diphtheria-tetanus-pertussis vaccine across 14 West African countries - An equity analysis	2020	Alhassan et al.	https://doi.org/10.1186/s12939-020-01204-5	Poorest - Richest	No			No			No	
Trends and correlates of maternal, newborn and child health services utilization in primary healthcare facilities: An explorative ecological study using DHIMSII data from one district in the Volta region of Ghana	2020	Alhassan et al.	https://doi.org/10.1186/s12884-020-03195-1		No			No			No	

1. BIBLIOGRAPHIC SOURCE												
**Title**	**Year**	**First author**	**UHL**	**Measure**	**Social capital or ressources**	**Name (acronym)**	**Measure**	**Age**	**Name (acronym)**	**Measure**	**Disability**	**Name (acronym)**
Determinants of equity in utilization of maternal health services in Butajira, Southern Ethiopia	2012	Aliy & Mariam	https://www.ajol.info/index.php/ejhd/article/view/116114	less than 1000 birha /1000 or more	No			Yes	Age of woman	below 35 years/35 and above	No	
Determinants of insecticide treated nets use among youth corp members in Edo State, Nigeria	2011	Amoran O. E. et al	http://www.biomedcentral.com/1471-2458/11/728		No			Yes	Age	28-24/>24	No	

1. BIBLIOGRAPHIC SOURCE												
**Title**	**Year**	**First author**	**UHL**	**Measure**	**Social capital or ressources**	**Name (acronym)**	**Measure**	**Age**	**Name (acronym)**	**Measure**	**Disability**	**Name (acronym)**
Towards achievement of Sustainable Development Goal 3: multilevel analyses of demographic and health survey data on health insurance coverage and maternal healthcare utilisation in sub-Saharan Africa	2022	Amu et al.	https://doi.org/10.1093/inthealth/ihac017	Poorest/Poor/Middle/Richer/Richest	Yes	Marital status	Never married/married/cohabiting/widow/divorced/separated	Yes	Maternal age	15-19/20-24/25-29/30-34/35-39/40-44/45-49	No	

1. BIBLIOGRAPHIC SOURCE												
**Title**	**Year**	**First author**	**UHL**	**Measure**	**Social capital or ressources**	**Name (acronym)**	**Measure**	**Age**	**Name (acronym)**	**Measure**	**Disability**	**Name (acronym)**
Equity and access to maternal and child health services in Ghana a cross-sectional study	2021	Anarwat et al.	https://doi.org/10.1186/s12913-021-06872-9	Poorest/Poor/Middle/Richer/Richest	No			No			No	
A subnational profiling analysis reveals regional differences as the main predictor of ITN ownership and use in Nigeria	2019	Andrada et al.	https://doi.org/10.1186/s12936-019-2816-9	Lowest/Second/Third/Fourth/Highest	No			No			No	
Inequality trends in maternal health services for young Ghanaian women with childbirth history between 2003 and 2014	2017	Asamoah & Agardh	https://doi.org/10.1136/bmjopen-2016-011663	Poor/Average/Rich	No			No			No	

1. BIBLIOGRAPHIC SOURCE												
**Title**	**Year**	**First author**	**UHL**	**Measure**	**Social capital or ressources**	**Name (acronym)**	**Measure**	**Age**	**Name (acronym)**	**Measure**	**Disability**	**Name (acronym)**
Leaving no one behind: Lessons from implementation of policies for universal HIV treatment to universal health coverage	2020	Assefa et al.	https://doi.org/10.1186/s12992-020-00549-4		No			Yes	Age	Adult/Children	No	
Inequalities in child immunization coverage in Ghana: evidence from a decomposition analysis	2018	Asuman et al.	https://doi.org/10.1186/s13561-018-0193-7	Low/Middle/High	Yes	Marital status	marital or consensus union	Yes	Age category	12-23/24-35/36-47/48-59	No	
A reassessment of global antenatal care coverage for improving maternal health using sub-Saharan Africa as a case study	2018	Ataguba	https://dx.doi.org/10.1371/journal.pone.0204822	Quintiles 1/2/3/4/5	No			No			No	

1. BIBLIOGRAPHIC SOURCE													
**Title**	**Year**	**First author**	**UHL**	**Measure**	**Social capital or ressources**	**Name (acronym)**	**Measure**	**Age**	**Name (acronym)**	**Measure**	**Disability**	**Name (acronym)**	
Socio-economic inequality in maternal health care utilization in Sub-Saharan Africa: Evidence from Togo	2021	Atake	http://dx.doi.org/10.1002/hpm.3083	Very poor/poor/Average/Rich/Very rich	No			Yes	Woman's age	15-19/20-29/30-39/40-49	No		
Poor prenatal service utilization and pregnancy outcome in a tertiary health facility in southwest Nigeria	2020	Awoleke & Olofinbiyi	https://doi.org/10.11604/pamj.2020.35.28.20426		No			Yes	Woman's age	<30, >=30	No		
Correlates of intra-household ITN use in Liberia: A multilevel analysis of household survey data	2016	Babalola et al.	http://dx.doi.org/10.1371/journal.pone.0158331	Quintiles 1/2/3/4/5	No			Yes	Household members	0–4 years, 5–17 years, and adults aged 18 years and above	No		

1. BIBLIOGRAPHIC SOURCE												
**Title**	**Year**	**First author**	**UHL**	**Measure**	**Social capital or ressources**	**Name (acronym)**	**Measure**	**Age**	**Name (acronym)**	**Measure**	**Disability**	**Name (acronym)**
Prevalence and determinants of maternal healthcare utilisation among young women in sub-Saharan Africa: cross-sectional analyses of demographic and health survey data	2022	Bain et al.	https://doi.org/10.1186/s12889-022-13037-8	Quintiles 1/2/3/4/5	Yes	Marital status	Never married/married/cohabiting/widow/divorced/separated	Yes	Maternal age, Partner's age	Maternal age (15-24, 20-24) Partner's age (15-24, 25-34, 35-44, 45+)	No	

1. BIBLIOGRAPHIC SOURCE												
**Title**	**Year**	**First author**	**UHL**	**Measure**	**Social capital or ressources**	**Name (acronym)**	**Measure**	**Age**	**Name (acronym)**	**Measure**	**Disability**	**Name (acronym)**
Factors influencing timing and frequency of antenatal care in Uganda	2011	Bbaale	https://www.ncbi.nlm.nih.gov/pmc/articles/PMC3562883/pdf/AMJ-04-431.pdf	Poorest/poor/Medium/Rich/Very rich	No			Yes	Maternal age	15-19/20-24/25-29/30-34/35-39/40-44/45-49	No	
Maternal education and childbirth care in Uganda	2011	Bbaale & Guloba	https://www.ncbi.nlm.nih.gov/pmc/articles/PMC3562941/pdf/AMJ-04-389.pdf	Poorest/poor/Medium/Rich/Very rich	No			Yes	Maternal age	15-19/20-24/25-29/30-34/35-39/40-44/45-50	No	

1. BIBLIOGRAPHIC SOURCE												
**Title**	**Year**	**First author**	**UHL**	**Measure**	**Social capital or ressources**	**Name (acronym)**	**Measure**	**Age**	**Name (acronym)**	**Measure**	**Disability**	**Name (acronym)**
A quantitative analysis of food insecurity and other barriers associated with ART nonadherence among women in rural communities of Eswatini	2021	Becker et al.	https://doi.org/10.1371/journal.pone.0256277	<=500, 500+	Yes	Community support, Marital status	Yes/No, Married or living with partner/Not married	Yes	Age	Age in Years	No	
Two decades of antenatal and delivery care in Uganda: a cross-sectional study using Demographic and Health Surveys	2018	Benova et al.	https://doi.org/10.1186/s12913-018-3546-3	Poorest/poor/middle/Rich/Very rich	No			No			No	
Utilization of sexual and reproductive health services in ethiopia - Does it affect sexual activity among high school students?	2015	Bilal et al.	https://doi.org/10.1016/j.srhc.2014.09.009	Low/Middle/High/Very High	No			No			No	

1. BIBLIOGRAPHIC SOURCE												
Title	Year	First author	UHL	Measure	Social capital or ressources	Name (acronym)	Measure	Age	Name (acronym)	Measure	Disability	Name (acronym)
Addressing the huge poor–rich gap of inequalities in accessing safe childbirth care: A first step to achieving universal maternal health coverage in Tanzania	2021	Bintabara	https://doi.org/10.1371/journal.pone.0246995	Poorest/Poor/Middle/Richer/Richest	Yes	Marital status	No spouse/Living with a spouse	Yes	Maternal age	15-19/20-24/25-29/30-34/35-39/40-44/45-50	No	
Twelve-year persistence of inequalities in antenatal care utilisation among women in Tanzania: A decomposition analysis of population-based cross-sectional surveys	2021	Bintabara & Basinda	https://doi.org/10.1136/bmjopen-2020-040450	Poorest/Poor/Middle/Richer/Richest	Yes	Marital status	No spouse/Living with a spouse	Yes	Maternal age	15-19/20-24/25-29/30-34/35-39/40-44/45-50	No	
Socio-demographic and economic inequalities in modern contraception in 11 low- And middle-income countries: An analysis of the PMA2020 surveys	2020	Blumenberg et al.	https://doi.org/10.1186/s12978-020-00931-w	Quintiles 1/2/3/4/5	No			Yes	Age	15-17/18-19/20-34/35-49	No	

1. BIBLIOGRAPHIC SOURCE												
**Title**	**Year**	**First author**	**UHL**	**Measure**	**Social capital or ressources**	**Name (acronym)**	**Measure**	**Age**	**Name (acronym)**	**Measure**	**Disability**	**Name (acronym)**
A decomposition analysis of change in skilled birth attendants, 2003 to 2008, Ghana demographic and health surveys	2014	Bosomprah et al.	https://doi.org/10.1186/s12884-014-0415-x	Poorest/Second/Middle/Fourth/Richest	No			Yes	Age	<20/20-34/35-49	No	
Slow and Steady can Still Win the Race': Childhood Vaccination Experience of Migrant Ebira Women Within the Health System in Ekiti State, Nigeria	2021	Olakanmi-Falade & Awoleke	https://www.ojhas.org/issue79/2021-3-3.html	≤19,999/20,000-39,000/40,000-59,000/60,000 and above	Yes	Marital status	Single/Married	Yes	Age	<=19/20-29/30-39/>=40	No	
Antiretroviral therapy in Walvis Bay, Namibia	2016	Callaghan	https://hdl.handle.net/1807/70825		No			No			No	

1. BIBLIOGRAPHIC SOURCE													
**Title**	**Year**	**First author**	**UHL**	**Measure**	**Social capital or ressources**	**Name (acronym)**	**Measure**	**Age**	**Name (acronym)**	**Measure**	**Disability**	**Name (acronym)**
Family planning, antenatal and delivery care: Cross-sectional survey evidence on levels of coverage and inequalities by public and private sector in 57 low- and middle-income countries	2016	Campbell et al.	https://doi.org/10.1111/tmi.12681	Poorest/Poor/Middle/Richer/Richest	No			No			No	

1. BIBLIOGRAPHIC SOURCE												
**Title**	**Year**	**First author**	**UHL**	**Measure**	**Social capital or ressources**	**Name (acronym)**	**Measure**	**Age**	**Name (acronym)**	**Measure**	**Disability**	**Name (acronym)**
Use of family planning and child health services in the private sector: An equity analysis of 12 DHS surveys	2018	Chakraborty & Sprockett	https://doi.org/10.1186/s12939-018-0763-7	Poorest/Poor/Middle/Richer/Richest	No			No			No	
Meeting demand for family planning within a generation: prospects and implications at country level	2015	Choi et al.	https://dx.doi.org/10.3402/gha.v8.29734	Poorest/Poor/Middle/Richer/Richest	No			No			No	
A Longitudinal Analysis of Mosquito Net Ownership and Use in an Indigenous Batwa Population after a Targeted Distribution	2016	Clark et al.	https://doi.org/10.1371/journal.pone.0154808		No			Yes	Age groups	Under five, Aged 6-12, 13-24, over 35	No	

1. BIBLIOGRAPHIC SOURCE												
**Title**	**Year**	**First author**	**UHL**	**Measure**	**Social capital or ressources**	**Name (acronym)**	**Measure**	**Age**	**Name (acronym)**	**Measure**	**Disability**	**Name (acronym)**
Malaria prevalence and long-lasting insecticidal net use in rural western Uganda: results of a cross-sectional survey conducted in an area of highly variable malaria transmission intensity	2021	Cote et al.	https://doi.org/10.1186/s12936-021-03835-7		No			No			No	
Women's Sexual Empowerment and Contraceptive Use in Ghana	2012	Crissman et al.	https://doi.org/10.1111/j.1728-4465.2012.00318.x	Quintiles 1/2/3/4/5	No			Yes	Age	15-24/25-34/35-49	No	
Socio-economic and demographic factors associated with reproductive and child health preventive care in Mozambique: a cross-sectional study	2020	Daca et al.	https://doi.org/10.1186/s12939-020-01303-3	Poorest/Poorer/Middle/Rich/Richest	Yes	Marital status	Single/Married/Other	Yes	Age	15-24/25-39/40-49	No	

1. BIBLIOGRAPHIC SOURCE													
**Title**	**Year**	**First author**	**UHL**	**Measure**	**Social capital or ressources**	**Name (acronym)**	**Measure**	**Age**	**Name (acronym)**	**Measure**	**Disability**	**Name (acronym)**
Assessing the contextual effect of community in the utilization of postnatal care services in Ghana	2021	Dankwah et al.	https://doi.org/10.1186/s12913-020-06028-1	Poor, Middle, Rich	Yes	Marital status	Single/Widow/divorced/separated/Married	Yes	Age	15 to 49 years	No	
Is South Africa closing the health gaps between districts? Monitoring progress towards universal health service coverage with routine facility data	2021	Day et al.	https://doi.org/10.1186/s12913-021-06171-3	Q1/Q2/Q3/Q4/Q5	No			No			No	
Neglected tropical diseases as a 'litmus test' for universal health coverage? understanding who is left behind and why in mass drug administration: Lessons from four country contexts	2019	Dean et al.	https://doi.org/10.1371/journal.pntd.0007847	Not reported	No			No			Yes	People with disability

1. BIBLIOGRAPHIC SOURCE													
**Title**	**Year**	**First author**	**UHL**	**Measure**	**Social capital or ressources**	**Name (acronym)**	**Measure**	**Age**	**Name (acronym)**	**Measure**	**Disability**	**Name (acronym)**
Preventive Health Service Coverage Among Infants and Children at Six Maternal-Child Health Clinics in Western Kenya: A Cross-Sectional Assessment	2022	Deathe et al.	https://doi.org/10.1007/s10995-021-03271-8		No			No			No	
Distance, difference in altitude and socioeconomic determinants of utilisation of maternal and child health services in Ethiopia: A geographic and multilevel modelling analysis	2021	Defar et al.	https://doi.org/10.1136/bmjopen-2020-042095	Low/Middle /Upper	No			Yes	Age	<20/21-25/26-30/31-35/36-49	No	
Geographic differences in maternal and child health care utilization in four Ethiopian regions; A cross-sectional study	2019	Defar et al.	https://doi.org/10.1186/s12939-019-1079-y	Households with a radio	No			Yes	Age of the child	Mean age of the child in month	No	

1. BIBLIOGRAPHIC SOURCE											
**Title**	**Year**	**First author**	**UHL**	**Measure**	**Social capital or ressources**	**Name (acronym)**	**Measure**	**Age**	**Name (acronym)**	**Measure**	**Disability**	**Name (acronym)**
Ownership and use of insecticide-treated nets in Oromia and Amhara Regional States of Ethiopia twoyears after a nationwide campaign	2011	Deressa et al.	https://doi.org/10.1111/j.1365-3156.2011.02875.x		No			No			No	
Effectiveness of post-campaign, door-to-door, hang-up, and communication interventions to increase long-lasting, insecticidal bed net utilization in Togo (2011-2012): A cluster randomized, control trial	2014	Desrochers et al.	https://doi.org/10.1186/1475-2875-13-260	Q1/Q2/Q3/Q4/Q5	No			No			No	
Immediate postnatal care following childbirth in Ugandan health facilities: An analysis of Demographic and Health Surveys between 2001 and 2016	2021	Dey et al.	https://doi.org/10.1136/bmjgh-2020-004230	Poorest/Poorer/Middle/Richer/Richest	Yes	Marital status	Married or not	Yes	Age group	<20/20-24.9/25-29.9/30-34.9/35-49.9	No	

1. BIBLIOGRAPHIC SOURCE												
**Title**	**Year**	**First author**	**UHL**	**Measure**	**Social capital or ressources**	**Name (acronym)**	**Measure**	**Age**	**Name (acronym)**	**Measure**	**Disability**	**Name (acronym)**
Inequalities in non-communicable diseases and effective responses	2013	Di Cesare et al.	https://doi.org/10.1016/s0140-6736(12)61851-0		No			Yes	Age group	25-34/35-44/45-54/55-64	No	
Insecticide-treated nets ownership and utilization among under-five children following the 2010 mass distribution in Burkina Faso	2014	Diabate et al.	https://dx.doi.org/10.1186/1475-2875-13-353	Ownership of agricultural land, ownership of cattle	No			Yes	Age group (months)	0-11/12-23/24-35/356-47/48-59	No	

1. BIBLIOGRAPHIC SOURCE												
**Title**	**Year**	**First author**	**UHL**	**Measure**	**Social capital or ressources**	**Name (acronym)**	**Measure**	**Age**	**Name (acronym)**	**Measure**	**Disability**	**Name (acronym)**
Impact of mining projects on water and sanitation infrastructures and associated child health outcomes: a multi-country analysis of Demographic and Health Surveys (DHS) in sub-Saharan Africa	2021	Dietler et al.	https://doi.org/10.1186/s12992-021-00723-2	Poorest/Poorer/Middle/Richer/Richest	No			No			No	

1. BIBLIOGRAPHIC SOURCE												
**Title**	**Year**	**First author**	**UHL**	**Measure**	**Social capital or ressources**	**Name (acronym)**	**Measure**	**Age**	**Name (acronym)**	**Measure**	**Disability**	**Name (acronym)**
Predictors of insecticidal net use among internally displaced persons aged 6-59 months in Abuja, Nigeria	2018	Ejembi et al.	http://dx.doi.org/10.11604/pamj.2018.29.136.13322		No			Yes	Age of child (months, Age of mother (years)	Child (<36/36-59) Mother (<=35/>=36)	No	
Changes in Inequality in Use of Maternal Health Care Services: Evidence from Skilled Birth Attendance in Mauritania for the Period 2007-2015	2022	Taleb El Hassen et al.	https://doi.org/10.3390/ijerph19063566	Poorest, Poor, Middle, Rich, Richest	No			Yes	Women's Age	15–24, 25–34, 35–44, 45–49	No	

1. BIBLIOGRAPHIC SOURCE												
**Title**	**Year**	**First author**	**UHL**	**Measure**	**Social capital or ressources**	**Name (acronym)**	**Measure**	**Age**	**Name (acronym)**	**Measure**	**Disability**	**Name (acronym)**
Trend in the use of modern contraception in sub-Saharan Africa: Does women's education matter?	2014	Emina et al.	http://dx.doi.org/10.1016/j.contraception.2014.02.001		No			No			No	

1. BIBLIOGRAPHIC SOURCE												
**Title**	**Year**	**First author**	**UHL**	**Measure**	**Social capital or ressources**	**Name (acronym)**	**Measure**	**Age**	**Name (acronym)**	**Measure**	**Disability**	**Name (acronym)**
Utilization, Predictors and Gaps in the Continuum of Care for Maternal and Newborn Health in Ghana	2021	Enos et al.	https://dx.doi.org/10.21106/ijma.425	No income/low to medium/high income	No			Yes	Age	<21/21-34/35+	No	
ART use and associated factors among HIV positive caregivers of orphans and vulnerable children in Tanzania	2020	Exavery et al.	https://doi.org/10.1186/s12889-020-09361-6	Lowest/Second/Middle/Fourth/Highest	Yes	Marital status	Married or living together, divorced or separated, never been married, widowed	Yes	Age	19-29/30-39/40-49/50-59/60+	Yes	Mentally or physically disabled
Trends and causes of socioeconomic inequalities in maternal healthcare in Ghana, 2003-2014	2019	Fenny et al.	https://doi.org/10.1108/ijse-03-2018-0148	1/2/3/4/5	No			No			No	

1. BIBLIOGRAPHIC SOURCE												
**Title**	**Year**	**First author**	**UHL**	**Measure**	**Social capital or ressources**	**Name (acronym)**	**Measure**	**Age**	**Name (acronym)**	**Measure**	**Disability**	**Name (acronym)**
Determination of the predictive factors of long-lasting insecticide-treated net ownership and utilisation in the Bamenda Health District of Cameroon	2017	Fokam et al.	https://doi.org/10.1186/s12889-017-4155-5		No			Yes	Age group	<5/6-25/26-49/>50	No	
The free caesareans policy in low-income settings: An interrupted time series analysis in Mali (2003-2012)	2014	Fournier et al.	https://doi.org/10.1371/journal.pone.0105130		No			No			No	

1. BIBLIOGRAPHIC SOURCE												
**Title**	**Year**	**First author**	**UHL**	**Measure**	**Social capital or ressources**	**Name (acronym)**	**Measure**	**Age**	**Name (acronym)**	**Measure**	**Disability**	**Name (acronym)**
DO BETTER INSTITUTIONS BROADEN ACCESS TO SANITATION IN SUB-SAHARA AFRICA?	2021	Francois et al.	https://doi.org/10.1111/coep.12512		No			No			No	

1. BIBLIOGRAPHIC SOURCE												
**Title**	**Year**	**First author**	**UHL**	**Measure**	**Social capital or ressources**	**Name (acronym)**	**Measure**	**Age**	**Name (acronym)**	**Measure**	**Disability**	**Name (acronym)**
Ethnic disparities in utilisation of maternal health care services in Ghana: evidence from the 2007 Ghana Maternal Health Survey	2016	Ganle	https://doi.org/10.1080/13557858.2015.1015499		No			No			No	
Risky sexual behaviour and contraceptive use in contexts of displacement: Insights from a cross-sectional survey of female adolescent refugees in Ghana	2019	Ganle et al.	https://doi.org/10.1186/s12939-019-1031-1		No			Yes	Age group	14–16/17–19	No	
Understanding how distance to facility and quality of care affect maternal health service utilization in Kenya and Haiti: A comparative geographic information system study	2019	Gao & Kelley	https://doi.org/10.4081/gh.2019.690		No			Yes	Age group	unclear	No	

1. BIBLIOGRAPHIC SOURCE												
**Title**	**Year**	**First author**	**UHL**	**Measure**	**Social capital or ressources**	**Name (acronym)**	**Measure**	**Age**	**Name (acronym)**	**Measure**	**Disability**	**Name (acronym)**
Gender differences in the use of insecticide-treated nets after a universal free distribution campaign in Kano State, Nigeria: Post-campaign survey results	2013	Garley et al.	https://doi.org/10.1186/1475-2875-12-119	Lowest/Second/Middle/Fourth/Highest	No			Yes	Age group	<5/5-15/15-25/25+	No	
Demand satisfied by modern contraceptive among married women of reproductive age in Kenya	2021	Gichangi et al.	https://doi.org/10.1371/journal.pone.0248393	Q1/Q2/Q3/Q4/Q5	No			Yes	Age group	15-19/20-24/25-29/30-34/35-39/40-44/45-49	No	
LLIN Evaluation in Uganda Project (LLINEUP): Factors associated with ownership and use of long-lasting insecticidal nets in Uganda: A cross-sectional survey of 48 districts ISRCTN17516395 ISRCTN	2018	Gonahasa et al.	https://doi.org/10.1186/s12936-018-2571-3	Poorest/Middle/Least poor	No			Yes	Age group of resident who slept under LLN	<5/5-15/>15	No	

1. BIBLIOGRAPHIC SOURCE												
Title	Year	First author	UHL	Measure	Social capital or ressources	Name (acronym)	Measure	Age	Name (acronym)	Measure	Disability	Name (acronym)
ART adherence and viral suppression are high among most non-pregnant individuals with early-stage, asymptomatic HIV infection: an observational study from Uganda and South Africa	2019	Haberer et al.	https://doi.org/10.1002/jia2.25232	Yes/No	Yes	Married; Instrumental support	Married-Yes/No; Instrumental support-mean score of social support	Yes	Age	Mean age (years)	No	
Low immunization coverage in Wonago district, southern Ethiopia: A community-based cross-sectional study	2019	Hailu et al.	https://doi.org/10.1371/journal.pone.0220144		No			Yes	Mother's age	Age in years	No	

1. BIBLIOGRAPHIC SOURCE													
**Title**	**Year**	**First author**	**UHL**	**Measure**	**Social capital or ressources**	**Name (acronym)**	**Measure**	**Age**	**Name (acronym)**	**Measure**	**Disability**	**Name (acronym)**	
Towards universal health coverage: The role of within-country wealth-related inequality in 28 countries in sub-Saharan Africa	2011	Hosseinpoor et al.	https://doi.org/10.2471/BLT.11.087536	Q1/Q2/Q3/Q4/Q5	No			No			No		

1. BIBLIOGRAPHIC SOURCE												
**Title**	**Year**	**First author**	**UHL**	**Measure**	**Social capital or ressources**	**Name (acronym)**	**Measure**	**Age**	**Name (acronym)**	**Measure**	**Disability**	**Name (acronym)**
Patterns and trends of contraceptive use among sexually active adolescents in Burkina Faso, Ethiopia, and Nigeria: evidence from cross-sectional studies	2015	Hounton et al.	https://doi.org/10.3402/gha.v8.29737	Q1/Q2/Q3/Q4/Q6	Yes	Marital status	Married or not in a union	No			No	
Towards universal health coverage for reproductive health services in Ethiopia: two policy recommendations	2015	Onarheim et al.	https://doi.org/10.1186/s12939-015-0218-3	Poorest/Poorer/Middle/Less-poor/Least-poor	No			No			No	

1. BIBLIOGRAPHIC SOURCE													
**Title**	**Year**	**First author**	**UHL**	**Measure**	**Social capital or ressources**	**Name (acronym)**	**Measure**	**Age**	**Name (acronym)**	**Measure**	**Disability**	**Name (acronym)**
Modern Contraception: Uptake and Correlates among Women of Reproductive Age-Group in a Rural Community of Osun State, Nigeria	2020	Idowu et al.	https://doi.org/10.4314/ejhs.v30i4.8	<=30,000/>31,000	Yes	Marital status	Married/Others	Yes	Age	<=29/>=30	No	
Determinants of geographical inequalities for DTP3 vaccine coverage in sub-Saharan Africa	2020	Ikilezi et al.	https://doi.org/10.1016/j.vaccine.2020.03.005		No			No			No	
Influence of women's decision-making autonomy on antenatal care utilisation and institutional delivery services in Nigeria: evidence from the Nigeria Demographic and Health Survey 2018	2022	Imo	https://doi.org/10.1186/s12884-022-04478-5	Lowest/Middle/Highest	No			Yes	Age	15-24/25-34/35+	No	

1. BIBLIOGRAPHIC SOURCE											
**Title**	**Year**	**First author**	**UHL**	**Measure**	**Social capital or ressources**	**Name (acronym)**	**Measure**	**Age**	**Name (acronym)**	**Measure**	**Disability**	**Name (acronym)**
Individual and community-level determinants of cervical cancer screening in Zimbabwe: a multi-level analyses of a nationwide survey	2022	Isabirye et al.	https://doi.org/10.1186/s12905-022-01881-0	Poorest/Poor/Middle/Rich/Richest	No			Yes	Age group	<30/31-49	No	
Two decades of maternity care fee exemption policies in Ghana: have they benefited the poor?	2016	Johnson et al.	https://doi.org/10.1093/heapol/czv017	Poorest/Poor/Middle/Rich/Richest	No			Yes	Age group	<20/20-34/35+	No	

1. BIBLIOGRAPHIC SOURCE												
**Title**	**Year**	**First author**	**UHL**	**Measure**	**Social capital or ressources**	**Name (acronym)**	**Measure**	**Age**	**Name (acronym)**	**Measure**	**Disability**	**Name (acronym)**
Socioeconomic inequalities in access to skilled birth attendance among urban and rural women in low-income and middle-income countries	2018	Joseph et al.	https://doi.org/10.1136/bmjgh-2018-000898	Poorest/Poor/Middle/Richer/Richest	No			No			No	

1. BIBLIOGRAPHIC SOURCE												
**Title**	**Year**	**First author**	**UHL**	**Measure**	**Social capital or ressources**	**Name (acronym)**	**Measure**	**Age**	**Name (acronym)**	**Measure**	**Disability**	**Name (acronym)**
Investigating the disparities in cervical cancer screening among Namibian women	2015	Kangmennaang et al.	https://doi.org/10.1016/j.ygyno.2015.05.036	Poorest/Poorer/Middle/Richer/Richest	No			Yes	Age	Age in years	No	
Contraceptive use and needs among adolescent women aged 15-19: Regional and global estimates and projections from 1990 to 2030 from a Bayesian hierarchical modelling study	2021	Kantorová et al.	https://doi.org/10.1371/journal.pone.0247479		Yes	Marital status	Married, unmarried	Yes	Women age	Adolescent (15–19 years), reproductive age (15-49 years)	No	
Changes in equity of maternal, newborn, and child health care practices in 115 districts of rural Ethiopia: Implications for the health extension program	2015	Karim et al.	https://doi.org/10.1186/s12884-015-0668-z	Poorest, Medium, Least poor	No			Yes	Age group	15 -19, 20-34, 35-49	No	

1. BIBLIOGRAPHIC SOURCE												
**Title**	**Year**	**First author**	**UHL**	**Measure**	**Social capital or ressources**	**Name (acronym)**	**Measure**	**Age**	**Name (acronym)**	**Measure**	**Disability**	**Name (acronym)**
Contraceptive dynamics during COVID-19 in sub-Saharan Africa: Longitudinal evidence from Burkina Faso and Kenya	2021	Karp et al.	https://doi.org/10.1136/bmjsrh-2020-200944	Lower, middle, high	No			Yes	Age	15–19, 20 - 24, 25–34, 35–49	No	
Long-lasting insecticidal net source, ownership and use in the context of universal coverage: A household survey in eastern Rwanda	2015	Kateera et al.	https://doi.org/10.1186/s12936-015-0915-9	Low, middle, high	No			Yes	1) Age group of all Household members in years; 2) Age group of heald of household in years	1) 0–5, 6–15, >15; 2) 18–30, 31–55, 56+	No	
A quasi-experimental evaluation of an interpersonal communication intervention to increase insecticide-treated net use among children in Zambia	2012	Keating et al.	https://doi.org/10.1186/1475-2875-11-313	Poorest, poor, middle, rich, richest	No			Yes	Age	0-11,12-23, 24-35, 36-47, 48-59 months	No	

1. BIBLIOGRAPHIC SOURCE												
**Title**	**Year**	**First author**	**UHL**	**Measure**	**Social capital or ressources**	**Name (acronym)**	**Measure**	**Age**	**Name (acronym)**	**Measure**	**Disability**	**Name (acronym)**
Assessment of Inequalities in Coverage of Essential Reproductive, Maternal, Newborn, Child, and Adolescent Health Interventions in Kenya	2018	Keats et al.	https://doi.org/10.1001/jamanetworkopen.2018.5152	Quintiles 1-5	No			No			No	
Charting health system reconstruction in post-war Liberia: a comparison of rural vs. remote healthcare utilization	2016	Kentoffioet al.	https://doi.org/10.1186/s12913-016-1709-7		No			No			No	

1. BIBLIOGRAPHIC SOURCE													
**Title**	**Year**	**First author**	**UHL**	**Measure**	**Social capital or ressources**	**Name (acronym)**	**Measure**	**Age**	**Name (acronym)**	**Measure**	**Disability**	**Name (acronym)**	
A Transparent Universal Health Coverage Index with Decomposition by Socioeconomic Groups: Application in Asian and African Settings	2019	Khan et al.	https://doi.org/10.1007/s40258-019-00464-9	poorest (Q1), Q2, Q3, Q4, richest (Q5)	No			No			No		
Effect of user preferences on ITN use: a review of literature and data	2017	Koenker & Yukich	https://doi.org/10.1186/s12936-017-1879-8	Poorest, Poorer, middle, richer, richest	No			Yes	Age of household head	<5, 35-40, 50+	No		

1. BIBLIOGRAPHIC SOURCE												
**Title**	**Year**	**First author**	**UHL**	**Measure**	**Social capital or ressources**	**Name (acronym)**	**Measure**	**Age**	**Name (acronym)**	**Measure**	**Disability**	**Name (acronym)**
Insecticide-treated net use before and after mass distribution in a fishing community along Lake Victoria, Kenya: successes and unavoidable pitfalls	2014	Larson et al.	http://www.malariajournal.com/content/13/1/466	5 (Least poor, 4, 3, 2, 1 (Poorest)	No			Yes	Age	0-5, 5-18, 18-30, 30+	No	
Lifetime Prevalence of Cervical Cancer Screening in 55 Low- and Middle-Income Countries	2020	Lemp et al.	https://doi.org/10.1001/jama.2020.16244	Top40%/bottom 40%	No			Yes	Age group	20-29 years/30-49 years	No	

1. BIBLIOGRAPHIC SOURCE												
**Title**	**Year**	**First author**	**UHL**	**Measure**	**Social capital or ressources**	**Name (acronym)**	**Measure**	**Age**	**Name (acronym)**	**Measure**	**Disability**	**Name (acronym)**
Financial accessibility and user fee reforms for maternal healthcare in five sub-Saharan countries: a quasi-experimental analysis	2016	Leone et al.	https://dx.doi.org/10.1136/bmjopen-2015-009692	Poorest/Below average/Above average/Average/Richest	No			Yes	Age group	20-24/25-29/30-34/35-39/40-44/45-49	No	
Antiretroviral treatment coverage in a rural district in Tanzania--a modeling study using empirical data	2015	Levira et al.	https://doi.org/10.1186/s12889-015-1460-8		No			Yes	Age group	15-19, 20-24, 25-29, 30-34, 35-39, 40-44, 45-49	No	
The extent of universal health coverage for maternal health services in eastern uganda: A cross sectional study	2021	Lindberg et al.	https://dx.doi.org/10.1007/s10995-021-03357-3	Poorest, poorer, poor, less poor, least poor	No			No			No	

1. BIBLIOGRAPHIC SOURCE												
**Title**	**Year**	**First author**	**UHL**	**Measure**	**Social capital or ressources**	**Name (acronym)**	**Measure**	**Age**	**Name (acronym)**	**Measure**	**Disability**	**Name (acronym)**
Freely distributed bed-net use among Chano Mille residents, south Ethiopia: A longitudinal study	2013	Loha et al.	https://doi.org/10.1186/1475-2875-12-23	presence of electricity, watch, TV, radio, mobile phone, refrigerator, separate room used for kitchen, bicycle, agricultural land, livestock, account in bank or credit association and latrine facility	No			Yes	Age group	0-4, 5-14, 15-24, >24	No	
Determinants of hanging and use of ITNs in the context of near universal coverage in Zambia	2012	Macintyre et al.	https://doi.org/10.1093/heapol/czr042	Lowest, Mid-low, Middle, Mid-high, High	No			No			No	

1. BIBLIOGRAPHIC SOURCE												
**Title**	**Year**	**First author**	**UHL**	**Measure**	**Social capital or ressources**	**Name (acronym)**	**Measure**	**Age**	**Name (acronym)**	**Measure**	**Disability**	**Name (acronym)**
Determinants and Consequences of Failure of Linkage to Antiretroviral Therapy at Primary Care Level in Blantyre, Malawi: A Prospective Cohort Study	2012	MacPherson et al.	https://doi.org/10.1371/journal.pone.0044794	Poorest quartile, Next poorest quartile, Next wealthiest quartile, Wealthiest quartile	No			Yes	Age (years) - continuous		No	
Disability and sexual and reproductive health service utilisation in Uganda: an intersectional analysis of demographic and health surveys between 2006 and 2016	2022	Mac-Seing et al.	https://doi.org/10.1186/s12889-022-12708-w	Quintile 1 (poorest), Quintile 2 (poorer), Quintile 3 (middle), Quintile 4 (richer), Quintile 5 (richest)	Yes	Marital status	Married/in unionS, Separated / divorced / widowed	Yes	Age in years	18-19, 20-24, 25-29, 30-34, 35-39, 40 and >	Yes	Disability

1. BIBLIOGRAPHIC SOURCE											
Title	Year	First author	UHL	Measure	Social capital or ressources	Name (acronym)	Measure	Age	Name (acronym)	Measure	Disability	Name (acronym)
Insight into Nigeria's progress towards the universal coverage of reproductive, maternal, newborn and child health services: a secondary data analysis	2022	Mafiana et al.	http://dx.doi.org/10.1136/bmjopen-2022-061595	Q1 (poorest), Q2, Q3, Q4, Q5 (richest)	No			No			No	
Implementation of Urban Health Equity Assessment and Response Tool: a Case of Matsapha, Swaziland	2018	Makadzange et al.	https://doi.org/10.1007/s11524-018-0241-y		No			No			No	
Effects of door-to-door hang-up visits on the use of long-lasting insecticide-treated mosquito nets in the democratic republic of the congo: A cluster randomized controlled trial	2021	Mankadi and Jin	https://doi.org/10.3390/ijerph18179048		No			Yes	Child age (months)		No	

1. BIBLIOGRAPHIC SOURCE												
**Title**	**Year**	**First author**	**UHL**	**Measure**	**Social capital or ressources**	**Name (acronym)**	**Measure**	**Age**	**Name (acronym)**	**Measure**	**Disability**	**Name (acronym)**
Effect of bed net colour and shape preferences on bed net usage: a secondary data analysis of the 2017 Malawi Malaria Indicator Survey	2020	Mategula et al.	https://doi.org/10.1186/s12936-020-03499-9	Poorest, Poorer, Middle, Richer, Richest	No			Yes	Age of household head in years	15-25, 25-35, 35-45, 45+	No	
HIV care coverage among HIV-positive adolescent girls and young women in South Africa: Results from the HERStory Study	2021	Mathews et al.	https://doi.org/10.7196/SAMJ.2021.v111i5.15351	Relatively low socioeconomic group, Relatively high socioeconomic group	No			Yes	Age group (years)	15-19, 20-24	No	
Facilitators and barriers to retention in care under universal antiretroviral therapy (Option B+) for the Prevention of Mother to Child Transmission of HIV (PMTCT): A narrative review	2021	Mbeya Munkhondya et al.	https://doi.org/10.1016/j.ijans.2021.100372		Yes	Social support	Not reported	Yes	Age	Not reported	No	

1. BIBLIOGRAPHIC SOURCE												
**Title**	**Year**	**First author**	**UHL**	**Measure**	**Social capital or ressources**	**Name (acronym)**	**Measure**	**Age**	**Name (acronym)**	**Measure**	**Disability**	**Name (acronym)**
The consequences of declining population access to insecticide-treated nets (ITNs) on net use patterns and physical degradation of nets after 22 months of ownership	2021	Mboma et al.	https://doi.org/10.1186/s12936-021-03686-2		No			Yes	Age in years	Under 5, 5-14, 15-24, 25-64, 64+	No	
Mosquito net coverage in years between mass distributions: a case study of Tanzania, 2013	2018	Mboma et al.	https://doi.org/10.1186/s12936-018-2247-z	Poorest, Second poorerest, Midium, Wealthier, Wealthiest	No			No			No	
Factors associated with contraceptive use in Tigray, North Ethiopia	2017	Medhanyie et al.	https://doi.org/10.1186/s12978-017-0281-x		Yes	Discuss with husband about family planning use	Yes, No	Yes	Age category of women	15-19, 20-24, 25-29, 30-34, 35-39, 40-49	No	

1. BIBLIOGRAPHIC SOURCE												
Title	Year	First author	UHL	Measure	Social capital or ressources	Name (acronym)	Measure	Age	Name (acronym)	Measure	Disability	Name (acronym)
Adolescent sexual and reproductive health in sub-Saharan Africa: who is left behind?	2020	Melesse et al.	https://doi.org/10.1136/bmjgh-2019-002231	Poorest, Wealthiest	No			No			No	
Sub-national levels and trends in contraceptive prevalence, unmet need, and demand for family planning in Nigeria with survey uncertainty	2019	Mercer et al.	https://doi.org/10.1186/s12889-019-8043-z		No			Yes	Age	15-24, 25-49	No	

1. BIBLIOGRAPHIC SOURCE												
**Title**	**Year**	**First author**	**UHL**	**Measure**	**Social capital or ressources**	**Name (acronym)**	**Measure**	**Age**	**Name (acronym)**	**Measure**	**Disability**	**Name (acronym)**
Exploring inequities in skilled care at birth among migrant population in a metropolitan city Addis Ababa, Ethiopia; A qualitative study	2014	Mirkuzie	http://www.equityhealthj.com/content/13/1/110		Yes	Social support	Strong, little	Yes	Age group 18-25 years	Yes, No	No	
Factors associated with the use of mosquito bed nets: results from two cross-sectional household surveys in Zambezia Province, Mozambique	2016	Moon et al.	https://doi.org/10.1186/s12936-016-1250-5	<1000 meticais per month, 1000+ meticais per month	No			Yes	Age of respondent	Multiple recording	No	
Sociocultural and Institutional Constraints to Family Planning Uptake Among Migrant Female Head Porters in Madina, a Suburb of Accra, Ghana	2021	Munemo et al.	https://doi.org/10.1177/088610992095441 9		Yes	Male partner support	Yes, No	No			No	

1. BIBLIOGRAPHIC SOURCE												
**Title**	**Year**	**First author**	**UHL**	**Measure**	**Social capital or ressources**	**Name (acronym)**	**Measure**	**Age**	**Name (acronym)**	**Measure**	**Disability**	**Name (acronym)**
Reframing non-communicable diseases and injuries for equity in the era of universal health coverage: Findings and recommendations from the Kenya NCDI poverty commission	2021	Mwangi et al.	https://doi.org/10.5334/aogh.3085	Q1 (Poorest), Q2, Q3, Q4, Q5 (Richest)	No			No			No	

1. BIBLIOGRAPHIC SOURCE													
**Title**	**Year**	**First author**	**UHL**	**Measure**	**Social capital or ressources**	**Name (acronym)**	**Measure**	**Age**	**Name (acronym)**	**Measure**	**Disability**	**Name (acronym)**
Inequities and their determinants in coverage of maternal health services in Burkina Faso	2018	Mwase et al.	https://doi.org/10.1186/s12939-018-0770-8	Poorest, 2, 3, 4, Last poor	Yes	Marital Status	Married, Not married	Yes	Age	15–20, 21-29, 30-39,, 40+	No	

1. BIBLIOGRAPHIC SOURCE												
**Title**	**Year**	**First author**	**UHL**	**Measure**	**Social capital or ressources**	**Name (acronym)**	**Measure**	**Age**	**Name (acronym)**	**Measure**	**Disability**	**Name (acronym)**
A Cross-Sectional Study on Hypertension Medication Adherence in a High-Burden Region in Namibia: Exploring Hypertension Interventions and Validation of the Namibia Hill-Bone Compliance Scale	2022	Nakwafila et al.	https://doi.org/10.3390/ijerph19074416	<500, 500–1999, 2000–4999, 5000–9999, 10,000+	Yes	1) Marital status; 2) Family and friends encourage taking of medication; 3) Family and friends provide company during follow ups	1) Single, Married/Cohabiting, Separated/Divorced; 2-3) No, Yes	Yes	Age (years)	20–29, 30–39, 40–49, 50–59, 60+	No	
Assessing Adherence to Antihypertensive Therapy in Primary Health Care in Namibia: Findings and Implications	2017	Nashilongo et al.	https://doi.org/10.1007/s10557-017-6756-8		Yes	1) Marital status; 2) Has support buddy	1) Married, Single; 2) Yes, No	Yes	Patient's age (years)	<40, >40	No	

1. BIBLIOGRAPHIC SOURCE													
**Title**	**Year**	**First author**	**UHL**	**Measure**	**Social capital or ressources**	**Name (acronym)**	**Measure**	**Age**	**Name (acronym)**	**Measure**	**Disability**	**Name (acronym)**	
Incomplete vaccination and associated factors among children aged 12–23 months in South Africa: an analysis of the South African demographic and health survey 2016	2021	Ndwandwe et al.	https://doi.org/10.1080/21645515.2020.1791509	Poor, Middle, Rich	Yes	Marital status	Married, Not married	Yes	Maternal age	15–24, 25–34, 35+	No		
Determining the effective coverage of maternal and child health services in Kenya, using demographic and health survey data sets: tracking progress towards universal health coverage	2017	Nguhiu et al.	https://doi.org/10.1111/tmi.12841	Poorest, Poor, Middle, Richer, Richest	No			No			No		

1. BIBLIOGRAPHIC SOURCE												
**Title**	**Year**	**First author**	**UHL**	**Measure**	**Social capital or ressources**	**Name (acronym)**	**Measure**	**Age**	**Name (acronym)**	**Measure**	**Disability**	**Name (acronym)**
Coverage and usage of insecticide treated nets (ITNs) within households: associated factors and effect on the prevalance of malaria parasitemia in the Mount Cameroon area	2019	Njumkeng et al.	https://doi.org/10.1186/s12889-019-7555-x		No			Yes	Age Group (Years)	<5, 5–9, 10–15, >15	No	
Socioeconomic inequalities in maternal health care utilization in Ghana	2019	Novignon et al.	https://doi.org/10.1186/s12939-019-1043-x	Poor, Poorer, Middle, Richer, Richest	No			Yes	Age group of women	15-19, 20-24, 25-29, 30-34, 35-39, 40-44, 45-49	No	

1. BIBLIOGRAPHIC SOURCE												
**Title**	**Year**	**First author**	**UHL**	**Measure**	**Social capital or ressources**	**Name (acronym)**	**Measure**	**Age**	**Name (acronym)**	**Measure**	**Disability**	**Name (acronym)**
Why rural women do not use primary health centres for pregnancy care: Evidence from a qualitative study in Nigeria	2019	Ntoimo et al.	https://doi.org/10.1186/s12884-019-2433-1		Yes	Husband support	Not reported	No			No	
Long-lasting insecticidal net (LLIN) ownership, use and cost of implementation after a mass distribution campaign in Kasaï Occidental Province, Democratic Republic of Congo	2017	Ntuku et al.	https://doi.org/10.1186/s12936-016-1671-1	Poorest,Second, Middle, Fourth, Least poor	No			Yes	Age	<5, 5–19, 20–29, ≥30 years	No	
Explaining socioeconomic disparities and gaps in the use of antenatal care services in 36 countries in sub-Saharan Africa	2021	Obse & Ataguba	https://doi.org/10.1093/heapol/czab036		No			No			No	

1. BIBLIOGRAPHIC SOURCE												
**Title**	**Year**	**First author**	**UHL**	**Measure**	**Social capital or ressources**	**Name (acronym)**	**Measure**	**Age**	**Name (acronym)**	**Measure**	**Disability**	**Name (acronym)**
Sociodemographic factors associated with the use of insecticide treated nets among under-fives in Nigeria: Evidence from a national survey	2022	Ojo et al.	https://doi.org/10.1177/00494755221110374	Lowest, Second, Middle, Frourth, Highest	No			Yes	Age in years	<1, 1, 2, 3, 4	No	
Towards making efficient use of household resources for appropriate prevention of malaria: investigating households' ownership, use and expenditures on ITNs and other preventive tools in Southeast Nigeria	2014	Onwujekwe et al.	http://www.biomedcentral.com/1471-2458/14/315	Rich, least poor, poor, very poor, most poor	No			Yes	Children, Adult, Youth, Others		No	

1. BIBLIOGRAPHIC SOURCE												
**Title**	**Year**	**First author**	**UHL**	**Measure**	**Social capital or ressources**	**Name (acronym)**	**Measure**	**Age**	**Name (acronym)**	**Measure**	**Disability**	**Name (acronym)**
Demographic disparities in unimproved drinking water and sanitation in Ghana: A nationally representative cross-sectional study	2022	Oppong et al.	http://dx.doi.org/10.1136/bmjopen-2021-060595		No			Yes	Age of household head.	<25, 25–35, 36–45, >45 years	No	
Inequities in Access to Maternal Health Care in Enugu State: Implications for Universal Health Coverage to Meet Vision 2030 in Nigeria	2019	Ozumba et al.	https://doi.org/10.1177/0272684X18819977	Richer, Poorer	No			Yes	Age group	Old (30–49), Young (15-29)	No	

1. BIBLIOGRAPHIC SOURCE												
Title	Year	First author	UHL	Measure	Social capital or ressources	Name (acronym)	Measure	Age	Name (acronym)	Measure	Disability	Name (acronym)
How do supply- and demand-side interventions influence equity in healthcare utilisation? Evidence from maternal healthcare in Senegal	2019	Parmar & Banerjee	https://doi.org/10.1016/j.socscimed.2019.112582	Q1 (the poorest 25% households), Q2, Q3, Q4 (the richest 25% households)	No			No			No	
Temporal and regional variations in use, equity and quality of antenatal care in Egypt: A repeat cross-sectional analysis using Demographic and Health Surveys	2019	Pugliese-Garcia et al.	https://doi.org/10.1186/s12884-019-2409-1	Poorest, Middle,Wealthier, Wealthiest	No			No			No	

1. BIBLIOGRAPHIC SOURCE												
**Title**	**Year**	**First author**	**UHL**	**Measure**	**Social capital or ressources**	**Name (acronym)**	**Measure**	**Age**	**Name (acronym)**	**Measure**	**Disability**	**Name (acronym)**
Geographical Inequalities in Use of Improved Drinking Water Supply and Sanitation across Sub-Saharan Africa: Mapping and Spatial Analysis of Cross-sectional Survey Data	2014	Pullan et al.	https://doi.org/10.1371/journal.pmed.1001626		No			No			No	
Individual and Network Factors Associated With HIV Care Continuum Outcomes Among Nigerian MSM Accessing Health Care Services	2018	Ramadhani et al.	https://dx.doi.org/10.1097/QAI.000000000000175[illegible]		Yes	1) Social support score; 2) Network size	1) < Median, ≥ Median; 2) 1–10, 11-20, 21-30, 30+	Yes	1) Age; 2) Age difference	1) ≤19, 20 – 29, ≥30; 2) Same age, Ego older, Alter older	No	
Non-adherence to long-lasting insecticide treated bednet use following successful malaria control in Tororo, Uganda	2020	Rek et al.	https://doi.org/10.1371/journal.pone.0243303	Poorest (lowest tertile), Least poor (all other households)	No			Yes	Age	Under 5, 5-17, >=18	No	

1. BIBLIOGRAPHIC SOURCE													
**Title**	**Year**	**First author**	**UHL**	**Measure**	**Social capital or ressources**	**Name (acronym)**	**Measure**	**Age**	**Name (acronym)**	**Measure**	**Disability**	**Name (acronym)**
Design, implementation and evaluation of a national campaign to deliver 18 million free long-lasting insecticidal nets to uncovered sleeping spaces in Tanzania	2013	Renggli et al.	https://doi.org/10.1186/1475-2875-12-85	Poorest (Q1), Q2, Q2, Q3, Q4, Least poor (Q5)	No			No			No	
A long way to go - Estimates of combined water, sanitation and hygiene coverage for 25 sub-Saharan African countries	2017	Roche et al.	https://doi.org/10.1371/journal.pone.0171783	Poorest (Quintile 1), Quintile 2, Quintile 3, Quintile 4, Richest (Quintile 5)	No			No			No	

1. BIBLIOGRAPHIC SOURCE												
**Title**	**Year**	**First author**	**UHL**	**Measure**	**Social capital or ressources**	**Name (acronym)**	**Measure**	**Age**	**Name (acronym)**	**Measure**	**Disability**	**Name (acronym)**
Determinants of bed net use in southeast Nigeria following mass distribution of LLINs: Implications for social behavior change interventions	2015	Russell et al.	https://doi.org/10.1371/journal.pone.0139447	Not reported	Yes	Social support score	Low, Moderate, High	No			No	
Factors associated with the non-use of insecticide-treated nets in Rwandan children	2016	Ruyange et al.	https://doi.org/10.1186/s12936-016-1403-6	1-2) Poorest/poor, Middle/higher	Yes	Mother marital status	Never in union, Widowed/divorced/no longer living together, Married/living with partner	Yes	Children age (M)	≤12 279, 13–23, >23	No	

1. BIBLIOGRAPHIC SOURCE												
**Title**	**Year**	**First author**	**UHL**	**Measure**	**Social capital or ressources**	**Name (acronym)**	**Measure**	**Age**	**Name (acronym)**	**Measure**	**Disability**	**Name (acronym)**
Wealth Status, Health Insurance, and Maternal Health Care Utilization in Africa: Evidence from Gabon	2020	Sanogo & Yaya	https://dx.doi.org/10.1155/2020/4036830	Poorest, poorer, middle, richer, richest	No			Yes	1) Age groups; 2) Age at first birth	1) 15–24, 25–29, 30–34, 35–39, 40–44, and 45–49; 2) <18, 18–25, ≥26	No	

1. BIBLIOGRAPHIC SOURCE												
**Title**	**Year**	**First author**	**UHL**	**Measure**	**Social capital or ressources**	**Name (acronym)**	**Measure**	**Age**	**Name (acronym)**	**Measure**	**Disability**	**Name (acronym)**
Factors associated with use of insecticide-treated net for malaria prevention in Manica District, Mozambique: a community-based cross-sectional survey	2021	Scott et al.	https://doi.org/10.1186/s12936-021-03738-7	Poorest, Middle, Wealthiest	No			Yes	Age category	10–14, 15–19, 20–24, 25–39, ≥40	No	
Healthcare utilisation, cancer screening and potential barriers to accessing cancer care in rural South West Nigeria: a cross-sectional study	2021	Sharma et al.	https://doi.org/10.1136/bmjopen-2020-040352	1-2) <10 000, 10 000–49 999, >50 000	Yes	Marital status	Single, Married, Others (divorced/cohabiting)	No			No	

1. BIBLIOGRAPHIC SOURCE													
**Title**	**Year**	**First author**	**UHL**	**Measure**	**Social capital or ressources**	**Name (acronym)**	**Measure**	**Age**	**Name (acronym)**	**Measure**	**Disability**	**Name (acronym)**	
Socio-economic inequalities in ANC attendance among mothers who gave birth in the past 12 months in Debre Brehan town and surrounding rural areas, North East Ethiopia: A community-based survey	2019	Shibre & Mekonnen	https://doi.org/10.1186/s12978-019-0768-8	Poorest, Poorer, Middle, Rich, Rechest	Yes	Marital status	Single, married, divorced/widowed	Yes	Mother's age at last birth	17–19, 20–30, 31–44	No		

1. BIBLIOGRAPHIC SOURCE												
**Title**	**Year**	**First author**	**UHL**	**Measure**	**Social capital or ressources**	**Name (acronym)**	**Measure**	**Age**	**Name (acronym)**	**Measure**	**Disability**	**Name (acronym)**
Individual and environmental characteristics associated with immunization of children in rural areas of Burkina Faso: A multi-level analysis	2007	Sia et al.	https://pubmed.ncbi.nlm.nih.gov/18299262/	Pauvre [Poor], Nantis [wealthy]	No			No			No	
Inequalities in access and utilization of maternal, newborn and child health services in sub-saharan africa: A special focus on urban settings	2021	Sidze et al.	https://dx.doi.org/10.1007/s10995-021-03250-z		Yes	Marital status		Yes	Maternal age		No	

1. BIBLIOGRAPHIC SOURCE											
**Title**	**Year**	**First author**	**UHL**	**Measure**	**Social capital or ressources**	**Name (acronym)**	**Measure**	**Age**	**Name (acronym)**	**Measure**	**Disability**	**Name (acronym)**
Insecticide-treated bed net access and use among preschool children in Nouna District, Burkina Faso	2020	Sié et al.	https://dx.doi.org/10.1093/inthealth/ihaa003	Score	No			Yes	Age (years)	0, 1, 2, 3, 4	No	
Evidence of improving antiretroviral therapy treatment delays: an analysis of eight years of programmatic outcomes in Blantyre, Malawi	2013	Sloan et al.	http://www.biomedcentral.com/1471-2458/13/490		No			Yes	Age group at initiation	0-5, 5-15, 15-25, 25-35, 35-45, >=45	No	
Low use of long-lasting insecticidal nets for malaria prevention in south-central Ethiopia: A community-based cohort study	2019	Solomon et al.	https://doi.org/10.1371/journal.pone.0210578	Poor, Medium, Rich	No			Yes	Age (years) group	<5, 5-14, 15-24, >=25	No	

1. BIBLIOGRAPHIC SOURCE												
**Title**	**Year**	**First author**	**UHL**	**Measure**	**Social capital or ressources**	**Name (acronym)**	**Measure**	**Age**	**Name (acronym)**	**Measure**	**Disability**	**Name (acronym)**
Empowerment and use of modern contraceptive methods among married women in Burkina Faso: a multilevel analysis	2021	Some et al.	https://dx.doi.org/10.1186/s12889-021-11541-x	Poor, Middle, Rich	No			Yes	Women's age	15–24, 25–39, 40–49	No	
Evaluation of the 2011 long-lasting, insecticide-treated net distribution for universal coverage in Togo	2013	Stevens et al.	http://www.malariajournal.com/content/12/1/162		No			Yes	Age group	<5, 5-14, 15-49, 50+	No	
On the way to universal coverage of maternal services in Iringa rural District in Tanzania. Who is yet to be reached?	2016	Straneo et al.	https://doi.org/10.4314/ahs.v16i2.10	Very low, low, medium, high, very high	No			Yes	Age at index delivery	≤20, 21-34, ≥35	No	

1. BIBLIOGRAPHIC SOURCE												
**Title**	**Year**	**First author**	**UHL**	**Measure**	**Social capital or ressources**	**Name (acronym)**	**Measure**	**Age**	**Name (acronym)**	**Measure**	**Disability**	**Name (acronym)**
Evaluation of long-lasting insecticidal net distribution through schools in Southern Tanzania	2022	Stuck et al.	https://doi.org/10.1093/heapol/czab140		No			No		Yes, no	No	
Not all inequalities are equal: differences in coverage across the continuum of reproductive health services	2019	Sully et al.	https://dx.doi.org/10.1136/bmjgh-2019-001695	Five quintiles	No			Yes	Age	15–19, 20–24, 25–34, 35–49	No	

1. BIBLIOGRAPHIC SOURCE												
**Title**	**Year**	**First author**	**UHL**	**Measure**	**Social capital or ressources**	**Name (acronym)**	**Measure**	**Age**	**Name (acronym)**	**Measure**	**Disability**	**Name (acronym)**
Determinants of long-lasting insecticidal net ownership and utilization in malaria transmission regions: Evidence from Zimbabwe Demographic and Health Surveys	2019	Tapera	https://doi.org/10.1186/s12936-019-2912-x	Poorest, Poorer, Middle, Richer, Rechest	No			Yes	Household head < 30 years	Yes, No	No	
Sociodemographic inequities in cervical cancer screening, treatment and care amongst women aged at least 25 years: evidence from surveys in Harare, Zimbabwe	2019	Tapera et al.	https://doi.org/10.1186/s12889-019-6749-6	1) No income, <US$200, 200–400, ≥ 430; 2) no income, <US$600, 600–1000, ≥ 1200; 3) poor, middle, rich	Yes	Marital status	Married/co-habiting, Never married, Widowed, Divorced/separated	Yes	Age (years)	25-44, 45 or more	No	

1. BIBLIOGRAPHIC SOURCE												
**Title**	**Year**	**First author**	**UHL**	**Measure**	**Social capital or ressources**	**Name (acronym)**	**Measure**	**Age**	**Name (acronym)**	**Measure**	**Disability**	**Name (acronym)**
Factors associated to bed net use in Cameroon: a retrospective study in Mfou health district in the Centre Region	2012	Tchinda et al.	http://www.panafrican-med-journal.com/content/article/12/112/full/		No			Yes	1) Age groups (years); 2) Child<5 years	1) < 5/5–24/25–49/>=50; 2) Yes/No	No	
Cervical cancer screening uptake and correlates among HIV-infected women: a cross-sectional survey in Cote d'Ivoire, West Africa	2019	Tchounga et al.	https://dx.doi.org/10.1136/bmjopen-2019-029882		No			Yes	Age category	<45/≥45	No	

1. BIBLIOGRAPHIC SOURCE												
**Title**	**Year**	**First author**	**UHL**	**Measure**	**Social capital or ressources**	**Name (acronym)**	**Measure**	**Age**	**Name (acronym)**	**Measure**	**Disability**	**Name (acronym)**
Duration and determinants of delayed tuberculosis diagnosis and treatment in high-burden countries: a mixed-methods systematic review and meta-analysis	2021	Teo et al.	https://doi.org/10.1186/s12931-021-01841-6	Low income, high income	Yes	Marital status	Married, widowed, divorced, separated, not married	Yes	Age	Older age, younger age	No	
Skilled delivery inequality in Ethiopia: To what extent are the poorest and uneducated mothers benefiting?	2017	Tesfaye et al.	https://doi.org/10.1186/s12939-017-0579-x	poorest, poorer, medium, richer and richest	No			No			No	

1. BIBLIOGRAPHIC SOURCE												
**Title**	**Year**	**First author**	**UHL**	**Measure**	**Social capital or ressources**	**Name (acronym)**	**Measure**	**Age**	**Name (acronym)**	**Measure**	**Disability**	**Name (acronym)**
Investigating the association between pregnancy intention and insecticide-treated bed net (ITN) use: A cross-sectional study of pregnant women in Rwanda	2015	Thogarapalli et al.	https://doi.org/10.1007/s10389-015-0676-5	Poorest, poorer, middle, richer, richest	Yes	Marital status	Never married, married or living with partner, divorced, separated or widowed	No			No	
Success of Senegal's first nationwide distribution of long-lasting insecticide-treated nets to children under five - Contribution toward universal coverage	2011	Thwing et al.	http://www.malariajournal.com/content/10/1/86		No			Yes	Child age	< 12 months, 12-59 months	No	
Declines in Malaria Burden and all-cause child mortality following increases in control interventions in Senegal, 2005-2010	2017	Thwing et al.	https://doi.org/10.4269/ajtmh.16-0953	Poorest, fourth, middle, second, richest	No			Yes	Age	6-11 months, 12-23 months, 24-59 months, neonatal (0-28 days), postnatal (1-12 months), infant (0-12months)	No	

1. BIBLIOGRAPHIC SOURCE											
**Title**	**Year**	**First author**	**UHL**	**Measure**	**Social capital or ressources**	**Name (acronym)**	**Measure**	**Age**	**Name (acronym)**	**Measure**	**Disability**	**Name (acronym)**
Individual-level and community-level determinants of cervical cancer screening among Kenyan women: a multilevel analysis of a Nationwide survey	2017	Tiruneh et al.	https://doi.org/10.1186/s12905-017-0469-9	Poorest, poor, middleclass, rich, and richest	No			Yes	Age	15–24, 25–34, and 35–49 years	No	
Evaluation of the coverage and effective use rate of long-lasting insecticidal nets after nation-wide scale up of their distribution in Benin	2013	Tokponnon et al.	https://doi.org/10.1186/1756-3305-6-265		No			Yes	Age	15-25, 26-35, 36-45, 46 and more years	No	
Factors associated with the upsurge in the use of delivery care services in Sierra Leone	2020	Tsawe & Susuman	https://doi.org/10.1016/j.puhe.2019.11.002	Poor, average, rich	Yes	Marital status	Never married, in union, formerly married	Yes	Maternal age, years	<20, 20-29, 30-39, 40-49	No	

1. BIBLIOGRAPHIC SOURCE												
**Title**	**Year**	**First author**	**UHL**	**Measure**	**Social capital or ressources**	**Name (acronym)**	**Measure**	**Age**	**Name (acronym)**	**Measure**	**Disability**	**Name (acronym)**
Which family members use the best nets? An analysis of the condition of mosquito nets and their distribution within households in Tanzania	2010	Tsuang et al.	http://www.malariajournal.com/content/9/1/211		No			Yes	Person type	infants (<1 year), young children (1-4 years), older children (5-14 years), adult males (≥15 years), adult non-pregnant females (15-49 years), adult pregnant females (15-49 years), and older females (≥50 years).	No	
Utilization of insecticide treated nets among pregnant women in enugu, South Eastern Nigeria	2013	Ugwu et al.	https://pubmed.ncbi.nlm.nih.gov/23771448/	I, II, III, IV, V	No			No			No	

1. BIBLIOGRAPHIC SOURCE												
**Title**	**Year**	**First author**	**UHL**	**Measure**	**Social capital or ressources**	**Name (acronym)**	**Measure**	**Age**	**Name (acronym)**	**Measure**	**Disability**	**Name (acronym)**
Finding the gap: Revealing local disparities in coverage of maternal, newborn and child health services in South Sudan using lot quality assurance sampling	2015	Valadez et al.	https://doi.org/10.1111/tmi.12613		No			No			No	
Group Medical Visit and Microfinance Intervention for Patients With Diabetes or Hypertension in Kenya	2021	Vedanthan et al.	https://doi.org/10.1016/j.jacc.2021.03.002	IWI 4.00-14.99, IWI 15-24.99, IWI 25-39.99, IWI>=40	No			Yes	Age	35-54, 55-74, >=75	No	

1. BIBLIOGRAPHIC SOURCE												
**Title**	**Year**	**First author**	**UHL**	**Measure**	**Social capital or ressources**	**Name (acronym)**	**Measure**	**Age**	**Name (acronym)**	**Measure**	**Disability**	**Name (acronym)**
Equity in Maternal Health in South Africa: Analysis of Health Service Access and Health Status in a National Household Survey	2013	Wabiri et al.	https://doi.org/10.1371/journal.pone.0073864	QI poorest, QII, QIV wealthiest	No			Yes	Age	15-19, 20-29, 30-39, 40-54	No	
Persisting Regional Disparities in Modern Contraceptive Use and Unmet Need for Contraception among Nigerian Women	2019	Wang & Cao	https://doi.org/10.1155/2019/9103928	poorest/poorer/middle/richer/richest	Yes	Marital status	in union/widowed/other	No			No	

1. BIBLIOGRAPHIC SOURCE												
**Title**	**Year**	**First author**	**UHL**	**Measure**	**Social capital or ressources**	**Name (acronym)**	**Measure**	**Age**	**Name (acronym)**	**Measure**	**Disability**	**Name (acronym)**
Effective coverage of facility delivery in Bangladesh, Haiti, Malawi, Nepal, Senegal, and Tanzania	2019	Wang et al.	https://doi.org/10.1371/journal.pone.0217853		No			No			No	
Use of long-lasting insecticide-treated bed nets in a population with universal coverage following a mass distribution campaign in Uganda	2016	Wanzira et al.	https://doi.org/10.1186/s12936-016-1360-0		No			Yes	Age categories	0-5, 6-14, 15-45, >45	No	

1. BIBLIOGRAPHIC SOURCE												
**Title**	**Year**	**First author**	**UHL**	**Measure**	**Social capital or ressources**	**Name (acronym)**	**Measure**	**Age**	**Name (acronym)**	**Measure**	**Disability**	**Name (acronym)**
Long-lasting insecticide-treated bed net ownership and use among children under five years of age following a targeted distribution in central Uganda	2014	Wanzira et al.	https://doi.org/10.1186/1475-2875-13-185	1= poorest; 5= poorest	No			No			No	
Evaluation of a national universal coverage campaign of long-lasting insecticidal nets in a rural district in north-west Tanzania	2012	West et al.	https://doi.org/10.1186/1475-2875-11-273	Poorest/Middle/Richest	No			No			No	
The impact of renewing long-lasting insecticide-treated nets in the event of malaria resurgence: Lessons from 10 years of net use in dielmo, Senegal	2021	Wotodjo et al.	https://doi.org/10.4269/AJTMH.20-0127		No			Yes	Age-group (years)	< 5, 5–9, 10–14, 15–29, 30–44, >=45	No	

1. BIBLIOGRAPHIC SOURCE													
**Title**	**Year**	**First author**	**UHL**	**Measure**	**Social capital or ressources**	**Name (acronym)**	**Measure**	**Age**	**Name (acronym)**	**Measure**	**Disability**	**Name (acronym)**
Wealth and Education Inequities in Maternal and Child Health Services Utilization in Rural Ethiopia	2022	Wuneh et al.	https://doi.org/10.3390/ijerph19095421	Poor, middle, better-off	No			No			No	
Inequalities in maternal health care utilization in Benin: A population based cross-sectional study	2018	Yaya et al.	https://doi.org/10.1186/s12884-018-1846-6	Poorest, poorer, middle, richer, richest	No			Yes	Age of individual woman	15–19, 20–24, 25–29, 30–34, 35–39, 40–44, 45–49	No	

1. BIBLIOGRAPHIC SOURCE												
Title	Year	First author	UHL	Measure	Social capital or ressources	Name (acronym)	Measure	Age	Name (acronym)	Measure	Disability	Name (acronym)
Long-lasting insecticide-treated bed net ownership, utilization and associated factors among school-age children in Dara Mallo and Uba Debretsehay districts, Southern Ethiopia	2020	Zerdo et al.	https://dx.doi.org/10.1186/s12936-020-03437-9	First, Second, Third, Fourth	No			Yes	1) Age of the school-age children (SAC); 2) Age of child's mother	1) Age of the SAC (7-9/10-14); 2) Age of household head (≤34/35–49/≥50); 3) Age of child's mother (≤34/35–49/≥50)	No	
Trends and projections of universal health coverage indicators in Ghana, 1995-2030: A national and subnational study	2019	Zhang et al.	https://doi.org/10.1371/journal.pone.0209126	Poorest, Poorer, Middle class, Richer, Richest	No			No			No	

1. BIBLIOGRAPHIC SOURCE												
**Title**	**Year**	**First author**	**UHL**	**Measure**	**Social capital or ressources**	**Name (acronym)**	**Measure**	**Age**	**Name (acronym)**	**Measure**	**Disability**	**Name (acronym)**
Developing Malawi's Universal Health Coverage Index	2022	Mchenga et al.	https://doi.org/10.3389/frhs.2021.786186	Pro-rich (yes, no)	No			No			No	
Socioeconomic Factors Associated with Compliance with Mass Drug Administration for Lymphatic Filariasis Elimination in Kenya: Descriptive Study Results	2012	Njomo et al.	https://www.proquest.com/docview/1115911778?pq-origsite=gscholar&fromopenview=true		No			No			No	

1. BIBLIOGRAPHIC SOURCE												
**Title**	**Year**	**First author**	**UHL**	**Measure**	**Social capital or ressources**	**Name (acronym)**	**Measure**	**Age**	**Name (acronym)**	**Measure**	**Disability**	**Name (acronym)**
Equality analysis of main health indicators among children under 5 years in Uganda	2019	Elduma	http://dx.doi.org/10.4314/ejhs.v29i2.8	Quintile 1 (poorest)/Quintile 2/Quintile 3/Quintile 4e/Quintile 5 (richest)	No			No			No	

1. BIBLIOGRAPHIC SOURCE												
**Title**	**Year**	**First author**	**UHL**	**Measure**	**Sexual orientation**	**Name (acronym)**	**Measure**	**Other**	**Name (acronym)**	**Measure**	**Technique used for measurement of**	**Family planning (FP)**
Reproductive Plans And Utilization of Contraceptives Among Women Living With HIV	2019	Adeleye et al.	https://dx.doi.org/10.21106/ijma.277		No			No			Relative	Yes
Provision of immediate postpartum contraception to women living with HIV in the Eastern Cape, South Africa; a cross-sectional analysis	2020	Adeniyi et al.	https://doi.org/10.1186/s12978-020-01049-9		No			No			Relative	Yes
Moving Up the Sanitation Ladder: A Study of the Coverage and Utilization of Improved Sanitation Facilities and Associated Factors Among Households in Southern Ethiopia	2022	Afework et al.	https://doi.org/10.1177/11786302221080825		No			No			Relative	No

1. BIBLIOGRAPHIC SOURCE												
Title	Year	First author	UHL	Measure	Sexual orientation	Name (acronym)	Measure	Other	Name (acronym)	Measure	Technique used for measurement of	Family planning (FP)
Maternal health care services utilisation in the context of 'Abiye' (safe motherhood) programme in Ondo State, Nigeria	2020	Ajayi et al.	https://doi.org/10.1186/s12889-020-08512-z		No			No			Relative	No
Slums, women and sanitary living in South-South Nigeria	2021	Akpabio et al.	https://doi.org/10.1007/s10901-020-09802-z		No			No			Unclear	No
Leaving no child behind: Decomposing socioeconomic inequalities in child health for india and south africa	2021	Alaba et al.	https://doi.org/10.3390/ijerph1813711 4		No			Yes			Relative & graph	No

1. BIBLIOGRAPHIC SOURCE												
Title	Year	First author	UHL	Measure	Sexual orientation	Name (acronym)	Measure	Other	Name (acronym)	Measure	Technique used for measurement of	Family planning (FP)
Access to skilled attendant at birth and the coverage of the third dose of diphtheria-tetanus-pertussis vaccine across 14 West African countries - An equity analysis	2020	Alhassan et al.	https://doi.org/10.1186/s12939-020-01204-5		No			No			Absolute & graph	No
Trends and correlates of maternal, newborn and child health services utilization in primary healthcare facilities: An explorative ecological study using DHIMSII data from one district in the Volta region of [illegible]	2020	Alhassan et al.	https://doi.org/10.1186/s12884-020-03195-1		No			No			Relative	No

1. BIBLIOGRAPHIC SOURCE													
**Title**	**Year**	**First author**	**UHL**	**Measure**	**Sexual orientation**	**Name (acronym)**	**Measure**	**Other**	**Name (acronym)**	**Measure**	**Technique used for measurement of**		**Family planning (FP)**
Determinants of equity in utilization of maternal health services in Butajira, Southern Ethiopia	2012	Aliy & Mariam	https://www.ajol.info/index.php/ejhd/article/view/116114		No			No		Walking distance to a health facility	Relative		No
Determinants of insecticide treated nets use among youth corp members in Edo State, Nigeria	2011	Amoran O. E. et al	http://www.biomedcentral.com/1471-2458/11/728		No			No			Relative		No

1. BIBLIOGRAPHIC SOURCE													
**Title**	**Year**	**First author**	**UHL**	**Measure**	**Sexual orientation**	**Name (acronym)**	**Measure**	**Other**	**Name (acronym)**	**Measure**	**Technique used for measurement of**		**Family planning (FP)**
Towards achievement of Sustainable Development Goal 3: multilevel analyses of demographic and health survey data on health insurance coverage and maternal healthcare utilisation in sub-Saharan Africa	2022	Amu et al.	https://doi.org/10.1093/inthealth/ihac017		No			No			Relative		No

1. BIBLIOGRAPHIC SOURCE												
**Title**	**Year**	**First author**	**UHL**	**Measure**	**Sexual orientation**	**Name (acronym)**	**Measure**	**Other**	**Name (acronym)**	**Measure**	**Technique used for measurement of**	**Family planning (FP)**
Equity and access to maternal and child health services in Ghana a cross-sectional study	2021	Anarwat et al.	https://doi.org/10.1186/s12913-021-06872-9		No			No			Relative	Yes
A subnational profiling analysis reveals regional differences as the main predictor of ITN ownership and use in Nigeria	2019	Andrada et al.	https://doi.org/10.1186/s12936-019-2816-9		No			No			Relative	No
Inequality trends in maternal health services for young Ghanaian women with childbirth history between 2003 and 2014	2017	Asamoah & Agardh	https://doi.org/10.1136/bmjopen-2016-011663		No			No			Relative	No

1. BIBLIOGRAPHIC SOURCE												
**Title**	**Year**	**First author**	**UHL**	**Measure**	**Sexual orientation**	**Name (acronym)**	**Measure**	**Other**	**Name (acronym)**	**Measure**	**Technique used for measurement of**	**Family planning (FP)**
Leaving no one behind: Lessons from implementation of policies for universal HIV treatment to universal health coverage	2020	Assefa et al.	https://doi.org/10.1186/s12992-020-00549-4		No			No			Absolute, relative & graph	No
Inequalities in child immunization coverage in Ghana: evidence from a decomposition analysis	2018	Asuman et al.	https://doi.org/10.1186/s13561-018-0193-7		No			No			Relative	No
A reassessment of global antenatal care coverage for improving maternal health using sub-Saharan Africa as a case study	2018	Ataguba	https://dx.doi.org/10.1371/journal.pone.0204822		No			No			Relative	No

1. BIBLIOGRAPHIC SOURCE													
**Title**	**Year**	**First author**	**UHL**	**Measure**	**Sexual orientation**	**Name (acronym)**	**Measure**	**Other**	**Name (acronym)**	**Measure**	**Technique used for measurement of**		**Family planning (FP)**
Socio-economic inequality in maternal health care utilization in Sub-Saharan Africa: Evidence from Togo	2021	Atake	http://dx.doi.org/10.1002/hpm.3083		No			No			Relative & graph		No
Poor prenatal service utilization and pregnancy outcome in a tertiary health facility in southwest Nigeria	2020	Awoleke & Olofinbiyi	https://doi.org/10.11604/pamj.2020.35.28.20426		No			No			Relative		No
Correlates of intra-household ITN use in Liberia: A multilevel analysis of household survey data	2016	Babalola et al.	http://dx.doi.org/10.1371/journal.pone.0158331		No			No			Relative		No

1. BIBLIOGRAPHIC SOURCE													
**Title**	**Year**	**First author**	**UHL**	**Measure**	**Sexual orientation**	**Name (acronym)**	**Measure**	**Other**	**Name (acronym)**	**Measure**	**Technique used for measurement of**		**Family planning (FP)**
Prevalence and determinants of maternal healthcare utilisation among young women in sub-Saharan Africa: cross-sectional analyses of demographic and health survey data	2022	Bain et al.	https://doi.org/10.1186/s12889-022-13037-8		No			No			Relative		No

1. BIBLIOGRAPHIC SOURCE													
**Title**	**Year**	**First author**	**UHL**	**Measure**	**Sexual orientation**	**Name (acronym)**	**Measure**	**Other**	**Name (acronym)**	**Measure**	**Technique used for measurement of**		**Family planning (FP)**
Factors influencing timing and frequency of antenatal care in Uganda	2011	Bbaale	https://www.ncbi.nlm.nih.gov/pmc/articles/PMC3562883/pdf/AMJ-04-431.pdf		No			No			Relative		No
Maternal education and childbirth care in Uganda	2011	Bbaale & Guloba	https://www.ncbi.nlm.nih.gov/pmc/articles/PMC3562941/pdf/AMJ-04-389.pdf		No			No			Relative		No

1. BIBLIOGRAPHIC SOURCE												
**Title**	**Year**	**First author**	**UHL**	**Measure**	**Sexual orientation**	**Name (acronym)**	**Measure**	**Other**	**Name (acronym)**	**Measure**	**Technique used for measurement of**	**Family planning (FP)**
A quantitative analysis of food insecurity and other barriers associated with ART nonadherence among women in rural communities of Eswatini	2021	Becker et al.	https://doi.org/10.1371/journal.pone.0256277		No			No			Relative	No
Two decades of antenatal and delivery care in Uganda: a cross-sectional study using Demographic and Health Surveys	2018	Benova et al.	https://doi.org/10.1186/s12913-018-3546-3		No			No			Absolute	No
Utilization of sexual and reproductive health services in ethiopia - Does it affect sexual activity among high school students?	2015	Bilal et al.	https://doi.org/10.1016/j.srhc.2014.09.009		No			No			Relative	Yes

1. BIBLIOGRAPHIC SOURCE												
**Title**	**Year**	**First author**	**UHL**	**Measure**	**Sexual orientation**	**Name (acronym)**	**Measure**	**Other**	**Name (acronym)**	**Measure**	**Technique used for measurement of**	**Family planning (FP)**
Addressing the huge poor–rich gap of inequalities in accessing safe childbirth care: A first step to achieving universal maternal health coverage in Tanzania	2021	Bintabara	https://doi.org/10.1371/journal.pone.0246995		No			No			Relative & graph	No
Twelve-year persistence of inequalities in antenatal care utilisation among women in Tanzania: A decomposition analysis of population-based cross-sectional surveys	2021	Bintabara & Basinda	https://doi.org/10.1136/bmjopen-2020-040450		No			No			Relative & graph	No
Socio-demographic and economic inequalities in modern contraception in 11 low- And middle-income countries: An analysis of the PMA2020 surveys	2020	Blumenberg et al.	https://doi.org/10.1186/s12978-020-00931-w		No			No			Relative	Yes

1. BIBLIOGRAPHIC SOURCE													
**Title**	**Year**	**First author**	**UHL**	**Measure**	**Sexual orientation**	**Name (acronym)**	**Measure**	**Other**	**Name (acronym)**	**Measure**	**Technique used for measurement of**		**Family planning (FP)**
A decomposition analysis of change in skilled birth attendants, 2003 to 2008, Ghana demographic and health surveys	2014	Bosomprah et al.	https://doi.org/10.1186/s12884-014-0415-x		No			No			Relative		No
Slow and Steady can Still Win the Race': Childhood Vaccination Experience of Migrant Ebira Women Within the Health System in Ekiti State, Nigeria	2021	Olakanmi-Falade & Awoleke	https://www.ojhas.org/issue79/2021-3-3.html		No			No			Relative		No
Antiretroviral therapy in Walvis Bay, Namibia	2016	Callaghan	https://hdl.handle.net/1807/70825		No			No			Absolute		No

1. BIBLIOGRAPHIC SOURCE													
**Title**	**Year**	**First author**	**UHL**	**Measure**	**Sexual orientation**	**Name (acronym)**	**Measure**	**Other**	**Name (acronym)**	**Measure**	**Technique used for measurement of**		**Family planning (FP)**
Family planning, antenatal and delivery care: Cross-sectional survey evidence on levels of coverage and inequalities by public and private sector in 57 low- and middle-income countries	2016	Campbell et al.	https://doi.org/10.1111/tmi.12681		No			No			Absolute		Yes

1. BIBLIOGRAPHIC SOURCE												
**Title**	**Year**	**First author**	**UHL**	**Measure**	**Sexual orientation**	**Name (acronym)**	**Measure**	**Other**	**Name (acronym)**	**Measure**	**Technique used for measurement of**	**Family planning (FP)**
Use of family planning and child health services in the private sector: An equity analysis of 12 DHS surveys	2018	Chakraborty & Sprockett	https://doi.org/10.1186/s12939-018-0763-7		No			No			Absolute	Yes
Meeting demand for family planning within a generation: prospects and implications at country level	2015	Choi et al.	https://dx.doi.org/10.3402/gha.v8.29734		No			No			Absolute	Yes
A Longitudinal Analysis of Mosquito Net Ownership and Use in an Indigenous Batwa Population after a Targeted Distribution	2016	Clark et al.	https://doi.org/10.1371/journal.pone.0154808		No			No			Relative	No

1. BIBLIOGRAPHIC SOURCE												
**Title**	**Year**	**First author**	**UHL**	**Measure**	**Sexual orientation**	**Name (acronym)**	**Measure**	**Other**	**Name (acronym)**	**Measure**	**Technique used for measurement of**	**Family planning (FP)**
Malaria prevalence and long-lasting insecticidal net use in rural western Uganda: results of a cross-sectional survey conducted in an area of highly variable malaria transmission intensity	2021	Cote et al.	https://doi.org/10.1186/s12936-021-03835-7		No			No			Relative	No
Women's Sexual Empowerment and Contraceptive Use in Ghana	2012	Crissman et al.	https://doi.org/10.1111/j.1728-4465.2012.00318.x		No			No			Relative	Yes
Socio-economic and demographic factors associated with reproductive and child health preventive care in Mozambique: a cross-sectional study	2020	Daca et al.	https://doi.org/10.1186/s12939-020-01303-3		No			No			Relative	Yes

1. BIBLIOGRAPHIC SOURCE												
**Title**	**Year**	**First author**	**UHL**	**Measure**	**Sexual orientation**	**Name (acronym)**	**Measure**	**Other**	**Name (acronym)**	**Measure**	**Technique used for measurement of**	**Family planning (FP)**
Assessing the contextual effect of community in the utilization of postnatal care services in Ghana	2021	Dankwah et al.	https://doi.org/10.1186/s12913-020-06028-1		No			No			Relative	No
Is South Africa closing the health gaps between districts? Monitoring progress towards universal health service coverage with routine facility data	2021	Day et al.	https://doi.org/10.1186/s12913-021-06171-3		No			No			Absolute	Yes
Neglected tropical diseases as a 'litmus test' for universal health coverage? understanding who is left behind and why in mass drug administration: Lessons from four country contexts	2019	Dean et al.	https://doi.org/10.1371/journal.pntd.0007847	Not reported	No			No			Unclear	No

1. BIBLIOGRAPHIC SOURCE												
**Title**	**Year**	**First author**	**UHL**	**Measure**	**Sexual orientation**	**Name (acronym)**	**Measure**	**Other**	**Name (acronym)**	**Measure**	**Technique used for measurement of**	**Family planning (FP)**
Preventive Health Service Coverage Among Infants and Children at Six Maternal-Child Health Clinics in Western Kenya: A Cross-Sectional Assessment	2022	Deathe et al.	https://doi.org/10.1007/s10995-021-03271-8		No			No			Absolute	No
Distance, difference in altitude and socioeconomic determinants of utilisation of maternal and child health services in Ethiopia: A geographic and multilevel modelling analysis	2021	Defar et al.	https://doi.org/10.1136/bmjopen-2020-042095		No			No			Relative	No
Geographic differences in maternal and child health care utilization in four Ethiopian regions; A cross-sectional study	2019	Defar et al.	https://doi.org/10.1186/s12939-019-1079-y		No			No			Relative	No

1. BIBLIOGRAPHIC SOURCE												
**Title**	**Year**	**First author**	**UHL**	**Measure**	**Sexual orientation**	**Name (acronym)**	**Measure**	**Other**	**Name (acronym)**	**Measure**	**Technique used for measurement of**	**Family planning (FP)**
Ownership and use of insecticide-treated nets in Oromia and Amhara Regional States of Ethiopia twoyears after a nationwide campaign	2011	Deressa et al.	https://doi.org/10.1111/j.1365-3156.2011.02875.x		No			No			Relative	No
Effectiveness of post-campaign, door-to-door, hang-up, and communication interventions to increase long-lasting, insecticidal bed net utilization in Togo (2011-2012): A cluster randomized, control trial	2014	Desrochers et al.	https://doi.org/10.1186/1475-2875-13-260		No			No			Absolute	No
Immediate postnatal care following childbirth in Ugandan health facilities: An analysis of Demographic and Health Surveys between 2001 and 2016	2021	Dey et al.	https://doi.org/10.1136/bmjgh-2020-004230		No			No			Relative	No

1. BIBLIOGRAPHIC SOURCE												
**Title**	**Year**	**First author**	**UHL**	**Measure**	**Sexual orientation**	**Name (acronym)**	**Measure**	**Other**	**Name (acronym)**	**Measure**	**Technique used for measurement of**	**Family planning (FP)**
Inequalities in non-communicable diseases and effective responses	2013	Di Cesare et al.	https://doi.org/10.1016/s0140-6736(12)61851-0		No			No			Absolute	No
Insecticide-treated nets ownership and utilization among under-five children following the 2010 mass distribution in Burkina Faso	2014	Diabate et al.	https://dx.doi.org/10.1186/1475-2875-13-353		No			No			Relative	No

1. BIBLIOGRAPHIC SOURCE													
**Title**	**Year**	**First author**	**UHL**	**Measure**	**Sexual orientation**	**Name (acronym)**	**Measure**	**Other**	**Name (acronym)**	**Measure**	**Technique used for measureme nt of**		**Family planning (FP)**
Impact of mining projects on water and sanitation infrastructures and associated child health outcomes: a multi-country analysis of Demographic and Health Surveys (DHS) in sub-Saharan Africa	2021	Dietler et al.	https://doi.org/10.1186/s12992-021-00723-2		No			No			Relative		No

1. BIBLIOGRAPHIC SOURCE												
**Title**	**Year**	**First author**	**UHL**	**Measure**	**Sexual orientation**	**Name (acronym)**	**Measure**	**Other**	**Name (acronym)**	**Measure**	**Technique used for measurement of**	**Family planning (FP)**
Predictors of insecticidal net use among internally displaced persons aged 6-59 months in Abuja, Nigeria	2018	Ejembi et al.	http://dx.doi.org/10.11604/pamj.2018.29.136.13322		No			No			Relative	No
Changes in Inequality in Use of Maternal Health Care Services: Evidence from Skilled Birth Attendance in Mauritania for the Period 2007-2015	2022	Taleb El Hassen et al.	https://doi.org/10.3390/ijerph19063566		No			No			Absolute, relative & graph	No

1. BIBLIOGRAPHIC SOURCE													
**Title**	**Year**	**First author**	**UHL**	**Measure**	**Sexual orientation**	**Name (acronym)**	**Measure**	**Other**	**Name (acronym)**	**Measure**	**Technique used for measurement of**		**Family planning (FP)**
Trend in the use of modern contraception in sub-Saharan Africa: Does women's education matter?	2014	Emina et al.	http://dx.doi.org/10.1016/j.contraception.2014.02.001		No			No			Relative		Yes

1. BIBLIOGRAPHIC SOURCE													
**Title**	**Year**	**First author**	**UHL**	**Measure**	**Sexual orientation**	**Name (acronym)**	**Measure**	**Other**	**Name (acronym)**	**Measure**	**Technique used for measurement of**		**Family planning (FP)**
Utilization, Predictors and Gaps in the Continuum of Care for Maternal and Newborn Health in Ghana	2021	Enos et al.	https://dx.doi.org/10.21106/ijma.425		No			No			Relative		No
ART use and associated factors among HIV positive caregivers of orphans and vulnerable children in Tanzania	2020	Exavery et al.	https://doi.org/10.1186/s12889-020-09361-6	Yes/No	No			No			Relative		No
Trends and causes of socioeconomic inequalities in maternal healthcare in Ghana, 2003-2014	2019	Fenny et al.	https://doi.org/10.1108/ijse-03-2018-0148		No			No			Relative		No

1. BIBLIOGRAPHIC SOURCE													
**Title**	**Year**	**First author**	**UHL**	**Measure**	**Sexual orientation**	**Name (acronym)**	**Measure**	**Other**	**Name (acronym)**	**Measure**	**Technique used for measureme nt of**		**Family planning (FP)**
Determination of the predictive factors of long-lasting insecticide-treated net ownership and utilisation in the Bamenda Health District of Cameroon	2017	Fokam et al.	https://doi.org/10.1186/s12889-017-4155-5		No			No			Relative		No
The free caesareans policy in low-income settings: An interrupted time series analysis in Mali (2003-2012)	2014	Fournier et al.	https://doi.org/10.1371/journal.pone.0105130		No			No			Absolute		No

1. BIBLIOGRAPHIC SOURCE													
**Title**	**Year**	**First author**	**UHL**	**Measure**	**Sexual orientation**	**Name (acronym)**	**Measure**	**Other**	**Name (acronym)**	**Measure**	**Technique used for measurement of**		**Family planning (FP)**
DO BETTER INSTITUTIONS BROADEN ACCESS TO SANITATION IN SUB-SAHARA AFRICA?	2021	Francois et al.	https://doi.org/10.1111/coep.12512		No			No			Absolute		No

1. BIBLIOGRAPHIC SOURCE													
**Title**	**Year**	**First author**	**UHL**	**Measure**	**Sexual orientation**	**Name (acronym)**	**Measure**	**Other**	**Name (acronym)**	**Measure**	**Technique used for measurement of**		**Family planning (FP)**
Ethnic disparities in utilisation of maternal health care services in Ghana: evidence from the 2007 Ghana Maternal Health Survey	2016	Ganle	https://doi.org/10.1080/13557858.2015.1015499		No			No			Absolute		No
Risky sexual behaviour and contraceptive use in contexts of displacement: Insights from a cross-sectional survey of female adolescent refugees in Ghana	2019	Ganle et al.	https://doi.org/10.1186/s12939-019-1031-1		No			No			Unclear		Yes
Understanding how distance to facility and quality of care affect maternal health service utilization in Kenya and Haiti: A comparative geographic information system study	2019	Gao & Kelley	https://doi.org/10.4081/gh.2019.690		No			No			Relative		No

1. BIBLIOGRAPHIC SOURCE													
**Title**	**Year**	**First author**	**UHL**	**Measure**	**Sexual orientation**	**Name (acronym)**	**Measure**	**Other**	**Name (acronym)**	**Measure**	**Technique used for measureme nt of**		**Family planning (FP)**
Gender differences in the use of insecticide-treated nets after a universal free distribution campaign in Kano State, Nigeria: Post-campaign survey results	2013	Garley et al.	https://doi.org/10.1186/1475-2875-12-119		No			No			Relative		No
Demand satisfied by modern contraceptive among married women of reproductive age in Kenya	2021	Gichangi et al.	https://doi.org/10.1371/journal.pone.0248393		No			No			Relative		Yes
LLIN Evaluation in Uganda Project (LLINEUP): Factors associated with ownership and use of long-lasting insecticidal nets in Uganda: A cross-sectional survey of 48 districts ISRCTN17516395 ISRCTN	2018	Gonahasa et al.	https://doi.org/10.1186/s12936-018-2571-3		No			No			Relative		No

1. BIBLIOGRAPHIC SOURCE												
**Title**	**Year**	**First author**	**UHL**	**Measure**	**Sexual orientation**	**Name (acronym)**	**Measure**	**Other**	**Name (acronym)**	**Measure**	**Technique used for measurement of**	**Family planning (FP)**
ART adherence and viral suppression are high among most non-pregnant individuals with early-stage, asymptomatic HIV infection: an observational study from Uganda and South Africa	2019	Haberer et al.	https://doi.org/10.1002/jia2.25232		No			No			Absolute	No
Low immunization coverage in Wonago district, southern Ethiopia: A community-based cross-sectional study	2019	Hailu et al.	https://doi.org/10.1371/journal.pone.0220144		No			No			Relative	No

1. BIBLIOGRAPHIC SOURCE												
Title	Year	First author	UHL	Measure	Sexual orientation	Name (acronym)	Measure	Other	Name (acronym)	Measure	Technique used for measurement of	Family planning (FP)
Towards universal health coverage: The role of within-country wealth-related inequality in 28 countries in sub-Saharan Africa	2011	Hosseinpoor et al.	https://doi.org/10.2471/BLT.11.087536		No			No			Absolute & relative	Yes

1. BIBLIOGRAPHIC SOURCE													
**Title**	**Year**	**First author**	**UHL**	**Measure**	**Sexual orientation**	**Name (acronym)**	**Measure**	**Other**	**Name (acronym)**	**Measure**	**Technique used for measureme nt of**		**Family planning (FP)**
Patterns and trends of contraceptive use among sexually active adolescents in Burkina Faso, Ethiopia, and Nigeria: evidence from cross-sectional studies	2015	Hounton et al.	https://doi.org/10.3402/gha.v8.29737		No			No			Relative		Yes
Towards universal health coverage for reproductive health services in Ethiopia: two policy recommendations	2015	Onarheim et al.	https://doi.org/10.1186/s12939-015-0218-3		No			Yes			Absolute & relative		Yes

1. BIBLIOGRAPHIC SOURCE												
**Title**	**Year**	**First author**	**UHL**	**Measure**	**Sexual orientation**	**Name (acronym)**	**Measure**	**Other**	**Name (acronym)**	**Measure**	**Technique used for measurement of**	**Family planning (FP)**
Modern Contraception: Uptake and Correlates among Women of Reproductive Age-Group in a Rural Community of Osun State, Nigeria	2020	Idowu et al.	https://doi.org/10.4314/ejhs.v30i4.8		No			No			Relative	Yes
Determinants of geographical inequalities for DTP3 vaccine coverage in sub-Saharan Africa	2020	Ikilezi et al.	https://doi.org/10.1016/j.vaccine.2020.03.005		No			No			Absolute & relative	No
Influence of women's decision-making autonomy on antenatal care utilisation and institutional delivery services in Nigeria: evidence from the Nigeria Demographic and Health Survey 2018	2022	Imo	https://doi.org/10.1186/s12884-022-04478-5		No			No			Relative	No

1. BIBLIOGRAPHIC SOURCE													
Title	Year	First author	UHL	Measure	Sexual orientation	Name (acronym)	Measure	Other	Name (acronym)	Measure	Technique used for measurement of		Family planning (FP)
Individual and community-level determinants of cervical cancer screening in Zimbabwe: a multi-level analyses of a nationwide survey	2022	Isabirye et al.	https://doi.org/10.1186/s12905-022-01881-0		No			No			Relative		No
Two decades of maternity care fee exemption policies in Ghana: have they benefited the poor?	2016	Johnson et al.	https://doi.org/10.1093/heapol/czv017		No			No			Relative		No

1. BIBLIOGRAPHIC SOURCE													
**Title**	**Year**	**First author**	**UHL**	**Measure**	**Sexual orientation**	**Name (acronym)**	**Measure**	**Other**	**Name (acronym)**	**Measure**	**Technique used for measurement of**		**Family planning (FP)**
Socioeconomic inequalities in access to skilled birth attendance among urban and rural women in low-income and middle-income countries	2018	Joseph et al.	https://doi.org/10.1136/bmjgh-2018-000898		No			No			Relative		No

1. BIBLIOGRAPHIC SOURCE												
**Title**	**Year**	**First author**	**UHL**	**Measure**	**Sexual orientation**	**Name (acronym)**	**Measure**	**Other**	**Name (acronym)**	**Measure**	**Technique used for measurement of**	**Family planning (FP)**
Investigating the disparities in cervical cancer screening among Namibian women	2015	Kangmennaang et al.	https://doi.org/10.1016/j.ygyno.2015.05.036		No			No			Relative	No
Contraceptive use and needs among adolescent women aged 15-19: Regional and global estimates and projections from 1990 to 2030 from a Bayesian hierarchical modelling study	2021	Kantorová et al.	https://doi.org/10.1371/journal.pone.0247479		No			No			Absolute, relative & graph	Yes
Changes in equity of maternal, newborn, and child health care practices in 115 districts of rural Ethiopia: Implications for the health extension program	2015	Karim et al.	https://doi.org/10.1186/s12884-015-0668-z		No			Yes			Relative	Yes

1. BIBLIOGRAPHIC SOURCE													
**Title**	**Year**	**First author**	**UHL**	**Measure**	**Sexual orientation**	**Name (acronym)**	**Measure**	**Other**	**Name (acronym)**	**Measure**	**Technique used for measurement of**		**Family planning (FP)**
Contraceptive dynamics during COVID-19 in sub-Saharan Africa: Longitudinal evidence from Burkina Faso and Kenya	2021	Karp et al.	https://doi.org/10.1136/bmjsrh-2020-200944		No			Yes			Relative		Yes
Long-lasting insecticidal net source, ownership and use in the context of universal coverage: A household survey in eastern Rwanda	2015	Kateera et al.	https://doi.org/10.1186/s12936-015-0915-9		No			Yes			Relative		No
A quasi-experimental evaluation of an interpersonal communication intervention to increase insecticide-treated net use among children in Zambia	2012	Keating et al.	https://doi.org/10.1186/1475-2875-11-313		No			Yes			Relative		No

1. BIBLIOGRAPHIC SOURCE												
**Title**	**Year**	**First author**	**UHL**	**Measure**	**Sexual orientation**	**Name (acronym)**	**Measure**	**Other**	**Name (acronym)**	**Measure**	**Technique used for measurement of**	**Family planning (FP)**
Assessment of Inequalities in Coverage of Essential Reproductive, Maternal, Newborn, Child, and Adolescent Health Interventions in Kenya	2018	Keats et al.	https://doi.org/10.1001/jamanetworkopen.2018.5152		No			No			Relative & graph	Yes
Charting health system reconstruction in post-war Liberia: a comparison of rural vs. remote healthcare utilization	2016	Kentoffioet al.	https://doi.org/10.1186/s12913-016-1709-7		No			No			Absolute & graph	No

1. BIBLIOGRAPHIC SOURCE													
**Title**	**Year**	**First author**	**UHL**	**Measure**	**Sexual orientation**	**Name (acronym)**	**Measure**	**Other**	**Name (acronym)**	**Measure**	**Technique used for measurement of**		**Family planning (FP)**
A Transparent Universal Health Coverage Index with Decomposition by Socioeconomic Groups: Application in Asian and African Settings	2019	Khan et al.	https://doi.org/10.1007/s40258-019-00464-9		No			No			Relative		No
Effect of user preferences on ITN use: a review of literature and data	2017	Koenker & Yukich	https://doi.org/10.1186/s12936-017-1879-8		No			No			Absolute, relative & graph		No

1. BIBLIOGRAPHIC SOURCE												
**Title**	**Year**	**First author**	**UHL**	**Measure**	**Sexual orientation**	**Name (acronym)**	**Measure**	**Other**	**Name (acronym)**	**Measure**	**Technique used for measurement of**	**Family planning (FP)**
Insecticide-treated net use before and after mass distribution in a fishing community along Lake Victoria, Kenya: successes and unavoidable pitfalls	2014	Larson et al.	http://www.malariajournal.com/content/13/1/466		No			Yes			Absolute & relative	No
Lifetime Prevalence of Cervical Cancer Screening in 55 Low- and Middle-Income Countries	2020	Lemp et al.	https://doi.org/10.1001/jama.2020.16244		No			Yes	Screening by HDI, Gender equality Indices and Health worker density		Absolute & relative	No

1. BIBLIOGRAPHIC SOURCE												
Title	Year	First author	UHL	Measure	Sexual orientation	Name (acronym)	Measure	Other	Name (acronym)	Measure	Technique used for measurement of	Family planning (FP)
Financial accessibility and user fee reforms for maternal healthcare in five sub-Saharan countries: a quasi-experimental analysis	2016	Leone et al.	https://dx.doi.org/10.1136/bmjopen-2015-009692		No			No			Relative	No
Antiretroviral treatment coverage in a rural district in Tanzania--a modeling study using empirical data	2015	Levira et al.	https://doi.org/10.1186/s12889-015-1460-8		No			No			Absolute	No
The extent of universal health coverage for maternal health services in eastern uganda: A cross sectional study	2021	Lindberg et al.	https://dx.doi.org/10.1007/s10995-021-03357-3		No			No			Absolute	No

1. BIBLIOGRAPHIC SOURCE												
**Title**	**Year**	**First author**	**UHL**	**Measure**	**Sexual orientation**	**Name (acronym)**	**Measure**	**Other**	**Name (acronym)**	**Measure**	**Technique used for measurement of**	**Family planning (FP)**
Freely distributed bed-net use among Chano Mille residents, south Ethiopia: A longitudinal study	2013	Loha et al.	https://doi.org/10.1186/1475-2875-12-23		No			No			Relative	No
Determinants of hanging and use of ITNs in the context of near universal coverage in Zambia	2012	Macintyre et al.	https://doi.org/10.1093/heapol/czr042		No			Yes			Relative	No

1. BIBLIOGRAPHIC SOURCE												
**Title**	**Year**	**First author**	**UHL**	**Measure**	**Sexual orientation**	**Name (acronym)**	**Measure**	**Other**	**Name (acronym)**	**Measure**	**Technique used for measurement of**	**Family planning (FP)**
Determinants and Consequences of Failure of Linkage to Antiretroviral Therapy at Primary Care Level in Blantyre, Malawi: A Prospective Cohort Study	2012	MacPherson et al.	https://doi.org/10.1371/journal.pone.0044794		No			Yes			Relative & graph	No
Disability and sexual and reproductive health service utilisation in Uganda: an intersectional analysis of demographic and health surveys between 2006 and 2016	2022	Mac-Seing et al.	https://doi.org/10.1186/s12889-022-12708-w	Overall (No difficulty), Difficulty seeing, Difficulty hearing, Difficulty walking / climbing steps, Difficulty remembering / concentrating, Difficulty with self-care, Difficulty communicating	No			Yes			Relative & graph	Yes

1. BIBLIOGRAPHIC SOURCE												
**Title**	**Year**	**First author**	**UHL**	**Measure**	**Sexual orientation**	**Name (acronym)**	**Measure**	**Other**	**Name (acronym)**	**Measure**	**Technique used for measurement of**	**Family planning (FP)**
Insight into Nigeria's progress towards the universal coverage of reproductive, maternal, newborn and child health services: a secondary data analysis	2022	Mafiana et al.	http://dx.doi.org/10.1136/bmjopen-2022-061595		No			No			Relative & graph	Yes
Implementation of Urban Health Equity Assessment and Response Tool: a Case of Matsapha, Swaziland	2018	Makadzange et al.	https://doi.org/10.1007/s11524-018-0241-y		No			Yes			Relative & qualitative	Yes
Effects of door-to-door hang-up visits on the use of long-lasting insecticide-treated mosquito nets in the democratic republic of the congo: A cluster randomized controlled trial	2021	Mankadi and Jin	https://doi.org/10.3390/ijerph18179048		No			No			Absolute & relative	No

1. BIBLIOGRAPHIC SOURCE												
**Title**	**Year**	**First author**	**UHL**	**Measure**	**Sexual orientation**	**Name (acronym)**	**Measure**	**Other**	**Name (acronym)**	**Measure**	**Technique used for measurement of**	**Family planning (FP)**
Effect of bed net colour and shape preferences on bed net usage: a secondary data analysis of the 2017 Malawi Malaria Indicator Survey	2020	Mategula et al.	https://doi.org/10.1186/s12936-020-03499-9		No			Yes			Relative	No
HIV care coverage among HIV-positive adolescent girls and young women in South Africa: Results from the HERStory Study	2021	Mathews et al.	https://doi.org/10.7196/SAMJ.2021.v111i5.15351		No			Yes			Relative & graph	No
Facilitators and barriers to retention in care under universal antiretroviral therapy (Option B+) for the Prevention of Mother to Child Transmission of HIV (PMTCT): A narrative review	2021	Mbeya Munkhondya et al.	https://doi.org/10.1016/j.ijans.2021.100372		No			Yes			Qualitative	No

1. BIBLIOGRAPHIC SOURCE												
**Title**	**Year**	**First author**	**UHL**	**Measure**	**Sexual orientation**	**Name (acronym)**	**Measure**	**Other**	**Name (acronym)**	**Measure**	**Technique used for measurement of**	**Family planning (FP)**
The consequences of declining population access to insecticide-treated nets (ITNs) on net use patterns and physical degradation of nets after 22 months of ownership	2021	Mboma et al.	https://doi.org/10.1186/s12936-021-03686-2		No			Yes			Relative & graph	No
Mosquito net coverage in years between mass distributions: a case study of Tanzania, 2013	2018	Mboma et al.	https://doi.org/10.1186/s12936-018-2247-z		No			Yes			Relative & graph	No
Factors associated with contraceptive use in Tigray, North Ethiopia	2017	Medhanyie et al.	https://doi.org/10.1186/s12978-017-0281-x		No			Yes			Relative	Yes

1. BIBLIOGRAPHIC SOURCE												
**Title**	**Year**	**First author**	**UHL**	**Measure**	**Sexual orientation**	**Name (acronym)**	**Measure**	**Other**	**Name (acronym)**	**Measure**	**Technique used for measurement of**	**Family planning (FP)**
Adolescent sexual and reproductive health in sub-Saharan Africa: who is left behind?	2020	Melesse et al.	https://doi.org/10.1136/bmjgh-2019-002231		No			Yes			Relative	Yes
Sub-national levels and trends in contraceptive prevalence, unmet need, and demand for family planning in Nigeria with survey uncertainty	2019	Mercer et al.	https://doi.org/10.1186/s12889-019-8043-z		No			Yes	Age-parity group		Relative	Yes

1. BIBLIOGRAPHIC SOURCE												
**Title**	**Year**	**First author**	**UHL**	**Measure**	**Sexual orientation**	**Name (acronym)**	**Measure**	**Other**	**Name (acronym)**	**Measure**	**Technique used for measurement of**	**Family planning (FP)**
Exploring inequities in skilled care at birth among migrant population in a metropolitan city Addis Ababa, Ethiopia; A qualitative study	2014	Mirkuzie	http://www.equityhealthj.com/content/13/1/110		No			No			Qualitative	No
Factors associated with the use of mosquito bed nets: results from two cross-sectional household surveys in Zambezia Province, Mozambique	2016	Moon et al.	https://doi.org/10.1186/s12936-016-1250-5		No			Yes			Relative	No
Sociocultural and Institutional Constraints to Family Planning Uptake Among Migrant Female Head Porters in Madina, a Suburb of Accra, Ghana	2021	Munemo et al.	https://doi.org/10.1177/088610992095441 9		No			Yes			Qualitative	Yes

1. BIBLIOGRAPHIC SOURCE												
Title	Year	First author	UHL	Measure	Sexual orientation	Name (acronym)	Measure	Other	Name (acronym)	Measure	Technique used for measurement of	Family planning (FP)
Reframing non-communicable diseases and injuries for equity in the era of universal health coverage: Findings and recommendations from the Kenya NCDI poverty commission	2021	Mwangi et al.	https://doi.org/10.5334/aogh.3085		No			No			Relative & graph	No

1. BIBLIOGRAPHIC SOURCE												
**Title**	**Year**	**First author**	**UHL**	**Measure**	**Sexual orientation**	**Name (acronym)**	**Measure**	**Other**	**Name (acronym)**	**Measure**	**Technique used for measurement of**	**Family planning (FP)**
Inequities and their determinants in coverage of maternal health services in Burkina Faso	2018	Mwase et al.	https://doi.org/10.1186/s12939-018-0770-8		No			Yes			Absolute & relative	No

1. BIBLIOGRAPHIC SOURCE												
**Title**	**Year**	**First author**	**UHL**	**Measure**	**Sexual orientation**	**Name (acronym)**	**Measure**	**Other**	**Name (acronym)**	**Measure**	**Technique used for measurement of**	**Family planning (FP)**
A Cross-Sectional Study on Hypertension Medication Adherence in a High-Burden Region in Namibia: Exploring Hypertension Interventions and Validation of the Namibia Hill-Bone Compliance Scale	2022	Nakwafila et al.	https://doi.org/10.3390/ijerph19074416		No			Yes			Absolute & relative	No
Assessing Adherence to Antihypertensive Therapy in Primary Health Care in Namibia: Findings and Implications	2017	Nashilongo et al.	https://doi.org/10.1007/s10557-017-6756-8		No			Yes			Relative	No

1. BIBLIOGRAPHIC SOURCE													
**Title**	**Year**	**First author**	**UHL**	**Measure**	**Sexual orientation**	**Name (acronym)**	**Measure**	**Other**	**Name (acronym)**	**Measure**	**Technique used for measurement of**		**Family planning (FP)**
Incomplete vaccination and associated factors among children aged 12–23 months in South Africa: an analysis of the South African demographic and health survey 2016	2021	Ndwandwe et al.	https://doi.org/10.1080/21645515.2020.1791509		No			Yes			Absolute & relative		No
Determining the effective coverage of maternal and child health services in Kenya, using demographic and health survey data sets: tracking progress towards universal health coverage	2017	Nguhiu et al.	https://doi.org/10.1111/tmi.12841		No			No			Relative		Yes

1. BIBLIOGRAPHIC SOURCE													
**Title**	**Year**	**First author**	**UHL**	**Measure**	**Sexual orientation**	**Name (acronym)**	**Measure**	**Other**	**Name (acronym)**	**Measure**	**Technique used for measurement of**		**Family planning (FP)**
Coverage and usage of insecticide treated nets (ITNs) within households: associated factors and effect on the prevalance of malaria parasitemia in the Mount Cameroon area	2019	Njumkeng et al.	https://doi.org/10.1186/s12889-019-7555-x		No			Yes			Absolute & relative		No
Socioeconomic inequalities in maternal health care utilization in Ghana	2019	Novignon et al.	https://doi.org/10.1186/s12939-019-1043-x		No			Yes	National Health Insurance Scheme (NHIS) covered	Yes, No	Relative & graph		No

1. BIBLIOGRAPHIC SOURCE												
**Title**	**Year**	**First author**	**UHL**	**Measure**	**Sexual orientation**	**Name (acronym)**	**Measure**	**Other**	**Name (acronym)**	**Measure**	**Technique used for measurement of**	**Family planning (FP)**
Why rural women do not use primary health centres for pregnancy care: Evidence from a qualitative study in Nigeria	2019	Ntoimo et al.	https://doi.org/10.1186/s12884-019-2433-1		No			Yes			Qualitative	No
Long-lasting insecticidal net (LLIN) ownership, use and cost of implementation after a mass distribution campaign in Kasaï Occidental Province, Democratic Republic of Congo	2017	Ntuku et al.	https://doi.org/10.1186/s12936-016-1671-1		No			Yes			Relative & graph	No
Explaining socioeconomic disparities and gaps in the use of antenatal care services in 36 countries in sub-Saharan Africa	2021	Obse & Ataguba	https://doi.org/10.1093/heapol/czab036		No			Yes			Relative & graph	No

1. BIBLIOGRAPHIC SOURCE													
**Title**	**Year**	**First author**	**UHL**	**Measure**	**Sexual orientation**	**Name (acronym)**	**Measure**	**Other**	**Name (acronym)**	**Measure**	**Technique used for measurement of**		**Family planning (FP)**
Sociodemographic factors associated with the use of insecticide treated nets among under-fives in Nigeria: Evidence from a national survey	2022	Ojo et al.	https://doi.org/10.1177/00494755221110374		No			Yes			Absolute & relative		No
Towards making efficient use of household resources for appropriate prevention of malaria: investigating households' ownership, use and expenditures on ITNs and other preventive tools in Southeast Nigeria	2014	Onwujekwe et al.	http://www.biomedcentral.com/1471-2458/14/315		No			No			Absolute & relative		No

1. BIBLIOGRAPHIC SOURCE												
Title	Year	First author	UHL	Measure	Sexual orientation	Name (acronym)	Measure	Other	Name (acronym)	Measure	Technique used for measurement of	Family planning (FP)
Demographic disparities in unimproved drinking water and sanitation in Ghana: A nationally representative cross-sectional study	2022	Oppong et al.	http://dx.doi.org/10.1136/bmjopen-2021-060595		No			No			Absolute & relative	No
Inequities in Access to Maternal Health Care in Enugu State: Implications for Universal Health Coverage to Meet Vision 2030 in Nigeria	2019	Ozumba et al.	https://doi.org/10.1177/0272684X18819977		No			Yes			Relative & qualitative	No

1. BIBLIOGRAPHIC SOURCE												
**Title**	**Year**	**First author**	**UHL**	**Measure**	**Sexual orientation**	**Name (acronym)**	**Measure**	**Other**	**Name (acronym)**	**Measure**	**Technique used for measurement of**	**Family planning (FP)**
How do supply- and demand-side interventions influence equity in healthcare utilisation? Evidence from maternal healthcare in Senegal	2019	Parmar & Banerjee	https://doi.org/10.1016/j.socscimed.2019.112582		No			No			Relative & graph	No
Temporal and regional variations in use, equity and quality of antenatal care in Egypt: A repeat cross-sectional analysis using Demographic and Health Surveys	2019	Pugliese-Garcia et al.	https://doi.org/10.1186/s12884-019-2409-1		No			No			Relative & graph	No

1. BIBLIOGRAPHIC SOURCE												
**Title**	**Year**	**First author**	**UHL**	**Measure**	**Sexual orientation**	**Name (acronym)**	**Measure**	**Other**	**Name (acronym)**	**Measure**	**Technique used for measurement of**	**Family planning (FP)**
Geographical Inequalities in Use of Improved Drinking Water Supply and Sanitation across Sub-Saharan Africa: Mapping and Spatial Analysis of Cross-sectional Survey Data	2014	Pullan et al.	https://doi.org/10.1371/journal.pmed.1001626		No			No			Relative & graph	No
Individual and Network Factors Associated With HIV Care Continuum Outcomes Among Nigerian MSM Accessing Health Care Services	2018	Ramadhani et al.	https://dx.doi.org/10.1097/QAI.0000000000001756		No			Yes			Relative	No
Non-adherence to long-lasting insecticide treated bednet use following successful malaria control in Tororo, Uganda	2020	Rek et al.	https://doi.org/10.1371/journal.pone.0243303		No			Yes			Relative & graph	No

1. BIBLIOGRAPHIC SOURCE												
**Title**	**Year**	**First author**	**UHL**	**Measure**	**Sexual orientation**	**Name (acronym)**	**Measure**	**Other**	**Name (acronym)**	**Measure**	**Technique used for measurement of**	**Family planning (FP)**
Design, implementation and evaluation of a national campaign to deliver 18 million free long-lasting insecticidal nets to uncovered sleeping spaces in Tanzania	2013	Renggli et al.	https://doi.org/10.1186/1475-2875-12-85		No			No			Relative & graph	No
A long way to go - Estimates of combined water, sanitation and hygiene coverage for 25 sub-Saharan African countries	2017	Roche et al.	https://doi.org/10.1371/journal.pone.0171783		No			No			Absolute & graph	No

1. BIBLIOGRAPHIC SOURCE												
**Title**	**Year**	**First author**	**UHL**	**Measure**	**Sexual orientation**	**Name (acronym)**	**Measure**	**Other**	**Name (acronym)**	**Measure**	**Technique used for measurement of**	**Family planning (FP)**
Determinants of bed net use in southeast Nigeria following mass distribution of LLINs: Implications for social behavior change interventions	2015	Russell et al.	https://doi.org/10.1371/journal.pone.0139447		No			Yes			Relative	No
Factors associated with the non-use of insecticide-treated nets in Rwandan children	2016	Ruyange et al.	https://doi.org/10.1186/s12936-016-1403-6		No			Yes			Relative	No

1. BIBLIOGRAPHIC SOURCE													
**Title**	**Year**	**First author**	**UHL**	**Measure**	**Sexual orientation**	**Name (acronym)**	**Measure**	**Other**	**Name (acronym)**	**Measure**	**Technique used for measurement of**		**Family planning (FP)**
Wealth Status, Health Insurance, and Maternal Health Care Utilization in Africa: Evidence from Gabon	2020	Sanogo & Yaya	https://dx.doi.org/10.1155/2020/4036830		No			Yes			Absolute & graph		No

1. BIBLIOGRAPHIC SOURCE												
**Title**	**Year**	**First author**	**UHL**	**Measure**	**Sexual orientation**	**Name (acronym)**	**Measure**	**Other**	**Name (acronym)**	**Measure**	**Technique used for measurement of**	**Family planning (FP)**
Factors associated with use of insecticide-treated net for malaria prevention in Manica District, Mozambique: a community-based cross-sectional survey	2021	Scott et al.	https://doi.org/10.1186/s12936-021-03738-7		No			Yes			Relative & graph	No
Healthcare utilisation, cancer screening and potential barriers to accessing cancer care in rural South West Nigeria: a cross-sectional study	2021	Sharma et al.	https://doi.org/10.1136/bmjopen-2020-040352		No			Yes	Insurance status	Yes, No	Absolute & relative	No

1. BIBLIOGRAPHIC SOURCE													
**Title**	**Year**	**First author**	**UHL**	**Measure**	**Sexual orientation**	**Name (acronym)**	**Measure**	**Other**	**Name (acronym)**	**Measure**	**Technique used for measurement of**		**Family planning (FP)**
Socio-economic inequalities in ANC attendance among mothers who gave birth in the past 12 months in Debre Brehan town and surrounding rural areas, North East Ethiopia: A community-based survey	2019	Shibre & Mekonnen	https://doi.org/10.1186/s12978-019-0768-8		No			Yes	1) Time to reach nearest health centers; 2) time to reach nearest hospital; 3) Insurance coverage	1-2) <15 min, 15–29 min, 30–59 min and 1–2 h; 3) Yes, No	Relative		No

1. BIBLIOGRAPHIC SOURCE													
**Title**	**Year**	**First author**	**UHL**	**Measure**	**Sexual orientation**	**Name (acronym)**	**Measure**	**Other**	**Name (acronym)**	**Measure**	**Technique used for measurement of**		**Family planning (FP)**
Individual and environmental characteristics associated with immunization of children in rural areas of Burkina Faso: A multi-level analysis	2007	Sia et al.	https://pubmed.ncbi.nlm.nih.gov/18299262/		No			Yes			Relative		No
Inequalities in access and utilization of maternal, newborn and child health services in sub-saharan africa: A special focus on urban settings	2021	Sidze et al.	https://dx.doi.org/10.1007/s10995-021-03250-z		No			Yes	Status in the country of residence	Migrant, Resident	Relative		No

1. BIBLIOGRAPHIC SOURCE												
**Title**	**Year**	**First author**	**UHL**	**Measure**	**Sexual orientation**	**Name (acronym)**	**Measure**	**Other**	**Name (acronym)**	**Measure**	**Technique used for measurement of**	**Family planning (FP)**
Insecticide-treated bed net access and use among preschool children in Nouna District, Burkina Faso	2020	Sié et al.	https://dx.doi.org/10.1093/inthealth/ihaa003		No			Yes	Child's mother resides in the house	No, Yes	Relative	No
Evidence of improving antiretroviral therapy treatment delays: an analysis of eight years of programmatic outcomes in Blantyre, Malawi	2013	Sloan et al.	http://www.biomedcentral.com/1471-2458/13/490		No			Yes			Absolute & relative	No
Low use of long-lasting insecticidal nets for malaria prevention in south-central Ethiopia: A community-based cohort study	2019	Solomon et al.	https://doi.org/10.1371/journal.pone.0210578		No			No			Absolute, relative & graph	No

1. BIBLIOGRAPHIC SOURCE												
**Title**	**Year**	**First author**	**UHL**	**Measure**	**Sexual orientation**	**Name (acronym)**	**Measure**	**Other**	**Name (acronym)**	**Measure**	**Technique used for measurement of**	**Family planning (FP)**
Empowerment and use of modern contraceptive methods among married women in Burkina Faso: a multilevel analysis	2021	Some et al.	https://dx.doi.org/10.1186/s12889-021-11541-x		No			Yes			Relative	Yes
Evaluation of the 2011 long-lasting, insecticide-treated net distribution for universal coverage in Togo	2013	Stevens et al.	http://www.malariajournal.com/content/12/1/162		No			No			Relative	No
On the way to universal coverage of maternal services in Iringa rural District in Tanzania. Who is yet to be reached?	2016	Straneo et al.	https://doi.org/10.4314/ahs.v16i2.10		No			Yes	1) Walking distance to facility; ; 2) Parity (range 1-10)	1) ≤1 hour, >1 hour; 2) 1, 2-4, ≥5	Absolute & relative	No

1. BIBLIOGRAPHIC SOURCE												
**Title**	**Year**	**First author**	**UHL**	**Measure**	**Sexual orientation**	**Name (acronym)**	**Measure**	**Other**	**Name (acronym)**	**Measure**	**Technique used for measurement of**	**Family planning (FP)**
Evaluation of long-lasting insecticidal net distribution through schools in Southern Tanzania	2022	Stuck et al.	https://doi.org/10.1093/heapol/czab140		No			No			Relative & graph	No
Not all inequalities are equal: differences in coverage across the continuum of reproductive health services	2019	Sully et al.	https://dx.doi.org/10.1136/bmjgh-2019-001695		No			Yes			Absolute & relative	Yes

1. BIBLIOGRAPHIC SOURCE												
**Title**	**Year**	**First author**	**UHL**	**Measure**	**Sexual orientation**	**Name (acronym)**	**Measure**	**Other**	**Name (acronym)**	**Measure**	**Technique used for measurement of**	**Family planning (FP)**
Determinants of long-lasting insecticidal net ownership and utilization in malaria transmission regions: Evidence from Zimbabwe Demographic and Health Surveys	2019	Tapera	https://doi.org/10.1186/s12936-019-2912-x		No			Yes			Relative	No
Sociodemographic inequities in cervical cancer screening, treatment and care amongst women aged at least 25 years: evidence from surveys in Harare, Zimbabwe	2019	Tapera et al.	https://doi.org/10.1186/s12889-019-6749-6		No			Yes	Medical insurance/aid	Yes, No	Relative	No

1. BIBLIOGRAPHIC SOURCE												
**Title**	**Year**	**First author**	**UHL**	**Measure**	**Sexual orientation**	**Name (acronym)**	**Measure**	**Other**	**Name (acronym)**	**Measure**	**Technique used for measurement of**	**Family planning (FP)**
Factors associated to bed net use in Cameroon: a retrospective study in Mfou health district in the Centre Region	2012	Tchinda et al.	http://www.panafrican-med-journal.com/content/article/12/112/full/		No			Yes	1) Status in the household (Children/Parents/Other); 2) Pregnant woman (Yes/No); 3) Household living standard (Low/Medium/High)		Absolute, relative & graph	No
Cervical cancer screening uptake and correlates among HIV-infected women: a cross-sectional survey in Cote d'Ivoire, West Africa	2019	Tchounga et al.	https://dx.doi.org/10.1136/bmjopen-2019-029882		No			No			Absolute & relative	No

1. BIBLIOGRAPHIC SOURCE												
**Title**	**Year**	**First author**	**UHL**	**Measure**	**Sexual orientation**	**Name (acronym)**	**Measure**	**Other**	**Name (acronym)**	**Measure**	**Technique used for measurement of**	**Family planning (FP)**
Duration and determinants of delayed tuberculosis diagnosis and treatment in high-burden countries: a mixed-methods systematic review and meta-analysis	2021	Teo et al.	https://doi.org/10.1186/s12931-021-01841-6		No			Yes	Tobacco and subance use	Smoking/non smoking	Relative	No
Skilled delivery inequality in Ethiopia: To what extent are the poorest and uneducated mothers benefiting?	2017	Tesfaye et al.	https://doi.org/10.1186/s12939-017-0579-x		No			No			Relative	No

1. BIBLIOGRAPHIC SOURCE												
**Title**	**Year**	**First author**	**UHL**	**Measure**	**Sexual orientation**	**Name (acronym)**	**Measure**	**Other**	**Name (acronym)**	**Measure**	**Technique used for measurement of**	**Family planning (FP)**
Investigating the association between pregnancy intention and insecticide-treated bed net (ITN) use: A cross-sectional study of pregnant women in Rwanda	2015	Thogarapalli et al.	https://doi.org/10.1007/s10389-015-0676-5		No			Yes	Pregnancy intention	Wanted, wanted but mistimed, unwanted	Relative	No
Success of Senegal's first nationwide distribution of long-lasting insecticide-treated nets to children under five - Contribution toward universal coverage	2011	Thwing et al.	http://www.malariajournal.com/content/10/1/86		No			Yes			Relative	No
Declines in Malaria Burden and all-cause child mortality following increases in control interventions in Senegal, 2005-2010	2017	Thwing et al.	https://doi.org/10.4269/ajtmh.16-0953		No			No			Relative	No

1. BIBLIOGRAPHIC SOURCE													
**Title**	**Year**	**First author**	**UHL**	**Measure**	**Sexual orientation**	**Name (acronym)**	**Measure**	**Other**	**Name (acronym)**	**Measure**	**Technique used for measurement of**		**Family planning (FP)**
Individual-level and community-level determinants of cervical cancer screening among Kenyan women: a multilevel analysis of a Nationwide survey	2017	Tiruneh et al.	https://doi.org/10.1186/s12905-017-0469-9		No			No	Health care access	Covered by healthcare access (yes/no)	Relative		No
Evaluation of the coverage and effective use rate of long-lasting insecticidal nets after nation-wide scale up of their distribution in Benin	2013	Tokponnon et al.	https://doi.org/10.1186/1756-3305-6-265		No			Yes	Seize of the household	0-1 member, 2-4 members, 5-7 members, 8 and more members	Relative		No
Factors associated with the upsurge in the use of delivery care services in Sierra Leone	2020	Tsawe & Susuman	https://doi.org/10.1016/j.puhe.2019.11.002		No			Yes	Media exposure	Yes/No	Relative		No

1. BIBLIOGRAPHIC SOURCE												
**Title**	**Year**	**First author**	**UHL**	**Measure**	**Sexual orientation**	**Name (acronym)**	**Measure**	**Other**	**Name (acronym)**	**Measure**	**Technique used for measurement of**	**Family planning (FP)**
Which family members use the best nets? An analysis of the condition of mosquito nets and their distribution within households in Tanzania	2010	Tsuang et al.	http://www.malariajournal.com/content/9/1/211		No			No			Relative & graph	No
Utilization of insecticide treated nets among pregnant women in enugu, South Eastern Nigeria	2013	Ugwu et al.	https://pubmed.ncbi.nlm.nih.gov/23771448/		No			No			Absolute & relative	No

1. BIBLIOGRAPHIC SOURCE													
**Title**	**Year**	**First author**	**UHL**	**Measure**	**Sexual orientation**	**Name (acronym)**	**Measure**	**Other**	**Name (acronym)**	**Measure**	**Technique used for measurement of**		**Family planning (FP)**
Finding the gap: Revealing local disparities in coverage of maternal, newborn and child health services in South Sudan using lot quality assurance sampling	2015	Valadez et al.	https://doi.org/10.1111/tmi.12613		No			No			Relative		No
Group Medical Visit and Microfinance Intervention for Patients With Diabetes or Hypertension in Kenya	2021	Vedanthan et al.	https://doi.org/10.1016/j.jacc.2021.03.002		No			No			Relative & graph		No

1. BIBLIOGRAPHIC SOURCE													
**Title**	**Year**	**First author**	**UHL**	**Measure**	**Sexual orientation**	**Name (acronym)**	**Measure**	**Other**	**Name (acronym)**	**Measure**	**Technique used for measurement of**		**Family planning (FP)**
Equity in Maternal Health in South Africa: Analysis of Health Service Access and Health Status in a National Household Survey	2013	Wabiri et al.	https://doi.org/10.1371/journal.pone.0073864		No			Yes	Household head	Yes/No	Relative		Yes
Persisting Regional Disparities in Modern Contraceptive Use and Unmet Need for Contraception among Nigerian Women	2019	Wang & Cao	https://doi.org/10.1155/2019/9103928		No			No			Relative		Yes

1. BIBLIOGRAPHIC SOURCE													
**Title**	**Year**	**First author**	**UHL**	**Measure**	**Sexual orientation**	**Name (acronym)**	**Measure**	**Other**	**Name (acronym)**	**Measure**	**Technique used for measurement of**		**Family planning (FP)**
Effective coverage of facility delivery in Bangladesh, Haiti, Malawi, Nepal, Senegal, and Tanzania	2019	Wang et al.	https://doi.org/10.1371/journal.pone.0217853		No			No			Absolute		No
Use of long-lasting insecticide-treated bed nets in a population with universal coverage following a mass distribution campaign in Uganda	2016	Wanzira et al.	https://doi.org/10.1186/s12936-016-1360-0		No			No			Relative		No

1. BIBLIOGRAPHIC SOURCE												
**Title**	**Year**	**First author**	**UHL**	**Measure**	**Sexual orientation**	**Name (acronym)**	**Measure**	**Other**	**Name (acronym)**	**Measure**	**Technique used for measurement of**	**Family planning (FP)**
Long-lasting insecticide-treated bed net ownership and use among children under five years of age following a targeted distribution in central Uganda	2014	Wanzira et al.	https://doi.org/10.1186/1475-2875-13-185		No			No			Relative	No
Evaluation of a national universal coverage campaign of long-lasting insecticidal nets in a rural district in north-west Tanzania	2012	West et al.	https://doi.org/10.1186/1475-2875-11-273		No			No			Relative	No
The impact of renewing long-lasting insecticide-treated nets in the event of malaria resurgence: Lessons from 10 years of net use in dielmo, Senegal	2021	Wotodjo et al.	https://doi.org/10.4269/AJTMH.20-0127		No			No			Absolute & graph	No

1. BIBLIOGRAPHIC SOURCE												
**Title**	**Year**	**First author**	**UHL**	**Measure**	**Sexual orientation**	**Name (acronym)**	**Measure**	**Other**	**Name (acronym)**	**Measure**	**Technique used for measurement of**	**Family planning (FP)**
Wealth and Education Inequities in Maternal and Child Health Services Utilization in Rural Ethiopia	2022	Wuneh et al.	https://doi.org/10.3390/ijerph19095421		No			Yes			Absolute, relative & graph	No
Inequalities in maternal health care utilization in Benin: A population based cross-sectional study	2018	Yaya et al.	https://doi.org/10.1186/s12884-018-1846-6		No			Yes	1)Women decision making power; 2) Parity	Women decision making power (Low,Moderate, High); 2) Parity (1-4, >4)	Relative	Yes

1. BIBLIOGRAPHIC SOURCE													
**Title**	**Year**	**First author**	**UHL**	**Measure**	**Sexual orientation**	**Name (acronym)**	**Measure**	**Other**	**Name (acronym)**	**Measure**	**Technique used for measurement of**		**Family planning (FP)**
Long-lasting insecticide-treated bed net ownership, utilization and associated factors among school-age children in Dara Mallo and Uba Debretsehay districts, Southern Ethiopia	2020	Zerdo et al.	https://dx.doi.org/10.1186/s12936-020-03437-9		No			Yes	Residence house	Private, Not private	Absolute & relative		No
Trends and projections of universal health coverage indicators in Ghana, 1995-2030: A national and subnational study	2019	Zhang et al.	https://doi.org/10.1371/journal.pone.0209126		No			No			Absolute, relative & graph		Yes

1. BIBLIOGRAPHIC SOURCE												
**Title**	**Year**	**First author**	**UHL**	**Measure**	**Sexual orientation**	**Name (acronym)**	**Measure**	**Other**	**Name (acronym)**	**Measure**	**Technique used for measurement of**	**Family planning (FP)**
Developing Malawi's Universal Health Coverage Index	2022	Mchenga et al.	https://doi.org/10.3389/frhs.2021.786186		No			Unclear			Relative	Yes
Socioeconomic Factors Associated with Compliance with Mass Drug Administration for Lymphatic Filariasis Elimination in Kenya: Descriptive Study Results	2012	Njomo et al.	https://www.proquest.com/docview/1115911778?pq-origsite=gscholar&fromopenview=true		No			No			Absolute & relative	No

1. BIBLIOGRAPHIC SOURCE												
**Title**	**Year**	**First author**	**UHL**	**Measure**	**Sexual orientation**	**Name (acronym)**	**Measure**	**Other**	**Name (acronym)**	**Measure**	**Technique used for measurement of**	**Family planning (FP)**
Equality analysis of main health indicators among children under 5 years in Uganda	2019	Elduma	http://dx.doi.org/10.4314/ejhs.v29i2.8		No			No			Relative & graph	No

1. BIBLIOGRAPHIC SOURCE				4. UNIVERSAL HEALTH COVERA								
**Title**	**Year**	**First author**	**UHL**	**Antenatal care (ANC)**	**Delivery care: Skilled birth attendance**	**Cesarean delivery**	**Postnatal care (PNC)**	**Child immunization (vaccine)**	**Care seeking for suspected pneumonia**	**Tuberculosis (TB) treatment**	**HIV therapy (ART)**	**Water and sanitation (WASH)**
Reproductive Plans And Utilization of Contraceptives Among Women Living With HIV	2019	Adeleye et al.	https://dx.doi.org/10.21106/ijma.277	No	No	No	No	No	No	No	No	No
Provision of immediate postpartum contraception to women living with HIV in the Eastern Cape, South Africa; a cross-sectional analysis	2020	Adeniyi et al.	https://doi.org/10.1186/s12978-020-01049-9	No	No	No	No	No	No	No	No	No
Moving Up the Sanitation Ladder: A Study of the Coverage and Utilization of Improved Sanitation Facilities and Associated Factors Among Households in Southern Ethiopia	2022	Afework et al.	https://doi.org/10.1177/1178630222108025	No	No	No	No	No	No	No	No	Yes

1. BIBLIOGRAPHIC SOURCE				4. UNIVERSAL HEALTH COVERA								
**Title**	**Year**	**First author**	**UHL**	**Antenatal care (ANC)**	**Delivery care: Skilled birth attendance**	**Cesarean delivery**	**Postnatal care (PNC)**	**Child immunization (vaccine)**	**Care seeking for suspected pneumonia**	**Tuberculosis (TB) treatment**	**HIV therapy (ART)**	**Water and sanitation (WASH)**
Maternal health care services utilisation in the context of 'Abiye' (safe motherhood) programme in Ondo State, Nigeria	2020	Ajayi et al.	https://doi.org/10.1186/s12889-020-08512-z	Yes	No	No	No	No	No	No	No	No
Slums, women and sanitary living in South-South Nigeria	2021	Akpabio et al.	https://doi.org/10.1007/s10901-020-09802-z	No	No	No	No	No	No	No	No	Yes
Leaving no child behind: Decomposing socioeconomic inequalities in child health for india and south africa	2021	Alaba et al.	https://doi.org/10.3390/ijerph18137114	No	No	No	No	Yes	No	No	No	No

1. BIBLIOGRAPHIC SOURCE				4. UNIVERSAL HEALTH COVERA								
Title	Year	First author	UHL	Antenatal care (ANC)	Delivery care: Skilled birth attendance	Cesarean delivery	Postnatal care (PNC)	Child immunization (vaccine)	Care seeking for suspected pneumonia	Tuberculosis (TB) treatment	HIV therapy (ART)	Water and sanitation (WASH)
Access to skilled attendant at birth and the coverage of the third dose of diphtheria-tetanus-pertussis vaccine across 14 West African countries - An equity analysis	2020	Alhassan et al.	https://doi.org/10.1186/s12939-020-01204-5	No	Yes	No	No	Yes	No	No	No	No
Trends and correlates of maternal, newborn and child health services utilization in primary healthcare facilities: An explorative ecological study using DHIMSII data from one district in the Volta region of Ghana	2020	Alhassan et al.	https://doi.org/10.1186/s12884-020-03195-1	Yes	No	No	No	No	No	No	No	No

1. BIBLIOGRAPHIC SOURCE				4. UNIVERSAL HEALTH COVERA								
**Title**	**Year**	**First author**	**UHL**	**Antenatal care (ANC)**	**Delivery care: Skilled birth attendance**	**Cesarean delivery**	**Postnatal care (PNC)**	**Child immunization (vaccine)**	**Care seeking for suspected pneumonia**	**Tuberculosis (TB) treatment**	**HIV therapy (ART)**	**Water and sanitation (WASH)**
Determinants of equity in utilization of maternal health services in Butajira, Southern Ethiopia	2012	Aliy & Mariam	https://www.ajol.info/index.php/ejhd/article/view/116114	Yes	No	No	No	No	No	No	No	No
Determinants of insecticide treated nets use among youth corp members in Edo State, Nigeria	2011	Amoran O. E. et al	http://www.biomedcentral.com/1471-2458/11/728	No	No	No	No	No	No	No	No	No

1. BIBLIOGRAPHIC SOURCE				4. UNIVERSAL HEALTH COVERA								
**Title**	**Year**	**First author**	**UHL**	**Antenatal care (ANC)**	**Delivery care: Skilled birth attendance**	**Cesarean delivery**	**Postnatal care (PNC)**	**Child immunization (vaccine)**	**Care seeking for suspected pneumonia**	**Tuberculosis (TB) treatment**	**HIV therapy (ART)**	**Water and sanitation (WASH)**
Towards achievement of Sustainable Development Goal 3: multilevel analyses of demographic and health survey data on health insurance coverage and maternal healthcare utilisation in sub-Saharan Africa	2022	Amu et al.	https://doi.org/10.1093/inthealth/ihac017	Yes	Yes	No	No	No	No	No	No	No

1. BIBLIOGRAPHIC SOURCE				4. UNIVERSAL HEALTH COVERA								
**Title**	**Year**	**First author**	**UHL**	**Antenatal care (ANC)**	**Delivery care: Skilled birth attendance**	**Cesarean delivery**	**Postnatal care (PNC)**	**Child immunization (vaccine)**	**Care seeking for suspected pneumonia**	**Tuberculosis (TB) treatment**	**HIV therapy (ART)**	**Water and sanitation (WASH)**
Equity and access to maternal and child health services in Ghana a cross-sectional study	2021	Anarwat et al.	https://doi.org/10.1186/s12913-021-06872-9	No	Yes	No	No	Yes	Yes	No	No	No
A subnational profiling analysis reveals regional differences as the main predictor of ITN ownership and use in Nigeria	2019	Andrada et al.	https://doi.org/10.1186/s12936-019-2816-9	No	No	No	No	No	No	No	No	No
Inequality trends in maternal health services for young Ghanaian women with childbirth history between 2003 and 2014	2017	Asamoah & Agardh	https://doi.org/10.1136/bmjopen-2016-011663	Yes	Yes	No	No	No	No	No	No	No

1. BIBLIOGRAPHIC SOURCE				4. UNIVERSAL HEALTH COVERA								
**Title**	**Year**	**First author**	**UHL**	**Antenatal care (ANC)**	**Delivery care: Skilled birth attendance**	**Cesarean delivery**	**Postnatal care (PNC)**	**Child immunization (vaccine)**	**Care seeking for suspected pneumonia**	**Tuberculosis (TB) treatment**	**HIV therapy (ART)**	**Water and sanitation (WASH)**
Leaving no one behind: Lessons from implementation of policies for universal HIV treatment to universal health coverage	2020	Assefa et al.	https://doi.org/10.1186/s12992-020-00549-4	No	No	No	No	No	No	No	Yes	No
Inequalities in child immunization coverage in Ghana: evidence from a decomposition analysis	2018	Asuman et al.	https://doi.org/10.1186/s13561-018-0193-7	No	No	No	No	Yes	No	No	No	No
A reassessment of global antenatal care coverage for improving maternal health using sub-Saharan Africa as a case study	2018	Ataguba	https://dx.doi.org/10.1371/journal.pone.020482[illegible]	Yes	No	No	No	No	No	No	No	No

1. BIBLIOGRAPHIC SOURCE				4. UNIVERSAL HEALTH COVERA								
**Title**	**Year**	**First author**	**UHL**	**Antenatal care (ANC)**	**Delivery care: Skilled birth attendance**	**Cesarean delivery**	**Postnatal care (PNC)**	**Child immunization (vaccine)**	**Care seeking for suspected pneumonia**	**Tuberculosis (TB) treatment**	**HIV therapy (ART)**	**Water and sanitation (WASH)**
Socio-economic inequality in maternal health care utilization in Sub-Saharan Africa: Evidence from Togo	2021	Atake	http://dx.doi.org/10.1002/hpm.3083	Yes	No	No	No	No	No	No	No	No
Poor prenatal service utilization and pregnancy outcome in a tertiary health facility in southwest Nigeria	2020	Awoleke & Olofinbiyi	https://doi.org/10.11604/pamj.2020.35.28.20426	Yes	No	No	No	No	No	No	No	No
Correlates of intra-household ITN use in Liberia: A multilevel analysis of household survey data	2016	Babalola et al.	http://dx.doi.org/10.1371/journal.pone.0158331	No	No	No	No	No	No	No	No	No

1. BIBLIOGRAPHIC SOURCE				4. UNIVERSAL HEALTH COVERA								
Title	Year	First author	UHL	Antenatal care (ANC)	Delivery care: Skilled birth attendance	Cesarean delivery	Postnatal care (PNC)	Child immunization (vaccine)	Care seeking for suspected pneumonia	Tuberculosis (TB) treatment	HIV therapy (ART)	Water and sanitation (WASH)
Prevalence and determinants of maternal healthcare utilisation among young women in sub-Saharan Africa: cross-sectional analyses of demographic and health survey data	2022	Bain et al.	https://doi.org/10.1186/s12889-022-13037-8	Yes	Yes	No	No	No	No	No	No	No

1. BIBLIOGRAPHIC SOURCE				4. UNIVERSAL HEALTH COVERA								
**Title**	**Year**	**First author**	**UHL**	**Antenatal care (ANC)**	**Delivery care: Skilled birth attendance**	**Cesarean delivery**	**Postnatal care (PNC)**	**Child immunization (vaccine)**	**Care seeking for suspected pneumonia**	**Tuberculosis (TB) treatment**	**HIV therapy (ART)**	**Water and sanitation (WASH)**
Factors influencing timing and frequency of antenatal care in Uganda	2011	Bbaale	https://www.ncbi.nlm.nih.gov/pmc/articles/PMC3562883/pdf/AMJ-04-431.pdf	Yes	No	No	No	No	No	No	No	No
Maternal education and childbirth care in Uganda	2011	Bbaale & Guloba	https://www.ncbi.nlm.nih.gov/pmc/articles/PMC3562941/pdf/AMJ-04-389.pdf	No	Yes	No	No	No	No	No	No	No

1. BIBLIOGRAPHIC SOURCE				4. UNIVERSAL HEALTH COVERA								
**Title**	**Year**	**First author**	**UHL**	**Antenatal care (ANC)**	**Delivery care: Skilled birth attendance**	**Cesarean delivery**	**Postnatal care (PNC)**	**Child immunization (vaccine)**	**Care seeking for suspected pneumonia**	**Tuberculosis (TB) treatment**	**HIV therapy (ART)**	**Water and sanitation (WASH)**
A quantitative analysis of food insecurity and other barriers associated with ART nonadherence among women in rural communities of Eswatini	2021	Becker et al.	https://doi.org/10.1371/journal.pone.0256277	No	No	No	No	No	No	No	Yes	No
Two decades of antenatal and delivery care in Uganda: a cross-sectional study using Demographic and Health Surveys	2018	Benova et al.	https://doi.org/10.1186/s12913-018-3546-3	Yes	Yes	No	No	No	No	No	No	No
Utilization of sexual and reproductive health services in ethiopia - Does it affect sexual activity among high school students?	2015	Bilal et al.	https://doi.org/10.1016/j.srhc.2014.09.009	No	No	No	No	No	No	No	No	No

1. BIBLIOGRAPHIC SOURCE				4. UNIVERSAL HEALTH COVERA								
**Title**	**Year**	**First author**	**UHL**	**Antenatal care (ANC)**	**Delivery care: Skilled birth attendance**	**Cesarean delivery**	**Postnatal care (PNC)**	**Child immunization (vaccine)**	**Care seeking for suspected pneumonia**	**Tuberculosis (TB) treatment**	**HIV therapy (ART)**	**Water and sanitation (WASH)**
Addressing the huge poor–rich gap of inequalities in accessing safe childbirth care: A first step to achieving universal maternal health coverage in Tanzania	2021	Bintabara	https://doi.org/10.1371/journal.pone.0246995	No	Yes	No	No	No	No	No	No	No
Twelve-year persistence of inequalities in antenatal care utilisation among women in Tanzania: A decomposition analysis of population-based cross-sectional surveys	2021	Bintabara & Basinda	https://doi.org/10.1136/bmjopen-2020-040450	Yes	No	No	No	No	No	No	No	No
Socio-demographic and economic inequalities in modern contraception in 11 low- And middle-income countries: An analysis of the PMA2020 surveys	2020	Blumenberg et al.	https://doi.org/10.1186/s12978-020-00931-w	No	No	No	No	No	No	No	No	No

1. BIBLIOGRAPHIC SOURCE				4. UNIVERSAL HEALTH COVERA								
Title	Year	First author	UHL	Antenatal care (ANC)	Delivery care: Skilled birth attendance	Cesarean delivery	Postnatal care (PNC)	Child immunization (vaccine)	Care seeking for suspected pneumonia	Tuberculosis (TB) treatment	HIV therapy (ART)	Water and sanitation (WASH)
A decomposition analysis of change in skilled birth attendants, 2003 to 2008, Ghana demographic and health surveys	2014	Bosomprah et al.	https://doi.org/10.1186/s12884-014-0415-x	No	Yes	No	No	No	No	No	No	No
Slow and Steady can Still Win the Race': Childhood Vaccination Experience of Migrant Ebira Women Within the Health System in Ekiti State, Nigeria	2021	Olakanmi-Falade & Awoleke	https://www.ojhas.org/issue79/2021-3-3.html	No	No	No	No	Yes	No	No	No	No
Antiretroviral therapy in Walvis Bay, Namibia	2016	Callaghan	https://hdl.handle.net/1807/70825	No	No	No	No	No	No	No	Yes	No

1. BIBLIOGRAPHIC SOURCE				4. UNIVERSAL HEALTH COVERA								
**Title**	**Year**	**First author**	**UHL**	**Antenatal care (ANC)**	**Delivery care: Skilled birth attendance**	**Cesarean delivery**	**Postnatal care (PNC)**	**Child immunization (vaccine)**	**Care seeking for suspected pneumonia**	**Tuberculosis (TB) treatment**	**HIV therapy (ART)**	**Water and sanitation (WASH)**
Family planning, antenatal and delivery care: Cross-sectional survey evidence on levels of coverage and inequalities by public and private sector in 57 low- and middle-income countries	2016	Campbell et al.	https://doi.org/10.1111/tmi.12681	Yes	No	No	No	No	No	No	No	No

1. BIBLIOGRAPHIC SOURCE				4. UNIVERSAL HEALTH COVER/								
**Title**	**Year**	**First author**	**UHL**	**Antenatal care (ANC)**	**Delivery care: Skilled birth attendance**	**Cesarean delivery**	**Postnatal care (PNC)**	**Child immunization (vaccine)**	**Care seeking for suspected pneumonia**	**Tuberculosis (TB) treatment**	**HIV therapy (ART)**	**Water and sanitation (WASH)**
Use of family planning and child health services in the private sector: An equity analysis of 12 DHS surveys	2018	Chakraborty & Sprockett	https://doi.org/10.1186/s12939-018-0763-7	No	No	No	No	No	No	No	No	No
Meeting demand for family planning within a generation: prospects and implications at country level	2015	Choi et al.	https://dx.doi.org/10.3402/gha.v8.29734	No	No	No	No	No	No	No	No	No
A Longitudinal Analysis of Mosquito Net Ownership and Use in an Indigenous Batwa Population after a Targeted Distribution	2016	Clark et al.	https://doi.org/10.1371/journal.pone.0154808	No	No	No	No	No	No	No	No	No

1. BIBLIOGRAPHIC SOURCE				4. UNIVERSAL HEALTH COVERA								
Title	Year	First author	UHL	Antenatal care (ANC)	Delivery care: Skilled birth attendance	Cesarean delivery	Postnatal care (PNC)	Child immunization (vaccine)	Care seeking for suspected pneumonia	Tuberculosis (TB) treatment	HIV therapy (ART)	Water and sanitation (WASH)
Malaria prevalence and long-lasting insecticidal net use in rural western Uganda: results of a cross-sectional survey conducted in an area of highly variable malaria transmission intensity	2021	Cote et al.	https://doi.org/10.1186/s12936-021-03835-7	No	No	No	No	No	No	No	No	No
Women's Sexual Empowerment and Contraceptive Use in Ghana	2012	Crissman et al.	https://doi.org/10.1111/j.1728-4465.2012.00318.x	No	No	No	No	No	No	No	No	No
Socio-economic and demographic factors associated with reproductive and child health preventive care in Mozambique: a cross-sectional study	2020	Daca et al.	https://doi.org/10.1186/s12939-020-01303-3	No	No	No	No	Yes	No	No	No	No

1. BIBLIOGRAPHIC SOURCE				4. UNIVERSAL HEALTH COVERA								
**Title**	**Year**	**First author**	**UHL**	**Antenatal care (ANC)**	**Delivery care: Skilled birth attendance**	**Cesarean delivery**	**Postnatal care (PNC)**	**Child immunization (vaccine)**	**Care seeking for suspected pneumonia**	**Tuberculosis (TB) treatment**	**HIV therapy (ART)**	**Water and sanitation (WASH)**
Assessing the contextual effect of community in the utilization of postnatal care services in Ghana	2021	Dankwah et al.	https://doi.org/10.1186/s12913-020-06028-1	No	No	No	Yes	No	No	No	No	No
Is South Africa closing the health gaps between districts? Monitoring progress towards universal health service coverage with routine facility data	2021	Day et al.	https://doi.org/10.1186/s12913-021-06171-3	Yes	No	No	No	Yes	Yes	Yes	Yes	Yes
Neglected tropical diseases as a 'litmus test' for universal health coverage? understanding who is left behind and why in mass drug administration: Lessons from four country contexts	2019	Dean et al.	https://doi.org/10.1371/journal.pntd.0007847	No	No	No	No	No	No	No	No	No

1. BIBLIOGRAPHIC SOURCE				4. UNIVERSAL HEALTH COVERA								
Title	Year	First author	UHL	Antenatal care (ANC)	Delivery care: Skilled birth attendance	Cesarean delivery	Postnatal care (PNC)	Child immunization (vaccine)	Care seeking for suspected pneumonia	Tuberculosis (TB) treatment	HIV therapy (ART)	Water and sanitation (WASH)
Preventive Health Service Coverage Among Infants and Children at Six Maternal-Child Health Clinics in Western Kenya: A Cross-Sectional Assessment	2022	Deathe et al.	https://doi.org/10.1007/s10995-021-03271-8	No	No	No	No	Yes	No	No	No	No
Distance, difference in altitude and socioeconomic determinants of utilisation of maternal and child health services in Ethiopia: A geographic and multilevel modelling analysis	2021	Defar et al.	https://doi.org/10.1136/bmjopen-2020-042095	Yes	No	No	No	Yes	No	No	No	No
Geographic differences in maternal and child health care utilization in four Ethiopian regions; A cross-sectional study	2019	Defar et al.	https://doi.org/10.1186/s12939-019-1079-y	Yes	No	No	No	Yes	Yes	No	No	No

1. BIBLIOGRAPHIC SOURCE				4. UNIVERSAL HEALTH COVERA								
**Title**	**Year**	**First author**	**UHL**	**Antenatal care (ANC)**	**Delivery care: Skilled birth attendance**	**Cesarean delivery**	**Postnatal care (PNC)**	**Child immunization (vaccine)**	**Care seeking for suspected pneumonia**	**Tuberculosis (TB) treatment**	**HIV therapy (ART)**	**Water and sanitation (WASH)**
Ownership and use of insecticide-treated nets in Oromia and Amhara Regional States of Ethiopia twoyears after a nationwide campaign	2011	Deressa et al.	https://doi.org/10.1111/j.1365-3156.2011.02875.x	No	No	No	No	No	No	No	No	No
Effectiveness of post-campaign, door-to-door, hang-up, and communication interventions to increase long-lasting, insecticidal bed net utilization in Togo (2011-2012): A cluster randomized, control trial	2014	Desrochers et al.	https://doi.org/10.1186/1475-2875-13-260	No	No	No	No	No	No	No	No	No
Immediate postnatal care following childbirth in Ugandan health facilities: An analysis of Demographic and Health Surveys between 2001 and 2016	2021	Dey et al.	https://doi.org/10.1136/bmjgh-2020-004230	No	No	No	Yes	No	No	No	No	No

1. BIBLIOGRAPHIC SOURCE				4. UNIVERSAL HEALTH COVERA								
**Title**	**Year**	**First author**	**UHL**	**Antenatal care (ANC)**	**Delivery care: Skilled birth attendance**	**Cesarean delivery**	**Postnatal care (PNC)**	**Child immunization (vaccine)**	**Care seeking for suspected pneumonia**	**Tuberculosis (TB) treatment**	**HIV therapy (ART)**	**Water and sanitation (WASH)**
Inequalities in non-communicable diseases and effective responses	2013	Di Cesare et al.	https://doi.org/10.1016/s0140-6736(12)61851-0	No	No	No	No	No	No	No	No	No
Insecticide-treated nets ownership and utilization among under-five children following the 2010 mass distribution in Burkina Faso	2014	Diabate et al.	https://dx.doi.org/10.1186/1475-2875-13-353	No	No	No	No	No	No	No	No	No

1. BIBLIOGRAPHIC SOURCE				4. UNIVERSAL HEALTH COVERA								
**Title**	**Year**	**First author**	**UHL**	**Antenatal care (ANC)**	**Delivery care: Skilled birth attendance**	**Cesarean delivery**	**Postnatal care (PNC)**	**Child immunization (vaccine)**	**Care seeking for suspected pneumonia**	**Tuberculosis (TB) treatment**	**HIV therapy (ART)**	**Water and sanitation (WASH)**
Impact of mining projects on water and sanitation infrastructures and associated child health outcomes: a multi-country analysis of Demographic and Health Surveys (DHS) in sub-Saharan Africa	2021	Dietler et al.	https://doi.org/10.1186/s12992-021-00723-2	No	No	No	No	No	No	No	No	Yes

1. BIBLIOGRAPHIC SOURCE				4. UNIVERSAL HEALTH COVERA								
**Title**	**Year**	**First author**	**UHL**	**Antenatal care (ANC)**	**Delivery care: Skilled birth attendance**	**Cesarean delivery**	**Postnatal care (PNC)**	**Child immunization (vaccine)**	**Care seeking for suspected pneumonia**	**Tuberculosis (TB) treatment**	**HIV therapy (ART)**	**Water and sanitation (WASH)**
Predictors of insecticidal net use among internally displaced persons aged 6-59 months in Abuja, Nigeria	2018	Ejembi et al.	http://dx.doi.org/10.11604/pamj.2018.29.136.13322	No	No	No	No	No	No	No	No	No
Changes in Inequality in Use of Maternal Health Care Services: Evidence from Skilled Birth Attendance in Mauritania for the Period 2007-2015	2022	Taleb El Hassen et al.	https://doi.org/10.3390/ijerph19063566	No	Yes	No	No	No	No	No	No	No

1. BIBLIOGRAPHIC SOURCE				4. UNIVERSAL HEALTH COVERA								
Title	Year	First author	UHL	Antenatal care (ANC)	Delivery care: Skilled birth attendance	Cesarean delivery	Postnatal care (PNC)	Child immunization (vaccine)	Care seeking for suspected pneumonia	Tuberculosis (TB) treatment	HIV therapy (ART)	Water and sanitation (WASH)
Trend in the use of modern contraception in sub-Saharan Africa: Does women's education matter?	2014	Emina et al.	http://dx.doi.org/10.1016/j.contraception.2014.02.001	No	No	No	No	No	No	No	No	No

1. BIBLIOGRAPHIC SOURCE				4. UNIVERSAL HEALTH COVERA								
**Title**	**Year**	**First author**	**UHL**	**Antenatal care (ANC)**	**Delivery care: Skilled birth attendance**	**Cesarean delivery**	**Postnatal care (PNC)**	**Child immunization (vaccine)**	**Care seeking for suspected pneumonia**	**Tuberculosis (TB) treatment**	**HIV therapy (ART)**	**Water and sanitation (WASH)**
Utilization, Predictors and Gaps in the Continuum of Care for Maternal and Newborn Health in Ghana	2021	Enos et al.	https://dx.doi.org/10.21106/ijma.425	Yes	Yes	No	No	No	No	No	No	No
ART use and associated factors among HIV positive caregivers of orphans and vulnerable children in Tanzania	2020	Exavery et al.	https://doi.org/10.1186/s12889-020-09361-6	No	No	No	No	No	No	No	Yes	No
Trends and causes of socioeconomic inequalities in maternal healthcare in Ghana, 2003-2014	2019	Fenny et al.	https://doi.org/10.1108/ijse-03-2018-0148	Yes	No	No	No	No	No	No	No	No

1. BIBLIOGRAPHIC SOURCE				4. UNIVERSAL HEALTH COVERA								
**Title**	**Year**	**First author**	**UHL**	**Antenatal care (ANC)**	**Delivery care: Skilled birth attendance**	**Cesarean delivery**	**Postnatal care (PNC)**	**Child immunization (vaccine)**	**Care seeking for suspected pneumonia**	**Tuberculosis (TB) treatment**	**HIV therapy (ART)**	**Water and sanitation (WASH)**
Determination of the predictive factors of long-lasting insecticide-treated net ownership and utilisation in the Bamenda Health District of Cameroon	2017	Fokam et al.	https://doi.org/10.1186/s12889-017-4155-5	No	No	No	No	No	No	No	No	No
The free caesareans policy in low-income settings: An interrupted time series analysis in Mali (2003-2012)	2014	Fournier et al.	https://doi.org/10.1371/journal.pone.0105130	No	No	Yes	No	No	No	No	No	No

1. BIBLIOGRAPHIC SOURCE				4. UNIVERSAL HEALTH COVERA								
**Title**	**Year**	**First author**	**UHL**	**Antenatal care (ANC)**	**Delivery care: Skilled birth attendance**	**Cesarean delivery**	**Postnatal care (PNC)**	**Child immunization (vaccine)**	**Care seeking for suspected pneumonia**	**Tuberculosis (TB) treatment**	**HIV therapy (ART)**	**Water and sanitation (WASH)**
DO BETTER INSTITUTIONS BROADEN ACCESS TO SANITATION IN SUB-SAHARA AFRICA?	2021	Francois et al.	https://doi.org/10.1111/coep.12512	No	No	No	No	No	No	No	No	Yes

1. BIBLIOGRAPHIC SOURCE				4. UNIVERSAL HEALTH COVERA[illegible]								
**Title**	**Year**	**First author**	**UHL**	**Antenatal care (ANC)**	**Delivery care: Skilled birth attendance**	**Cesarean delivery**	**Postnatal care (PNC)**	**Child immunization (vaccine)**	**Care seeking for suspected pneumonia**	**Tuberculosis (TB) treatment**	**HIV therapy (ART)**	**Water and sanitation (WASH)**
Ethnic disparities in utilisation of maternal health care services in Ghana: evidence from the 2007 Ghana Maternal Health Survey	2016	Ganle	https://doi.org/10.1080/13557858.2015.1015499	Yes	Yes	No	No	No	No	No	No	No
Risky sexual behaviour and contraceptive use in contexts of displacement: Insights from a cross-sectional survey of female adolescent refugees in Ghana	2019	Ganle et al.	https://doi.org/10.1186/s12939-019-1031-1	No	No	No	No	No	No	No	No	No
Understanding how distance to facility and quality of care affect maternal health service utilization in Kenya and Haiti: A comparative geographic information system study	2019	Gao & Kelley	https://doi.org/10.4081/gh.2019.690	Yes	No	No	No	No	No	No	No	No

1. BIBLIOGRAPHIC SOURCE				4. UNIVERSAL HEALTH COVERA								
**Title**	**Year**	**First author**	**UHL**	**Antenatal care (ANC)**	**Delivery care: Skilled birth attendance**	**Cesarean delivery**	**Postnatal care (PNC)**	**Child immunization (vaccine)**	**Care seeking for suspected pneumonia**	**Tuberculosis (TB) treatment**	**HIV therapy (ART)**	**Water and sanitation (WASH)**
Gender differences in the use of insecticide-treated nets after a universal free distribution campaign in Kano State, Nigeria: Post-campaign survey results	2013	Garley et al.	https://doi.org/10.1186/1475-2875-12-119	No	No	No	No	No	No	No	No	No
Demand satisfied by modern contraceptive among married women of reproductive age in Kenya	2021	Gichangi et al.	https://doi.org/10.1371/journal.pone.0248393	No	No	No	No	No	No	No	No	No
LLIN Evaluation in Uganda Project (LLINEUP): Factors associated with ownership and use of long-lasting insecticidal nets in Uganda: A cross-sectional survey of 48 districts ISRCTN17516395 ISRCTN	2018	Gonahasa et al.	https://doi.org/10.1186/s12936-018-2571-3	No	No	No	No	No	No	No	No	No

1. BIBLIOGRAPHIC SOURCE				4. UNIVERSAL HEALTH COVERA								
**Title**	**Year**	**First author**	**UHL**	**Antenatal care (ANC)**	**Delivery care: Skilled birth attendance**	**Cesarean delivery**	**Postnatal care (PNC)**	**Child immunization (vaccine)**	**Care seeking for suspected pneumonia**	**Tuberculosis (TB) treatment**	**HIV therapy (ART)**	**Water and sanitation (WASH)**
ART adherence and viral suppression are high among most non-pregnant individuals with early-stage, asymptomatic HIV infection: an observational study from Uganda and South Africa	2019	Haberer et al.	https://doi.org/10.1002/jia2.25232	No	No	No	No	No	No	No	Yes	No
Low immunization coverage in Wonago district, southern Ethiopia: A community-based cross-sectional study	2019	Hailu et al.	https://doi.org/10.1371/journal.pone.0220144	No	No	No	No	Yes	No	No	No	No

1. BIBLIOGRAPHIC SOURCE				4. UNIVERSAL HEALTH COVERA								
**Title**	**Year**	**First author**	**UHL**	**Antenatal care (ANC)**	**Delivery care: Skilled birth attendance**	**Cesarean delivery**	**Postnatal care (PNC)**	**Child immunization (vaccine)**	**Care seeking for suspected pneumonia**	**Tuberculosis (TB) treatment**	**HIV therapy (ART)**	**Water and sanitation (WASH)**
Towards universal health coverage: The role of within-country wealth-related inequality in 28 countries in sub-Saharan Africa	2011	Hosseinpoor et al.	https://doi.org/10.2471/BLT.11.087536	Yes	Yes	No	No	No	Yes	No	No	No

1. BIBLIOGRAPHIC SOURCE				4. UNIVERSAL HEALTH COVERA								
**Title**	**Year**	**First author**	**UHL**	**Antenatal care (ANC)**	**Delivery care: Skilled birth attendance**	**Cesarean delivery**	**Postnatal care (PNC)**	**Child immunization (vaccine)**	**Care seeking for suspected pneumonia**	**Tuberculosis (TB) treatment**	**HIV therapy (ART)**	**Water and sanitation (WASH)**
Patterns and trends of contraceptive use among sexually active adolescents in Burkina Faso, Ethiopia, and Nigeria: evidence from cross-sectional studies	2015	Hounton et al.	https://doi.org/10.3402/gha.v8.29737	No	No	No	No	No	No	No	No	No
Towards universal health coverage for reproductive health services in Ethiopia: two policy recommendations	2015	Onarheim et al.	https://doi.org/10.1186/s12939-015-0218-3	Yes	Yes	No	No	No	No	No	No	No

1. BIBLIOGRAPHIC SOURCE				4. UNIVERSAL HEALTH COVERA								
**Title**	**Year**	**First author**	**UHL**	**Antenatal care (ANC)**	**Delivery care: Skilled birth attendance**	**Cesarean delivery**	**Postnatal care (PNC)**	**Child immunization (vaccine)**	**Care seeking for suspected pneumonia**	**Tuberculosis (TB) treatment**	**HIV therapy (ART)**	**Water and sanitation (WASH)**
Modern Contraception: Uptake and Correlates among Women of Reproductive Age-Group in a Rural Community of Osun State, Nigeria	2020	Idowu et al.	https://doi.org/10.4314/ejhs.v30i4.8	No	No	No	No	No	No	No	No	No
Determinants of geographical inequalities for DTP3 vaccine coverage in sub-Saharan Africa	2020	Ikilezi et al.	https://doi.org/10.1016/j.vaccine.2020.03.005	No	No	No	No	Yes	No	No	No	No
Influence of women's decision-making autonomy on antenatal care utilisation and institutional delivery services in Nigeria: evidence from the Nigeria Demographic and Health Survey 2018	2022	Imo	https://doi.org/10.1186/s12884-022-04478-5	Yes	No	No	No	No	No	No	No	No

1. BIBLIOGRAPHIC SOURCE				4. UNIVERSAL HEALTH COVERA								
**Title**	**Year**	**First author**	**UHL**	**Antenatal care (ANC)**	**Delivery care: Skilled birth attendance**	**Cesarean delivery**	**Postnatal care (PNC)**	**Child immunization (vaccine)**	**Care seeking for suspected pneumonia**	**Tuberculosis (TB) treatment**	**HIV therapy (ART)**	**Water and sanitation (WASH)**
Individual and community-level determinants of cervical cancer screening in Zimbabwe: a multi-level analyses of a nationwide survey	2022	Isabirye et al.	https://doi.org/10.1186/s12905-022-01881-0	No	No	No	No	No	No	No	No	No
Two decades of maternity care fee exemption policies in Ghana: have they benefited the poor?	2016	Johnson et al.	https://doi.org/10.1093/heapol/czv017	No	Yes	No	No	No	No	No	No	No

1. BIBLIOGRAPHIC SOURCE				4. UNIVERSAL HEALTH COVERA								
Title	Year	First author	UHL	Antenatal care (ANC)	Delivery care: Skilled birth attendance	Cesarean delivery	Postnatal care (PNC)	Child immunization (vaccine)	Care seeking for suspected pneumonia	Tuberculosis (TB) treatment	HIV therapy (ART)	Water and sanitation (WASH)
Socioeconomic inequalities in access to skilled birth attendance among urban and rural women in low-income and middle-income countries	2018	Joseph et al.	https://doi.org/10.1136/bmjgh-2018-000898	No	Yes	No	No	No	No	No	No	No

1. BIBLIOGRAPHIC SOURCE				4. UNIVERSAL HEALTH COVERA								
**Title**	**Year**	**First author**	**UHL**	**Antenatal care (ANC)**	**Delivery care: Skilled birth attendance**	**Cesarean delivery**	**Postnatal care (PNC)**	**Child immunization (vaccine)**	**Care seeking for suspected pneumonia**	**Tuberculosis (TB) treatment**	**HIV therapy (ART)**	**Water and sanitation (WASH)**
Investigating the disparities in cervical cancer screening among Namibian women	2015	Kangmennaang et al.	https://doi.org/10.1016/j.ygyno.2015.05.036	No	No	No	No	No	No	No	No	No
Contraceptive use and needs among adolescent women aged 15-19: Regional and global estimates and projections from 1990 to 2030 from a Bayesian hierarchical modelling study	2021	Kantorová et al.	https://doi.org/10.1371/journal.pone.0247479	No	No	No	No	No	No	No	No	No
Changes in equity of maternal, newborn, and child health care practices in 115 districts of rural Ethiopia: Implications for the health extension program	2015	Karim et al.	https://doi.org/10.1186/s12884-015-0668-z	No	Yes	No	Yes	Yes	No	No	No	No

1. BIBLIOGRAPHIC SOURCE				4. UNIVERSAL HEALTH COVERA[illegible]								
**Title**	**Year**	**First author**	**UHL**	**Antenatal care (ANC)**	**Delivery care: Skilled birth attendance**	**Cesarean delivery**	**Postnatal care (PNC)**	**Child immunization (vaccine)**	**Care seeking for suspected pneumonia**	**Tuberculosis (TB) treatment**	**HIV therapy (ART)**	**Water and sanitation (WASH)**
Contraceptive dynamics during COVID-19 in sub-Saharan Africa: Longitudinal evidence from Burkina Faso and Kenya	2021	Karp et al.	https://doi.org/10.1136/bmjsrh-2020-200944	No	No	No	No	No	No	No	No	No
Long-lasting insecticidal net source, ownership and use in the context of universal coverage: A household survey in eastern Rwanda	2015	Kateera et al.	https://doi.org/10.1186/s12936-015-0915-9	No	No	No	No	No	No	No	No	No
A quasi-experimental evaluation of an interpersonal communication intervention to increase insecticide-treated net use among children in Zambia	2012	Keating et al.	https://doi.org/10.1186/1475-2875-11-313	No	No	No	No	No	No	No	No	No

1. BIBLIOGRAPHIC SOURCE				4. UNIVERSAL HEALTH COVERA								
**Title**	**Year**	**First author**	**UHL**	**Antenatal care (ANC)**	**Delivery care: Skilled birth attendance**	**Cesarean delivery**	**Postnatal care (PNC)**	**Child immunization (vaccine)**	**Care seeking for suspected pneumonia**	**Tuberculosis (TB) treatment**	**HIV therapy (ART)**	**Water and sanitation (WASH)**
Assessment of Inequalities in Coverage of Essential Reproductive, Maternal, Newborn, Child, and Adolescent Health Interventions in Kenya	2018	Keats et al.	https://doi.org/10.1001/jamanetworkopen.2018.5152	Yes	Yes	No	No	Yes	Yes	No	No	No
Charting health system reconstruction in post-war Liberia: a comparison of rural vs. remote healthcare utilization	2016	Kentoffioet al.	https://doi.org/10.1186/s12913-016-1709-7	Yes	No	No	Yes	No	No	No	No	No

1. BIBLIOGRAPHIC SOURCE				4. UNIVERSAL HEALTH COVER[illegible]								
**Title**	**Year**	**First author**	**UHL**	**Antenatal care (ANC)**	**Delivery care: Skilled birth attendance**	**Cesarean delivery**	**Postnatal care (PNC)**	**Child immunization (vaccine)**	**Care seeking for suspected pneumonia**	**Tuberculosis (TB) treatment**	**HIV therapy (ART)**	**Water and sanitation (WASH)**
A Transparent Universal Health Coverage Index with Decomposition by Socioeconomic Groups: Application in Asian and African Settings	2019	Khan et al.	https://doi.org/10.1007/s40258-019-00464-9	No	No	No	No	No	No	No	No	No
Effect of user preferences on ITN use: a review of literature and data	2017	Koenker & Yukich	https://doi.org/10.1186/s12936-017-1879-8	No	No	No	No	No	No	No	No	No

1. BIBLIOGRAPHIC SOURCE				4. UNIVERSAL HEALTH COVERA								
**Title**	**Year**	**First author**	**UHL**	**Antenatal care (ANC)**	**Delivery care: Skilled birth attendance**	**Cesarean delivery**	**Postnatal care (PNC)**	**Child immunization (vaccine)**	**Care seeking for suspected pneumonia**	**Tuberculosis (TB) treatment**	**HIV therapy (ART)**	**Water and sanitation (WASH)**
Insecticide-treated net use before and after mass distribution in a fishing community along Lake Victoria, Kenya: successes and unavoidable pitfalls	2014	Larson et al.	http://www.malariajournal.com/content/13/1/466	No	No	No	No	No	No	No	No	No
Lifetime Prevalence of Cervical Cancer Screening in 55 Low- and Middle-Income Countries	2020	Lemp et al.	https://doi.org/10.1001/jama.2020.16244	No	No	No	No	No	No	No	No	No

1. BIBLIOGRAPHIC SOURCE				4. UNIVERSAL HEALTH COVERA[illegible]								
**Title**	**Year**	**First author**	**UHL**	**Antenatal care (ANC)**	**Delivery care: Skilled birth attendance**	**Cesarean delivery**	**Postnatal care (PNC)**	**Child immunization (vaccine)**	**Care seeking for suspected pneumonia**	**Tuberculosis (TB) treatment**	**HIV therapy (ART)**	**Water and sanitation (WASH)**
Financial accessibility and user fee reforms for maternal healthcare in five sub-Saharan countries: a quasi-experimental analysis	2016	Leone et al.	https://dx.doi.org/10.1136/bmjopen-2015-009692	No	No	Yes	No	No	No	No	No	No
Antiretroviral treatment coverage in a rural district in Tanzania--a modeling study using empirical data	2015	Levira et al.	https://doi.org/10.1186/s12889-015-1460-8	No	No	No	No	No	No	No	Yes	No
The extent of universal health coverage for maternal health services in eastern uganda: A cross sectional study	2021	Lindberg et al.	https://dx.doi.org/10.1007/s10995-021-03357-3	Yes	Yes	No	Yes	No	No	No	No	No

1. BIBLIOGRAPHIC SOURCE				4. UNIVERSAL HEALTH COVER[illegible]								
Title	Year	First author	UHL	Antenatal care (ANC)	Delivery care: Skilled birth attendance	Cesarean delivery	Postnatal care (PNC)	Child immunization (vaccine)	Care seeking for suspected pneumonia	Tuberculosis (TB) treatment	HIV therapy (ART)	Water and sanitation (WASH)
Freely distributed bed-net use among Chano Mille residents, south Ethiopia: A longitudinal study	2013	Loha et al.	https://doi.org/10.1186/1475-2875-12-23	No	No	No	No	No	No	No	No	No
Determinants of hanging and use of ITNs in the context of near universal coverage in Zambia	2012	Macintyre et al.	https://doi.org/10.1093/heapol/czr042	No	No	No	No	No	No	No	No	No

1. BIBLIOGRAPHIC SOURCE				4. UNIVERSAL HEALTH COVERA								
**Title**	**Year**	**First author**	**UHL**	**Antenatal care (ANC)**	**Delivery care: Skilled birth attendance**	**Cesarean delivery**	**Postnatal care (PNC)**	**Child immunization (vaccine)**	**Care seeking for suspected pneumonia**	**Tuberculosis (TB) treatment**	**HIV therapy (ART)**	**Water and sanitation (WASH)**
Determinants and Consequences of Failure of Linkage to Antiretroviral Therapy at Primary Care Level in Blantyre, Malawi: A Prospective Cohort Study	2012	MacPherson et al.	https://doi.org/10.1371/journal.pone.0044794	No	No	No	No	No	No	No	Yes	No
Disability and sexual and reproductive health service utilisation in Uganda: an intersectional analysis of demographic and health surveys between 2006 and 2016	2022	Mac-Seing et al.	https://doi.org/10.1186/s12889-022-12708-w	Yes	No	No	No	No	No	No	No	No

1. BIBLIOGRAPHIC SOURCE				4. UNIVERSAL HEALTH COVERA								
**Title**	**Year**	**First author**	**UHL**	**Antenatal care (ANC)**	**Delivery care: Skilled birth attendance**	**Cesarean delivery**	**Postnatal care (PNC)**	**Child immunization (vaccine)**	**Care seeking for suspected pneumonia**	**Tuberculosis (TB) treatment**	**HIV therapy (ART)**	**Water and sanitation (WASH)**
Insight into Nigeria's progress towards the universal coverage of reproductive, maternal, newborn and child health services: a secondary data analysis	2022	Mafiana et al.	http://dx.doi.org/10.1136/bmjopen-2022-061595	Yes	No	No	No	Yes	Yes	No	No	No
Implementation of Urban Health Equity Assessment and Response Tool: a Case of Matsapha, Swaziland	2018	Makadzange et al.	https://doi.org/10.1007/s11524-018-0241-y	No	Yes	No	No	Yes	No	No	No	Yes
Effects of door-to-door hang-up visits on the use of long-lasting insecticide-treated mosquito nets in the democratic republic of the congo: A cluster randomized controlled trial	2021	Mankadi and Jin	https://doi.org/10.3390/ijerph18179048	No	No	No	No	No	No	No	No	No

1. BIBLIOGRAPHIC SOURCE				4. UNIVERSAL HEALTH COVERA[illegible]								
**Title**	**Year**	**First author**	**UHL**	**Antenatal care (ANC)**	**Delivery care: Skilled birth attendance**	**Cesarean delivery**	**Postnatal care (PNC)**	**Child immunization (vaccine)**	**Care seeking for suspected pneumonia**	**Tuberculosis (TB) treatment**	**HIV therapy (ART)**	**Water and sanitation (WASH)**
Effect of bed net colour and shape preferences on bed net usage: a secondary data analysis of the 2017 Malawi Malaria Indicator Survey	2020	Mategula et al.	https://doi.org/10.1186/s12936-020-03499-9	No	No	No	No	No	No	No	No	No
HIV care coverage among HIV-positive adolescent girls and young women in South Africa: Results from the HERStory Study	2021	Mathews et al.	https://doi.org/10.7196/SAMJ.2021.v111i5.15351	No	No	No	No	No	No	No	Yes	No
Facilitators and barriers to retention in care under universal antiretroviral therapy (Option B+) for the Prevention of Mother to Child Transmission of HIV (PMTCT): A narrative review	2021	Mbeya Munkhondya et al.	https://doi.org/10.1016/j.ijans.2021.100372	No	No	No	No	No	No	No	Yes	No

1. BIBLIOGRAPHIC SOURCE				4. UNIVERSAL HEALTH COVERA								
Title	Year	First author	UHL	Antenatal care (ANC)	Delivery care: Skilled birth attendance	Cesarean delivery	Postnatal care (PNC)	Child immunization (vaccine)	Care seeking for suspected pneumonia	Tuberculosis (TB) treatment	HIV therapy (ART)	Water and sanitation (WASH)
The consequences of declining population access to insecticide-treated nets (ITNs) on net use patterns and physical degradation of nets after 22 months of ownership	2021	Mboma et al.	https://doi.org/10.1186/s12936-021-03686-2	No	No	No	No	No	No	No	No	No
Mosquito net coverage in years between mass distributions: a case study of Tanzania, 2013	2018	Mboma et al.	https://doi.org/10.1186/s12936-018-2247-z	No	No	No	No	No	No	No	No	No
Factors associated with contraceptive use in Tigray, North Ethiopia	2017	Medhanyie et al.	https://doi.org/10.1186/s12978-017-0281-x	No	No	No	No	No	No	No	No	No

1. BIBLIOGRAPHIC SOURCE				4. UNIVERSAL HEALTH COVERA[illegible]								
**Title**	**Year**	**First author**	**UHL**	**Antenatal care (ANC)**	**Delivery care: Skilled birth attendance**	**Cesarean delivery**	**Postnatal care (PNC)**	**Child immunization (vaccine)**	**Care seeking for suspected pneumonia**	**Tuberculosis (TB) treatment**	**HIV therapy (ART)**	**Water and sanitation (WASH)**
Adolescent sexual and reproductive health in sub-Saharan Africa: who is left behind?	2020	Melesse et al.	https://doi.org/10.1136/bmjgh-2019-002231	No	No	No	No	No	No	No	No	No
Sub-national levels and trends in contraceptive prevalence, unmet need, and demand for family planning in Nigeria with survey uncertainty	2019	Mercer et al.	https://doi.org/10.1186/s12889-019-8043-z	No	No	No	No	No	No	No	No	No

1. BIBLIOGRAPHIC SOURCE				4. UNIVERSAL HEALTH COVERA								
**Title**	**Year**	**First author**	**UHL**	**Antenatal care (ANC)**	**Delivery care: Skilled birth attendance**	**Cesarean delivery**	**Postnatal care (PNC)**	**Child immunization (vaccine)**	**Care seeking for suspected pneumonia**	**Tuberculosis (TB) treatment**	**HIV therapy (ART)**	**Water and sanitation (WASH)**
Exploring inequities in skilled care at birth among migrant population in a metropolitan city Addis Ababa, Ethiopia; A qualitative study	2014	Mirkuzie	http://www.equityhealthj.com/content/13/1/110	Yes	Yes	No	Yes	No	No	No	No	No
Factors associated with the use of mosquito bed nets: results from two cross-sectional household surveys in Zambezia Province, Mozambique	2016	Moon et al.	https://doi.org/10.1186/s12936-016-1250-5	No	No	No	No	No	No	No	No	No
Sociocultural and Institutional Constraints to Family Planning Uptake Among Migrant Female Head Porters in Madina, a Suburb of Accra, Ghana	2021	Munemo et al.	https://doi.org/10.1177/088610992095441 9	No	No	No	No	No	No	No	No	No

1. BIBLIOGRAPHIC SOURCE				4. UNIVERSAL HEALTH COVERA								
**Title**	**Year**	**First author**	**UHL**	**Antenatal care (ANC)**	**Delivery care: Skilled birth attendance**	**Cesarean delivery**	**Postnatal care (PNC)**	**Child immunization (vaccine)**	**Care seeking for suspected pneumonia**	**Tuberculosis (TB) treatment**	**HIV therapy (ART)**	**Water and sanitation (WASH)**
Reframing non-communicable diseases and injuries for equity in the era of universal health coverage: Findings and recommendations from the Kenya NCDI poverty commission	2021	Mwangi et al.	https://doi.org/10.5334/aogh.3085	No	No	No	No	No	No	No	No	No

1. BIBLIOGRAPHIC SOURCE				4. UNIVERSAL HEALTH COVERA								
**Title**	**Year**	**First author**	**UHL**	**Antenatal care (ANC)**	**Delivery care: Skilled birth attendance**	**Cesarean delivery**	**Postnatal care (PNC)**	**Child immunization (vaccine)**	**Care seeking for suspected pneumonia**	**Tuberculosis (TB) treatment**	**HIV therapy (ART)**	**Water and sanitation (WASH)**
Inequities and their determinants in coverage of maternal health services in Burkina Faso	2018	Mwase et al.	https://doi.org/10.1186/s12939-018-0770-8	Yes	No	No	Yes	No	No	No	No	No

1. BIBLIOGRAPHIC SOURCE				4. UNIVERSAL HEALTH COVERA								
**Title**	**Year**	**First author**	**UHL**	**Antenatal care (ANC)**	**Delivery care: Skilled birth attendance**	**Cesarean delivery**	**Postnatal care (PNC)**	**Child immunization (vaccine)**	**Care seeking for suspected pneumonia**	**Tuberculosis (TB) treatment**	**HIV therapy (ART)**	**Water and sanitation (WASH)**
A Cross-Sectional Study on Hypertension Medication Adherence in a High-Burden Region in Namibia: Exploring Hypertension Interventions and Validation of the Namibia Hill-Bone Compliance Scale	2022	Nakwafila et al.	https://doi.org/10.3390/ijerph19074416	No	No	No	No	No	No	No	No	No
Assessing Adherence to Antihypertensive Therapy in Primary Health Care in Namibia: Findings and Implications	2017	Nashilongo et al.	https://doi.org/10.1007/s10557-017-6756-8	No	No	No	No	No	No	No	No	No

1. BIBLIOGRAPHIC SOURCE				4. UNIVERSAL HEALTH COVER								
**Title**	**Year**	**First author**	**UHL**	**Antenatal care (ANC)**	**Delivery care: Skilled birth attendance**	**Cesarean delivery**	**Postnatal care (PNC)**	**Child immunization (vaccine)**	**Care seeking for suspected pneumonia**	**Tuberculosis (TB) treatment**	**HIV therapy (ART)**	**Water and sanitation (WASH)**
Incomplete vaccination and associated factors among children aged 12–23 months in South Africa: an analysis of the South African demographic and health survey 2016	2021	Ndwandwe et al.	https://doi.org/10.1080/21645515.2020.1791509	No	No	No	No	Yes	No	No	No	No
Determining the effective coverage of maternal and child health services in Kenya, using demographic and health survey data sets: tracking progress towards universal health coverage	2017	Nguhiu et al.	https://doi.org/10.1111/tmi.12841	Yes	Yes	No	No	Yes	No	No	No	No

1. BIBLIOGRAPHIC SOURCE				4. UNIVERSAL HEALTH COVERA								
**Title**	**Year**	**First author**	**UHL**	**Antenatal care (ANC)**	**Delivery care: Skilled birth attendance**	**Cesarean delivery**	**Postnatal care (PNC)**	**Child immunization (vaccine)**	**Care seeking for suspected pneumonia**	**Tuberculosis (TB) treatment**	**HIV therapy (ART)**	**Water and sanitation (WASH)**
Coverage and usage of insecticide treated nets (ITNs) within households: associated factors and effect on the prevalance of malaria parasitemia in the Mount Cameroon area	2019	Njumkeng et al.	https://doi.org/10.1186/s12889-019-7555-x	No	No	No	No	No	No	No	No	No
Socioeconomic inequalities in maternal health care utilization in Ghana	2019	Novignon et al.	https://doi.org/10.1186/s12939-019-1043-x	Yes	Yes	No	No	No	No	No	No	No

1. BIBLIOGRAPHIC SOURCE				4. UNIVERSAL HEALTH COVERA								
**Title**	**Year**	**First author**	**UHL**	**Antenatal care (ANC)**	**Delivery care: Skilled birth attendance**	**Cesarean delivery**	**Postnatal care (PNC)**	**Child immunization (vaccine)**	**Care seeking for suspected pneumonia**	**Tuberculosis (TB) treatment**	**HIV therapy (ART)**	**Water and sanitation (WASH)**
Why rural women do not use primary health centres for pregnancy care: Evidence from a qualitative study in Nigeria	2019	Ntoimo et al.	https://doi.org/10.1186/s12884-019-2433-1	Yes	No	No	No	No	No	No	No	No
Long-lasting insecticidal net (LLIN) ownership, use and cost of implementation after a mass distribution campaign in Kasaï Occidental Province, Democratic Republic of Congo	2017	Ntuku et al.	https://doi.org/10.1186/s12936-016-1671-1	No	No	No	No	No	No	No	No	No
Explaining socioeconomic disparities and gaps in the use of antenatal care services in 36 countries in sub-Saharan Africa	2021	Obse & Ataguba	https://doi.org/10.1093/heapol/czab036	Yes	No	No	No	No	No	No	No	No

1. BIBLIOGRAPHIC SOURCE				4. UNIVERSAL HEALTH COVERA								
**Title**	**Year**	**First author**	**UHL**	**Antenatal care (ANC)**	**Delivery care: Skilled birth attendance**	**Cesarean delivery**	**Postnatal care (PNC)**	**Child immunization (vaccine)**	**Care seeking for suspected pneumonia**	**Tuberculosis (TB) treatment**	**HIV therapy (ART)**	**Water and sanitation (WASH)**
Sociodemographic factors associated with the use of insecticide treated nets among under-fives in Nigeria: Evidence from a national survey	2022	Ojo et al.	https://doi.org/10.1177/00494755221110374	No	No	No	No	No	No	No	No	No
Towards making efficient use of household resources for appropriate prevention of malaria: investigating households' ownership, use and expenditures on ITNs and other preventive tools in Southeast Nigeria	2014	Onwujekwe et al.	http://www.biomedcentral.com/1471-2458/14/315	No	No	No	No	No	No	No	No	No

1. BIBLIOGRAPHIC SOURCE				4. UNIVERSAL HEALTH COVERA								
**Title**	**Year**	**First author**	**UHL**	**Antenatal care (ANC)**	**Delivery care: Skilled birth attendance**	**Cesarean delivery**	**Postnatal care (PNC)**	**Child immunization (vaccine)**	**Care seeking for suspected pneumonia**	**Tuberculosis (TB) treatment**	**HIV therapy (ART)**	**Water and sanitation (WASH)**
Demographic disparities in unimproved drinking water and sanitation in Ghana: A nationally representative cross-sectional study	2022	Oppong et al.	http://dx.doi.org/10.1136/bmjopen-2021-060595	No	No	No	No	No	No	No	No	Yes
Inequities in Access to Maternal Health Care in Enugu State: Implications for Universal Health Coverage to Meet Vision 2030 in Nigeria	2019	Ozumba et al.	https://doi.org/10.1177/0272684X18819977	Yes	No	No	No	No	No	No	No	No

1. BIBLIOGRAPHIC SOURCE				4. UNIVERSAL HEALTH COVERA								
**Title**	**Year**	**First author**	**UHL**	**Antenatal care (ANC)**	**Delivery care: Skilled birth attendance**	**Cesarean delivery**	**Postnatal care (PNC)**	**Child immunization (vaccine)**	**Care seeking for suspected pneumonia**	**Tuberculosis (TB) treatment**	**HIV therapy (ART)**	**Water and sanitation (WASH)**
How do supply- and demand-side interventions influence equity in healthcare utilisation? Evidence from maternal healthcare in Senegal	2019	Parmar & Banerjee	https://doi.org/10.1016/j.socscimed.2019.112582	No	Yes	No	No	No	No	No	No	No
Temporal and regional variations in use, equity and quality of antenatal care in Egypt: A repeat cross-sectional analysis using Demographic and Health Surveys	2019	Pugliese-Garcia et al.	https://doi.org/10.1186/s12884-019-2409-1	Yes	No	No	No	No	No	No	No	No

1. BIBLIOGRAPHIC SOURCE				4. UNIVERSAL HEALTH COVERA								
**Title**	**Year**	**First author**	**UHL**	**Antenatal care (ANC)**	**Delivery care: Skilled birth attendance**	**Cesarean delivery**	**Postnatal care (PNC)**	**Child immunization (vaccine)**	**Care seeking for suspected pneumonia**	**Tuberculosis (TB) treatment**	**HIV therapy (ART)**	**Water and sanitation (WASH)**
Geographical Inequalities in Use of Improved Drinking Water Supply and Sanitation across Sub-Saharan Africa: Mapping and Spatial Analysis of Cross-sectional Survey Data	2014	Pullan et al.	https://doi.org/10.1371/journal.pmed.1001626	No	No	No	No	No	No	No	No	Yes
Individual and Network Factors Associated With HIV Care Continuum Outcomes Among Nigerian MSM Accessing Health Care Services	2018	Ramadhani et al.	https://dx.doi.org/10.1097/QAI.000000000000175[illegible]	No	No	No	No	No	No	No	Yes	No
Non-adherence to long-lasting insecticide treated bednet use following successful malaria control in Tororo, Uganda	2020	Rek et al.	https://doi.org/10.1371/journal.pone.0243303	No	No	No	No	No	No	No	No	No

1. BIBLIOGRAPHIC SOURCE				4. UNIVERSAL HEALTH COVERA								
**Title**	**Year**	**First author**	**UHL**	**Antenatal care (ANC)**	**Delivery care: Skilled birth attendance**	**Cesarean delivery**	**Postnatal care (PNC)**	**Child immunization (vaccine)**	**Care seeking for suspected pneumonia**	**Tuberculosis (TB) treatment**	**HIV therapy (ART)**	**Water and sanitation (WASH)**
Design, implementation and evaluation of a national campaign to deliver 18 million free long-lasting insecticidal nets to uncovered sleeping spaces in Tanzania	2013	Renggli et al.	https://doi.org/10.1186/1475-2875-12-85	No	No	No	No	No	No	No	No	No
A long way to go - Estimates of combined water, sanitation and hygiene coverage for 25 sub-Saharan African countries	2017	Roche et al.	https://doi.org/10.1371/journal.pone.0171783	No	No	No	No	No	No	No	No	Yes

1. BIBLIOGRAPHIC SOURCE				4. UNIVERSAL HEALTH COVERA								
**Title**	**Year**	**First author**	**UHL**	**Antenatal care (ANC)**	**Delivery care: Skilled birth attendance**	**Cesarean delivery**	**Postnatal care (PNC)**	**Child immunization (vaccine)**	**Care seeking for suspected pneumonia**	**Tuberculosis (TB) treatment**	**HIV therapy (ART)**	**Water and sanitation (WASH)**
Determinants of bed net use in southeast Nigeria following mass distribution of LLINs: Implications for social behavior change interventions	2015	Russell et al.	https://doi.org/10.1371/journal.pone.0139447	No	No	No	No	No	No	No	No	No
Factors associated with the non-use of insecticide-treated nets in Rwandan children	2016	Ruyange et al.	https://doi.org/10.1186/s12936-016-1403-6	No	No	No	No	No	No	No	No	No

1. BIBLIOGRAPHIC SOURCE				4. UNIVERSAL HEALTH COVERA								
**Title**	**Year**	**First author**	**UHL**	**Antenatal care (ANC)**	**Delivery care: Skilled birth attendance**	**Cesarean delivery**	**Postnatal care (PNC)**	**Child immunization (vaccine)**	**Care seeking for suspected pneumonia**	**Tuberculosis (TB) treatment**	**HIV therapy (ART)**	**Water and sanitation (WASH)**
Wealth Status, Health Insurance, and Maternal Health Care Utilization in Africa: Evidence from Gabon	2020	Sanogo & Yaya	https://dx.doi.org/10.1155/2020/4036830	Yes	No	No	Yes	No	No	No	No	No

1. BIBLIOGRAPHIC SOURCE				4. UNIVERSAL HEALTH COVERA								
**Title**	**Year**	**First author**	**UHL**	**Antenatal care (ANC)**	**Delivery care: Skilled birth attendance**	**Cesarean delivery**	**Postnatal care (PNC)**	**Child immunization (vaccine)**	**Care seeking for suspected pneumonia**	**Tuberculosis (TB) treatment**	**HIV therapy (ART)**	**Water and sanitation (WASH)**
Factors associated with use of insecticide-treated net for malaria prevention in Manica District, Mozambique: a community-based cross-sectional survey	2021	Scott et al.	https://doi.org/10.1186/s12936-021-03738-7	No	No	No	No	No	No	No	No	No
Healthcare utilisation, cancer screening and potential barriers to accessing cancer care in rural South West Nigeria: a cross-sectional study	2021	Sharma et al.	https://doi.org/10.1136/bmjopen-2020-04035 2	No	No	No	No	No	No	No	No	No

1. BIBLIOGRAPHIC SOURCE				4. UNIVERSAL HEALTH COVERA								
**Title**	**Year**	**First author**	**UHL**	**Antenatal care (ANC)**	**Delivery care: Skilled birth attendance**	**Cesarean delivery**	**Postnatal care (PNC)**	**Child immunization (vaccine)**	**Care seeking for suspected pneumonia**	**Tuberculosis (TB) treatment**	**HIV therapy (ART)**	**Water and sanitation (WASH)**
Socio-economic inequalities in ANC attendance among mothers who gave birth in the past 12 months in Debre Brehan town and surrounding rural areas, North East Ethiopia: A community-based survey	2019	Shibre & Mekonnen	https://doi.org/10.1186/s12978-019-0768-8	Yes	No	No	No	No	No	No	No	No

1. BIBLIOGRAPHIC SOURCE				4. UNIVERSAL HEALTH COVERA								
**Title**	**Year**	**First author**	**UHL**	**Antenatal care (ANC)**	**Delivery care: Skilled birth attendance**	**Cesarean delivery**	**Postnatal care (PNC)**	**Child immunization (vaccine)**	**Care seeking for suspected pneumonia**	**Tuberculosis (TB) treatment**	**HIV therapy (ART)**	**Water and sanitation (WASH)**
Individual and environmental characteristics associated with immunization of children in rural areas of Burkina Faso: A multi-level analysis	2007	Sia et al.	https://pubmed.ncbi.nlm.nih.gov/18299262/	No	No	No	No	Yes	No	No	No	No
Inequalities in access and utilization of maternal, newborn and child health services in sub-saharan africa: A special focus on urban settings	2021	Sidze et al.	https://dx.doi.org/10.1007/s10995-021-03250-z	Yes	Yes	No	Yes	Yes	No	No	No	No

1. BIBLIOGRAPHIC SOURCE				4. UNIVERSAL HEALTH COVERA								
**Title**	**Year**	**First author**	**UHL**	**Antenatal care (ANC)**	**Delivery care: Skilled birth attendance**	**Cesarean delivery**	**Postnatal care (PNC)**	**Child immunization (vaccine)**	**Care seeking for suspected pneumonia**	**Tuberculosis (TB) treatment**	**HIV therapy (ART)**	**Water and sanitation (WASH)**
Insecticide-treated bed net access and use among preschool children in Nouna District, Burkina Faso	2020	Sié et al.	https://dx.doi.org/10.1093/inthealth/ihaa003	No	No	No	No	No	No	No	No	No
Evidence of improving antiretroviral therapy treatment delays: an analysis of eight years of programmatic outcomes in Blantyre, Malawi	2013	Sloan et al.	http://www.biomedcentral.com/1471-2458/13/490	No	No	No	No	No	No	No	Yes	No
Low use of long-lasting insecticidal nets for malaria prevention in south-central Ethiopia: A community-based cohort study	2019	Solomon et al.	https://doi.org/10.1371/journal.pone.0210578	No	No	No	No	No	No	No	No	No

1. BIBLIOGRAPHIC SOURCE				4. UNIVERSAL HEALTH COVERA								
**Title**	**Year**	**First author**	**UHL**	**Antenatal care (ANC)**	**Delivery care: Skilled birth attendance**	**Cesarean delivery**	**Postnatal care (PNC)**	**Child immunization (vaccine)**	**Care seeking for suspected pneumonia**	**Tuberculosis (TB) treatment**	**HIV therapy (ART)**	**Water and sanitation (WASH)**
Empowerment and use of modern contraceptive methods among married women in Burkina Faso: a multilevel analysis	2021	Some et al.	https://dx.doi.org/10.1186/s12889-021-11541-x	No	No	No	No	No	No	No	No	No
Evaluation of the 2011 long-lasting, insecticide-treated net distribution for universal coverage in Togo	2013	Stevens et al.	http://www.malariajournal.com/content/12/1/162	No	No	No	No	No	No	No	No	No
On the way to universal coverage of maternal services in Iringa rural District in Tanzania. Who is yet to be reached?	2016	Straneo et al.	https://doi.org/10.4314/ahs.v16i2.10	Yes	No	No	No	No	No	No	No	No

1. BIBLIOGRAPHIC SOURCE				4. UNIVERSAL HEALTH COVERA								
**Title**	**Year**	**First author**	**UHL**	**Antenatal care (ANC)**	**Delivery care: Skilled birth attendance**	**Cesarean delivery**	**Postnatal care (PNC)**	**Child immunization (vaccine)**	**Care seeking for suspected pneumonia**	**Tuberculosis (TB) treatment**	**HIV therapy (ART)**	**Water and sanitation (WASH)**
Evaluation of long-lasting insecticidal net distribution through schools in Southern Tanzania	2022	Stuck et al.	https://doi.org/10.1093/heapol/czab140	No	No	No	No	No	No	No	No	No
Not all inequalities are equal: differences in coverage across the continuum of reproductive health services	2019	Sully et al.	https://dx.doi.org/10.1136/bmjgh-2019-001695	Yes	No	No	No	No	No	No	No	No

1. BIBLIOGRAPHIC SOURCE				4. UNIVERSAL HEALTH COVERA[illegible]								
**Title**	**Year**	**First author**	**UHL**	**Antenatal care (ANC)**	**Delivery care: Skilled birth attendance**	**Cesarean delivery**	**Postnatal care (PNC)**	**Child immunization (vaccine)**	**Care seeking for suspected pneumonia**	**Tuberculosis (TB) treatment**	**HIV therapy (ART)**	**Water and sanitation (WASH)**
Determinants of long-lasting insecticidal net ownership and utilization in malaria transmission regions: Evidence from Zimbabwe Demographic and Health Surveys	2019	Tapera	https://doi.org/10.1186/s12936-019-2912-x	No	No	No	No	No	No	No	No	No
Sociodemographic inequities in cervical cancer screening, treatment and care amongst women aged at least 25 years: evidence from surveys in Harare, Zimbabwe	2019	Tapera et al.	https://doi.org/10.1186/s12889-019-6749-6	No	No	No	No	No	No	No	No	No

1. BIBLIOGRAPHIC SOURCE				4. UNIVERSAL HEALTH COVERA								
**Title**	**Year**	**First author**	**UHL**	**Antenatal care (ANC)**	**Delivery care: Skilled birth attendance**	**Cesarean delivery**	**Postnatal care (PNC)**	**Child immunization (vaccine)**	**Care seeking for suspected pneumonia**	**Tuberculosis (TB) treatment**	**HIV therapy (ART)**	**Water and sanitation (WASH)**
Factors associated to bed net use in Cameroon: a retrospective study in Mfou health district in the Centre Region	2012	Tchinda et al.	http://www.panafrican-med-journal.com/content/article/12/112/full/	No	No	No	No	No	No	No	No	No
Cervical cancer screening uptake and correlates among HIV-infected women: a cross-sectional survey in Cote d'Ivoire, West Africa	2019	Tchounga et al.	https://dx.doi.org/10.1136/bmjopen-2019-029882	No	No	No	No	No	No	No	No	No

1. BIBLIOGRAPHIC SOURCE				4. UNIVERSAL HEALTH COVERA								
Title	Year	First author	UHL	Antenatal care (ANC)	Delivery care: Skilled birth attendance	Cesarean delivery	Postnatal care (PNC)	Child immunization (vaccine)	Care seeking for suspected pneumonia	Tuberculosis (TB) treatment	HIV therapy (ART)	Water and sanitation (WASH)
Duration and determinants of delayed tuberculosis diagnosis and treatment in high-burden countries: a mixed-methods systematic review and meta-analysis	2021	Teo et al.	https://doi.org/10.1186/s12931-021-01841-6	No	No	No	No	No	No	Yes	No	No
Skilled delivery inequality in Ethiopia: To what extent are the poorest and uneducated mothers benefiting?	2017	Tesfaye et al.	https://doi.org/10.1186/s12939-017-0579-x	No	Yes	No	No	No	No	No	No	No

1. BIBLIOGRAPHIC SOURCE				4. UNIVERSAL HEALTH COVERA[illegible]								
**Title**	**Year**	**First author**	**UHL**	**Antenatal care (ANC)**	**Delivery care: Skilled birth attendance**	**Cesarean delivery**	**Postnatal care (PNC)**	**Child immunization (vaccine)**	**Care seeking for suspected pneumonia**	**Tuberculosis (TB) treatment**	**HIV therapy (ART)**	**Water and sanitation (WASH)**
Investigating the association between pregnancy intention and insecticide-treated bed net (ITN) use: A cross-sectional study of pregnant women in Rwanda	2015	Thogarapalli et al.	https://doi.org/10.1007/s10389-015-0676-5	No	No	No	No	No	No	No	No	No
Success of Senegal's first nationwide distribution of long-lasting insecticide-treated nets to children under five - Contribution toward universal coverage	2011	Thwing et al.	http://www.malariajournal.com/content/10/1/86	No	No	No	No	No	No	No	No	No
Declines in Malaria Burden and all-cause child mortality following increases in control interventions in Senegal, 2005-2010	2017	Thwing et al.	https://doi.org/10.4269/ajtmh.16-0953	No	No	No	No	No	No	No	No	No

1. BIBLIOGRAPHIC SOURCE				4. UNIVERSAL HEALTH COVERA								
**Title**	**Year**	**First author**	**UHL**	**Antenatal care (ANC)**	**Delivery care: Skilled birth attendance**	**Cesarean delivery**	**Postnatal care (PNC)**	**Child immunization (vaccine)**	**Care seeking for suspected pneumonia**	**Tuberculosis (TB) treatment**	**HIV therapy (ART)**	**Water and sanitation (WASH)**
Individual-level and community-level determinants of cervical cancer screening among Kenyan women: a multilevel analysis of a Nationwide survey	2017	Tiruneh et al.	https://doi.org/10.1186/s12905-017-0469-9	No	No	No	No	No	No	No	No	No
Evaluation of the coverage and effective use rate of long-lasting insecticidal nets after nation-wide scale up of their distribution in Benin	2013	Tokpponnon et al.	https://doi.org/10.1186/1756-3305-6-265	No	No	No	No	No	No	No	No	No
Factors associated with the upsurge in the use of delivery care services in Sierra Leone	2020	Tsawe & Susuman	https://doi.org/10.1016/j.puhe.2019.11.002	No	Yes	No	No	No	No	No	No	No

1. BIBLIOGRAPHIC SOURCE				4. UNIVERSAL HEALTH COVERA								
**Title**	**Year**	**First author**	**UHL**	**Antenatal care (ANC)**	**Delivery care: Skilled birth attendance**	**Cesarean delivery**	**Postnatal care (PNC)**	**Child immunization (vaccine)**	**Care seeking for suspected pneumonia**	**Tuberculosis (TB) treatment**	**HIV therapy (ART)**	**Water and sanitation (WASH)**
Which family members use the best nets? An analysis of the condition of mosquito nets and their distribution within households in Tanzania	2010	Tsuang et al.	http://www.malariajournal.com/content/9/1/211	No	No	No	No	No	No	No	No	No
Utilization of insecticide treated nets among pregnant women in enugu, South Eastern Nigeria	2013	Ugwu et al.	https://pubmed.ncbi.nlm.nih.gov/23771448/	No	No	No	No	No	No	No	No	No

1. BIBLIOGRAPHIC SOURCE				4. UNIVERSAL HEALTH COVERA								
**Title**	**Year**	**First author**	**UHL**	**Antenatal care (ANC)**	**Delivery care: Skilled birth attendance**	**Cesarean delivery**	**Postnatal care (PNC)**	**Child immunization (vaccine)**	**Care seeking for suspected pneumonia**	**Tuberculosis (TB) treatment**	**HIV therapy (ART)**	**Water and sanitation (WASH)**
Finding the gap: Revealing local disparities in coverage of maternal, newborn and child health services in South Sudan using lot quality assurance sampling	2015	Valadez et al.	https://doi.org/10.1111/tmi.12613	Yes	No	No	No	Yes	No	No	No	No
Group Medical Visit and Microfinance Intervention for Patients With Diabetes or Hypertension in Kenya	2021	Vedanthan et al.	https://doi.org/10.1016/j.jacc.2021.03.002	No	No	No	No	No	No	No	No	No

1. BIBLIOGRAPHIC SOURCE				4. UNIVERSAL HEALTH COVERA								
**Title**	**Year**	**First author**	**UHL**	**Antenatal care (ANC)**	**Delivery care: Skilled birth attendance**	**Cesarean delivery**	**Postnatal care (PNC)**	**Child immunization (vaccine)**	**Care seeking for suspected pneumonia**	**Tuberculosis (TB) treatment**	**HIV therapy (ART)**	**Water and sanitation (WASH)**
Equity in Maternal Health in South Africa: Analysis of Health Service Access and Health Status in a National Household Survey	2013	Wabiri et al.	https://doi.org/10.1371/journal.pone.0073864	Yes	Yes	No	No	No	No	No	No	No
Persisting Regional Disparities in Modern Contraceptive Use and Unmet Need for Contraception among Nigerian Women	2019	Wang & Cao	https://doi.org/10.1155/2019/9103928	No	No	No	No	No	No	No	No	No

1. BIBLIOGRAPHIC SOURCE				4. UNIVERSAL HEALTH COVERA								
**Title**	**Year**	**First author**	**UHL**	**Antenatal care (ANC)**	**Delivery care: Skilled birth attendance**	**Cesarean delivery**	**Postnatal care (PNC)**	**Child immunization (vaccine)**	**Care seeking for suspected pneumonia**	**Tuberculosis (TB) treatment**	**HIV therapy (ART)**	**Water and sanitation (WASH)**
Effective coverage of facility delivery in Bangladesh, Haiti, Malawi, Nepal, Senegal, and Tanzania	2019	Wang et al.	https://doi.org/10.1371/journal.pone.0217853	No	Yes	No	No	No	No	No	No	No
Use of long-lasting insecticide-treated bed nets in a population with universal coverage following a mass distribution campaign in Uganda	2016	Wanzira et al.	https://doi.org/10.1186/s12936-016-1360-0	No	No	No	No	No	No	No	No	No

1. BIBLIOGRAPHIC SOURCE				4. UNIVERSAL HEALTH COVERA								
**Title**	**Year**	**First author**	**UHL**	**Antenatal care (ANC)**	**Delivery care: Skilled birth attendance**	**Cesarean delivery**	**Postnatal care (PNC)**	**Child immunization (vaccine)**	**Care seeking for suspected pneumonia**	**Tuberculosis (TB) treatment**	**HIV therapy (ART)**	**Water and sanitation (WASH)**
Long-lasting insecticide-treated bed net ownership and use among children under five years of age following a targeted distribution in central Uganda	2014	Wanzira et al.	https://doi.org/10.1186/1475-2875-13-185	No	No	No	No	No	No	No	No	No
Evaluation of a national universal coverage campaign of long-lasting insecticidal nets in a rural district in north-west Tanzania	2012	West et al.	https://doi.org/10.1186/1475-2875-11-273	No	No	No	No	No	No	No	No	No
The impact of renewing long-lasting insecticide-treated nets in the event of malaria resurgence: Lessons from 10 years of net use in dielmo, Senegal	2021	Wotodjo et al.	https://doi.org/10.4269/AJTMH.20-0127	No	No	No	No	No	No	No	No	No

1. BIBLIOGRAPHIC SOURCE				4. UNIVERSAL HEALTH COVERA								
**Title**	**Year**	**First author**	**UHL**	**Antenatal care (ANC)**	**Delivery care: Skilled birth attendance**	**Cesarean delivery**	**Postnatal care (PNC)**	**Child immunization (vaccine)**	**Care seeking for suspected pneumonia**	**Tuberculosis (TB) treatment**	**HIV therapy (ART)**	**Water and sanitation (WASH)**
Wealth and Education Inequities in Maternal and Child Health Services Utilization in Rural Ethiopia	2022	Wuneh et al.	https://doi.org/10.3390/ijerph19095421	Yes	Yes	No	No	Yes	No	No	No	No
Inequalities in maternal health care utilization in Benin: A population based cross-sectional study	2018	Yaya et al.	https://doi.org/10.1186/s12884-018-1846-6	Yes	Yes	No	Yes	No	No	No	No	No

1. BIBLIOGRAPHIC SOURCE				4. UNIVERSAL HEALTH COVERA								
**Title**	**Year**	**First author**	**UHL**	**Antenatal care (ANC)**	**Delivery care: Skilled birth attendance**	**Cesarean delivery**	**Postnatal care (PNC)**	**Child immunization (vaccine)**	**Care seeking for suspected pneumonia**	**Tuberculosis (TB) treatment**	**HIV therapy (ART)**	**Water and sanitation (WASH)**
Long-lasting insecticide-treated bed net ownership, utilization and associated factors among school-age children in Dara Mallo and Uba Debretsehay districts, Southern Ethiopia	2020	Zerdo et al.	https://dx.doi.org/10.1186/s12936-020-03437-9	No	No	No	No	No	No	No	No	No
Trends and projections of universal health coverage indicators in Ghana, 1995-2030: A national and subnational study	2019	Zhang et al.	https://doi.org/10.1371/journal.pone.0209126	Yes	Yes	No	Yes	Yes	Yes	No	No	Yes

1. BIBLIOGRAPHIC SOURCE				4. UNIVERSAL HEALTH COVERA								
**Title**	**Year**	**First author**	**UHL**	**Antenatal care (ANC)**	**Delivery care: Skilled birth attendance**	**Cesarean delivery**	**Postnatal care (PNC)**	**Child immunization (vaccine)**	**Care seeking for suspected pneumonia**	**Tuberculosis (TB) treatment**	**HIV therapy (ART)**	**Water and sanitation (WASH)**
Developing Malawi's Universal Health Coverage Index	2022	Mchenga et al.	https://doi.org/10.3389/frhs.2021.786186	Yes	Yes	No	Yes	Yes	Yes	No	No	Yes
Socioeconomic Factors Associated with Compliance with Mass Drug Administration for Lymphatic Filariasis Elimination in Kenya: Descriptive Study Results	2012	Njomo et al.	https://www.proquest.com/docview/11159117 78?pq-origsite=gscholar&fromopenview=true	No	No	No	No	No	No	No	No	No

1. BIBLIOGRAPHIC SOURCE				4. UNIVERSAL HEALTH COVERA								
**Title**	**Year**	**First author**	**UHL**	**Antenatal care (ANC)**	**Delivery care: Skilled birth attendance**	**Cesarean delivery**	**Postnatal care (PNC)**	**Child immunization (vaccine)**	**Care seeking for suspected pneumonia**	**Tuberculosis (TB) treatment**	**HIV therapy (ART)**	**Water and sanitation (WASH)**
Equality analysis of main health indicators among children under 5 years in Uganda	2019	Elduma	http://dx.doi.org/10.4314/ejhs.v29i2.8	No	No	No	No	Yes	No	No	No	No

1. BIBLIOGRAPHIC SOURCE				AGE							
**Title**	**Year**	**First author**	**UHL**	**Insecticide treated bed net (ITN)**	**Neglected tropical disease (NTD)**	**Prevention and treatment of raised**	**Prevention and treatment of raised**	**Cervical cancer screening**	**Tobacco (non-)smoking**	**Other UHC indicators (e.g., UHC index)**	**Other - Specify the names**
Reproductive Plans And Utilization of Contraceptives Among Women Living With HIV	2019	Adeleye et al.	https://dx.doi.org/10.21106/ijma.277	No	No	No	No	No	No	No	
Provision of immediate postpartum contraception to women living with HIV in the Eastern Cape, South Africa; a cross-sectional analysis	2020	Adeniyi et al.	https://doi.org/10.1186/s12978-020-01049-9	No	No	No	No	No	No	No	
Moving Up the Sanitation Ladder: A Study of the Coverage and Utilization of Improved Sanitation Facilities and Associated Factors Among Households in Southern Ethiopia	2022	Afework et al.	https://doi.org/10.1177/11786302221080825	No	No	No	No	No	No	No	

1. BIBLIOGRAPHIC SOURCE				AGE								
**Title**	**Year**	**First author**	**UHL**	**Insecticide treated bed net (ITN)**	**Neglected tropical disease (NTD)**	**Prevention and treatment of raised**	**Prevention and treatment of raised**	**Cervical cancer screening**	**Tobacco (non-)smoking**	**Other UHC indicators (e.g., UHC index)**	**Other - Specify the names**	[redacted]
Maternal health care services utilisation in the context of 'Abiye' (safe motherhood) programme in Ondo State, Nigeria	2020	Ajayi et al.	https://doi.org/10.1186/s12889-020-08512-z	No	No	No	No	No	No	No		
Slums, women and sanitary living in South-South Nigeria	2021	Akpabio et al.	https://doi.org/10.1007/s10901-020-09802-z	No	No	No	No	No	No	No		
Leaving no child behind: Decomposing socioeconomic inequalities in child health for india and south africa	2021	Alaba et al.	https://doi.org/10.3390/ijerph1813714	No	No	No	No	No	No	No		

1. BIBLIOGRAPHIC SOURCE				AGE							
**Title**	**Year**	**First author**	**UHL**	**Insecticide treated bed net (ITN)**	**Neglected tropical disease (NTD)**	**Prevention and treatment of raised**	**Prevention and treatment of raised**	**Cervical cancer screening**	**Tobacco (non-)smoking**	**Other UHC indicators (e.g., UHC index)**	**Other - Specify the names**
Access to skilled attendant at birth and the coverage of the third dose of diphtheria-tetanus-pertussis vaccine across 14 West African countries - An equity analysis	2020	Alhassan et al.	https://doi.org/10.1186/s12939-020-01204-5	No	No	No	No	No	No	No	
Trends and correlates of maternal, newborn and child health services utilization in primary healthcare facilities: An explorative ecological study using DHIMSII data from one district in the Volta region of [illegible]	2020	Alhassan et al.	https://doi.org/10.1186/s12884-020-03195-1	No	No	No	No	No	No	No	

1. BIBLIOGRAPHIC SOURCE				AGE							
**Title**	**Year**	**First author**	**UHL**	**Insecticide treated bed net (ITN)**	**Neglected tropical disease (NTD)**	**Prevention and treatment of raised**	**Prevention and treatment of raised**	**Cervical cancer screening**	**Tobacco (non-)smoking**	**Other UHC indicators (e.g., UHC index)**	**Other - Specify the names**
Determinants of equity in utilization of maternal health services in Butajira, Southern Ethiopia	2012	Aliy & Mariam	https://www.ajol.info/index.php/ejhd/article/view/116114	No	No	No	No	No	No	No	
Determinants of insecticide treated nets use among youth corp members in Edo State, Nigeria	2011	Amoran O. E. et al	http://www.biomedcentral.com/1471-2458/11/728	Yes	No	No	No	No	No	No	

1. BIBLIOGRAPHIC SOURCE				AGE							
**Title**	**Year**	**First author**	**UHL**	**Insecticide treated bed net (ITN)**	**Neglected tropical disease (NTD)**	**Prevention and treatment of raised**	**Prevention and treatment of raised**	**Cervical cancer screening**	**Tobacco (non-)smoking**	**Other UHC indicators (e.g., UHC index)**	**Other - Specify the names**
Towards achievement of Sustainable Development Goal 3: multilevel analyses of demographic and health survey data on health insurance coverage and maternal healthcare utilisation in sub-Saharan Africa	2022	Amu et al.	https://doi.org/10.1093/inthealth/ihac017	No	No	No	No	No	No	No	

1. BIBLIOGRAPHIC SOURCE				AGE							
**Title**	**Year**	**First author**	**UHL**	**Insecticide treated bed net (ITN)**	**Neglected tropical disease (NTD)**	**Prevention and treatment of raised**	**Prevention and treatment of raised**	**Cervical cancer screening**	**Tobacco (non-)smoking**	**Other UHC indicators (e.g., UHC index)**	**Other - Specify the names**
Equity and access to maternal and child health services in Ghana a cross-sectional study	2021	Anarwat et al.	https://doi.org/10.1186/s12913-021-06872-9	No	No	No	No	No	No	No	
A subnational profiling analysis reveals regional differences as the main predictor of ITN ownership and use in Nigeria	2019	Andrada et al.	https://doi.org/10.1186/s12936-019-2816-9	Yes	No	No	No	No	No	No	
Inequality trends in maternal health services for young Ghanaian women with childbirth history between 2003 and 2014	2017	Asamoah & Agardh	https://doi.org/10.1136/bmjopen-2016-011663	No	No	No	No	No	No	No	

1. BIBLIOGRAPHIC SOURCE				AGE							
Title	Year	First author	UHL	Insecticide treated bed net (ITN)	Neglected tropical disease (NTD)	Prevention and treatment of raised	Prevention and treatment of raised	Cervical cancer screening	Tobacco (non-)smoking	Other UHC indicators (e.g., UHC index)	Other - Specify the names
Leaving no one behind: Lessons from implementation of policies for universal HIV treatment to universal health coverage	2020	Assefa et al.	https://doi.org/10.1186/s12992-020-00549-4	No	No	No	No	No	No	No	
Inequalities in child immunization coverage in Ghana: evidence from a decomposition analysis	2018	Asuman et al.	https://doi.org/10.1186/s13561-018-0193-7	No	No	No	No	No	No	No	
A reassessment of global antenatal care coverage for improving maternal health using sub-Saharan Africa as a case study	2018	Ataguba	https://dx.doi.org/10.1371/journal.pone.0204822	No	No	No	No	No	No	No	

1. BIBLIOGRAPHIC SOURCE				AGE							
**Title**	**Year**	**First author**	**UHL**	**Insecticide treated bed net (ITN)**	**Neglected tropical disease (NTD)**	**Prevention and treatment of raised**	**Prevention and treatment of raised**	**Cervical cancer screening**	**Tobacco (non-)smoking**	**Other UHC indicators (e.g., UHC index)**	**Other - Specify the names**
Socio-economic inequality in maternal health care utilization in Sub-Saharan Africa: Evidence from Togo	2021	Atake	http://dx.doi.org/10.1002/hpm.3083	No	No	No	No	No	No	No	
Poor prenatal service utilization and pregnancy outcome in a tertiary health facility in southwest Nigeria	2020	Awoleke & Olofinbiyi	https://doi.org/10.11604/pamj.2020.35.28.20426	No	No	No	No	No	No	No	
Correlates of intra-household ITN use in Liberia: A multilevel analysis of household survey data	2016	Babalola et al.	http://dx.doi.org/10.1371/journal.pone.0158331	Yes	No	No	No	No	No	No	

1. BIBLIOGRAPHIC SOURCE				AGE								
Title	Year	First author	UHL	Insecticide treated bed net (ITN)	Neglected tropical disease (NTD)	Prevention and treatment of raised	Prevention and treatment of raised	Cervical cancer screening	Tobacco (non-)smoking	Other UHC indicators (e.g., UHC index)	Other - Specify the names	[redacted]
Prevalence and determinants of maternal healthcare utilisation among young women in sub-Saharan Africa: cross-sectional analyses of demographic and health survey data	2022	Bain et al.	https://doi.org/10.1186/s12889-022-13037-8	No	No	No	No	No	No	No		

1. BIBLIOGRAPHIC SOURCE				AGE							
**Title**	**Year**	**First author**	**UHL**	**Insecticide treated bed net (ITN)**	**Neglected tropical disease (NTD)**	**Prevention and treatment of raised**	**Prevention and treatment of raised**	**Cervical cancer screening**	**Tobacco (non-)smoking**	**Other UHC indicators (e.g., UHC index)**	**Other - Specify the names**
Factors influencing timing and frequency of antenatal care in Uganda	2011	Bbaale	https://www.ncbi.nlm.nih.gov/pmc/articles/PMC3562883/pdf/AMJ-04-431.pdf	No	No	No	No	No	No	No	
Maternal education and childbirth care in Uganda	2011	Bbaale & Guloba	https://www.ncbi.nlm.nih.gov/pmc/articles/PMC3562941/pdf/AMJ-04-389.pdf	No	No	No	No	No	No	No	

1. BIBLIOGRAPHIC SOURCE				AGE							
**Title**	**Year**	**First author**	**UHL**	**Insecticide treated bed net (ITN)**	**Neglected tropical disease (NTD)**	**Prevention and treatment of raised**	**Prevention and treatment of raised**	**Cervical cancer screening**	**Tobacco (non-)smoking**	**Other UHC indicators (e.g., UHC index)**	**Other - Specify the names**
A quantitative analysis of food insecurity and other barriers associated with ART nonadherence among women in rural communities of Eswatini	2021	Becker et al.	https://doi.org/10.1371/journal.pone.0256277	No	No	No	No	No	No	No	
Two decades of antenatal and delivery care in Uganda: a cross-sectional study using Demographic and Health Surveys	2018	Benova et al.	https://doi.org/10.1186/s12913-018-3546-3	No	No	No	No	No	No	No	
Utilization of sexual and reproductive health services in ethiopia - Does it affect sexual activity among high school students?	2015	Bilal et al.	https://doi.org/10.1016/j.srhc.2014.09.009	No	No	No	No	No	No	No	

1. BIBLIOGRAPHIC SOURCE				AGE							
Title	Year	First author	UHL	Insecticide treated bed net (ITN)	Neglected tropical disease (NTD)	Prevention and treatment of raised	Prevention and treatment of raised	Cervical cancer screening	Tobacco (non-)smoking	Other UHC indicators (e.g., UHC index)	Other - Specify the names
Addressing the huge poor–rich gap of inequalities in accessing safe childbirth care: A first step to achieving universal maternal health coverage in Tanzania	2021	Bintabara	https://doi.org/10.1371/journal.pone.0246995	No	No	No	No	No	No	No	
Twelve-year persistence of inequalities in antenatal care utilisation among women in Tanzania: A decomposition analysis of population-based cross-sectional surveys	2021	Bintabara & Basinda	https://doi.org/10.1136/bmjopen-2020-040450	No	No	No	No	No	No	No	
Socio-demographic and economic inequalities in modern contraception in 11 low- And middle-income countries: An analysis of the PMA2020 surveys	2020	Blumenberg et al.	https://doi.org/10.1186/s12978-020-00931-w	No	No	No	No	No	No	No	

1. BIBLIOGRAPHIC SOURCE				AGE							
Title	Year	First author	UHL	Insecticide treated bed net (ITN)	Neglected tropical disease (NTD)	Prevention and treatment of raised	Prevention and treatment of raised	Cervical cancer screening	Tobacco (non-)smoking	Other UHC indicators (e.g., UHC index)	Other - Specify the names
A decomposition analysis of change in skilled birth attendants, 2003 to 2008, Ghana demographic and health surveys	2014	Bosomprah et al.	https://doi.org/10.1186/s12884-014-0415-x	No	No	No	No	No	No	No	
Slow and Steady can Still Win the Race': Childhood Vaccination Experience of Migrant Ebira Women Within the Health System in Ekiti State, Nigeria	2021	Olakanmi-Falade & Awoleke	https://www.ojhas.org/issue79/2021-3-3.html	No	No	No	No	No	No	No	
Antiretroviral therapy in Walvis Bay, Namibia	2016	Callaghan	https://hdl.handle.net/1807/70825	No	No	No	No	No	No	No	

1. BIBLIOGRAPHIC SOURCE				AGE							
**Title**	**Year**	**First author**	**UHL**	**Insecticide treated bed net (ITN)**	**Neglected tropical disease (NTD)**	**Prevention and treatment of raised**	**Prevention and treatment of raised**	**Cervical cancer screening**	**Tobacco (non-)smoking**	**Other UHC indicators (e.g., UHC index)**	**Other - Specify the names**
Family planning, antenatal and delivery care: Cross-sectional survey evidence on levels of coverage and inequalities by public and private sector in 57 low- and middle-income countries	2016	Campbell et al.	https://doi.org/10.1111/tmi.12681	No	No	No	No	No	No	No	

1. BIBLIOGRAPHIC SOURCE				AGE							
**Title**	**Year**	**First author**	**UHL**	**Insecticide treated bed net (ITN)**	**Neglected tropical disease (NTD)**	**Prevention and treatment of raised**	**Prevention and treatment of raised**	**Cervical cancer screening**	**Tobacco (non-)smoking**	**Other UHC indicators (e.g., UHC index)**	**Other - Specify the names**
Use of family planning and child health services in the private sector: An equity analysis of 12 DHS surveys	2018	Chakraborty & Sprockett	https://doi.org/10.1186/s12939-018-0763-7	No	No	No	No	No	No	No	
Meeting demand for family planning within a generation: prospects and implications at country level	2015	Choi et al.	https://dx.doi.org/10.3402/gha.v8.29734	No	No	No	No	No	No	No	
A Longitudinal Analysis of Mosquito Net Ownership and Use in an Indigenous Batwa Population after a Targeted Distribution	2016	Clark et al.	https://doi.org/10.1371/journal.pone.0154808	Yes	No	No	No	No	No	No	

1. BIBLIOGRAPHIC SOURCE				AGE							
Title	Year	First author	UHL	Insecticide treated bed net (ITN)	Neglected tropical disease (NTD)	Prevention and treatment of raised	Prevention and treatment of raised	Cervical cancer screening	Tobacco (non-)smoking	Other UHC indicators (e.g., UHC index)	Other - Specify the names
Malaria prevalence and long-lasting insecticidal net use in rural western Uganda: results of a cross-sectional survey conducted in an area of highly variable malaria transmission intensity	2021	Cote et al.	https://doi.org/10.1186/s12936-021-03835-7	Yes	No	No	No	No	No	No	
Women's Sexual Empowerment and Contraceptive Use in Ghana	2012	Crissman et al.	https://doi.org/10.1111/j.1728-4465.2012.00318.x	No	No	No	No	No	No	No	
Socio-economic and demographic factors associated with reproductive and child health preventive care in Mozambique: a cross-sectional study	2020	Daca et al.	https://doi.org/10.1186/s12939-020-01303-3	Yes	No	No	No	No	No	No	

1. BIBLIOGRAPHIC SOURCE				AGE							
**Title**	**Year**	**First author**	**UHL**	**Insecticide treated bed net (ITN)**	**Neglected tropical disease (NTD)**	**Prevention and treatment of raised**	**Prevention and treatment of raised**	**Cervical cancer screening**	**Tobacco (non-)smoking**	**Other UHC indicators (e.g., UHC index)**	**Other - Specify the names**
Assessing the contextual effect of community in the utilization of postnatal care services in Ghana	2021	Dankwah et al.	https://doi.org/10.1186/s12913-020-06028-1	No	No	No	No	No	No	No	
Is South Africa closing the health gaps between districts? Monitoring progress towards universal health service coverage with routine facility data	2021	Day et al.	https://doi.org/10.1186/s12913-021-06171-3	No	No	Yes	Yes	Yes	Yes	No	
Neglected tropical diseases as a 'litmus test' for universal health coverage? understanding who is left behind and why in mass drug administration: Lessons from four country contexts	2019	Dean et al.	https://doi.org/10.1371/journal.pntd.0007847	No	Yes	No	No	No	No	No	

1. BIBLIOGRAPHIC SOURCE				AGE							
**Title**	**Year**	**First author**	**UHL**	**Insecticide treated bed net (ITN)**	**Neglected tropical disease (NTD)**	**Prevention and treatment of raised**	**Prevention and treatment of raised**	**Cervical cancer screening**	**Tobacco (non-)smoking**	**Other UHC indicators (e.g., UHC index)**	**Other - Specify the names**
Preventive Health Service Coverage Among Infants and Children at Six Maternal-Child Health Clinics in Western Kenya: A Cross-Sectional Assessment	2022	Deathe et al.	https://doi.org/10.1007/s10995-021-03271-8	No	No	No	No	No	No	No	
Distance, difference in altitude and socioeconomic determinants of utilisation of maternal and child health services in Ethiopia: A geographic and multilevel modelling analysis	2021	Defar et al.	https://doi.org/10.1136/bmjopen-2020-042095	No	No	No	No	No	No	No	
Geographic differences in maternal and child health care utilization in four Ethiopian regions; A cross-sectional study	2019	Defar et al.	https://doi.org/10.1186/s12939-019-1079-y	No	No	No	No	No	No	No	

1. BIBLIOGRAPHIC SOURCE				AGE							
**Title**	**Year**	**First author**	**UHL**	**Insecticide treated bed net (ITN)**	**Neglected tropical disease (NTD)**	**Prevention and treatment of raised**	**Prevention and treatment of raised**	**Cervical cancer screening**	**Tobacco (non-)smoking**	**Other UHC indicators (e.g., UHC index)**	**Other - Specify the names**
Ownership and use of insecticide-treated nets in Oromia and Amhara Regional States of Ethiopia twoyears after a nationwide campaign	2011	Deressa et al.	https://doi.org/10.1111/j.1365-3156.2011.02875.x	Yes	No	No	No	No	No	No	
Effectiveness of post-campaign, door-to-door, hang-up, and communication interventions to increase long-lasting, insecticidal bed net utilization in Togo (2011-2012): A cluster randomized, control trial	2014	Desrochers et al.	https://doi.org/10.1186/1475-2875-13-260	Yes	No	No	No	No	No	No	
Immediate postnatal care following childbirth in Ugandan health facilities: An analysis of Demographic and Health Surveys between 2001 and 2016	2021	Dey et al.	https://doi.org/10.1136/bmjgh-2020-004230	No	No	No	No	No	No	No	

1. BIBLIOGRAPHIC SOURCE				AGE								
**Title**	**Year**	**First author**	**UHL**	**Insecticide treated bed net (ITN)**	**Neglected tropical disease (NTD)**	**Prevention and treatment of raised**	**Prevention and treatment of raised**	**Cervical cancer screening**	**Tobacco (non-)smoking**	**Other UHC indicators (e.g., UHC index)**	**Other - Specify the names**	[redacted]
Inequalities in non-communicable diseases and effective responses	2013	Di Cesare et al.	https://doi.org/10.1016/s0140-6736(12)61851-0	No	No	Yes	Yes	No	Yes	No		
Insecticide-treated nets ownership and utilization among under-five children following the 2010 mass distribution in Burkina Faso	2014	Diabate et al.	https://dx.doi.org/10.1186/1475-2875-13-353	Yes	No	No	No	No	No	No		

1. BIBLIOGRAPHIC SOURCE				AGE							
**Title**	**Year**	**First author**	**UHL**	**Insecticide treated bed net (ITN)**	**Neglected tropical disease (NTD)**	**Prevention and treatment of raised**	**Prevention and treatment of raised**	**Cervical cancer screening**	**Tobacco (non-)smoking**	**Other UHC indicators (e.g., UHC index)**	**Other - Specify the names**
Impact of mining projects on water and sanitation infrastructures and associated child health outcomes: a multi-country analysis of Demographic and Health Surveys (DHS) in sub-Saharan Africa	2021	Dietler et al.	https://doi.org/10.1186/s12992-021-00723-2	No	No	No	No	No	No	No	

1. BIBLIOGRAPHIC SOURCE				AGE								
**Title**	**Year**	**First author**	**UHL**	**Insecticide treated bed net (ITN)**	**Neglected tropical disease (NTD)**	**Prevention and treatment of raised**	**Prevention and treatment of raised**	**Cervical cancer screening**	**Tobacco (non-)smoking**	**Other UHC indicators (e.g., UHC index)**	**Other - Specify the names**	[redacted]
Predictors of insecticidal net use among internally displaced persons aged 6-59 months in Abuja, Nigeria	2018	Ejembi et al.	http://dx.doi.org/10.11604/pamj.2018.29.136.13322	Yes	No	No	No	No	No	No		
Changes in Inequality in Use of Maternal Health Care Services: Evidence from Skilled Birth Attendance in Mauritania for the Period 2007-2015	2022	Taleb El Hassen et al.	https://doi.org/10.3390/ijerph19063566	No	No	No	No	No	No	No		

1. BIBLIOGRAPHIC SOURCE				AGE							
**Title**	**Year**	**First author**	**UHL**	**Insecticide treated bed net (ITN)**	**Neglected tropical disease (NTD)**	**Prevention and treatment of raised**	**Prevention and treatment of raised**	**Cervical cancer screening**	**Tobacco (non-)smoking**	**Other UHC indicators (e.g., UHC index)**	**Other - Specify the names**
Trend in the use of modern contraception in sub-Saharan Africa: Does women's education matter?	2014	Emina et al.	http://dx.doi.org/10.1016/j.contraception.2014.02.001	No	No	No	No	No	No	No	

1. BIBLIOGRAPHIC SOURCE				AGE								
**Title**	**Year**	**First author**	**UHL**	**Insecticide treated bed net (ITN)**	**Neglected tropical disease (NTD)**	**Prevention and treatment of raised**	**Prevention and treatment of raised**	**Cervical cancer screening**	**Tobacco (non-)smoking**	**Other UHC indicators (e.g., UHC index)**	**Other - Specify the names**	[redacted]
Utilization, Predictors and Gaps in the Continuum of Care for Maternal and Newborn Health in Ghana	2021	Enos et al.	https://dx.doi.org/10.21106/ijma.425	No	No	No	No	No	No	No		
ART use and associated factors among HIV positive caregivers of orphans and vulnerable children in Tanzania	2020	Exavery et al.	https://doi.org/10.1186/s12889-020-09361-6	No	No	No	No	No	No	No		
Trends and causes of socioeconomic inequalities in maternal healthcare in Ghana, 2003-2014	2019	Fenny et al.	https://doi.org/10.1108/ijse-03-2018-0148	No	No	No	No	No	No	No		

1. BIBLIOGRAPHIC SOURCE				AGE								
**Title**	**Year**	**First author**	**UHL**	**Insecticide treated bed net (ITN)**	**Neglected tropical disease (NTD)**	**Prevention and treatment of raised**	**Prevention and treatment of raised**	**Cervical cancer screening**	**Tobacco (non-)smoking**	**Other UHC indicators (e.g., UHC index)**	**Other - Specify the names**	[redacted]
Determination of the predictive factors of long-lasting insecticide-treated net ownership and utilisation in the Bamenda Health District of Cameroon	2017	Fokam et al.	https://doi.org/10.1186/s12889-017-4155-5	Yes	No	No	No	No	No	No		
The free caesareans policy in low-income settings: An interrupted time series analysis in Mali (2003-2012)	2014	Fournier et al.	https://doi.org/10.1371/journal.pone.0105130	No	No	No	No	No	No	No		

1. BIBLIOGRAPHIC SOURCE				AGE								
**Title**	**Year**	**First author**	**UHL**	**Insecticide treated bed net (ITN)**	**Neglected tropical disease (NTD)**	**Prevention and treatment of raised**	**Prevention and treatment of raised**	**Cervical cancer screening**	**Tobacco (non-)smoking**	**Other UHC indicators (e.g., UHC index)**	**Other - Specify the names**	[redacted]
DO BETTER INSTITUTIONS BROADEN ACCESS TO SANITATION IN SUB-SAHARA AFRICA?	2021	Francois et al.	https://doi.org/10.1111/coep.12512	No	No	No	No	No	No	No		

1. BIBLIOGRAPHIC SOURCE				AGE							
**Title**	**Year**	**First author**	**UHL**	**Insecticide treated bed net (ITN)**	**Neglected tropical disease (NTD)**	**Prevention and treatment of raised**	**Prevention and treatment of raised**	**Cervical cancer screening**	**Tobacco (non-)smoking**	**Other UHC indicators (e.g., UHC index)**	**Other - Specify the names**
Ethnic disparities in utilisation of maternal health care services in Ghana: evidence from the 2007 Ghana Maternal Health Survey	2016	Ganle	https://doi.org/10.1080/13557858.2015.1015499	No	No	No	No	No	No	No	
Risky sexual behaviour and contraceptive use in contexts of displacement: Insights from a cross-sectional survey of female adolescent refugees in Ghana	2019	Ganle et al.	https://doi.org/10.1186/s12939-019-1031-1	No	No	No	No	No	No	No	
Understanding how distance to facility and quality of care affect maternal health service utilization in Kenya and Haiti: A comparative geographic information system study	2019	Gao & Kelley	https://doi.org/10.4081/gh.2019.690	No	No	No	No	No	No	No	

1. BIBLIOGRAPHIC SOURCE				AGE							
**Title**	**Year**	**First author**	**UHL**	**Insecticide treated bed net (ITN)**	**Neglected tropical disease (NTD)**	**Prevention and treatment of raised**	**Prevention and treatment of raised**	**Cervical cancer screening**	**Tobacco (non-)smoking**	**Other UHC indicators (e.g., UHC index)**	**Other - Specify the names**
Gender differences in the use of insecticide-treated nets after a universal free distribution campaign in Kano State, Nigeria: Post-campaign survey results	2013	Garley et al.	https://doi.org/10.1186/1475-2875-12-119	Yes	No	No	No	No	No	No	
Demand satisfied by modern contraceptive among married women of reproductive age in Kenya	2021	Gichangi et al.	https://doi.org/10.1371/journal.pone.0248393	No	No	No	No	No	No	No	
LLIN Evaluation in Uganda Project (LLINEUP): Factors associated with ownership and use of long-lasting insecticidal nets in Uganda: A cross-sectional survey of 48 districts ISRCTN17516395 ISRCTN	2018	Gonahasa et al.	https://doi.org/10.1186/s12936-018-2571-3	Yes	No	No	No	No	No	No	

1. BIBLIOGRAPHIC SOURCE				AGE							
**Title**	**Year**	**First author**	**UHL**	**Insecticide treated bed net (ITN)**	**Neglected tropical disease (NTD)**	**Prevention and treatment of raised**	**Prevention and treatment of raised**	**Cervical cancer screening**	**Tobacco (non-)smoking**	**Other UHC indicators (e.g., UHC index)**	**Other - Specify the names**
ART adherence and viral suppression are high among most non-pregnant individuals with early-stage, asymptomatic HIV infection: an observational study from Uganda and South Africa	2019	Haberer et al.	https://doi.org/10.1002/jia2.25232	No	No	No	No	No	No	No	
Low immunization coverage in Wonago district, southern Ethiopia: A community-based cross-sectional study	2019	Hailu et al.	https://doi.org/10.1371/journal.pone.0220144	No	No	No	No	No	No	No	

1. BIBLIOGRAPHIC SOURCE				AGE							
**Title**	**Year**	**First author**	**UHL**	**Insecticide treated bed net (ITN)**	**Neglected tropical disease (NTD)**	**Prevention and treatment of raised**	**Prevention and treatment of raised**	**Cervical cancer screening**	**Tobacco (non-)smoking**	**Other UHC indicators (e.g., UHC index)**	**Other - Specify the names**
Towards universal health coverage: The role of within-country wealth-related inequality in 28 countries in sub-Saharan Africa	2011	Hosseinpoor et al.	https://doi.org/10.2471/BLT.11.087536	No	No	No	No	No	No	Yes	Skilled birth attendance; one or more antenatal care visits;measles immunization; receipt of a third dose of vaccine against diphtheria, per-tussis and tetanus (DPT3); treatment of acute respiratory infection in children under 5 years of age; and family planning.

1. BIBLIOGRAPHIC SOURCE				AGE							
**Title**	**Year**	**First author**	**UHL**	**Insecticide treated bed net (ITN)**	**Neglected tropical disease (NTD)**	**Prevention and treatment of raised**	**Prevention and treatment of raised**	**Cervical cancer screening**	**Tobacco (non-)smoking**	**Other UHC indicators (e.g., UHC index)**	**Other - Specify the names**
Patterns and trends of contraceptive use among sexually active adolescents in Burkina Faso, Ethiopia, and Nigeria: evidence from cross-sectional studies	2015	Hounton et al.	https://doi.org/10.3402/gha.v8.29737	No	No	No	No	No	No	No	
Towards universal health coverage for reproductive health services in Ethiopia: two policy recommendations	2015	Onarheim et al.	https://doi.org/10.1186/s12939-015-0218-3	No	No	No	No	No	No	No	

1. BIBLIOGRAPHIC SOURCE				AGE								
**Title**	**Year**	**First author**	**UHL**	**Insecticide treated bed net (ITN)**	**Neglected tropical disease (NTD)**	**Prevention and treatment of raised**	**Prevention and treatment of raised**	**Cervical cancer screening**	**Tobacco (non-)smoking**	**Other UHC indicators (e.g., UHC index)**	**Other - Specify the names**	[redacted]
Modern Contraception: Uptake and Correlates among Women of Reproductive Age-Group in a Rural Community of Osun State, Nigeria	2020	Idowu et al.	https://doi.org/10.4314/ejhs.v30i4.8	No	No	No	No	No	No	No		
Determinants of geographical inequalities for DTP3 vaccine coverage in sub-Saharan Africa	2020	Ikilezi et al.	https://doi.org/10.1016/j.vaccine.2020.03.005	No	No	No	No	No	No	No		
Influence of women's decision-making autonomy on antenatal care utilisation and institutional delivery services in Nigeria: evidence from the Nigeria Demographic and Health Survey 2018	2022	Imo	https://doi.org/10.1186/s12884-022-04478-5	No	No	No	No	No	No	No		

1. BIBLIOGRAPHIC SOURCE				AGE								[redacted]
**Title**	**Year**	**First author**	**UHL**	**Insecticide treated bed net (ITN)**	**Neglected tropical disease (NTD)**	**Prevention and treatment of raised**	**Prevention and treatment of raised**	**Cervical cancer screening**	**Tobacco (non-)smoking**	**Other UHC indicators (e.g., UHC index)**	**Other - Specify the names**	
Individual and community-level determinants of cervical cancer screening in Zimbabwe: a multi-level analyses of a nationwide survey	2022	Isabirye et al.	https://doi.org/10.1186/s12905-022-01881-0	No	No	No	No	Yes	No	No		
Two decades of maternity care fee exemption policies in Ghana: have they benefited the poor?	2016	Johnson et al.	https://doi.org/10.1093/heapol/czv017	No	No	No	No	No	No	No		

1. BIBLIOGRAPHIC SOURCE				AGE							
**Title**	**Year**	**First author**	**UHL**	**Insecticide treated bed net (ITN)**	**Neglected tropical disease (NTD)**	**Prevention and treatment of raised**	**Prevention and treatment of raised**	**Cervical cancer screening**	**Tobacco (non-)smoking**	**Other UHC indicators (e.g., UHC index)**	**Other - Specify the names**
Socioeconomic inequalities in access to skilled birth attendance among urban and rural women in low-income and middle-income countries	2018	Joseph et al.	https://doi.org/10.1136/bmjgh-2018-000898	No	No	No	No	No	No	No	

1. BIBLIOGRAPHIC SOURCE				AGE							
**Title**	**Year**	**First author**	**UHL**	**Insecticide treated bed net (ITN)**	**Neglected tropical disease (NTD)**	**Prevention and treatment of raised**	**Prevention and treatment of raised**	**Cervical cancer screening**	**Tobacco (non-)smoking**	**Other UHC indicators (e.g., UHC index)**	**Other - Specify the names**
Investigating the disparities in cervical cancer screening among Namibian women	2015	Kangmennaang et al.	https://doi.org/10.1016/j.ygyno.2015.05.036	No	No	No	No	Yes	No	No	
Contraceptive use and needs among adolescent women aged 15-19: Regional and global estimates and projections from 1990 to 2030 from a Bayesian hierarchical modelling study	2021	Kantorová et al.	https://doi.org/10.1371/journal.pone.0247479	No	No	No	No	No	No	No	
Changes in equity of maternal, newborn, and child health care practices in 115 districts of rural Ethiopia: Implications for the health extension program	2015	Karim et al.	https://doi.org/10.1186/s12884-015-0668-z	No	No	No	No	No	No	No	

1. BIBLIOGRAPHIC SOURCE				AGE							
**Title**	**Year**	**First author**	**UHL**	**Insecticide treated bed net (ITN)**	**Neglected tropical disease (NTD)**	**Prevention and treatment of raised**	**Prevention and treatment of raised**	**Cervical cancer screening**	**Tobacco (non-)smoking**	**Other UHC indicators (e.g., UHC index)**	**Other - Specify the names**
Contraceptive dynamics during COVID-19 in sub-Saharan Africa: Longitudinal evidence from Burkina Faso and Kenya	2021	Karp et al.	https://doi.org/10.1136/bmjsrh-2020-200944	No	No	No	No	No	No	No	
Long-lasting insecticidal net source, ownership and use in the context of universal coverage: A household survey in eastern Rwanda	2015	Kateera et al.	https://doi.org/10.1186/s12936-015-0915-9	Yes	No	No	No	No	No	No	
A quasi-experimental evaluation of an interpersonal communication intervention to increase insecticide-treated net use among children in Zambia	2012	Keating et al.	https://doi.org/10.1186/1475-2875-11-313	Yes	No	No	No	No	No	No	

1. BIBLIOGRAPHIC SOURCE				AGE							
**Title**	**Year**	**First author**	**UHL**	**Insecticide treated bed net (ITN)**	**Neglected tropical disease (NTD)**	**Prevention and treatment of raised**	**Prevention and treatment of raised**	**Cervical cancer screening**	**Tobacco (non-)smoking**	**Other UHC indicators (e.g., UHC index)**	**Other - Specify the names**
Assessment of Inequalities in Coverage of Essential Reproductive, Maternal, Newborn, Child, and Adolescent Health Interventions in Kenya	2018	Keats et al.	https://doi.org/10.1001/jamanetworkopen.2018.5152	No	No	No	No	No	No	Yes	1) Composite Coverage Index (CCI); 2) the co-coverage indicator
Charting health system reconstruction in post-war Liberia: a comparison of rural vs. remote healthcare utilization	2016	Kentoffio et al.	https://doi.org/10.1186/s12913-016-1709-7	No	No	No	No	No	No	No	

1. BIBLIOGRAPHIC SOURCE				AGE								
**Title**	**Year**	**First author**	**UHL**	**Insecticide treated bed net (ITN)**	**Neglected tropical disease (NTD)**	**Prevention and treatment of raised**	**Prevention and treatment of raised**	**Cervical cancer screening**	**Tobacco (non-)smoking**	**Other UHC indicators (e.g., UHC index)**	**Other - Specify the names**	[redacted]
A Transparent Universal Health Coverage Index with Decomposition by Socioeconomic Groups: Application in Asian and African Settings	2019	Khan et al.	https://doi.org/10.1007/s40258-019-00464-9	No	No	No	No	No	No	Yes	Universal health coverage index	
Effect of user preferences on ITN use: a review of literature and data	2017	Koenker & Yukich	https://doi.org/10.1186/s12936-017-1879-8	Yes	No	No	No	No	No	No		

1. BIBLIOGRAPHIC SOURCE				AGE								
**Title**	**Year**	**First author**	**UHL**	**Insecticide treated bed net (ITN)**	**Neglected tropical disease (NTD)**	**Prevention and treatment of raised**	**Prevention and treatment of raised**	**Cervical cancer screening**	**Tobacco (non-)smoking**	**Other UHC indicators (e.g., UHC index)**	**Other - Specify the names**	[redacted]
Insecticide-treated net use before and after mass distribution in a fishing community along Lake Victoria, Kenya: successes and unavoidable pitfalls	2014	Larson et al.	http://www.malariajournal.com/content/13/1/466	Yes	No	No	No	No	No	No		
Lifetime Prevalence of Cervical Cancer Screening in 55 Low- and Middle-Income Countries	2020	Lemp et al.	https://doi.org/10.1001/jama.2020.16244	No	No	No	No	Yes	No	No		

1. BIBLIOGRAPHIC SOURCE				AGE							
**Title**	**Year**	**First author**	**UHL**	**Insecticide treated bed net (ITN)**	**Neglected tropical disease (NTD)**	**Prevention and treatment of raised**	**Prevention and treatment of raised**	**Cervical cancer screening**	**Tobacco (non-)smoking**	**Other UHC indicators (e.g., UHC index)**	**Other - Specify the names**
Financial accessibility and user fee reforms for maternal healthcare in five sub-Saharan countries: a quasi-experimental analysis	2016	Leone et al.	https://dx.doi.org/10.1136/bmjopen-2015-009692	No	No	No	No	No	No	No	
Antiretroviral treatment coverage in a rural district in Tanzania--a modeling study using empirical data	2015	Levira et al.	https://doi.org/10.1186/s12889-015-1460-8	No	No	No	No	No	No	No	
The extent of universal health coverage for maternal health services in eastern uganda: A cross sectional study	2021	Lindberg et al.	https://dx.doi.org/10.1007/s10995-021-03357-3	No	No	No	No	No	No	No	

1. BIBLIOGRAPHIC SOURCE				AGE								
**Title**	**Year**	**First author**	**UHL**	**Insecticide treated bed net (ITN)**	**Neglected tropical disease (NTD)**	**Prevention and treatment of raised**	**Prevention and treatment of raised**	**Cervical cancer screening**	**Tobacco (non-)smoking**	**Other UHC indicators (e.g., UHC index)**	**Other - Specify the names**	[redacted]
Freely distributed bed-net use among Chano Mille residents, south Ethiopia: A longitudinal study	2013	Loha et al.	https://doi.org/10.1186/1475-2875-12-23	Yes	No	No	No	No	No	No		
Determinants of hanging and use of ITNs in the context of near universal coverage in Zambia	2012	Macintyre et al.	https://doi.org/10.1093/heapol/czr042	Yes	No	No	No	No	No	No		

1. BIBLIOGRAPHIC SOURCE				AGE							
**Title**	**Year**	**First author**	**UHL**	**Insecticide treated bed net (ITN)**	**Neglected tropical disease (NTD)**	**Prevention and treatment of raised**	**Prevention and treatment of raised**	**Cervical cancer screening**	**Tobacco (non-)smoking**	**Other UHC indicators (e.g., UHC index)**	**Other - Specify the names**
Determinants and Consequences of Failure of Linkage to Antiretroviral Therapy at Primary Care Level in Blantyre, Malawi: A Prospective Cohort Study	2012	MacPherson et al.	https://doi.org/10.1371/journal.pone.0044794	No	No	No	No	No	No	No	
Disability and sexual and reproductive health service utilisation in Uganda: an intersectional analysis of demographic and health surveys between 2006 and 2016	2022	Mac-Seing et al.	https://doi.org/10.1186/s12889-022-12708-w	No	No	No	No	No	No	No	

1. BIBLIOGRAPHIC SOURCE				AGE							
**Title**	**Year**	**First author**	**UHL**	**Insecticide treated bed net (ITN)**	**Neglected tropical disease (NTD)**	**Prevention and treatment of raised**	**Prevention and treatment of raised**	**Cervical cancer screening**	**Tobacco (non-)smoking**	**Other UHC indicators (e.g., UHC index)**	**Other - Specify the names**
Insight into Nigeria's progress towards the universal coverage of reproductive, maternal, newborn and child health services: a secondary data analysis	2022	Mafiana et al.	http://dx.doi.org/10.1136/bmjopen-2022-061595	No	No	No	No	No	No	Yes	Reproductive, maternal, newborn and child health (RMNCH) index
Implementation of Urban Health Equity Assessment and Response Tool: a Case of Matsapha, Swaziland	2018	Makadzange et al.	https://doi.org/10.1007/s11524-018-0241-y	No	No	No	No	No	No	No	
Effects of door-to-door hang-up visits on the use of long-lasting insecticide-treated mosquito nets in the democratic republic of the congo: A cluster randomized controlled trial	2021	Mankadi and Jin	https://doi.org/10.3390/ijerph18179048	Yes	No	No	No	No	No	No	

1. BIBLIOGRAPHIC SOURCE				GE							
**Title**	**Year**	**First author**	**UHL**	**Insecticide treated bed net (ITN)**	**Neglected tropical disease (NTD)**	**Prevention and treatment of raised**	**Prevention and treatment of raised**	**Cervical cancer screening**	**Tobacco (non-)smoking**	**Other UHC indicators (e.g., UHC index)**	**Other - Specify the names**
Effect of bed net colour and shape preferences on bed net usage: a secondary data analysis of the 2017 Malawi Malaria Indicator Survey	2020	Mategula et al.	https://doi.org/10.1186/s12936-020-03499-9	Yes	No	No	No	No	No	No	
HIV care coverage among HIV-positive adolescent girls and young women in South Africa: Results from the HERStory Study	2021	Mathews et al.	https://doi.org/10.7196/SAMJ.2021.v111i5.15351	No	No	No	No	No	No	No	
Facilitators and barriers to retention in care under universal antiretroviral therapy (Option B+) for the Prevention of Mother to Child Transmission of HIV (PMTCT): A narrative review	2021	Mbeya Munkhondya et al.	https://doi.org/10.1016/j.ijans.2021.100372	No	No	No	No	No	No	No	

1. BIBLIOGRAPHIC SOURCE				AGE								
**Title**	**Year**	**First author**	**UHL**	**Insecticide treated bed net (ITN)**	**Neglected tropical disease (NTD)**	**Prevention and treatment of raised**	**Prevention and treatment of raised**	**Cervical cancer screening**	**Tobacco (non-)smoking**	**Other UHC indicators (e.g., UHC index)**	**Other - Specify the names**	[redacted]
The consequences of declining population access to insecticide-treated nets (ITNs) on net use patterns and physical degradation of nets after 22 months of ownership	2021	Mboma et al.	https://doi.org/10.1186/s12936-021-03686-2	Yes	No	No	No	No	No	No		
Mosquito net coverage in years between mass distributions: a case study of Tanzania, 2013	2018	Mboma et al.	https://doi.org/10.1186/s12936-018-2247-z	Yes	No	No	No	No	No	No		
Factors associated with contraceptive use in Tigray, North Ethiopia	2017	Medhanyie et al.	https://doi.org/10.1186/s12978-017-0281-x	No	No	No	No	No	No	No		

**1. BIBLIOGRAPHIC SOURCE**				**AGE**							
**Title**	**Year**	**First author**	**UHL**	**Insecticide treated bed net (ITN)**	**Neglected tropical disease (NTD)**	**Prevention and treatment of raised**	**Prevention and treatment of raised**	**Cervical cancer screening**	**Tobacco (non-)smoking**	**Other UHC indicators (e.g., UHC index)**	**Other - Specify the names**
Adolescent sexual and reproductive health in sub-Saharan Africa: who is left behind?	2020	Melesse et al.	https://doi.org/10.1136/bmjgh-2019-002231	No	No	No	No	No	No	No	
Sub-national levels and trends in contraceptive prevalence, unmet need, and demand for family planning in Nigeria with survey uncertainty	2019	Mercer et al.	https://doi.org/10.1186/s12889-019-8043-z	No	No	No	No	No	No	No	

1. BIBLIOGRAPHIC SOURCE				AGE							
Title	Year	First author	UHL	Insecticide treated bed net (ITN)	Neglected tropical disease (NTD)	Prevention and treatment of raised	Prevention and treatment of raised	Cervical cancer screening	Tobacco (non-)smoking	Other UHC indicators (e.g., UHC index)	Other - Specify the names
Exploring inequities in skilled care at birth among migrant population in a metropolitan city Addis Ababa, Ethiopia; A qualitative study	2014	Mirkuzie	http://www.equityhealthj.com/content/13/1/110	No	No	No	No	No	No	No	
Factors associated with the use of mosquito bed nets: results from two cross-sectional household surveys in Zambezia Province, Mozambique	2016	Moon et al.	https://doi.org/10.1186/s12936-016-1250-5	Yes	No	No	No	No	No	No	
Sociocultural and Institutional Constraints to Family Planning Uptake Among Migrant Female Head Porters in Madina, a Suburb of Accra, Ghana	2021	Munemo et al.	https://doi.org/10.1177/0886109920954419	No	No	No	No	No	No	No	

1. BIBLIOGRAPHIC SOURCE				[illegible]AGE								
**Title**	**Year**	**First author**	**UHL**	**Insecticide treated bed net (ITN)**	**Neglected tropical disease (NTD)**	**Prevention and treatment of raised**	**Prevention and treatment of raised**	**Cervical cancer screening**	**Tobacco (non-)smoking**	**Other UHC indicators (e.g., UHC index)**	**Other - Specify the names**	[redacted]
Reframing non-communicable diseases and injuries for equity in the era of universal health coverage: Findings and recommendations from the Kenya NCDI poverty commission	2021	Mwangi et al.	https://doi.org/10.5334/aogh.3085	No	No	Yes	Yes	Yes	No	No		[redacted]

1. BIBLIOGRAPHIC SOURCE				AGE							
**Title**	**Year**	**First author**	**UHL**	**Insecticide treated bed net (ITN)**	**Neglected tropical disease (NTD)**	**Prevention and treatment of raised**	**Prevention and treatment of raised**	**Cervical cancer screening**	**Tobacco (non-)smoking**	**Other UHC indicators (e.g., UHC index)**	**Other - Specify the names**
Inequities and their determinants in coverage of maternal health services in Burkina Faso	2018	Mwase et al.	https://doi.org/10.1186/s12939-018-0770-8	No	No	No	No	No	No	No	

1. BIBLIOGRAPHIC SOURCE				AGE								
Title	Year	First author	UHL	Insecticide treated bed net (ITN)	Neglected tropical disease (NTD)	Prevention and treatment of raised	Prevention and treatment of raised	Cervical cancer screening	Tobacco (non-)smoking	Other UHC indicators (e.g., UHC index)	Other - Specify the names	[redacted]
A Cross-Sectional Study on Hypertension Medication Adherence in a High-Burden Region in Namibia: Exploring Hypertension Interventions and Validation of the Namibia Hill-Bone Compliance Scale	2022	Nakwafila et al.	https://doi.org/10.3390/ijerph19074416	No	No	Yes	No	No	No	No		[redacted]
Assessing Adherence to Antihypertensive Therapy in Primary Health Care in Namibia: Findings and Implications	2017	Nashilongo et al.	https://doi.org/10.1007/s10557-017-6756-8	No	No	Yes	No	No	No	No		[redacted]

1. BIBLIOGRAPHIC SOURCE				AGE								
**Title**	**Year**	**First author**	**UHL**	**Insecticide treated bed net (ITN)**	**Neglected tropical disease (NTD)**	**Prevention and treatment of raised**	**Prevention and treatment of raised**	**Cervical cancer screening**	**Tobacco (non-)smoking**	**Other UHC indicators (e.g., UHC index)**	**Other - Specify the names**	[redacted]
Incomplete vaccination and associated factors among children aged 12–23 months in South Africa: an analysis of the South African demographic and health survey 2016	2021	Ndwandwe et al.	https://doi.org/10.1080/21645515.2020.1791509	No	No	No	No	No	No	No		
Determining the effective coverage of maternal and child health services in Kenya, using demographic and health survey data sets: tracking progress towards universal health coverage	2017	Nguhiu et al.	https://doi.org/10.1111/tmi.12841	Yes	No	No	No	No	No	No		

1. BIBLIOGRAPHIC SOURCE				AGE							
Title	Year	First author	UHL	Insecticide treated bed net (ITN)	Neglected tropical disease (NTD)	Prevention and treatment of raised	Prevention and treatment of raised	Cervical cancer screening	Tobacco (non-)smoking	Other UHC indicators (e.g., UHC index)	Other - Specify the names
Coverage and usage of insecticide treated nets (ITNs) within households: associated factors and effect on the prevalance of malaria parasitemia in the Mount Cameroon area	2019	Njumkeng et al.	https://doi.org/10.1186/s12889-019-7555-x	Yes	No	No	No	No	No	No	
Socioeconomic inequalities in maternal health care utilization in Ghana	2019	Novignon et al.	https://doi.org/10.1186/s12939-019-1043-x	No	No	No	No	No	No	No	

1. BIBLIOGRAPHIC SOURCE				AGE							
**Title**	**Year**	**First author**	**UHL**	**Insecticide treated bed net (ITN)**	**Neglected tropical disease (NTD)**	**Prevention and treatment of raised**	**Prevention and treatment of raised**	**Cervical cancer screening**	**Tobacco (non-)smoking**	**Other UHC indicators (e.g., UHC index)**	**Other - Specify the names**
Why rural women do not use primary health centres for pregnancy care: Evidence from a qualitative study in Nigeria	2019	Ntoimo et al.	https://doi.org/10.1186/s12884-019-2433-1	No	No	No	No	No	No	No	
Long-lasting insecticidal net (LLIN) ownership, use and cost of implementation after a mass distribution campaign in Kasaï Occidental Province, Democratic Republic of Congo	2017	Ntuku et al.	https://doi.org/10.1186/s12936-016-1671-1	Yes	No	No	No	No	No	No	
Explaining socioeconomic disparities and gaps in the use of antenatal care services in 36 countries in sub-Saharan Africa	2021	Obse & Ataguba	https://doi.org/10.1093/heapol/czab036	No	No	No	No	No	No	No	

1. BIBLIOGRAPHIC SOURCE				GE							
**Title**	**Year**	**First author**	**UHL**	**Insecticide treated bed net (ITN)**	**Neglected tropical disease (NTD)**	**Prevention and treatment of raised**	**Prevention and treatment of raised**	**Cervical cancer screening**	**Tobacco (non-)smoking**	**Other UHC indicators (e.g., UHC index)**	**Other - Specify the names**
Sociodemographic factors associated with the use of insecticide treated nets among under-fives in Nigeria: Evidence from a national survey	2022	Ojo et al.	https://doi.org/10.1177/00494755221110374	Yes	No	No	No	No	No	No	
Towards making efficient use of household resources for appropriate prevention of malaria: investigating households' ownership, use and expenditures on ITNs and other preventive tools in Southeast Nigeria	2014	Onwujekwe et al.	http://www.biomedcentral.com/1471-2458/14/315	Yes	No	No	No	No	No	No	

1. BIBLIOGRAPHIC SOURCE				AGE								
**Title**	**Year**	**First author**	**UHL**	**Insecticide treated bed net (ITN)**	**Neglected tropical disease (NTD)**	**Prevention and treatment of raised**	**Prevention and treatment of raised**	**Cervical cancer screening**	**Tobacco (non-)smoking**	**Other UHC indicators (e.g., UHC index)**	**Other - Specify the names**	[redacted]
Demographic disparities in unimproved drinking water and sanitation in Ghana: A nationally representative cross-sectional study	2022	Oppong et al.	http://dx.doi.org/10.1136/bmjopen-2021-060595	No	No	No	No	No	No	No		[redacted]
Inequities in Access to Maternal Health Care in Enugu State: Implications for Universal Health Coverage to Meet Vision 2030 in Nigeria	2019	Ozumba et al.	https://doi.org/10.1177/0272684X18819977	No	No	No	No	No	No	No		[redacted]

1. BIBLIOGRAPHIC SOURCE				AGE							
**Title**	**Year**	**First author**	**UHL**	**Insecticide treated bed net (ITN)**	**Neglected tropical disease (NTD)**	**Prevention and treatment of raised**	**Prevention and treatment of raised**	**Cervical cancer screening**	**Tobacco (non-)smoking**	**Other UHC indicators (e.g., UHC index)**	**Other - Specify the names**
How do supply- and demand-side interventions influence equity in healthcare utilisation? Evidence from maternal healthcare in Senegal	2019	Parmar & Banerjee	https://doi.org/10.1016/j.socscimed.2019.112582	No	No	No	No	No	No	No	
Temporal and regional variations in use, equity and quality of antenatal care in Egypt: A repeat cross-sectional analysis using Demographic and Health Surveys	2019	Pugliese-Garcia et al.	https://doi.org/10.1186/s12884-019-2409-1	No	No	No	No	No	No	No	

1. BIBLIOGRAPHIC SOURCE				AGE							
**Title**	**Year**	**First author**	**UHL**	**Insecticide treated bed net (ITN)**	**Neglected tropical disease (NTD)**	**Prevention and treatment of raised**	**Prevention and treatment of raised**	**Cervical cancer screening**	**Tobacco (non-)smoking**	**Other UHC indicators (e.g., UHC index)**	**Other - Specify the names**
Geographical Inequalities in Use of Improved Drinking Water Supply and Sanitation across Sub-Saharan Africa: Mapping and Spatial Analysis of Cross-sectional Survey Data	2014	Pullan et al.	https://doi.org/10.1371/journal.pmed.1001626	No	No	No	No	No	No	No	
Individual and Network Factors Associated With HIV Care Continuum Outcomes Among Nigerian MSM Accessing Health Care Services	2018	Ramadhani et al.	https://dx.doi.org/10.1097/QAI.0000000000001756	No	No	No	No	No	No	No	
Non-adherence to long-lasting insecticide treated bednet use following successful malaria control in Tororo, Uganda	2020	Rek et al.	https://doi.org/10.1371/journal.pone.0243303	Yes	No	No	No	No	No	No	

1. BIBLIOGRAPHIC SOURCE				AGE								
**Title**	**Year**	**First author**	**UHL**	**Insecticide treated bed net (ITN)**	**Neglected tropical disease (NTD)**	**Prevention and treatment of raised**	**Prevention and treatment of raised**	**Cervical cancer screening**	**Tobacco (non-)smoking**	**Other UHC indicators (e.g., UHC index)**	**Other - Specify the names**	[redacted]
Design, implementation and evaluation of a national campaign to deliver 18 million free long-lasting insecticidal nets to uncovered sleeping spaces in Tanzania	2013	Renggli et al.	https://doi.org/10.1186/1475-2875-12-85	Yes	No	No	No	No	No	No		
A long way to go - Estimates of combined water, sanitation and hygiene coverage for 25 sub-Saharan African countries	2017	Roche et al.	https://doi.org/10.1371/journal.pone.0171783	No	No	No	No	No	No	No		

1. BIBLIOGRAPHIC SOURCE				AGE							
**Title**	**Year**	**First author**	**UHL**	**Insecticide treated bed net (ITN)**	**Neglected tropical disease (NTD)**	**Prevention and treatment of raised**	**Prevention and treatment of raised**	**Cervical cancer screening**	**Tobacco (non-)smoking**	**Other UHC indicators (e.g., UHC index)**	**Other - Specify the names**
Determinants of bed net use in southeast Nigeria following mass distribution of LLINs: Implications for social behavior change interventions	2015	Russell et al.	https://doi.org/10.1371/journal.pone.0139447	Yes	No	No	No	No	No	No	
Factors associated with the non-use of insecticide-treated nets in Rwandan children	2016	Ruyange et al.	https://doi.org/10.1186/s12936-016-1403-6	Yes	No	No	No	No	No	No	

1. BIBLIOGRAPHIC SOURCE				AGE							
**Title**	**Year**	**First author**	**UHL**	**Insecticide treated bed net (ITN)**	**Neglected tropical disease (NTD)**	**Prevention and treatment of raised**	**Prevention and treatment of raised**	**Cervical cancer screening**	**Tobacco (non-)smoking**	**Other UHC indicators (e.g., UHC index)**	**Other - Specify the names**
Wealth Status, Health Insurance, and Maternal Health Care Utilization in Africa: Evidence from Gabon	2020	Sanogo & Yaya	https://dx.doi.org/10.1155/2020/4036830	No	No	No	No	No	No	No	

1. BIBLIOGRAPHIC SOURCE				AGE								
**Title**	**Year**	**First author**	**UHL**	**Insecticide treated bed net (ITN)**	**Neglected tropical disease (NTD)**	**Prevention and treatment of raised**	**Prevention and treatment of raised**	**Cervical cancer screening**	**Tobacco (non-)smoking**	**Other UHC indicators (e.g., UHC index)**	**Other - Specify the names**	[redacted]
Factors associated with use of insecticide-treated net for malaria prevention in Manica District, Mozambique: a community-based cross-sectional survey	2021	Scott et al.	https://doi.org/10.1186/s12936-021-03738-7	Yes	No	No	No	No	No	No		
Healthcare utilisation, cancer screening and potential barriers to accessing cancer care in rural South West Nigeria: a cross-sectional study	2021	Sharma et al.	https://doi.org/10.1136/bmjopen-2020-040352	No	No	No	No	Yes	No	No		

1. BIBLIOGRAPHIC SOURCE				AGE							
**Title**	**Year**	**First author**	**UHL**	**Insecticide treated bed net (ITN)**	**Neglected tropical disease (NTD)**	**Prevention and treatment of raised**	**Prevention and treatment of raised**	**Cervical cancer screening**	**Tobacco (non-)smoking**	**Other UHC indicators (e.g., UHC index)**	**Other - Specify the names**
Socio-economic inequalities in ANC attendance among mothers who gave birth in the past 12 months in Debre Brehan town and surrounding rural areas, North East Ethiopia: A community-based survey	2019	Shibre & Mekonnen	https://doi.org/10.1186/s12978-019-0768-8	No	No	No	No	No	No	No	

1. BIBLIOGRAPHIC SOURCE				AGE							
**Title**	**Year**	**First author**	**UHL**	**Insecticide treated bed net (ITN)**	**Neglected tropical disease (NTD)**	**Prevention and treatment of raised**	**Prevention and treatment of raised**	**Cervical cancer screening**	**Tobacco (non-)smoking**	**Other UHC indicators (e.g., UHC index)**	**Other - Specify the names**
Individual and environmental characteristics associated with immunization of children in rural areas of Burkina Faso: A multi-level analysis	2007	Sia et al.	https://pubmed.ncbi.nlm.nih.gov/18299262/	No	No	No	No	No	No	No	
Inequalities in access and utilization of maternal, newborn and child health services in sub-saharan africa: A special focus on urban settings	2021	Sidze et al.	https://dx.doi.org/10.1007/s10995-021-03250-z	No	No	No	No	No	No	No	

1. BIBLIOGRAPHIC SOURCE				AGE								
**Title**	**Year**	**First author**	**UHL**	**Insecticide treated bed net (ITN)**	**Neglected tropical disease (NTD)**	**Prevention and treatment of raised**	**Prevention and treatment of raised**	**Cervical cancer screening**	**Tobacco (non-)smoking**	**Other UHC indicators (e.g., UHC index)**	**Other - Specify the names**	[redacted]
Insecticide-treated bed net access and use among preschool children in Nouna District, Burkina Faso	2020	Sié et al.	https://dx.doi.org/10.1093/inthealth/ihaa003	Yes	No	No	No	No	No	No		
Evidence of improving antiretroviral therapy treatment delays: an analysis of eight years of programmatic outcomes in Blantyre, Malawi	2013	Sloan et al.	http://www.biomedcentral.com/1471-2458/13/490	No	No	No	No	No	No	No		
Low use of long-lasting insecticidal nets for malaria prevention in south-central Ethiopia: A community-based cohort study	2019	Solomon et al.	https://doi.org/10.1371/journal.pone.0210578	Yes	No	No	No	No	No	No		

1. BIBLIOGRAPHIC SOURCE				AGE							
Title	Year	First author	UHL	Insecticide treated bed net (ITN)	Neglected tropical disease (NTD)	Prevention and treatment of raised	Prevention and treatment of raised	Cervical cancer screening	Tobacco (non-)smoking	Other UHC indicators (e.g., UHC index)	Other - Specify the names
Empowerment and use of modern contraceptive methods among married women in Burkina Faso: a multilevel analysis	2021	Some et al.	https://dx.doi.org/10.1186/s12889-021-11541-x	No	No	No	No	No	No	No	
Evaluation of the 2011 long-lasting, insecticide-treated net distribution for universal coverage in Togo	2013	Stevens et al.	http://www.malariajournal.com/content/12/1/162	Yes	No	No	No	No	No	No	
On the way to universal coverage of maternal services in Iringa rural District in Tanzania. Who is yet to be reached?	2016	Straneo et al.	https://doi.org/10.4314/ahs.v16i2.10	No	No	No	No	No	No	No	

1. BIBLIOGRAPHIC SOURCE				AGE								
**Title**	**Year**	**First author**	**UHL**	**Insecticide treated bed net (ITN)**	**Neglected tropical disease (NTD)**	**Prevention and treatment of raised**	**Prevention and treatment of raised**	**Cervical cancer screening**	**Tobacco (non-)smoking**	**Other UHC indicators (e.g., UHC index)**	**Other - Specify the names**	[redacted]
Evaluation of long-lasting insecticidal net distribution through schools in Southern Tanzania	2022	Stuck et al.	https://doi.org/10.1093/heapol/czab140	Yes	No	No	No	No	No	No		[redacted]
Not all inequalities are equal: differences in coverage across the continuum of reproductive health services	2019	Sully et al.	https://dx.doi.org/10.1136/bmjgh-2019-001695	No	No	No	No	No	No	No		[redacted]

1. BIBLIOGRAPHIC SOURCE				AGE							
**Title**	**Year**	**First author**	**UHL**	**Insecticide treated bed net (ITN)**	**Neglected tropical disease (NTD)**	**Prevention and treatment of raised**	**Prevention and treatment of raised**	**Cervical cancer screening**	**Tobacco (non-)smoking**	**Other UHC indicators (e.g., UHC index)**	**Other - Specify the names**
Determinants of long-lasting insecticidal net ownership and utilization in malaria transmission regions: Evidence from Zimbabwe Demographic and Health Surveys	2019	Tapera	https://doi.org/10.1186/s12936-019-2912-x	Yes	No	No	No	No	No	No	
Sociodemographic inequities in cervical cancer screening, treatment and care amongst women aged at least 25 years: evidence from surveys in Harare, Zimbabwe	2019	Tapera et al.	https://doi.org/10.1186/s12889-019-6749-6	No	No	No	No	Yes	No	No	

1. BIBLIOGRAPHIC SOURCE				AGE								
**Title**	**Year**	**First author**	**UHL**	**Insecticide treated bed net (ITN)**	**Neglected tropical disease (NTD)**	**Prevention and treatment of raised**	**Prevention and treatment of raised**	**Cervical cancer screening**	**Tobacco (non-)smoking**	**Other UHC indicators (e.g., UHC index)**	**Other - Specify the names**	[redacted]
Factors associated to bed net use in Cameroon: a retrospective study in Mfou health district in the Centre Region	2012	Tchinda et al.	http://www.panafrican-med-journal.com/content/article/12/112/full/	Yes	No	No	No	No	No	No		
Cervical cancer screening uptake and correlates among HIV-infected women: a cross-sectional survey in Cote d'Ivoire, West Africa	2019	Tchounga et al.	https://dx.doi.org/10.1136/bmjopen-2019-029882	No	No	No	No	Yes	No	No		

1. BIBLIOGRAPHIC SOURCE				AGE							
**Title**	**Year**	**First author**	**UHL**	**Insecticide treated bed net (ITN)**	**Neglected tropical disease (NTD)**	**Prevention and treatment of raised**	**Prevention and treatment of raised**	**Cervical cancer screening**	**Tobacco (non-)smoking**	**Other UHC indicators (e.g., UHC index)**	**Other - Specify the names**
Duration and determinants of delayed tuberculosis diagnosis and treatment in high-burden countries: a mixed-methods systematic review and meta-analysis	2021	Teo et al.	https://doi.org/10.1186/s12931-021-01841-6	No	No	No	No	No	No	No	
Skilled delivery inequality in Ethiopia: To what extent are the poorest and uneducated mothers benefiting?	2017	Tesfaye et al.	https://doi.org/10.1186/s12939-017-0579-x	No	No	No	No	No	No	No	

1. BIBLIOGRAPHIC SOURCE				AGE							
**Title**	**Year**	**First author**	**UHL**	**Insecticide treated bed net (ITN)**	**Neglected tropical disease (NTD)**	**Prevention and treatment of raised**	**Prevention and treatment of raised**	**Cervical cancer screening**	**Tobacco (non-)smoking**	**Other UHC indicators (e.g., UHC index)**	**Other - Specify the names**
Investigating the association between pregnancy intention and insecticide-treated bed net (ITN) use: A cross-sectional study of pregnant women in Rwanda	2015	Thogarapalli et al.	https://doi.org/10.1007/s10389-015-0676-5	Yes	No	No	No	No	No	No	
Success of Senegal's first nationwide distribution of long-lasting insecticide-treated nets to children under five - Contribution toward universal coverage	2011	Thwing et al.	http://www.malariajournal.com/content/10/1/86	Yes	No	No	No	No	No	No	
Declines in Malaria Burden and all-cause child mortality following increases in control interventions in Senegal, 2005-2010	2017	Thwing et al.	https://doi.org/10.4269/ajtmh.16-0953	Yes	No	No	No	No	No	No	

1. BIBLIOGRAPHIC SOURCE				AGE							
**Title**	**Year**	**First author**	**UHL**	**Insecticide treated bed net (ITN)**	**Neglected tropical disease (NTD)**	**Prevention and treatment of raised**	**Prevention and treatment of raised**	**Cervical cancer screening**	**Tobacco (non-)smoking**	**Other UHC indicators (e.g., UHC index)**	**Other - Specify the names**
Individual-level and community-level determinants of cervical cancer screening among Kenyan women: a multilevel analysis of a Nationwide survey	2017	Tiruneh et al.	https://doi.org/10.1186/s12905-017-0469-9	No	No	No	No	Yes	No	No	
Evaluation of the coverage and effective use rate of long-lasting insecticidal nets after nation-wide scale up of their distribution in Benin	2013	Tokponnon et al.	https://doi.org/10.1186/1756-3305-6-265	Yes	No	No	No	No	No	No	
Factors associated with the upsurge in the use of delivery care services in Sierra Leone	2020	Tsawe & Susuman	https://doi.org/10.1016/j.puhe.2019.11.002	No	No	No	No	No	No	No	

1. BIBLIOGRAPHIC SOURCE				AGE							
**Title**	**Year**	**First author**	**UHL**	**Insecticide treated bed net (ITN)**	**Neglected tropical disease (NTD)**	**Prevention and treatment of raised**	**Prevention and treatment of raised**	**Cervical cancer screening**	**Tobacco (non-)smoking**	**Other UHC indicators (e.g., UHC index)**	**Other - Specify the names**
Which family members use the best nets? An analysis of the condition of mosquito nets and their distribution within households in Tanzania	2010	Tsuang et al.	http://www.malariajournal.com/content/9/1/211	Yes	No	No	No	No	No	No	
Utilization of insecticide treated nets among pregnant women in enugu, South Eastern Nigeria	2013	Ugwu et al.	https://pubmed.ncbi.nlm.nih.gov/23771448/	Yes	No	No	No	No	No	No	

1. BIBLIOGRAPHIC SOURCE				AGE								
**Title**	**Year**	**First author**	**UHL**	**Insecticide treated bed net (ITN)**	**Neglected tropical disease (NTD)**	**Prevention and treatment of raised**	**Prevention and treatment of raised**	**Cervical cancer screening**	**Tobacco (non-)smoking**	**Other UHC indicators (e.g., UHC index)**	**Other - Specify the names**	[redacted]
Finding the gap: Revealing local disparities in coverage of maternal, newborn and child health services in South Sudan using lot quality assurance sampling	2015	Valadez et al.	https://doi.org/10.1111/tmi.12613	No	No	No	No	No	No	No		
Group Medical Visit and Microfinance Intervention for Patients With Diabetes or Hypertension in Kenya	2021	Vedanthan et al.	https://doi.org/10.1016/j.jacc.2021.03.002	No	No	Yes	No	No	No	No		

1. BIBLIOGRAPHIC SOURCE				AGE							
**Title**	**Year**	**First author**	**UHL**	**Insecticide treated bed net (ITN)**	**Neglected tropical disease (NTD)**	**Prevention and treatment of raised**	**Prevention and treatment of raised**	**Cervical cancer screening**	**Tobacco (non-)smoking**	**Other UHC indicators (e.g., UHC index)**	**Other - Specify the names**
Equity in Maternal Health in South Africa: Analysis of Health Service Access and Health Status in a National Household Survey	2013	Wabiri et al.	https://doi.org/10.1371/journal.pone.0073864	No	No	No	No	No	No	No	
Persisting Regional Disparities in Modern Contraceptive Use and Unmet Need for Contraception among Nigerian Women	2019	Wang & Cao	https://doi.org/10.1155/2019/9103928	No	No	No	No	No	No	No	

1. BIBLIOGRAPHIC SOURCE				AGE							
**Title**	**Year**	**First author**	**UHL**	**Insecticide treated bed net (ITN)**	**Neglected tropical disease (NTD)**	**Prevention and treatment of raised**	**Prevention and treatment of raised**	**Cervical cancer screening**	**Tobacco (non-)smoking**	**Other UHC indicators (e.g., UHC index)**	**Other - Specify the names**
Effective coverage of facility delivery in Bangladesh, Haiti, Malawi, Nepal, Senegal, and Tanzania	2019	Wang et al.	https://doi.org/10.1371/journal.pone.0217853	No	No	No	No	No	No	No	
Use of long-lasting insecticide-treated bed nets in a population with universal coverage following a mass distribution campaign in Uganda	2016	Wanzira et al.	https://doi.org/10.1186/s12936-016-1360-0	Yes	No	No	No	No	No	No	

1. BIBLIOGRAPHIC SOURCE				AGE							
**Title**	**Year**	**First author**	**UHL**	**Insecticide treated bed net (ITN)**	**Neglected tropical disease (NTD)**	**Prevention and treatment of raised**	**Prevention and treatment of raised**	**Cervical cancer screening**	**Tobacco (non-)smoking**	**Other UHC indicators (e.g., UHC index)**	**Other - Specify the names**
Long-lasting insecticide-treated bed net ownership and use among children under five years of age following a targeted distribution in central Uganda	2014	Wanzira et al.	https://doi.org/10.1186/1475-2875-13-185	Yes	No	No	No	No	No	No	
Evaluation of a national universal coverage campaign of long-lasting insecticidal nets in a rural district in north-west Tanzania	2012	West et al.	https://doi.org/10.1186/1475-2875-11-273	Yes	No	No	No	No	No	No	
The impact of renewing long-lasting insecticide-treated nets in the event of malaria resurgence: Lessons from 10 years of net use in dielmo, Senegal	2021	Wotodjo et al.	https://doi.org/10.4269/AJTMH.20-0127	Yes	No	No	No	No	No	No	

1. BIBLIOGRAPHIC SOURCE				AGE							
**Title**	**Year**	**First author**	**UHL**	**Insecticide treated bed net (ITN)**	**Neglected tropical disease (NTD)**	**Prevention and treatment of raised**	**Prevention and treatment of raised**	**Cervical cancer screening**	**Tobacco (non-)smoking**	**Other UHC indicators (e.g., UHC index)**	**Other - Specify the names**
Wealth and Education Inequities in Maternal and Child Health Services Utilization in Rural Ethiopia	2022	Wuneh et al.	https://doi.org/10.3390/ijerph190954 21	No	No	No	No	No	No	No	
Inequalities in maternal health care utilization in Benin: A population based cross-sectional study	2018	Yaya et al.	https://doi.org/10.1186/s12884-018-1846-6	No	No	No	No	No	No	No	

1. BIBLIOGRAPHIC SOURCE				AGE								[redacted]
**Title**	**Year**	**First author**	**UHL**	**Insecticide treated bed net (ITN)**	**Neglected tropical disease (NTD)**	**Prevention and treatment of raised**	**Prevention and treatment of raised**	**Cervical cancer screening**	**Tobacco (non-)smoking**	**Other UHC indicators (e.g., UHC index)**	**Other - Specify the names**	
Long-lasting insecticide-treated bed net ownership, utilization and associated factors among school-age children in Dara Mallo and Uba Debretsehay districts, Southern Ethiopia	2020	Zerdo et al.	https://dx.doi.org/10.1186/s12936-020-03437-9	Yes	No	No	No	No	No	No		
Trends and projections of universal health coverage indicators in Ghana, 1995-2030: A national and subnational study	2019	Zhang et al.	https://doi.org/10.1371/journal.pone.0209126	Yes	No	No	No	No	Yes	Yes	Composite Coverage Index (CCI)	

1. BIBLIOGRAPHIC SOURCE				AGE							
**Title**	**Year**	**First author**	**UHL**	**Insecticide treated bed net (ITN)**	**Neglected tropical disease (NTD)**	**Prevention and treatment of raised**	**Prevention and treatment of raised**	**Cervical cancer screening**	**Tobacco (non-)smoking**	**Other UHC indicators (e.g., UHC index)**	**Other - Specify the names**
Developing Malawi's Universal Health Coverage Index	2022	Mchenga et al.	https://doi.org/10.3389/frhs.2021.786186	Yes	No	Yes	Yes	Yes	Yes	No	Universal Health Coverage (UHC) index
Socioeconomic Factors Associated with Compliance with Mass Drug Administration for Lymphatic Filariasis Elimination in Kenya: Descriptive Study Results	2012	Njomo et al.	https://www.proquest.com/docview/1115911778?pq-origsite=gscholar&fromopenview=true	No	Yes	No	No	No	No	No	

1. BIBLIOGRAPHIC SOURCE				[illegible]AGE							
**Title**	**Year**	**First author**	**UHL**	**Insecticide treated bed net (ITN)**	**Neglected tropical disease (NTD)**	**Prevention and treatment of raised**	**Prevention and treatment of raised**	**Cervical cancer screening**	**Tobacco (non-)smoking**	**Other UHC indicators (e.g., UHC index)**	**Other - Specify the names**
Equality analysis of main health indicators among children under 5 years in Uganda	2019	Elduma	http://dx.doi.org/10.4314/ejhs.v29i2.8	Yes	No	No	No	No	No	No	
