# Supplementary material for: Mapping inequalities in health service coverage in Africa: a scoping review
Source: BMJ Open. 2024 Nov 24;14(11):e082918. doi: 10.1136/bmjopen-2023-082918 (PMC11590813; doi:10.1136/bmjopen-2023-082918)
Supplement: online supplemental table 1 [file bmjopen-14-11-s004.pdf]

Supplementary Table 1. Characteristics of Included Studies

Categorical variables	n	%		Categorical variables	n	%
**African regions involved**[1]				**Type of data source**[1]		
Single country	145	81.5		Primary source	75	42.1
East	68	38.2		Secondary source	96	53.9
West	58	32.6		Primary and secondary source	7	3.9
South	12	6.7		**Type of data collected**[1]		
Middle	6	3.4		Quantitative data	164	92.1
North	1	0.6		Qualitative data	6	3.4
Multiple countries	33	18.5		Mixed data	8	4.5
**Year of publication**[1]				**Sample coverage**[1]		
2007	1	0.6		National	92	51.7
2010 - 2013	24	13.5		Subnational	73	41.0
2014 - 2017	44	24.7		Organizational	8	4.5
2018 - 2022	109	61.2		Multiple levels	5	2.8
**Study design**[1]				**Types of funding**[1]		
Knowledge syntheses	4	2.2		Non commercial	102	57.3
Experimental or quasi-experimental trials	12	6.7		No funding	36	20.2
Cross-sectional studies	141	79.2		Unclear	40	22.5
Longitudinal studies	11	6.2		**Services for RMNCH**[2]	96	53.9
Qualitative studies	5	2.8		Family planning	37	20.8

Mixed methods studies	2	1.1		Antenatal or prenatal care	48	27.0
Multi-pronged study designs	3	1.7		Skilled birth attendance	34	19.1
**Inequality stratifiers**[2]				Cesarean delivery care	2	1.1
Place of residence	105	59.0		Postnatal care	12	6.7
Race, ethnicity, or culture	7	3.9		Child immunization	25	14.0
Occupation	47	26.4		Health-seeking behaviour for pneumonia	8	4.5
Sex or gender	61	34.3		**Services for infectious diseases**[2]	77	43.3
Religion	29	16.3		Tuberculosis treatment	3	1.7
Education	110	61.8		Human immunodeficiency virus (HIV) therapy	13	7.3
Socioeconomic status	125	70.2		Basic sanitation	11	6.2
Social capital or ressources	38	21.3		Insecticide treated bed net (ITN)	54	30.3
Age	111	62.4		Neglected tropical disease (NTD) treatment	2	1.1
Disability	3	1.7		**Services for noncommunicable diseases**[2]	15	8.4
**Categorical variables**	**median (IQR)**			Prevention and treatment of raised blood pressure (i.e., hypertension)	7	3.9
Number of stratifiers (range: 1-8)	3 (3)			Prevention and treatment of raised blood glucose (i.e., diabetes)	4	2.2
Number of health service coverage indicators (range: 0-15)	1 (1)			Cervical cancer screening	10	5.6
Number of indicators for RMNCH services (range: 0-6)	1 (1)			Tobacco non-smoking	4	2.2
Number of indicators for infection disease services (range: 0-4)	0 (1)			**UHC composite indices**	6	3.4

Number of indicators for noncommunicable disease services (range: 0-4)	0 (0)				
Number of composite indicators for UHC (range: 0-1)	0 (0)				

Abbreviations: HIV: human immunodeficiency virus; ITN: insecticide treated bed net; RMNCH: Reproductive, maternal, newborn and child health; WHO: World Health Organization; IQR: interquartile range; UHC: universal health coverage;
Notes: [1]Percentages in the column may add up to ± 100% because of rounding error; [2]The numbers or percentages in the column do not add up since they are not mutually exclusive.
