# Supplementary material for: Mapping inequalities in health service coverage in Africa: a scoping review
Source: BMJ Open. 2024 Nov 24;14(11):e082918. doi: 10.1136/bmjopen-2023-082918 (PMC11590813; doi:10.1136/bmjopen-2023-082918)
Supplement: Supplementary file 5 [file bmjopen-2023-082918.reviewer_comments.pdf]

PEER REVIEW HISTORY

BMJ Open publishes all reviews undertaken for accepted manuscripts. Reviewers are asked to complete a checklist review form and are provided with free text boxes to elaborate on their assessment. These free text comments are reproduced below.

ARTICLE DETAILS

Title (Provisional)

Mapping inequalities in health service coverage in Africa: A scoping review

Authors

Karamagi, Humphrey Cyprian; Afriyie, Doris Osei; Ben Charif, Ali; Sy, Sokona; Kipruto, Hillary; Moyo, Thandelike; Oyelade, Taiwo; Droti, Benson

---

VERSION 1 - REVIEW

**Reviewer**	**1**
**Name**	**Winata, Fikriyah**
**Affiliation**	**Texas A&M University, Geography**
**Date**	**07-Feb-2024**
**COI**	**No competing interests and I reviewed the paper by myself.**

Review

Thank you for conducting a scoping review study addressing the health service inequalities in Africa. This study is needed and has the potential to be published in the BMJ Open. Reading through the manuscript, I found a few things that need to be addressed and clarified. In addition, I provide a few suggestions to improve the paper.

Abstract:

1. The authors need to briefly add one sentence to describe what the JBI Manual for Evidence Synthesis is.
2. Was there any specific reason to only review articles published from January 1, 2005, to August 29, 2022? How about papers published before 2005? It is imperative to justify the cut-off time of papers in this time frame.

Introduction:

1. The authors need to expand the SDS since this abbreviation first appeared in the text.
2. What did ‘A rising tide lifts all boats’ mean in this context?
3. Similar comments as #1 for the PHC.

Methods:

1. Although the authors mentioned ABC and DOA did the "full-text eligibility review," I could not find the inclusions and exclusions of the eligibility criteria of their review. I suggest the author provide their inclusions and exclusions criteria in the methods section.
2. I suggest the author provide a scheme or flow diagram of their review process since the first stage of review includes a number of papers that were reviewed in the first stage (title and abstract) and so on. This diagram is very standard for a review paper, and it will be helpful for readers to understand the process of scoping review process.

Results:

1. The percentage information the authors provided on the results and authors labeled as quantitative findings could have been clearer because the info presented mainly was percentage and descriptive. I suggest the author change the wording label rather than using "quantitative findings." There are similar comments for the "quantitative" figure as well. Such as Figure 1, the authors labeled Figure 1 as quantitative findings; I would instead call it the number of service utilization by equity stratifier, just like what the authors put on the diagram. I do not think this diagram is quantitative findings because whenever something is labeled as quantitative findings, the readers expect to see the statistical modeling results or at least the descriptive statistics. I have a similar comment with Figure 2, too. If the 'quantitative findings' in this paper imply the quantitative findings in the papers they reviewed, please differentiate them. Later, I noticed that in some texts, the wording "quantitative findings" implies the quantitative method that the papers used.
2. **Place of residence:** One of the authors even commented (on the comment box) "how many," implying that the manuscript they submitted still had an internal review by the authors. I agree to add how many studies reported the urban-rural gap has declined.
3. **Gender:** rather than "biological differences (men or women" I would say using gender differences is better, especially the authors labeled the subsection of Gender.
4. This sentence needs a citation "In South-South Nigeria; one study reported that although water, sanitation, and hygiene (WASH) services are poor for slum dwellers, women in slums bear the disproportionate burden of inadequate services due to the gendered distribution of childcare, household work and the need to fulfil the personal sanitary needs." Also, the rest of the sentence in the paragraph needs a citation.
5. Some things could be more consistent in providing citations. In some subsections, the authors provided citation numbers after "six studies (then citation numbers) including in the Age subsection." However, I found in another subsection, such as in the Gender subsection, "Fout of the quantitative studies examined…" no citations of these four studies. Also, in the Language subsection, "seven quantitative studies reported…" but no citations were provided. Please be consistent with how many numbers of studies you want to provide citations (I understand maybe less than ten?), and they must be consistent throughout the paper.
6. What is Plus? I am not sure "Plus" as subsection heading.
7. Overall: Each subsection (place of residence, education, gender, language/race/ethnicity, religion, occupation, social capital, age, and plus is

mostly descriptive of several papers, whether reported quantitative or qualitative findings. I hope the authors can provide some helpful narrative or interpretation of the results, like what do the findings mean. Why do they matter? Why are they important for the African region? A bit of context and interpretation will be helpful rather than only reporting the number of studies found.

Discussion:

1. Please check some grammatical errors in the text, including using capital letters and not capital letters in the middle of the sentence.
2. These sentences need citations, "For example, the review showed that structural and financial social support from spouses were crucial factors for women using ANC in Ghana." Also, the following sentence needs citation, "In Ethiopia,….."
3. I suggest discussing the strengths of the study, then followed by the limitations, not the other way around.

Additional comment: I suggest the author mention whether the study included all papers from all countries in the African continent (including Arab-speaking countries in North Africa). I bring up this because Arab-speaking countries in North Africa culturally differ from the rest of Africa, and sometimes, from the regional standpoint, this part of Africa is better known as part of the Middle East. So, it is important to mention whether the paper includes studies from those countries in North Africa or not. If the paper included those countries, I just suggest the authors to mention it in the paper briefly, maybe somewhere in the introduction section.

**Reviewer**	**2**
**Name**	**Achoki, Tom**
**Affiliation**	**University of Pretoria , School of Health Systems and Public Health**
**Date**	**07-Mar-2024**
**COI**	**None**

Thank you for the opportunity to review this paper that covers an important topic of health inequalities. While I appreciate the significant effort that has been put into this paper, I would like to recommend major revisions to strengthen it.

This paper needs through proofreading before submission. There are obvious grammar issues and incomplete edits (page 7 line 37) that are included in the submitted manuscript that need to be addressed before the paper is submitted for peer review.

More specifically,

Abstract:

The abbreviations are included in a very unstructured manner, and it is difficult to follow the train of the arguments presented.

The strengths and limitations:

It is not very clear what the strengths of the paper are considering what is reported on this section-- restrictive MESH terms are not a strength. In addition, the second limitation of not ensuring the quality of the papers presented is concerning and casts questions on what has been included in the results. Finally, there is no mention on how articles published in other languages other than English were handled. That is a limitation that needs to be reported.

Introduction:

This section needs proofreading and better elaboration. The literature review here needs to be strengthened. Has this kind of review been done elsewhere? What is different with your work? what additional value does your work present? This could include work done in other regions so that the reader appreciates that your work is comparable to others and that there is added value.

Methods:

The methods are fairly well described but there are fundamental issues that weaken this paper. It would be important to address the issue of how the papers were assessed for quality for inclusion and exclusion. How was the issue of language beyond English addressed? Another major question revolves around the measures of health outcomes included in this paper might not be exhaustive. There is significant work that has been done in Africa on the subnational burden of disease in Ethiopia, Kenya and South Africa on overall health inequalities and seems to be missing from your reporting.

Results:

The results section needs a lot of work if this paper is to be ready for publication. The information needs to be better organized. Lumping quantitative and qualitative results per inequality driver makes for difficult reading. It would be better to have distinct sections dealing with quantitative and qualitative results separately. Some of the results that are reported are insignificant and of no consequence and therefore can be eliminated so that the section is better structured.

The figures are not fully labeled and therefore makes it difficult to know what is being presented in the results. It would be better to stick with reporting proportions in the figures so that it's not duplicative (numbers and proportions in the text).

Discussion:

The discussion could be strengthened by leaning on the health system framework as elaborated by the WHO and covering the different facets of inequality. For instance, the aspect of cost effectiveness and efficiency are missing from the discussion. A more detailed and directed literature review based on the results could strengthen the discussion. -- what

are the health policy implications and future research? what are the limitations of this work that readers need to pay attention.

****The reviewer provided a marked copy with additional comments. Please contact the publisher for full details.**

VERSION 1 - AUTHOR RESPONSE

Reviewer	Comment	Response
**Abstract**		
1	The authors need to briefly add one sentence to describe what the JBI Manual for Evidence Synthesis is.	Thank you for your comment. The JBI Manual for Evidence Synthesis is a manual providing guidance on developing systematic reviews. The clarification has been added. Indeed, this manual guides authors who wish to conduct systematic and scoping reviews following JBI methodologies. As explained in the published protocol: "*[the JBI] methodology is based on the Arksey and O'Malley's framework*[15] *and an enhanced version developed by Levac and colleagues*[16] *for conducting scoping reviews.*"<br><br>Please note that all comprehensive details regarding the methodologies employed in this study have been previously provided in the published protocol: https://doi.org/10.1136/bmjopen-2022-068903. Thus, we have added the following sentence: "Given the comprehensive nature of the methods outlined in our protocol, we prioritized providing more detailed results in our manuscript and directing our readers to the protocol for further information on the methodology employed."
1	Was there any specific reason to only review articles published from January 1, 2005, to August 29, 2022? How about papers published before 2005? It is imperative to justify the cut-off time of papers in this time frame.	Thank you for your comment. The timeframe was narrowed to January 1, 2005, to August 29, 2022, because 2005 marks the first time of the mention of UHC into public health discourse.
2	The abbreviations are included in a very unstructured manner, and it is difficult to follow the train of the arguments presented.	Thank you for your comment. All abbreviations have been structured following the journal guidelines.

**Strengths and limitations**		
2	It is not very clear what the strengths of the paper are considering what is reported on this section-- restrictive MESH terms are not a strength. In addition, the second limitation of not ensuring the quality of the papers presented is concerning and casts questions on what has been included in the results. Finally, there is no mention on how articles published in other languages other than English were handled. That is a limitation that needs to be reported.	Thank you for your comment, both sections have been updated accordingly. Strengths and limitations have been updated as follows:<br>• A strength of our scoping review is bringing together a wide scope of studies using a comprehensive strategy.<br>• By applying the PROGRESS-Plus framework, we ensured a broader dimension of social process factors to uncover inequities in service utilization.<br>• We focused on peer-reviewed papers only and assumed that these would be of high quality.<br>• Adapting the WHO UHC service coverage indicators prevented us from exploring inequalities in the quality of services provided, as the coverage indicators are primarily focused on utilization.<br>• Research was skewed towards certain drivers of health inequalities such as language and ethnicity.<br>• Our search strategy primarily included articles published in English due to resource constraints and the predominant use of English in the databases we searched.
**Introduction**		
1	The authors need to expand the SDS since this abbreviation first appeared in the text.	Thank you for highlighting this. We meant, SDG and not SDS. This has been corrected.
1	What did ‘A rising tide lifts all boats’ mean in this context?	Thank you for your comment. In this context, the phrase "a rising tide lifts all boats" is used to highlight the importance of distinguishing between improvements that are due to specific interventions and those that are due to broader, unrelated factors. For example, an improving economy may lead to better health outcomes. Therefore, the health sector should not claim credit for health improvements that are actually a consequence of economic growth. The rising tide (improving economy) lifts all boats (health, education, gender equality, etc.), meaning that different sectors may benefit simultaneously from general economic improvements rather than from sector-specific interventions.
1	Similar comments as #1 for the PHC.	Thank you for your comment, this has been revised in the manuscript.

2	This section needs proofreading and better elaboration. The literature review here needs to be strengthened. Has this kind of review been done elsewhere? What is different with your work? what additional value does your work present? This could include work done in other regions so that the reader appreciates that your work is comparable to others and that there is added value.	Thank you for your comment. Few reviews have specifically examined inequalities in the context of universal health coverage. The limited reviews that have been conducted have focused primarily on specific services, such as reproductive, maternal, newborn, and child health (RMNCH) services. In addition, other reviews have assessed disparities using selected stratifiers, such as socioeconomic status and age.<br><br>In this review, we aim to consolidate the evidence on inequalities in service coverage in Africa using a comprehensive set of stratifiers. These stratifiers include, but are not limited to, socioeconomic status, age, gender, geographic location, and ethnicity. Our approach provides a more holistic understanding of the different dimensions of inequality in the context of UHC.
**Methods**		
1	Although the authors mentioned ABC and DOA did the "full-text eligibility review," I could not find the inclusions and exclusions of the eligibility criteria of their review. I suggest the author provide their inclusions and exclusions criteria in the methods section.	As suggested, we have added the following sentences and cited the published protocol: "We included any empirical research that assessed inequalities in relation to services for reproductive, maternal, newborn and child health (e.g., family planning), infectious diseases (e.g., tuberculosis treatment) and non-communicable diseases (e.g., cervical cancer screening) in Africa (11)."<br><br>Indeed, all comprehensive details regarding the methodologies employed in this study have been previously provided in the published protocol: https://doi.org/10.1136/bmjopen-2022-068903. This includes detailed information about inclusion and exclusion criteria and summary table of these criteria. We have also added the following sentence: "Given the comprehensive nature of the methods outlined in our protocol, we prioritized providing more detailed results in our manuscript and directing our readers to the protocol for further information on the methodology employed."
1	I suggest the author provide a scheme or flow diagram of their review process since the first stage of review includes a number of papers that were reviewed in the first stage (title and abstract) and so	Thank you for pointing this out. We have provided the PRISMA 2020 flow diagram of the study inclusion process as Figure 1.

	on. This diagram is very standard for a review paper, and it will be helpful for readers to understand the process of scoping review process.	
2	The methods are fairly well described but there are fundamental issues that weaken this paper. It would be important to address the issue of how the papers were assessed for quality for inclusion and exclusion. How was the issue of language beyond English addressed?<br><br>Another major question revolves around the measures of health outcomes included in this paper might not be exhaustive. There is significant work that has been done in Africa on the subnational burden of disease in Ethiopia, Kenya and South Africa on overall health inequalities and seems to be missing from your reporting.	Thank you for your comments and questions. We respond as listed below:<br><br>**Updated Methods Section**<br><br>To address the concerns raised, we have updated our methods section as follows:<br><br>**Methods**<br><br>We conducted a scoping review following the JBI Manual for Evidence Synthesis, to explore what evidence exists for the contribution of each driver of health inequalities in Africa. This was done following the methodology recommended in the Joanna Briggs Institute (JBI) Manual for Evidence Synthesis, a manual that provides guidance on developing systematic reviews [9] and was reported according to the PRISMA [10]. Our protocol was registered with the Open Science Framework (OSF) (identifier: https://osf.io/zd5bt), peer reviewed and published [11]. Given the comprehensive nature of the methods outlined in our protocol, we prioritized providing more detailed results in this manuscript and directing our readers to the protocol for further information on the methodology employed.<br><br>The electronic search strategy were conducted on MEDLINE, Embase, Web of Science, CINAHL, PyscINFO and Cochrane Library from their dates of inception to 29 august 2022, in line with this protocol to identify studies of interest. We included any empirical research that assessed inequalities in relation to services for reproductive, maternal, newborn and child health (e.g., family planning), infectious diseases (e.g., tuberculosis treatment) and non-communicable diseases (e.g., cervical cancer screening) in Africa [11]. We excluded retracted publications, conference abstracts, study protocols, and editorial materials. We also excluded studies with significant methodological flaws or those not directly relevant to health inequalities. Only studies

		published from January 1, 2005 to August 26, 2022, were considered. Our search strategy primarily included English-language articles. However, we utilized databases like Global Index Medicus to capture studies in multiple languages. We calculated an inter-reviewer agreement using the weighted Cohen's kappa [12].<br><br>**Quality Assessment of Included Papers**<br><br>In our scoping review, we followed the JBI Manual for Evidence Synthesis, which recommends that scoping reviews do not necessarily require a formal assessment of the methodological quality of included studies, as the primary aim is to map the existing literature rather than to assess the quality of evidence. However, we acknowledge the importance of quality assessment for providing context to our findings.<br><br>To address this, we performed preliminary appraisal of the included studies based on the following criteria:<br><br>● Study design<br>● Sample size<br>● Data collection methods<br>● Relevance to the research question<br><br>We ensured that studies with major methodological flaws or those not directly relevant to our research question were excluded. In future, we will provide more detailed descriptions of the quality assessment process in our methods section to enhance the transparency and rigor of our review.<br><br>**Language of Included Studies**<br><br>Our search strategy primarily included articles published in English, which is a limitation that we acknowledge. This was due to resource constraints and the predominant use of English in the databases we searched. However, we understand the importance of including studies published in other languages to provide a more comprehensive review.<br><br>To mitigate this limitation, we included studies indexed in databases that cover a wide range of languages, such as Global Index Medicus, which includes literature from various WHO regional databases, often in multiple languages.

		In future reviews, we aim to incorporate a broader range of languages by collaborating with multilingual researchers and utilizing translation services.<br><br>**Exhaustiveness of Health Outcomes**<br><br>We appreciate your observation regarding the comprehensiveness of health outcomes included in our review. Our inclusion criteria focused on articles examining inequalities in the utilization of health services for reproductive, maternal, newborn, and child health (RMNCH), as well as infectious and non-communicable diseases across Africa.<br><br>We recognize the importance of including studies on the subnational burden of disease, particularly in countries like Ethiopia, Kenya, and South Africa, where significant research has been conducted. To address this, we have undertaken the following steps:<br><br>• We performed an additional search to specifically capture studies on subnational health inequalities in these countries.<br>• We screened the references of identified studies to ensure we included relevant work on the subnational burden of disease.<br>• We included studies from national and regional health reports where applicable.<br><br>**Focus on health services**<br><br>We strongly agree that equity in financial protection is an essential component of UHC that needs to be assessed. However, to the best of our knowledge, published knowledge syntheses overviews of reviews (reviews of reviews) have prioritized financial protection over service coverage (Bhatia et al. 2022; http://dx.doi.org/10.1136/bmjopen-2021-052041). Furthermore, within the African region, health service coverage is one of the lowest. For this reason, the WHO Regional Office for Africa suggested focusing this review on use of health services, an area of unexplored potential.<br><br>**Literature search**

		UHC and equity represented broad concepts, but we made our search strategy comprehensive by including all "necessary/important/suggested" search terms and validated search filters to reflect three concepts: 1) equity, 2) universal health coverage, and 3) African regions. We have also attempted to expand our review to some sources, including publicly available information produced by all levels of government, academic institutions, business, and industry, in print and electronic formats, which are not controlled by commercial publishers. No restrictions were placed on date of publication, language, place of publication or type of reports in our electronic search strategy. Our search strategy was reviewed by an information specialist and our core research team of international experts in health equity, UHC, health information systems or knowledge syntheses from Africa will review this preliminary search strategy.
**Results**		
1	The percentage information the authors provided on the results and authors labeled as quantitative findings could have been clearer because the info presented mainly was percentage and descriptive. I suggest the author change the wording label<br><br>rather than using “quantitative findings.” There are similar comments for the “quantitative” figure as well. Such as Figure 1, the authors labeled Figure 1 as quantitative findings; I would instead call it the number of service utilization by equity stratifier, just like what the authors put on the diagram. I do not think this diagram is quantitative findings because whenever something is labeled as quantitative findings, the readers expect to see the statistical modeling results or at least the descriptive statistics. I have a similar comment with Figure 2, too. If the ‘quantitative findings’ in this paper imply the quantitative findings in the papers they reviewed, please	Thank you for your feedback. We understand that the term "quantitative findings" may have caused confusion, as it typically suggests detailed statistical analysis. To address this, we have revised the labels and descriptions in the manuscript to more accurately reflect the content.

	differentiate them. Later, I noticed that in some texts, the wording "quantitative findings" implies the quantitative method that the papers used.	
1	Place of residence: How many studies reported the urban-rural gap decline.	Thank you for your comments. We have conducted a thorough review of the sections you have highlighted and added the necessary citations to ensure consistency throughout the paper.
11	Gender: rather than "biological differences (men or women" I would say using gender differences is better, especially the authors labeled the subsection of Gender.	Thank you for your comment. We have revised the text to use "gender differences" instead of "biological differences (men or women)" to better align with the subsection title.
1	This sentence needs a citation "In South-South Nigeria; one study reported that although water, sanitation, and hygiene (WASH) services are poor for slum dwellers, women in slums bear the disproportionate burden of inadequate services due to the gendered distribution of childcare, household work and the need to fulfil the personal sanitary needs." Also, the rest of the sentence in the paragraph needs a citation.	Thank you for your comments. We have conducted a thorough review of the sections you have highlighted and added the necessary citations to ensure consistency throughout the paper.
1	Some things could be more consistent in providing citations. In some subsections, the authors provided citation numbers after "six studies (then citation numbers) including in the Age subsection." However, I found in another subsection, such as in the Gender subsection, "Fout of the quantitative studies examined…" no citations of these four studies. Also, in the Language subsection, "seven quantitative studies reported…" but no citations were provided. Please be consistent with how many numbers of studies you want to provide citations (I understand maybe less than ten?), and they must be consistent throughout the paper.	Thank you for your comments. We have conducted a thorough review of the sections you have highlighted and added the necessary citations to ensure consistency throughout the paper.

1	What is Plus? I am not sure “Plus” as subsection heading.	Thank you for your comment, the manuscript has been revised. “Plus” refer to other factors in which health inequalities may exist such as age or disability.
1	Overall: Each subsection (place of residence, education, gender, language/race/ethnicity, religion, occupation, social capital, age, and plus is mostly descriptive of several papers, whether reported quantitative or qualitative findings. I hope the authors can provide some helpful narrative or interpretation of the results, like what do the findings mean. Why do they matter? Why are they important for the African region? A bit of context and interpretation will be helpful rather than only reporting the number of studies found.	Thank you for your feedback. We have taken this feedback into consideration in the revisions that we have made. We have enhanced the discussion section to include more detailed narrative and interpretation of the findings.
2	The results section needs a lot of work if this paper is to be ready for publication. The information needs to be better organized. Lumping quantitative and qualitative results per inequality driver makes for difficult reading. It would be better to have distinct sections dealing with quantitative and qualitative results separately. Some of the results that are reported are insignificant and of no consequence and therefore can be eliminated so that the section is better structured.	Thank you for your feedback. We have made the results section clearer and easier to read by removing irrelevant results and making it more organized.
2	The figures are not fully labeled and therefore makes it difficult to know what is being presented in the results. It would be better to stick with reporting proportions in the figures so that it's not duplicative (numbers and proportions in the text).	Thanks for your feedback. We have revised the figures to include comprehensive labels and only show proportions to avoid duplication with the text.
**Discussion**		
1	Please check some grammatical errors in the text, including using capital letters and not capital letters in the middle of the sentence.	Thank you for your comment. We have reviewed the text and corrected the grammatical errors.

1	These sentences need citations, “For example, the review showed that structural and financial social support from spouses were crucial factors for women using ANC in Ghana.” Also, the following sentence needs citation, “In Ethiopia,…..”	Thank you for your comments. We have conducted a thorough review of the sections you have highlighted and added the necessary citations to ensure consistency throughout the paper.
1	I suggest discussing the strengths of the study, then followed by the limitations, not the other way around.	Thank you for your comment, we are now discussing the strengths of the study then the limitations.
2	The discussion could be strengthened by leaning on the health system framework as elaborated by the WHO and covering the different facets of inequality. For instance, the aspect of cost effectiveness and efficiency are missing from the discussion. A more detailed and directed literature review based on the results could strengthen the discussion. -- what are the health policy implications and future research? what are the limitations of this work that readers need to pay attention.	Thank you for your comment. The manuscript has been revised, including the implications for policy in the African region as well as future research recommendations.
**Others**		
1	Additional comment: I suggest the author mention whether the study included all papers from all countries in the African continent (including Arab-speaking countries in North Africa). I bring up this because Arab-speaking countries in North Africa culturally differ from the rest of Africa, and sometimes, from the regional standpoint, this part of Africa is better known as part of the Middle East. So, it is important to mention whether the paper includes studies from those countries in North Africa or not. If the paper included those countries, I just suggest the authors to mention it in the paper briefly, maybe somewhere in the introduction section.	Thank you for your comment. As detailed in the protocol, the paper considers any countries or geographic regions in the African continent such as the 47 member states of the WHO Regional Office for Africa, the Maghreb and all other African regions.

VERSION 2 - AUTHOR RESPONSE

Comment	Response
Please revise the formatting of your abstract so that it includes the following sections: Objectives >> Design >> Data Sources >> Eligibility Criteria >> Data extraction and synthesis >> Results >> Conclusions. Please see the following published Abstract as an example: https://bmjopen.bmj.com/content/12/2/e054120	Thank you for your comment. We revised the abstract to align with the required format, following the sections: Objectives, Design, Data Sources, Eligibility Criteria, Data Extraction and Synthesis, Results, and Conclusions, as per the guideline.
Please fully outline the methodology of your study in the Methods section. Readers should be able to understand the methods of your study without referring to the protocol.	Thank you for your valuable feedback. We have revised the Methods section to provide a comprehensive outline of our study's methodology, ensuring that readers can fully understand our methods without needing to refer to the protocol. This includes detailed descriptions of our search strategy, inclusion criteria, data abstraction processes, and the criteria used for assessing the quality of the studies.
Please include, as a supplementary file, the precise, full search strategy (or strategies) for all databases, registers and websites, including any filters and limits used.	Thank you for your constructive suggestion. We have included the precise and complete search strategy as a supplementary file (Appendix 1), detailing the specific strategies employed. We appreciate your feedback and believe that this addition enhances the transparency and reproducibility of our study.
Please add a Patient and Public Involvement statement to your manuscript (this is usually placed at the end of the main text Methods section). If there was no involvement, please state "None.". See our Instructions for Authors for further details: https://bmjopen.bmj.com/pages/authors/#reporting_patient_and_public_involvement_in_research	Thank you for your feedback. We have added a Patient and Public Involvement statement to the end of the Methods section, as recommended.
Please complete a thorough proofread of the text and correct any errors and English-language grammar and phrasing issues that you identify.	Thank you for your valuable feedback which has helped improve the quality of our manuscript. We have conducted a thorough proofreading of the manuscript and corrected any errors related to grammar and overall language clarity.
Please complete an updated search as the last search was conducted in 2022. Please also ensure you report all relevant searches in the manuscript to ensure transparency.	We recognize the importance of maintaining up to date data. However, the delay in revising this manuscript is largely due to the extended turnaround time for review, as the paper was first submitted on December 6, 2023. After reviewing a sample of the identified 1,257 additional new records, we determined that they do not materially alter the findings or conclusions we reached. For this reason, we would suggest that an additional

	search would not substantially improve the value of the manuscript.
Please include a statement relating to the ethical approval obtained for your study in the Methods section. If ethical approval was not required, please explain why this is the case.	Thank you for your comment. Ethical approval was not required as the review did not involve human participants or unpublished secondary data. We have added a statement clarifying this in the Methods section to ensure full transparency.
Please address the following comment as per the reviewer's request:<br><br>2. Place of residence: One of the authors even commented (on the comment box) "how many," implying that the manuscript they submitted still had an internal review by the authors. I agree to add how many studies reported the urban-rural gap has declined.	Thank you for your valuable comment. Two studies reported the urban-rural gap decline. This has been clarified in the manuscript.
Please include the below responses to the reviewers' comments in the manuscript:<br><br>What did 'A rising tide lifts all boats' mean in this context?<br><br>Response: Thank you for your comment. In this context, the phrase "a rising tide lifts all boats" is used to highlight the importance of distinguishing between improvements that are due to specific interventions and those that are due to broader, unrelated factors. For example, an improving economy may lead to better health outcomes. Therefore, the health sector should not claim credit for health improvements that are actually a consequence of economic growth. The rising tide (improving economy) lifts all boats (health, education, gender equality, etc.), meaning that different sectors may benefit simultaneously from general economic improvements rather than from sector-specific interventions.<br><br>Was there any specific reason to only review articles published from January 1, 2005, to August 29, 2022? How about papers published before 2005? It is imperative to justify the cut-off time of papers in this time frame.<br><br>Response: Thank you for your comment. The timeframe was narrowed to January 1, 2005, to August 29, 2022, because 2005 marks the first time of the mention of UHC into public health discourse.<br><br>*The methods are fairly well described but there are fundamental issues that weaken this paper. It would be	Thank you for your comment regarding the phrase "a rising tide lifts all boats." After careful consideration, we have decided to remove this sentence from the manuscript. We believe that its removal enhances the clarity of the manuscript.<br><br>Thank you for your comment. We have added clarification to the manuscript to justify our decision to focus on articles published from January 1, 2005, to August 29, 2022.<br><br>Thank you for your constructive feedback on the methods section.

important to address the issue of how the papers were assessed for quality for inclusion and exclusion. How was the issue of language beyond English addressed?<br><br>Another major question revolves around the measures of health outcomes included in this paper might not be exhaustive. There is significant work that has been done in Africa on the subnational burden of disease in Ethiopia, Kenya and South Africa on overall health inequalities and seems to be missing from your reporting.<br><br>Response:<br><br>Quality Assessment of Included Papers<br><br>In our scoping review, we followed the JBI Manual for Evidence Synthesis, which recommends that scoping reviews do not necessarily require a formal assessment of the methodological quality of included studies, as the primary aim is to map the existing literature rather than to assess the quality of evidence. However, we acknowledge the importance of quality assessment for providing context to our findings.<br><br>To address this, we performed preliminary appraisal of the included studies based on the following criteria:<br><br>● Study design<br><br>● Sample size<br><br>● Data collection methods<br><br>● Relevance to the research question<br><br>We ensured that studies with major methodological flaws or those not directly relevant to our research question were excluded. In future, we will provide more detailed descriptions of the quality assessment process in our methods section to enhance the transparency and rigor of our review.<br><br>Language of Included Studies<br><br>Our search strategy primarily included articles published in English, which is a limitation that we acknowledge. This was due to resource constraints and the predominant use of English in the databases we searched. However, we understand the importance of including studies published in other languages to provide a more comprehensive review.<br><br>To mitigate this limitation, we included studies indexed in databases that cover a wide range of languages, such as Global Index Medicus, which	We have clarified all relevant points, including the processes for assessing the quality of papers for inclusion and exclusion. This information has been detailed in the revised Methods section to ensure transparency in our approach. Additionally, we have addressed how we managed language diversity in our review in the Methods section. We believe these clarifications strengthen the rigor and comprehensiveness of our methodology, and we appreciate your guidance in highlighting these important aspects.<br><br>We also thank you for raising this important point regarding the measures of health outcomes in our paper. In response to your concern, we have expanded our discussion in the Methods section to clarify the steps we undertook to ensure a comprehensive review.<br><br>Regarding our literature search, we have clarified in the manuscript that our search strategy was comprehensive, incorporating all necessary/important/suggested search terms and validated filters to reflect the key concepts of equity, UHC, and African region.<br><br>We also provided more information regarding our search strategy that was by our core research team of international experts in health equity, UHC, health information systems, or knowledge syntheses from Africa. We appreciate your suggestions, which have contributed to improving the clarity and rigor of our methodology.

includes literature from various WHO regional databases, often in multiple languages. In future reviews, we aim to incorporate a broader range of languages by collaborating with multilingual researchers and utilizing translation services.<br><br>Exhaustiveness of Health Outcomes<br><br>We appreciate your observation regarding the comprehensiveness of health outcomes included in our review. Our inclusion criteria focused on articles examining inequalities in the utilization of health services for reproductive, maternal, newborn, and child health (RMNCH), as well as infectious and non-communicable diseases across Africa.<br><br>We recognize the importance of including studies on the subnational burden of disease, particularly in countries like Ethiopia, Kenya, and South Africa, where significant research has been conducted. To address this, we have undertaken the following steps:<br><br>● We performed an additional search to specifically capture studies on subnational health inequalities in these countries.<br><br>● We screened the references of identified studies to ensure we included relevant work on the subnational burden of disease.<br><br>● We included studies from national and regional health reports where applicable.<br><br>Focus on health services<br><br>We strongly agree that equity in financial protection is an essential component of UHC that needs to be assessed. However, to the best of our knowledge, published knowledge syntheses overviews of reviews (reviews of reviews) have prioritized financial protection over service coverage (Bhatia et al. 2022; http://dx.doi.org/10.1136/bmjopen-2021-052041). Furthermore, within the African region, health service coverage is one of the lowest. For this reason, the WHO Regional Office for Africa suggested focusing this review on use of health services, an area of unexplored potential.<br><br>Literature search<br><br>UHC and equity represented broad concepts, but we made our search strategy comprehensive by including all "necessary/important/suggested" search terms and validated search filters to reflect three concepts: 1)	

equity, 2) universal health coverage, and 3) African regions. We have also attempted to expand our review to some sources, including publicly available information produced by all levels of government, academic institutions, business, and industry, in print and electronic formats, which are not controlled by commercial publishers. No restrictions were placed on date of publication, language, place of publication or type of reports in our electronic search strategy. Our search strategy was reviewed by an information specialist and our core research team of international experts in health equity, UHC, health information systems or knowledge syntheses from Africa will review this preliminary search strategy.<br><br>*This section needs proofreading and better elaboration. The literature review here needs to be strengthened. Has this kind of review been done elsewhere? What is different with your work? what additional value does your work present? This could include work done in other regions so that the reader appreciates that your work is comparable to others and that there is added value.<br><br>Response: Thank you for your comment. Few reviews have specifically examined inequalities in the context of universal health coverage. The limited reviews that have been conducted have focused primarily on specific services, such as reproductive, maternal, newborn, and child health (RMNCH) services. In addition, other reviews have assessed disparities using selected stratifiers, such as socioeconomic status and age.<br><br>In this review, we aim to consolidate the evidence on inequalities in service coverage in Africa using a comprehensive set of stratifiers. These stratifiers include, but are not limited to, socioeconomic status, age, gender, geographic location, and ethnicity. Our approach provides a more holistic understanding of the different dimensions of inequality in the context of UHC.	Thank you for your feedback. We have clarified in the Introduction that reviews have been conducted primarily focusing on specific services, such as reproductive, maternal, newborn, and child health services or have assessed disparities using selected stratifiers, such as socioeconomic status and age.<br><br>In the Abstract, we have also clearly outlined that the objective of this scoping review is to consolidate the evidence on inequalities in service coverage in Africa using a comprehensive set of stratifiers. We appreciate your input, which has helped enhance the clarity and focus of our manuscript.

VERSION 3 - AUTHOR RESPONSE

Comment	Response
Please review the manuscript for any instances of repetition such as: “The search strategy was rigorously reviewed by an information specialist, and our core research team. Our search strategy was reviewed by an information specialist and our core research team of international experts in health equity, UHC, health information systems or knowledge syntheses from Africa reviewed the preliminary search strategy to ensure its thoroughness and relevance.”	Thank you for highlighting the repetition in this section. We have revised the manuscript. The updated sentence now reads: “Our search strategy was reviewed by an information specialist and our core research team of international experts in health equity, UHC, health information systems or knowledge syntheses from Africa reviewed the preliminary search strategy to ensure its thoroughness and relevance”.
Please clarify where this change has been made in the manuscript: "Two studies reported the urban-rural gap decline. This has been clarified in the manuscript."	Thank you for your comments. In response to the inquiry regarding studies on the decline in the urban-rural gap, we reviewed the relevant section and added citations to ensure consistency across the paper. This update is reflected on page 8.
Please ensure that you have fully discussed the methodological limitations of the study in the Discussion section of the main text.	Thank you for this valuable comment. In response, we have expanded the Discussion section to fully outline the methodological limitations of our study. Specifically, we have highlighted limitations in our search strategy, including the predominance of English-language articles due to resource constraints and the language availability in databases. Additionally, we addressed the limitations related to adapting the WHO UHC service coverage indicators, which constrained our ability to assess inequalities in service quality, as these indicators focus primarily on utilization. These additions can now be found in the final paragraph of the revised Discussion section.
